# Pyridine-promoted dediazoniation of aryldiazonium tetrafluoroborates: Application to the synthesis of SF_5_-substituted phenylboronic esters and iodobenzenes

**DOI:** 10.3762/bjoc.11.162

**Published:** 2015-08-26

**Authors:** George Iakobson, Junyi Du, Alexandra M Z Slawin, Petr Beier

**Affiliations:** 1 Institute of Organic Chemistry and Biochemistry, Academy of Sciences of the Czech Republic, Flemingovo nám. 2, 166 10 Prague 6, Czech Republic; 2 EaStCHEM School of Chemistry, University of St Andrews, St Andrews, KY16 9ST, United Kingdom

**Keywords:** borylation, diazonium salts, iodination, pyridine, sulfur pentafluorides

## Abstract

Pyridine promotes dediazoniation of aryldiazonium tetrafluoroborates. The formed aryl radicals were trapped with B_2_pin_2_, iodine, or tetrahydrofuran to afford boronic esters, iodobenzenes and benzenes, respectively. The application to the synthesis of (pentafluorosulfanyl)phenylboronic esters, iodo(pentafluorosulfanyl)benzenes and (pentafluorosulfanyl)benzene is shown.

## Introduction

Pentafluorosulfanyl-containing compounds have been known for more than half a century [1–4]; however, for a long time they remained a relatively underdeveloped class of compounds [5–6]. The main reason for the slow development of the chemistry of SF_5_-containing compounds was the lack of availability of key building blocks. However, in recent years, the scientific community has been witnessing a renewed interest in this functional group. Synthetic methods towards aliphatic SF_5_-containing compounds are based on free radical addition of SF_5_Cl or SF_5_Br to unsaturated compounds [7–9], whereas aromatic derivatives are available either by the Umemoto’s two-step synthesis from diaryl disulfides or benzenethiols [10–12], or by the reaction of nitrophenyl disulfides with elemental fluorine [13–16]. Aromatic and heteroaromatic SF_5_ compounds are mostly prepared by the derivatization of commercial nitro-(pentafluorosulfanyl)benzenes [14,17–27] and approaches from SF_5_-aliphatics have also been studied [28–30]. The unique combination of properties the SF_5_ group imparts includes high chemical, thermal, and metabolic stability, strong electron-acceptor property, and high lipophilicity. Furthermore, applications of SF_5_ compounds in catalysis [31–32], life-science [6,18,33–38], and material sciences [5,19,38–39] are emerging.

Arylboronic acids and arylboronates represent versatile building blocks in organic synthesis [40]. They have found wide applications in transition metal-catalyzed cross-coupling reactions [41–42]. These boron compounds are accessed mainly by the reactions of arylmagnesium or aryllithium species with trialkylboronates [43–44], Pd- or Cu-catalyzed borylations of aryl halides using B_2_pin_2_, H-Bpin [45–50] or R_2_N-BH_2_ [51], direct borylations via aromatic C–H bond activations [52–58], Lewis acid catalyzed electrophilic borylations of electron-rich arenes [59–62], and Sandmeyer-type borylation of arylamines or diazonium salts with B_2_pin_2_ [63–67], B_2_(OH)_4_ [68] or R_2_N-BH_2_ [69]. Several attempts were made to synthesize the SF_5_-phenylboronates. Patent literature describes the synthesis of 3- or 4-(pentafluorosulfanyl)phenylboronates or boronic acids from SF_5_-bromobenzenes via lithiation or magnesiation. These approaches suffer from low yields and other drawbacks [70–71]. For lithiation of the aryl bromide, *t*-BuLi had to be used and the formation of Grignard reagents is inefficient. On the other hand, Shibata and co-workers have recently reported the synthesis of 3,5-bis(pentafluorosulfanyl)phenylboronic acid from the corresponding aryl bromide, trimethyl borate and iPrMgBr [32]. Finally, Joliton and Carreira have recently shown efficient Ir-catalyzed C–H borylation of several 1-substituted-3-(pentafluorosulfanyl)benzenes and applied the products of borylation to the Pd-catalyzed Suzuki–Miyaura reaction with aryl bromides or iodides. However, the reaction is limited to borylations in position five of 1-substituted-3-(pentafluorosulfanyl)benzenes [72].

Straightforward access to SF_5_-phenylboronic acids or boronates would be highly desirable since it would allow easy installation of the SF_5_-phenyl group by the subsequent Suzuki–Miyaura reaction. Nitro-(pentafluorosulfanyl)benzenes are the primary industrial SF_5_-aromatics, therefore the easiest access to SF_5_-phenylboronates appears to be starting from readily available SF_5_-substituted anilines or diazonium salts rather than SF_5_-containing halobenzenes. Herein, we report a new protocol for efficient borylation, iodination and hydrodediazoniation of SF_5_-phenyldiazonium tetrafluoroborates in the presence of pyridine. The generality of the borylation and iodination reactions was demonstrated on several examples.

## Results and Discussion

At the onset of our investigation, Sandmeyer-type borylation of 3- and 4-(pentafluorosulfanyl)anilines (**1a** and **1b**, respectively) to pinacolboronates **2a** and **2b** according to Wang and co-corkers was studied [64–65] (Table 1). The borylation of **1a** took place in a reasonable yield in the presence of catalytic amounts of benzoyl peroxide (BPO, Table 1, entry 1), while for **1b**, heating without any additives was preferable; however, the yield of **2b** was only moderate (Table 1, entry 3).

**Table 1:** Synthesis of boronates **2** from aniline derivatives **1**^a^.

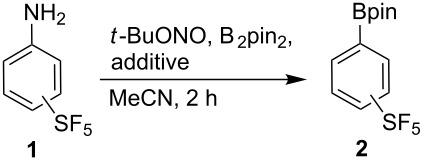				

Entry	**1**	Additive	Temp. (°C)	**2**, Yield (%)^b^

1	**1a**, 3-SF_5_	BPO	rt	**2a**, 70
2	**1b**, 4-SF_5_	BPO	rt	**2b**, traces
3	**1b**, 4-SF_5_	—	80	**2b**, 55^c^

^a^Reaction conditions: **1** (1 mmol), *t*-BuONO (1.5 mmol), B_2_pin_2_ (1.1 mmol), additive (2 mol %), MeCN (4 mL). ^b^Isolated yield. ^c^82% purity.

For a detailed investigation of the borylation reaction, the diazonium tetrafluoroborates **3a** and **3b** were prepared and isolated according to Doyle conditions [73]. High yields (above 90%) of both isomers of the diazonium tetrafluoroborates **3a** and **3b** were obtained on 0.3–6 g scale (Table 2). Crystal structures of **3a** (CCDC 1009848) and **3b** (CCDC 1009849) were determined confirming the nature of the products. During the course of our studies, Okazaki and co-workers reported the synthesis of **3b** in 84% yield under similar conditions and have shown its reactivity in various cross-coupling reactions with varied degree of success. The most efficient cross-coupling reactions were the Heck reactions with alkenes, a biaryl homocoupling reaction, an azo coupling to electron-rich arenes, and a dediazoniation with TMSN_3_ in an ionic liquid medium [74–75].

**Table 2:** Synthesis of diazonium tetrafluoroborates **3**^a^ and boronates **2**^b^.

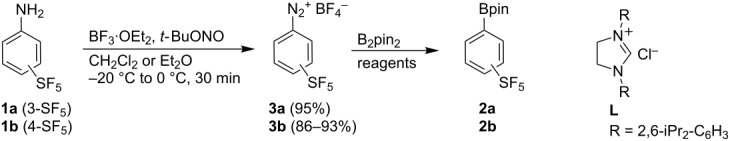					

Entry	**3** (mmol)	Reagents (equiv), solvent	Temp. (°C)	Time (h)	**2**, Yield (%)^c^

1	**3a** (1)	Pd(OAc)_2_ (0.01), **L** (0.02), THF	rt	18	**2a**, 43
2	**3b** (1)	Pd(OAc)_2_ (0.01), **L** (0.02), THF	rt	18	**2b**, 57
3	**3a** (1)	NaOAc (2), MeCN	−50 to rt	3	**2a**, 73
4	**3b** (1)	NaOAc (2), MeCN	−50 to rt	3	**2b**, 49
5	**3b** (1)	CuBr (0.05), MeCN/H_2_O (3:1)	rt	330	**2b**, 23
6	**3a** (1)	—, MeOH	rt	24	**2a**, 32
7	**3b** (1)	—, pyridine^d^	−30 to rt	2	**2b**, 55
8	**3b** (1)	pyridine (4), MeCN^d^	−30 to rt	2	**2b**, 77
9	**3b** (3)	pyridine (4), MeCN^d^	−30 to rt	2	**2b**, 77
10	**3a** (2)	pyridine (4), MeCN^d^	−30 to rt	2	**2a**, 80

^a^Reaction conditions: **1** (1–28 mmol), BF_3_·OEt_2_ (2.1 equiv), *t*-BuONO (1 equiv), CH_2_Cl_2_ or Et_2_O (3 mL/1 mmol of **1**), 30 min. ^b^Reaction conditions: **3** (1–3 mmol), B_2_pin_2_ (1 equiv), reagents, solvent (2 mL/1 mmol of **3**) under N_2_. ^c^Isolated yield. ^d^The reaction was conducted under air.

An efficient borylation of aryldiazonium tetrafluoroborates with NHC-Pd catalysts was reported recently [63]. When applied to **3a** and **3b** using Pd(OAc)_2_ and NHC ligand precursors **L**, the borylated products **2a** and **2b** were isolated in only moderate yields (Table 2, entries 1 and 2). However, it was found that the Pd catalyst was not required for an efficient reaction. Alkali metal acetates are known to facilitate decomposition of aryldiazonium salts by the formation of diazoacetates and diazo anhydrides, which decompose to aryl radicals [76]. These additives were used in Meerwein arylation of isopropenyl acetate [77]. In our case, two-fold excess of sodium acetate in acetonitrile afforded **2a** and **2b** in good and moderate yields, respectively (Table 2 ,entries 3 and 4). The conditions reported by Yu and co-workers [66] (Table 2, entry 5) provided a mixture with starting **3b** as the major component. While the diazonium salts **3a** and **3b** were found to be stable in acetonitrile, we observed slow decomposition in methanol and in the presence of B_2_pin_2_ under strictly Pd-free conditions (new Teflon stirring bar and glassware), the borylation took place with low conversion (Table 2, entry 6) [78]. In pyridine, however, the decomposition of **3b** was very fast and a vigorous evolution of nitrogen was observed affording the borylated product in a moderate yield together with a mixture of SF_5_-pyridines in ca. 10% GC–MS yield (Table 2, entry 7). Finally, the use of a 4-fold excess of pyridine in acetonitrile was found to be optimal. Conducting the reaction on a gram scale proceeded without a notable loss of efficiency and the reaction can be performed in air (Table 2, entries 8–10).

**Scheme 1:**
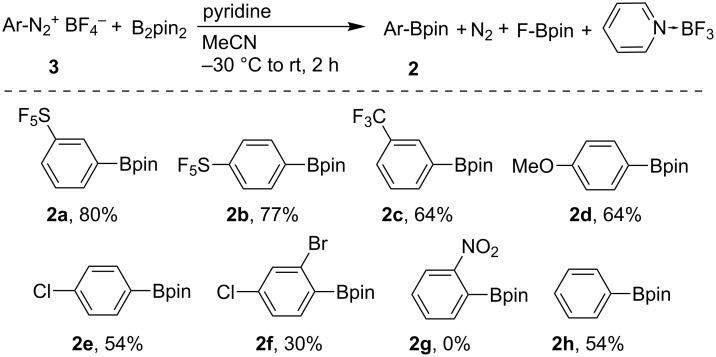
Borylation of aryldiazonium tetrafluoroborates **3**. Reaction conditions: **3** (1 mmol), B_2_pin_2_ (1 mmol), pyridine (4 mmol), MeCN (2 mL), 2 h.

Pyridine is known to induce decomposition of aryldiazonium salts. Zollinger and Abramovitch studied the interaction of pyridine with aryldiazonium tetrafluoroborates and suggested the formation of diazopyridinium salts which homolytically decompose to aryl radicals, nitrogen and a pyridinium tetrafluoroborate radical [79–80]. Tanaka and co-workers have used the combination of PhN_2_^+^BF_4_^−^ and pyridine for arylations of silylenol ethers [81]. They have observed the formation of large amounts of phenylpyridines. In our case, we detected SF_5_-phenylpyridines in trace amounts only during borylation using pyridine as a solvent. The major products in the borylation reaction apart from **2a** and **2b** were found to be F-Bpin and pyridine·BF_3_ complexes. Both compounds are easily hydrolyzable but they were observed by NMR of the crude reaction mixture and compared to the synthetized authentic samples. On the other hand, we were not able to observe a pyridine·B_2_pin_2_ complex by ^11^B NMR in CD_3_CN or CDCl_3_. The borylation was extended to several other aryldiazonium tetrafluoroborates showing that both electron-donor and electron-acceptor substituted phenyldiazonium tetrafluoroborates undergo efficient borylation with an equimolar amount of B_2_pin_2_ (Scheme 1); however, *ortho*-substituted phenyldiazonium salts were found to be either not efficient substrates (**3f**) or completely unreactive (**3g**), presumably due to a large steric demand of B_2_pin_2_.

**Scheme 2:**
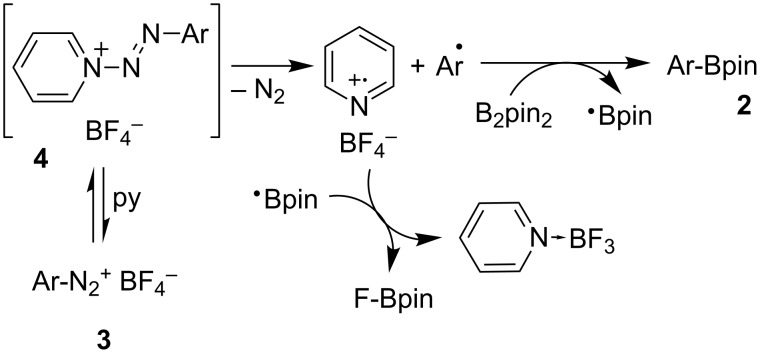
Proposed reaction mechanism.

The mechanism of this borylation reaction remains to be elucidated. Based on experimental results and literature precedent, we propose the following free-radical mechanism (Scheme 2). Aryldiazonium salt **3** reacts with pyridine to form aryldiazopyridinium **4** which decomposes to an aryl radical, a pyridinium tetrafluoroborate radical and nitrogen. The aryl radical reacts with B_2_pin_2_ to form the borylated product **2** and the Bpin radical (likely to be stabilized by pyridine) [82]. The byproducts pyridine·BF_3_ and F-Bpin are formed by the reaction of the pyridinium tetrafluoroborate radical and the Bpin radical.

**Scheme 3:**
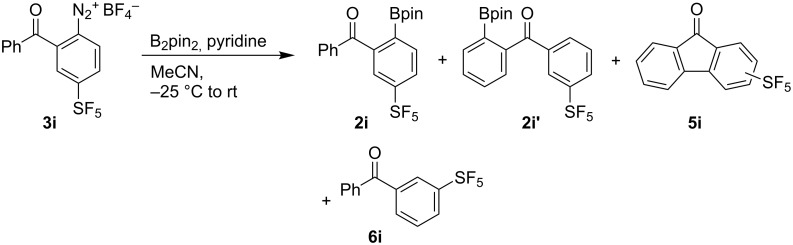
Reaction of diazonium salt **3i** under borylation conditions.

Subjecting the diazonium salt **3i** to the borylation conditions gave further indirect evidence for the formation of aryl radicals (Scheme 3). Full conversion of **3i** was observed affording a mixture of products **2i**, **2i’**, **5i** (two isomers of unknown configuration) and **6i** in 16:31:28:25 GC–MS ratio. The presence of these products can be explained only by the formation of the substituted SF_5_-phenyl radical which undergoes borylation to **2i**, hydrogen atom transfer followed by borylation to **2i’**, intramolecular cyclization to **5**i or hydrogen abstraction to **6i**.

Starting from aniline derivative **1b**, a one pot diazotization–borylation sequence using different acids afforded the corresponding borylated product **2b** in good yields (Table 3).

**Table 3:** One pot diazotization-borylation of **1b**^a^.

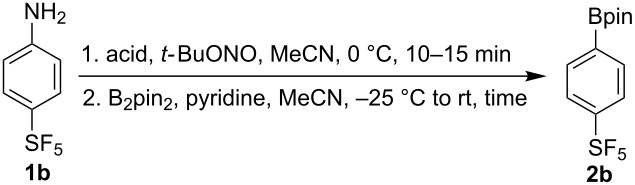			

Entry	Acid (equiv)	Time (h)	**2b**, Yield (%)^b^

1^c^	*p*-TsOH·H_2_O (1)	15	51
2	aq HBF_4_ (1.7)	1	81
3	aq HCl (1.7)	1	78

^a^Reaction conditions: **1b** (1 mmol), acid, *t*-BuONO (1 mmol), MeCN (3 mL), B_2_pin_2_ (1.0–1.1 mmol), pyridine (4 mmol). ^b^Isolated yield. ^c^Reaction temperature for steps 1 and 2 was rt.

**Scheme 4:**
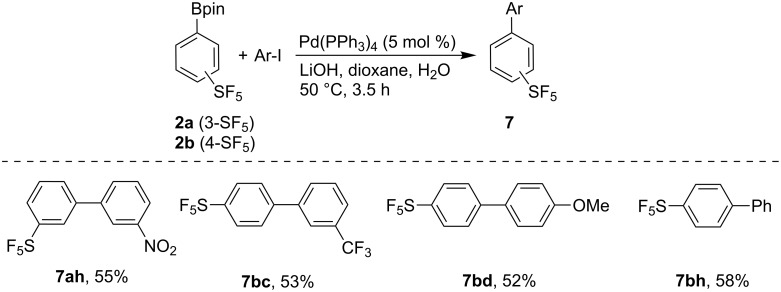
Suzuki–Miyaura reaction of boronates **2a** and **2b** with aryl iodides. Reaction conditions: **2** (1 mmol), ArI (1.1 mmol), Pd(PPh_3_)_4_ (5 mol %), LiOH·H_2_O (4 mmol), 1,4-dioxane (2 mL), water (1 mL), 3.5 h.

Suzuki–Miyaura cross-coupling reactions of boronates **2a** and **2b** with aryl iodides using a simple system without any optimization proceeded in satisfactory yields considering the electron-deficient character of the boronates and consequently less efficient transmetallation step (Scheme 4).

**Scheme 5:**
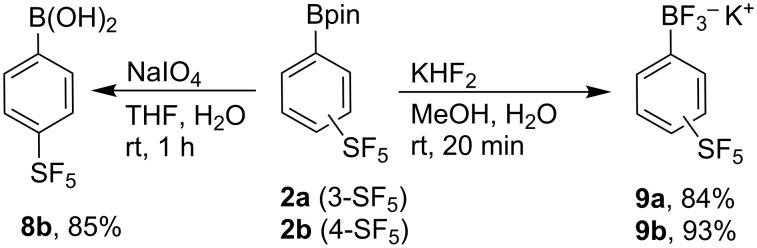
Syntesis of boronic acid **8b** and trifluoroborates **9**. Reaction conditions for the synthesis of **8b**: **2** (2 mmol), NaIO_4_ (8 mmol), THF (8 mL), H_2_O (2 mL), rt, 1 h. Reaction conditions for the synthesis of **9**: **2** (2 mmol), KHF_2_ (10 mmol), MeOH (8 mL), H_2_O (3.6 mL), rt, 20 min.

Transformation to SF_5_-phenylboronic acid **8b** and potassium trifluoroborates **9** was straightforward under standard conditions (Scheme 5). Similar potassium SF_5_-phenyltrifluoroborates were found to be highly reactive with a variety of aryl bromides and iodides in the presence of catalytic amounts of PdCl_2_(dppf)·CH_2_Cl_2_ or Pd(OAc)_2_ [72]. The recently published synthesis of arylboronic acids from anilines or aryldiazonium tetrafluoroborates using B_2_(OH)_4_ [68] applied to **3b** provided **8b** in only 25% ^19^F NMR yield.

**Scheme 6:**
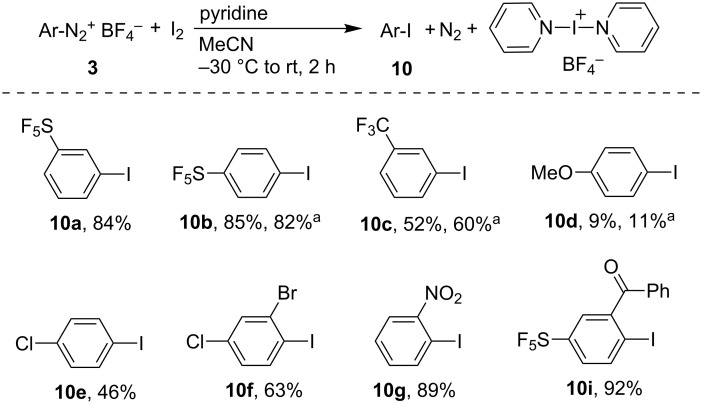
Iodination of aryldiazonium tetrafluoroborates **3**. Reaction conditions: **3** (1 mmol), I_2_ (1.1 mmol), pyridine (4 mmol), MeCN (3 mL), 2 h. ^a^Yields in the presence of collidine instead of pyridine.

To extend the synthetic utility of the pyridine-mediated derivatization of aryldiazonium tetrafluoroborates we investigated the reaction with iodobenzene and iodine as efficient scavengers of aryl radicals [83–84]. A competitive experiment starting from **3a** and equimolar amounts of B_2_pin_2_ and iodobenzene in the presence of pyridine (4 equiv) in MeCN afforded a mixture of **2a** (72% yield) and 1-iodo-3-(pentafluorosulfanyl)benzene (**10a**, 23% yield). Additionally, a reaction of **3b** with PhI (4 equiv) in the absence of B_2_pin_2_ gave 1-iodo-4-(pentafluorosulfanyl)benzene (**10b**) in 35% yield. Both experiments point to the formation of aryl radicals during the reaction and suggest a possibility to conduct practical aromatic iodination. Indeed, the iodination reaction with I_2_ proved to be more efficient than with PhI (Scheme 6). In contrast to borylation, iodination with I_2_ shows a higher sensitivity to electronic properties of substituents on the aromatic ring. Electron-acceptor substituted aryldiazonium compounds are excellent substrates while those with electron-donor groups react much less efficiently. The substitution of pyridine with collidine (2,4,6-trimethylpyridine) gave similar yields. Unlike borylations, the iodination reactions were not sensitive to *ortho* substitutions. In the case of **3i**, compound **10i** was the sole product; no product of hydrogen atom transfer or cyclization was observed, demonstrating that the reaction with I_2_ is much faster than with B_2_pin_2_. Importantly, the yields of SF_5_-phenyl iodides **10a** and **10b** using our two-step diazotization–iodination method significantly exceed those obtained by classical Sandmeyer reaction (ca. 80% yield over two steps compared to 63% for **10a** and 50% for **10b** by one-pot Sandmeyer procedure requiring 10 fold excess of KI) [14]. The side-product in the iodination of **3** was bis(pyridine)iodonium tetrafluoroborate, which can be easily isolated from the reaction mixture by precipitation upon addition of diethyl ether. This iodonium salt was first synthetized by Barluenga [85] and later used for mild iodination of alkenes, alkynes and aromatics [85–87]. Its formation can be explained by the reaction of the pyridinium tetrafluoroborate radical (Scheme 2), pyridine and I_2_ or iodine radical. Bromination of **3b** with Br_2_ was attempted under conditions similar to iodination but the reaction was slow and inefficient resulting in a mixture of products with expected 1-bromo-4-(pentafluorosulfanyl)benzene as a minor product. With equimolar NBS instead of bromine, the reaction is much cleaner but slow; after overnight at ambient temperature the bromo product was isolated in 35% yield.

Finally, hydrodediazoniation using tributyltin hydride or THF was tested and THF proved to be a more efficient hydrogen atom donor. The addition of excess pyridine to MeCN/THF solution of diazonium tetrafluoroborates **3a** or **3b** led to an efficient hydrodediazoniation and the formation of (pentafluorosulfanyl)benzene (**6**). Deuteration experiments established that the hydrogen atom in the product comes exclusively from THF and not from pyridine or MeCN (Table 4). The observed deuterium enrichment using THF-*d*_8_ was around 80%. Thermal decomposition of aryldiazonium salts prepared from immobilized triazene precursors and the formation of deuterated aromatics using THF-*d*_8_ was reported [88]. We explain the reduced yield of **6**-D (48% yield) and the formation of tar products by hydrogen atom abstraction from **3** or **6** and subsequent polymerization. No significant amounts of double deuterated products were detected. The kinetic isotope effect was determined from intermolecular competition experiment using **3b** and a 1:1 mixture of THF and THF-*d*_8_ giving KIE = 5.5 (**6**:**6**-D ratio determined by GC–MS) and combined yield of 62%. This means that the hydrogen abstraction is much faster than the deuterium abstraction and suggests the C-H(D) bond formation as the rate-limiting step. For unambiguous identification of the rate-limiting step, individual rate constants *k*_H_ and *k*_D_ in two parallel reactions would have to be determined [89]. Dihydrofuran (**11**) and pyridinium tetrafluoroborate were identified as byproducts of the dediazoniation reactions. Similarly to the previous processes, we presume the formation of aryldiazopyridinium **4** and its decomposition to an aryl radical and the pyridinium tetrafluoroborate radical. The aryl radical abstracts a hydrogen atom from THF forming **6** and a THF radical. The THF radical then transfers the hydrogen atom to the pyridinium tetrafluoroborate radical giving dihydrofuran (**11**) and pyridinium salt.

**Table 4:** Hydrodediazoniation of **3a** and **3b** with THF^a^.

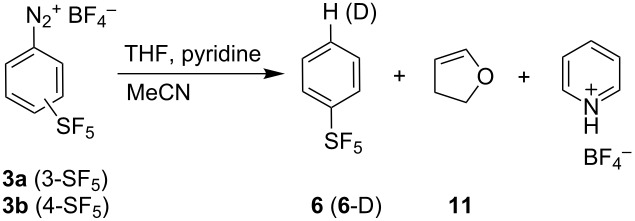				

Entry	**3** (mmol)	D source	D enrichment^b^	**6**, Yield (%)^c^

1	**3a** (0.5)	—	n/a	**6**, 75
2	**3b** (0.5)	—	n/a	**6**, 70
3	**3b** (0.5)	THF-*d*_8_ (0.5 mL)	77–82	**6**-D, 48
4	**3b** (0.25)	C_5_D_5_N (4 mmol)	0	**6**, 70
5	**3b** (0.25)	CD_3_CN (0.75 mL)	0	**6**, 67

^a^Reaction conditions: **3** (0.25–0.5 mmol), pyridine (4 equiv), THF (1 mL/1 mmol of **3**), MeCN (3–4 mL/1 mmol of **3**), 2 h. ^b^Based on GC–MS. ^c^Based on ^19^F NMR using 1-nitro-4-(pentafluorosulfanyl)benzene as an internal standard.

## Conclusion

In conclusion, a novel dediazoniation–borylation methodology was developed based on the reaction of aryldiazonium tetrafluoroborates with pyridine and B_2_pin_2_ to give arylpinacolborates. Particular emphasis was on the synthesis of SF_5_-phenylboronates where our methodology represents a considerable improvement in reaction efficiency compared to previously published syntheses. Furthermore, no transition metals are needed and mild reaction conditions are used. The borylation is applicable to a variety of aryldiazonium tetrafluoroborates with electron-donor or acceptor groups while *ortho*-substituted substrates are less reactive. A mechanism involving aryl radicals is suggested. The Suzuki–Miyaura reaction of SF_5_-phenylboronates with aryl iodides provided the cross-coupling biaryl products. In analogy to the borylation reaction, iodination of aryldiazonium tetrafluoroborates with pyridine and iodine resulted in aryl iodides. An efficient reaction was observed with electron-acceptor substituted aromatic compounds even with *ortho*-substituted derivatives. In the case of SF_5_-substituted iodobenzenes, the method is much more efficient than the classical Sandmeyer reaction starting from SF_5_-containing aniline derivatives. Finally, hydrodediazoniation of SF_5_-phenyldiazonium tetrafluoroborates by hydrogen atom abstraction from THF in the presence of pyridine provided (pentafluorosulfanyl)benzene.

## Supporting Information

Synthesis and characterization of all products, copies of ^1^H, ^13^C, and ^19^F NMR spectra of newly synthesized products, and X-ray crystallographic files of the compounds **3a** and **3b**.

File 1 Experimental part.

File 2 Crystal structure of compound **3a**.

File 3 Crystal structure of compound **3b**.
