# Supplementary material for: Pyridine-promoted dediazoniation of aryldiazonium tetrafluoroborates: Application to the synthesis of SF5-substituted phenylboronic esters and iodobenzenes
Source: Beilstein J Org Chem. 2015 Aug 26;11:1494–502. doi: 10.3762/bjoc.11.162 (PMC4578358; doi:10.3762/bjoc.11.162)
Supplement: File 1 — Experimental part. [file Beilstein_J_Org_Chem-11-1494-s001.pdf]

# Supporting Information
for
# Pyridine-promoted dediazoniation of aryldiazonium tetrafluoroborates: Application to the synthesis of $SF_5$-substituted phenylboronic esters and iodobenzenes

George Iakobson[1], Junyi Du[2], Alexandra M. Z. Slawin[2] and Petr Beier*[1]

Adresses: [1]Institute of Organic Chemistry and Biochemistry, Academy of Sciences of the Czech Republic, Flemingovo nám. 2, 166 10 Prague 6, Czech Republic and [2]EaStCHEM School of Chemistry, University of St Andrews, St Andrews, KY16 9ST, United Kingdom
Email: Petr Beier - beier@uochb.cas.cz
* Corresponding author

## Experimental part

### Table of Contents
General information S1
Synthesis of aryldiazonium tetrafluoroborates **3** S2
Synthesis of arylboronates **2** S4
Synthesis of biaryls **7** by Suzuki coupling reaction, General procedure S9
Synthesis of 4-(pentafluorosulfanyl)phenylboronic acid (**8b**) S10
Synthesis of potassium (pentafluorosufanyl)phenyltrifluoroborates **9** S11
Trapping experiment, pyridine-promoted dediazoniation in the presence of iodobenzene S12
Competitive experiment, pyridine-promoted dediazoniation-borylation vs. –iodination in the presence of $B_2pin_2$ and iodobenzene S12
Synthesis of aryl iodides **10**, General procedure S12
Synthesis of 1-bromo-4-(pentafluorosulfanyl)benzene S14
Synthesis of (pentafluorosulfanyl)benzenes **6** by hydrodediazoniation of aryldizaonium tetrafluoroborates, General procedure S14
KIE experiment S15
Copies of NMR spectra S17
Crystal structure of **3a** S131
Crystal structure of **3b** S132
References S133

### General information

NMR spectra were recorded at 25 °C in $CD_2Cl_2$, $CDCl_3$, $[D_6]$acetone, $[D_6]$DMSO or $CD_3CN$ on a Bruker Avance 400 MHz or 600 MHz instruments. Chemical shifts (δ) are reported in ppm and coupling constants (*J*) are given in Hertz and referenced to residual signals of solvents or internal standards: $CD_2Cl_2$ $\delta_H = 5.32$, $\delta_C = 53.80$; $[D_6]$acetone $\delta_H = 2.05$, $\delta_C = 29.84$; $[D_6]$DMSO $\delta_H = 2.50$, $\delta_C = 39.52$; $CD_3CN$ $\delta_H = 1.94$, $\delta_C = 1.32$; $Me_4Si$ $\delta_H = 0.00$; $CFCl_3$ $\delta_F = 0.0$; $BF_3 \cdot OEt_2$ (capillary) $\delta_F = -153.0$, $\delta_B = 0.0$. $^{13}C$ and $^{19}F$ NMR spectra were $^1H$ decoupled. Due to low intensity some aromatic quaternary carbons were not observed in the $^{13}C$ NMR. GC–MS spectra were recorded on an Agilent 7890A gas chromatograph coupled with a 5975C quadrupole mass-selective electron impact (EI) detector (70 eV). High-resolution mass spectra (HRMS) were recorded on an Agilent 7890A gas chromatograph coupled with a Waters GCT Premier orthogonal acceleration time-of-flight detector using chemical (CI) ionizations or LTQ Orbitrap XL instrument using electrospray (ESI) ionization. Elemental analyses were obtained using a Perkin–Elmer PE 2400 Series II CHNS. Infrared spectra were measured on a FTIR instrument. Purification of the products was performed by flash chromatography using silica gel 60. Dry solvents if used were obtained the following way: $Et_2O$ and THF were freshly distilled over

Na/benzophenone, MeCN was distilled over $P_2O_5$ and kept over activated 4 Å molecular sieves. Commercial 90% *t*-butyl nitrite was used.

**Synthesis of aryldiazonium tetrafluoroborates 3**

1. General procedure 1

To a stirred solution of **1** (18.3 mmol) in dry $Et_2O$ (30 mL) $BF_3{\cdot}OEt_2$ (5.46 g, 38.4 mmol, 2.1 equiv) was added and the resulting solution was cooled to −20 °C. *t*-BuONO (2.03 g, 20.0 mmol, 1.1 equiv) in dry $Et_2O$ (6 mL) was added over 5 min and the reaction mixture was warmed to rt over 30 min. EtOH (10 mL) was added, the precipitate was filtered and washed with EtOH (2 × 5 mL) and $Et_2O$ (2 × 5 mL). If the solid on the filter changed the color from white to yellow within minutes, washing was repeated. Traces of solvent were removed under reduced pressure providing pure **3**.

2. General procedure 2

To a stirred solution of **1** (22.8 mmol) in $CH_2Cl_2$ (65 mL) $BF_3{\cdot}OEt_2$ (6.81 g, 47.9 mmol, 2.1 equiv) was added. After stirring for 5 min, dry THF (10 mL) was added to give clear solution followed by cooling to −20 °C. *t*-Butyl nitrite (2.94 g, 22.8 mmol, 1.0 equiv) in $CH_2Cl_2$ (7 mL) was added and the reaction mixture was warmed to 0 °C over 30 min. The formed precipitate was filtered, washed with $Et_2O$ (25 mL) and dried under reduced pressure affording pure **3**.

3. General procedure 3

A mixture of aqueous HF (2.08 g, 104 mmol, 17.2 equiv), $H_3BO_3$ (2.29 g, 26.0 mmol, 4.3 equiv) and water (5 mL) was added to **1** (6.0 mmol). After 5 min of stirring at rt, $NaNO_2$ (765 mg, 11.1 mmol, 1.9 equiv) was added in few portions over 3 min. After 15 minutes, the formed precipitate was filtered, washed with $Et_2O$ (5 mL), suspended in MeCN (10 mL), filtered and dissolved in MeCN (4 mL). After addition of $Et_2O$, the formed precipitate was filtered and dried under reduced pressure affording pure **3**.

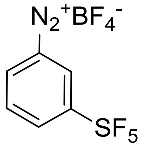


**3a**: Prepared according to the general procedure 1 starting from **1a** (4.00 g, 18.3 mmol), $BF_3{\cdot}OEt_2$ (5.46 g, 38.4 mmol, 2.1 equiv) and *t*-BuONO (2.03 g, 20.0 mmol, 1.1 equiv) affording **3a** as a white solid (5.51 g, 95% yield): FT-IR (film): $\nu_{max}$ (cm$^{-1}$) = 3097, 1589, 2299 ($N_2^+$), 1574, 1476, 1437, 1300, 1054 ($BF_4^-$), 1037 ($BF_4^-$), 864, 851 (SF); $^1$H NMR (400 MHz, $CD_3CN$): $\delta$ = 8.14 (t, $^3J_{H,H}$ = 8.5, 1H), 8.66 (ddd, $^3J_{H,H}$ = 8.6, $^4J$ = 2.0, $^4J$ = 1.0, 1H), 8.74 (dd, $^3J_{H,H}$ = 8.6, $^4J_{H,H}$ = 1.5, 1H), 9.03 (t, $^4J_{H,H}$ = 2.0, 1H); $^1$H NMR (400 MHz, [D$_6$]acetone): $\delta$ = 8.37 (t, $^3J_{H,H}$ = 8.5, 1H), 8.87 (ddd, $^3J_{H,H}$ = 8.5, $^4J_{H,H}$ = 2.3, 0.9, 1H), 9.11 (dd, $^3J_{H,H}$ = 8.3, $^4J_{H,H}$ = 1.3, 1H), 9.43 (t, $^4J_{H,H}$ = 2.0, 1H); $^{11}$B NMR (128 MHz, $CD_3CN$): $\delta$ = –0.6 (s); $^{13}$C NMR (101 MHz, $CD_3CN$): $\delta$ = 117.7, 131.8 (quint, $^3J_{C,F}$ = 5.3), 134.3, 136.9, 139.8 (quint, $^3J_{C,F}$ = 4.6), 153.7–154.6 (m); $^{19}$F NMR (377 MHz, [D$_6$]acetone): $\delta$ = -149.3 (s), 63.3 (d, $^2J_{F,F}$ = 150.3, 4F), 78.7–80.5 (m, 1F); Elem. anal.: Calc. for $C_6H_4BF_9N_2S$: C, 22.66; H, 1.27; F, 57.77; N, 8.81; S, 10.08. Found: C, 22.58; H, 1.15; F, 57.60; N, 8.75; S, 10.43.

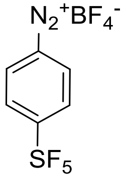


**3b**: Prepared according to the general procedure 1 starting from **1b** (6.00 g, 27.4 mmol), $BF_3{\cdot}OEt_2$ (8.1 g, 57.5 mmol, 2.1 equiv) and *t*-BuONO (3.10 g, 30.1mmol, 1.1 equiv) affording **3b** as a white solid (8.08 g, 93% yield). Also prepared according to the general procedure 2 starting from **1a** (5.00 g, 22.8 mmol), $BF_3{\cdot}OEt_2$ (6.81 g, 47.9 mmol, 2.1 equiv) and *t*-BuONO (2.90 g, 22.8 mmol, 1.0 equiv) affording **3b** as a white solid (6.22 g, 86% yield): FT-IR (film): $\nu_{max}$ (cm$^{-1}$) = 3119, 3070, 3031, 3014, 2986, 2304 ($N_2^+$), 1573, 1470, 1412, 1303, 1124, 1054 ($BF_4^-$), 842 (SF); $^1$H NMR (400 MHz, $CD_3CN$): $\delta$ = 8.34–8.38 (m, 2H),

8.71–8.74 (m, 2H); $^{1}$H NMR (400 MHz, [D$_6$]acetone): $\delta$ = 8.61–8.65 (m, 2H), 9.09–9.11 (m, 2H); $^{11}$B NMR (128 MHz, [D$_6$]acetone): $\delta$ = –0.3 (s); $^{13}$C NMR (101 MHz, CD$_3$CN): $\delta$ = 120.3, 130.7 (quint, $^{3}J_{C,F}$ = 4.8), 134.9, 161.8 (quint, $^{2}J_{C,F}$ = 20.5); $^{19}$F NMR (377 MHz, CD$_3$CN): $\delta$ = -149.5 (s), 62.2 (d, $^{2}J_{F,F}$ = 149.4, 4F), 78.3–79.9 (m, 1F); $^{19}$F NMR (377 MHz, [D$_6$]acetone): $\delta$ = –149.2 (s), 62.2 (d, $^{2}J_{F,F}$ = 149.7, 4F), 78.6–80.3 (m, 1F); HRMS (ESI$^+$): *m/z* = Calc. for $C_6H_4N_2F_5S$, 231.00099; Found, 231.00094; Elem. anal.: Calc. for $C_6H_4BF_9N_2S$: C, 22.66; H, 1.27; F, 57.77; N, 8.81; S, 10.08. Found: C, 22.58; H, 1.07; F, 57.48; N, 8.84; S, 10.63.

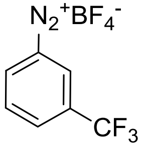


**3c**: Prepared according to general procedure 2 staring from **1c** (644 mg, 4.0 mmol), $BF_3{\cdot}OEt_2$ (1.20 g, 8.4 mmol, 2.1 equiv) and *t*-BuONO (453 mg, 4.4 mmol, 1.1 equiv) affording **3c** as a white solid (940 mg, 90% yield): $^{1}$H NMR (400 MHz, CD$_3$CN): $\delta$ = 8.15 (t, $^{3}J_{H,H}$ = 8.2, 1H), 8.53 (d, $^{3}J_{H,H}$ = 8.3, 1H), 8.76 (d, $^{3}J_{H,H}$ = 8.6, 1H), 8.85 (s, 1H); $^{13}$C NMR (101 MHz, CD$_3$CN): $\delta$ = 117.7, 123.0 (q, $^{1}J_{C,F}$ = 272.9), 130.9 (q, $^{3}J_{C,F}$ = 4.2), 133.8 (q, $^{2}J_{C,F}$ = 36.0), 134.2, 137.1, 139.4 (q, $^{3}J_{C,F}$ = 3.4); $^{19}$F NMR (376 MHz, CD$_3$CN): $\delta$ = -149.7 (s), -62.8 (s). Elem. anal.: Calc. for $C_7H_4BF_7N_2$: C, 32.35; H, 1.55; N, 10.78. Found: C, 32.42; H, 1.50; N, 10.92.

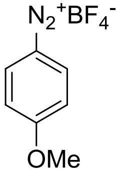


**3d**:[1] Prepared according to general procedure 3 staring from **1d** (738 mg, 6.0 mmol), HBF$_4$ (3.17 g, 36.0 mmol, 6.0 equiv), NaNO$_2$ (765 mg, 11.1 mmol, 1.9 equiv) and water (5 mL) affording **3d** as a pale violet solid (988 mg, 74% yield): $^{1}$H NMR (400 MHz, CD$_3$CN): $\delta$ = 4.05 (s, 3H), 7.32–7.36 (m, 2H), 8.40–8.44 (m, 2H); $^{13}$C NMR (101 MHz, CD$_3$CN): $\delta$ = 58.5, 102.8, 118.9, 136.8, 171.3; $^{19}$F NMR (376 MHz, [D$_6$]acetone): $\delta$ = –150.1 (s).

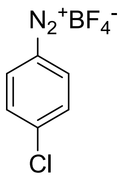


**3e**:[2] Prepared according to general procedure 3 staring from **1e** (762 mg, 6.0 mmol), HBF$_4$ (3.17 g, 36.0 mmol, 6.0 equiv), NaNO$_2$ (765 mg, 11.1 mmol, 1.9 equiv) and water (3 mL) affording **3e** as a white solid (1.06 g, 78% yield): $^{1}$H NMR (400 MHz, CD$_3$CN): $\delta$ = 7.91–7.95 (m, 2H), 8.45–8.50 (m, 2H); $^{13}$C NMR (101 MHz, CD$_3$CN): $\delta$ = 133.3, 134.9, 150.1; $^{19}$F NMR (376 MHz, CD$_3$CN): $\delta$ = -150.0 (s).

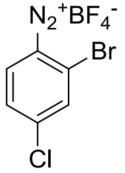


**3f**: Prepared according to general procedure 2 staring from **1f** (824 mg, 4.0 mmol), $BF_3{\cdot}OEt_2$ (1.20 g, 8.4 mmol, 2.1 equiv) and *t*-BuONO (453 mg, 4.4 mmol, 1.1 equiv) affording **3f** as a white solid (1.14 g, 93% yield): $^{1}$H NMR (400 MHz, CD$_3$CN): $\delta$ = 7.92 (dd, $^{3}J_{H,H}$ = 9.1, $^{4}J_{H,H}$ = 2.0, 1H), 8.25 (d, $^{4}J_{H,H}$ = 2.0, 1H), 8.55 (d, $^{3}J_{H,H}$ = 9.1, 1H); $^{13}$C NMR (101 MHz, CD$_3$CN): $\delta$ = 127.2, 132.6, 136.8, 137.2, 151.0; $^{19}$F NMR (376 MHz, CD$_3$CN): δ = -150.1 (s). Elem. anal.: Calc. for $C_6H_3BBrClF_4N_2$: C, 23.61; H, 0.99; N, 9.18. Found: C, 23.90; H, 1.02; N, 9.32.

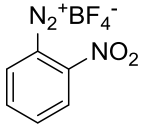


**3g**:[1] Prepared according to general procedure 2 staring from **1g** (552 mg, 4.0 mmol), $BF_3{\cdot}OEt_2$ (1.20 g, 2.1 equiv, 8.4 mmol) and *t*-BuONO (453 mg, 4.4 mmol, 1.1 equiv) affording **3g** as a white solid (898 mg, 95% yield): $^{1}$H NMR (400 MHz, CD$_3$CN): $\delta$ = 8.30–8.35 (m, 1H), 8.49–8.53 (m, 1H), 8.70 (dd, $^{3}J_{H,H}$ = 8.5, $^{4}J_{H,H}$ = 1.1, 1H), 8.88 (dd, $^{3}J_{H,H}$ = 8.2, $^{3}J_{H,H}$ = 1.4, 1H); $^{13}$C NMR (101 MHz, CD$_3$CN): $\delta$ = 129.7, 137.9, 138.0, 144.4; $^{19}$F NMR (376 MHz, CD$_3$CN): $\delta$ = -150.2 (s).

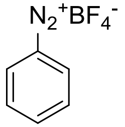


**3h**: Prepared according to general procedure 3 staring from aniline (**1h**) (10.0 g, 108 mmol), HBF$_4$ (57 g, 648 mmol, 6.0 equiv), NaNO$_2$ (11.2 g, 162 mmol, 1.5 equiv) and water (20 mL) affording 3 h as a white solid turning pink (4.91 g,

23% yield): $^{1}$H NMR (400 MHz, $CD_3CN$): $\delta$ = 7.90–7.94 (m, 2H), 8.25 (tt, $^{3}J_{H,H}$ = 7.7, $^{4}J_{H,H}$ = 1.2, 1H), 8.49–8.51 (m, 2H); $^{13}$C NMR (126 MHz, $CD_3CN$): $\delta$ = 115.7, 132.8, 133.4, 142.9; $^{19}$F NMR (470 MHz, $CD_3CN$): $\delta$ = -150.3 (s); Elem. anal.: Calc. for $C_6H_5BF_4N_2$: C, 37.55; H, 2.63; B, 5.63; F, 39.60; N, 14.60. Found: C, 37.69; H, 2.71; N, 14.69.

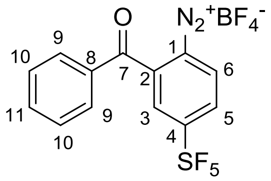


**3i**: Prepared according to general procedure 2 staring from **1i** (969 mg, 3.0 mmol), $BF_3{\cdot}OEt_2$ (894 mg, 6.3 mmol, 2.1 equiv) and *t*-BuONO (309 mg, 3.3 mmol, 1.1 equiv) affording **3i** as a white solid (859 mg, 97% yield): FT-IR (film): $\nu_{max}$ (cm$^{-1}$) = 3069, 3024, 2300 ($N_2^+$), 1673 (CO), 1597, 1582, 1450, 1395, 1317, 1308, 1290, 1258, 1058 ($BF_4^-$), 1036 ($BF_4^-$), 952, 845 (SF); $^{1}$H NMR (500 MHz, $CD_3CN$): $\delta$ = 7.65–7.69 (m, 2H, C10H), 7.82–7.86 (m, 1H, C11H), 7.91–7.93 (m, 2H, C9H), 8.48 (d, $^{4}J_{H,H}$ = 2.2, 1H, C3H), 8.62 (dd, $^{3}J_{H,H}$ = 9.0, $^{4}J_{H,H}$ = 2.3, 1H, C5H), 9.02 (d, $^{3}J_{H,H}$ = 8.9, 1H, C6H); $^{11}$B NMR (160 MHz, $CD_3CN$): $\delta$ = -0.6 (br s); $^{13}$C NMR (126 MHz, $CD_3CN$): $\delta$ = 119.7 (C1), 130.3 (C10), 131.8 (C9), 132.8 (quint, $^{3}J_{C,F}$ = 4.7, C3), 133.6 (quint, $^{3}J_{C,F}$ = 4.7, C5), 134.6 (C8), 136.4 (C11), 138.1 (C6), 140.8 (C2), 160.5 (quint, $^{2}J_{C,F}$ = 21.4, C4), 189.7 (C7); $^{19}$F NMR (377 MHz, $CD_3CN$): $\delta$ = -150.7 (s), 62.6 (d, $^{2}J_{F,F}$ = 149.7, 4F), 77.3–79.0 (m, 1F); HRMS (ESI$^+$): *m/z* = Calc. for $C_{13}H_8F_5N_2OS$ $[M]^+$, 335.02720; Found, 335.02733.

## Synthesis of arylboronates 2

1. From anilines **1**[3]

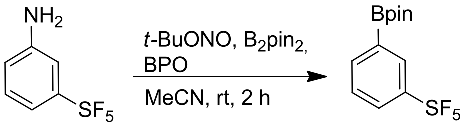


**1a** **2a**

A mixture of **1a** (219 mg, 1.0 mmol), $B_2pin_2$ (279 mg, 1.1 mmol, 1.1 equiv), *t*-BuONO (155 mg, 1.5 mmol, 1.5 equiv), dibenzoyl peroxide (BPO) (2 mg, 0.02 mmol, 2 mol%) in MeCN (3 mL) was stirred at rt for 2 h. The solvent was removed under reduced pressure. Purification by column chromatography ($CH_2Cl_2$/$Et_2O$/hexane, 5:5:90) afforded **2a** as a brown solid (240 mg, 70% yield).

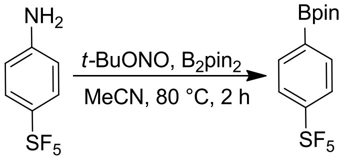


**1b** **2b**

A mixture of **1b** (219 mg, 1.0 mmol), $B_2pin_2$ (279 mg, 1.1 mmol, 1.1 equiv), *t*-BuONO (155 mg, 1.5 mmol, 1.5 equiv) and MeCN (3 mL) was stirred and heated to 80 °C for 2 h. The solvent was removed under reduced pressure. Purification by column chromatography ($CH_2Cl_2$/$Et_2O$/hexane, 5:5:90) afforded **2b** as a brown solid (180 mg, 55% yield, 82% purity according to $^{1}$H NMR).

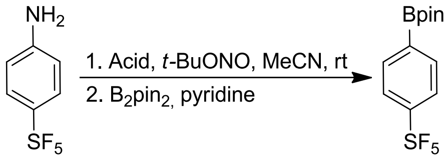


**1b** **2b**

To a stirred mixture of **1b** (219 mg, 1.0 mmol) and *p*TsOH·$H_2O$ (190 mg, 1.0 mmol, 1 equiv) in MeCN (3 mL), *t*-BuONO (103 mg, 1.0 mmol, 1.0 equiv) in MeCN (1 mL) was added over 1 min. After 10 min of stirring at rt $B_2pin_2$ (254 mg, 1.0 mmol, 1.0 equiv) was added at once followed by addition of pyridine (125 mg, 1.6 mmol, 1.6 equiv) over 45 min. The mixture was stirred overnight followed by removal of solvent under reduced pressure and column chromatography ($CH_2Cl_2$/$Et_2O$/hexane, 5:5:90) to afford **2b** as a pale yellow solid (167 mg, 51%).

To a stirred solution of **1b** (219 mg, 1.0 mmol) in MeCN (1.0 mL) at 0 °C, 33% $HBF_4$ (450 mg, 1.7 equiv) in MeCN (1.0 mL) was added. The mixture was stirred for 5 min followed by addition of *t*-BuONO (107 mg, 1.05 mmol, 1.05 equiv) in MeCN (1.0 mL). After 15 min stirring $B_2pin_2$ (254 mg, 1.0 mmol, 1.0 equiv) in MeCN (1.0 mL) was

added and the reaction mixture was transferred to a −25 °C bath. Pyridine (330 µL, 4.0 mmol, 4.0 equiv) in MeCN (1.0 mL) was added over ca. 30 s and the mixture was allowed to warm to rt over 1 h. Solvent was removed under reduced pressure. Solution of 1M HCl (10 mL) was added to the crude product mixture, extraction with $Et_2O$ (2 × 10 mL), washing the combined organic phase with water (10 mL), brine (10 mL) and drying ($MgSO_4$), followed by column chromatography ($CH_2Cl_2$/$Et_2O$/hexane, 5:5:90) afforded **2b** as a pale yellow solid (266 mg, 81%). The same procedure using 35% HCl (177 mg, 1.7 equiv) provided **2b** as a pale yellow solid (257 mg, 78%).

2. From aryldiazonium tetrafluoroborates **3** using pyridine, General procedure

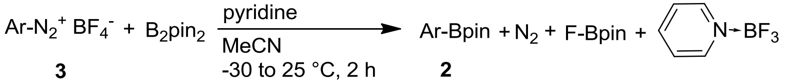


**3** (1 mmol), $B_2pin_2$ (254 mg, 1 mmol, 1 equiv) and MeCN (2 mL) were mixed and cooled to −30 °C. Pyridine (0.33 mL, 4 mmol, 4 equiv) was added to the stirred solution over half a minute and the mixture was slowly warmed to rt over 2 h. Solvent was removed under reduced pressure and the residue was dissolved in a minimum amount of MeCN. Pyridine trifluoroborate complex precipitated upon addition of $Et_2O$ and was filtered, washed with $Et_2O$ and dried under reduced pressure. The filtrate was concentrated and purified by column chromatography ($Et_2O$/$CH_2Cl_2$/hexane, 5:5:90) affording the desired arylboronate **2**. Side products, pyridine-$BF_3$ complex and 2-fluoro-4,4,5,5-tetramethyl-1,3,2-dioxaborolane (F-Bpin), were observed by $^{11}$B and $^{19}$F NMR of the crude reaction mixture and compared with authentic samples.

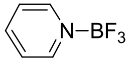


Prepared independently by mixing $BF_3{\cdot}OEt_2$ (30 mg, 0.21 mmol), pyridine (17 mg, 0.21 mmol, 1.0 equiv) and $Et_2O$ (0.5 mL), solvent removal under reduced pressure and addition of $CD_3CN$: $^{11}$B NMR (128 MHz, $CD_3CN$): $\delta$ = 1.0 (q, $^2J_{BF}$ = 10.4); $^{19}$F NMR (376 MHz, $CD_3CN$): $\delta$ = -150.1 to -149.9 (m).

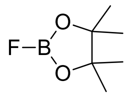


Prepared independently by a modified literature procedure[4] by slow addition of *n*-BuLi (2.5 M, 0.80 mL, 2.0 mmol, 2.0 equiv) to a solution of pinacol (118 mg, 1.0 mmol) in dry THF (3 mL) causing precipitation of the bislithium salt of pinacol. $BF_3{\cdot}OEt_2$ (156 mg, 1.1 mmol, 1.1 equiv) in dry THF (1 mL) was added dropwise and the mixture was stirred for 15 min. Solvent was removed under reduced pressure, and $CD_3CN$ was added: $^{11}$B NMR (128 MHz, $CDCl_3$): $\delta$ = 23.0 (br s) (4.2[5], ca. 1.2[6]); $^{19}$F NMR (376 MHz, $CD_3CN$): $\delta$ = –151.5 (s) (–152.5[6]).

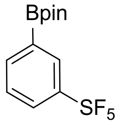


**2a**: Prepared according to the general procedure from **3a** (318 mg, 1.0 mmol), $B_2pin_2$ (254 mg, 1.0 mmol, 1 equiv), MeCN (2 mL) and pyridine (0.33 mL, 4.0 mmol, 4 equiv) affording **2a** as a white solid (265 mg, 80% yield): $R_f$ = 0.55 ($Et_2O$/$CH_2Cl_2$/hexane, 5:5:90); mp = 72–74 °C (MeOH); FT-IR (film): $\nu_{max}$ (cm$^{-1}$) = 3007, 2988, 2973, 2934, 1603, 1484, 1460, 1424, 1437, 1374, 1356, 1350, 1335, 1272, 1214, 1169, 1146 (BO), 1101, 1082, 916, 845 (SF), 838, 599; $^1$H NMR (400 MHz, $CDCl_3$): $\delta$ = 1.35 (s, 12H), 7.46 (t, $^3J_{H,H}$ = 7.8, 1H), 7.82 (ddd, $^3J_{H,H}$ = 8.3, $^4J_{H,H}$ = 2.4, 1.1, 1H), 7.90–7.95 (m, 1H), 8.17 (d, $^4J_{H,H}$ = 2.2, 1H); $^{11}$B NMR (128 MHz, $CDCl_3$): $\delta$ = 30.1 (br s); $^{13}$C NMR (101 MHz, $CDCl_3$): $\delta$ = 24.8, 84.5, 128.2, 128.4 (quint, $^3J_{C,F}$ = 4.7), 131.7 (quint, $^3J_{C,F}$ = 4.5), 137.8, 153.7 (quint, $^2J_{C,F}$ = 16.6); $^{19}$F NMR (377 MHz, $CDCl_3$): $\delta$ = 62.2 (d, $^2J_{F,F}$ = 149.7, 4F), 83.3–85.0 (m, 1F); MS (EI$^+$): *m/z* (%) = 315 (82) [M - Me]$^+$, 314 (20), 231 (89), 230 (22), 180 (15), 179 (14), 131 (47), 104 (18), 103 (23), 85 (27), 77 (14), 59 (32), 58 (44), 57 (23), 43 (100), 42 (48),

41 (43), 39 (16); HRMS (Cl$^+$): $m/z$ = Calc. for $C_{12}H_{17}BF_5O_2S$ [M + H]$^+$, 331.0962; Found, 331.0974.

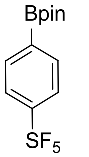

**2b**: Prepared according to the general procedure from **3b** (954 mg, 3.0 mmol), $B_2pin_2$ (762 mg, 3.0 mmol, 1.0 equiv), MeCN (6 mL) and pyridine (1.0 mL, 12.0 mmol, 4.0 equiv) affording **2b** as a white solid (760 mg, 77% yield): $R_f$ = 0.44 ($Et_2O/CH_2Cl_2$/hexane, 5:5:90); mp = 85–89 °C (MeOH); FT-IR (film): $\nu_{max}$ (cm$^{-1}$) = 3001, 2977, 2925, 1602, 1400, 1374, 1366, 1336, 1275, 1213, 1169, 1145 (BO), 1083, 1019, 839 (SF), 595; $^1$H NMR (400 MHz, $CDCl_3$): $\delta$ = 1.35 (s, 12H), 7.72–7.74 (m, 2H), 7.87–7.90 (m, 2H); $^{11}$B NMR (128 MHz, $CDCl_3$): $\delta$ = 30.0 (br s); $^{13}$C NMR (101 MHz, $CDCl_3$) $\delta$ = 24.8, 84.4, 125.0 (quint, $^3J_{C,F}$ = 4.6), 135.1, 156.1 (quint, $^2J_{C,F}$ = 17.6); $^{19}$F NMR (377 MHz, $CDCl_3$): $\delta$ = 61.7 (d, $^2J_{F,F}$ = 149.7, 4F), 82.8–84.5 (m, 1F); MS (EI$^+$): $m/z$ (%) = 316 (14), 315 (100) [M - Me]$^+$, 314 (24), 245 (11), 244 (88), 231 (62), 230 (15), 104 (11), 103 (10), 85 (18), 59 (20), 58 (28), 57 (15), 43 (39), 42 (31), 41 (28), 39 (10); HRMS (Cl$^+$): $m/z$ = Calc. for $C_{12}H_{17}BF_5O_2S$ [M + H]$^+$, 331.0962; Found, 331.0959.

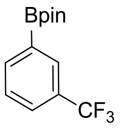

**2c**:[7] Prepared according to the general procedure from **3c** (260 mg, 1.0 mmol), $B_2pin_2$ (254 mg, 1.0 mmol, 1.0 equiv), MeCN (2 mL) and pyridine (0.33 mL, 4.0 mmol, 4.0 equiv) affording **2c** as a yellow liquid which slowly solidified (215 mg, 64% yield); $R_f$ = 0.45 ($Et_2O/CH_2Cl_2$/hexane, 5:5:90); $^1$H NMR (400 MHz, $CDCl_3$): $\delta$ = 1.35 (s, 12H), 7.48 (t, $^3J_{H,H}$ = 7.6, 1H), 7.66–7.73 (m, 1H), 7.98 (d, $^3J_{H,H}$ = 7.3, 1H), 8.07 (s, 1H); $^{13}$C NMR (101 MHz, $CDCl_3$): $\delta$ = 24.9, 84.3, 124.3 (q, $^1J_{C,F}$ = 272.2), 127.8 (q, $^3J_{C,F}$ = 3.7), 128.0, 130.0 (q, $^2J_{F,C}$ = 32.0), 131.3 (q, $^3J_{F,C}$ = 3.7), 138.0 (q, $^5J_{F,C}$ = 1.5 Hz); $^{19}$F NMR (376 MHz, $CDCl_3$): $\delta$ = -63.1 (s).

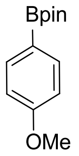

**2d**:[5] Prepared according to the general procedure from **3d** (222 mg, 1.0 mmol), $B_2pin_2$ (254 mg, 1.0 mmol, 1.0 equiv), MeCN (2 mL) and pyridine (0.33 mL, 4.0 mmol, 4.0 equiv) affording **2d** as a yellow liquid (148 mg, 64% yield); $R_f$ = 0.45 ($Et_2O/CH_2Cl_2$/hexane, 5:5:90); $^1$H NMR (400 MHz, $CDCl_3$): $\delta$ = 1.33 (s, 12H), 3.82 (s, 3H), 6.87–6.92 (m, 2H), 7.73–7.78 (m, 2H); $^{13}$C NMR (101 MHz, $CDCl_3$): $\delta$ = 24.8, 55.1, 83.5, 113.3, 136.5, 162.1.

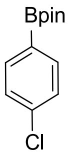

**2e**:[6] Prepared according to the general procedure from **3e** (226 mg, 1.0 mmol), $B_2pin_2$ (254 mg, 1.0 mmol, 1.0 equiv), MeCN (2 mL) and pyridine (0.33 mL, 4.0 mmol, 4.0 equiv) affording **2e** as a yellow liquid which slowly solidifies (129 mg, 54% yield); $R_f$ = 0.69 ($Et_2O/CH_2Cl_2$/hexane, 5:5:90); $^1$H NMR (400 MHz, $CDCl_3$) $\delta$ = 1.34 (s, 12H), 7.32–7.35 (m, 2H), 7.71–7.74 (m, 2H); $^{13}$C NMR (101 MHz, $CDCl_3$) $\delta$ = 24.8, 84.0, 128.0, 136.1, 137.5.

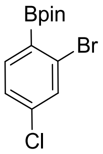

**2f**:[8] Prepared according to the general procedure from **3f** (305 mg, 1.0 mmol), $B_2pin_2$ (254 mg, 1.0 mmol, 1.0 equiv), MeCN (2 mL) and pyridine (0.33 mL, 4.0 mmol, 4.0 equiv) affording **2f** as a yellow liquid (92 mg, 30% yield); $R_f$ = 0.65 ($Et_2O/CH_2Cl_2$/hexane, 5:5:90); FT-IR (film): $\nu_{max}$ (cm$^{-1}$) 3086, 3056, 1580, 1543, 1472, 1380, 1372, 1344, 1320 (B-O), 1273, 1253, 1214, 1167, 1146 (B-O), 1104, 1029, 962, 858; $^1$H NMR (400 MHz, $CDCl_3$): $\delta$ = 1.37 (s, 12H), 7.26 (dd, $^3J_{H,H}$ = 8.1, $^4J_{H,H}$ = 1.8, 1H), 7.56 (d, $^3J_{H,H}$ = 8.1, 1H), 7.57 (d, $^4J_{H,H}$ = 2.0, 1H); $^{13}$C NMR (101 MHz, $CDCl_3$): $\delta$ = 24.8, 84.4, 126.7, 128.4, 132.5, 137.2, 137.4; MS (EI$^+$): $m/z$ (%) = 318 (19) [M]$^+$, 303 (24) [M - Me]$^+$, 239 (34), 238 (21), 237 (100), 236 (25), 221 (23), 219 (89), 218 (47), 217 (73), 216 (33), 197 (25), 195 (74), 137 (20), 85 (29), 43 (41), 41 (36); HRMS (Cl$^+$): $m/z$ = Calc. for $C_{12}H_{17}BBrClO_2$ [M + H]$^+$, 317.0115; Found, 317.0116.

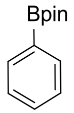


**2h**:[9] Prepared according to the general procedure from **3h** (192 mg, 1.0 mmol), $B_2pin_2$ (254 mg, 1.0 mmol, 1.0 equiv), MeCN (2 mL) and pyridine (0.33 mL, 4.0 mmol, 4.0 equiv) affording **2h** as a pale yellow liquid which solidifies (110 mg, 54%); $R_f$ = 0.43 ($Et_2O/CH_2Cl_2$/hexane, 5:5:90); $^1H$ NMR (400 MHz, $CDCl_3$): $\delta$ = 1.34 (s, 12H), 7.34–7.38 (m, 2H), 7.43–7.47 (m, 1H), 7.80–7.82 (m, 2H); $^{13}C$ NMR (101 MHz, $CDCl_3$): $\delta$ = 24.8, 83.7, 127.7, 131.2, 134.7.

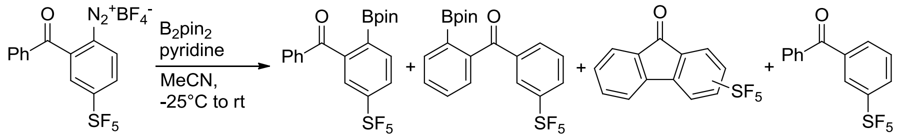


**3i** **2i** **2i'** **5i** **6i**

Borylation of **3i**: **3i** (380 mg, 0.9 mmol) was subjected to borylation according to the general procedure with $B_2pin_2$ (229 mg, 0.9 mmol, 1.0 equiv), MeCN (2 mL) and pyridine (0.30 mL, 3.64 mmol, 4.0 equiv) affording a mixture of unreacted $B_2pin_2$ and products **2i**, **2i'**, **5i** and **6i** in 16:31:28:25 ratio (GCMS, Figures SI1-SI7). $B_2pin_2$: MS (EI$^+$): *m/z* (%) = 239 (38) [M - Me]$^+$, 238 (18), 113 (10), 85 (25), 84 (100) $[C_6H_{12}]^+$, 83 (44), 69 (26), 59 (11), 57 (10), 55 (14), 43 (23), 42 (11), 41 (30), 39 (10); **2i**: MS (EI$^+$): *m/z* (%) = 419 (13) [M - Me]$^+$, 377 (19), 376 (100), 375 (28), 336 (13), 335 (79), 334 (18), 105 (7) [PhCO]$^+$, 77 (9) [Ph]$^+$; **2i'**: MS (EI$^+$): *m/z* (%) = 419 (10) [M - Me]$^+$, 377 (20), 376 (100), 375 (28), 336 (11), 335 (70), 334 (18), 207 (12), 164 (8); **5i** isomer 1: MS (EI$^+$): *m/z* (%) = 307 (15) [M + 1]$^+$, 306 (100) [M]$^+$, 198 (36), 179 (28) [M - $SF_5$]$^+$, 170 (25), 169 (9), 151 (56), 150 (35); **5i** isomer 2: MS (EI$^+$): *m/z* (%) = 307 (16) [M + 1]$^+$, 306 (100) [M]$^+$, 199 (14), 198 (96), 179 (10) [M - $SF_5$]$^+$, 170 (35), 169 (8), 151 (40), 150 (39), 85 (10), 75 (10); **6i**: MS (EI$^+$): *m/z* (%) = 308 (53) [M]$^+$, 231 (28) [M - Ph]$^+$, 181 (10) [M - $SF_5$]$^+$, 105 (100) [PhCO]$^+$, 95 (10), 77 (41) [Ph]$^+$, 76 (13), 75 (12), 51 (12).

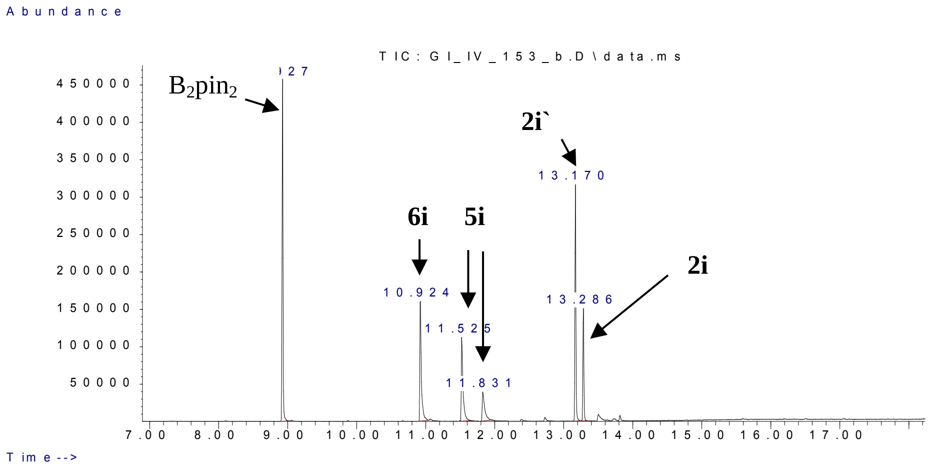


**Figure S1:** GC chromatogram of the crude product mixture of borylation of **3i**.

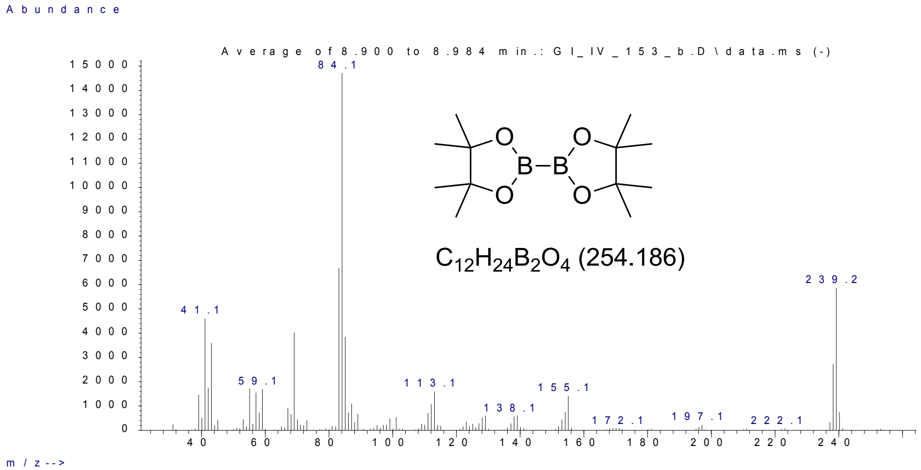


**Figure S2:** MS (EI$^+$) spectrum of $B_2pin_2$.

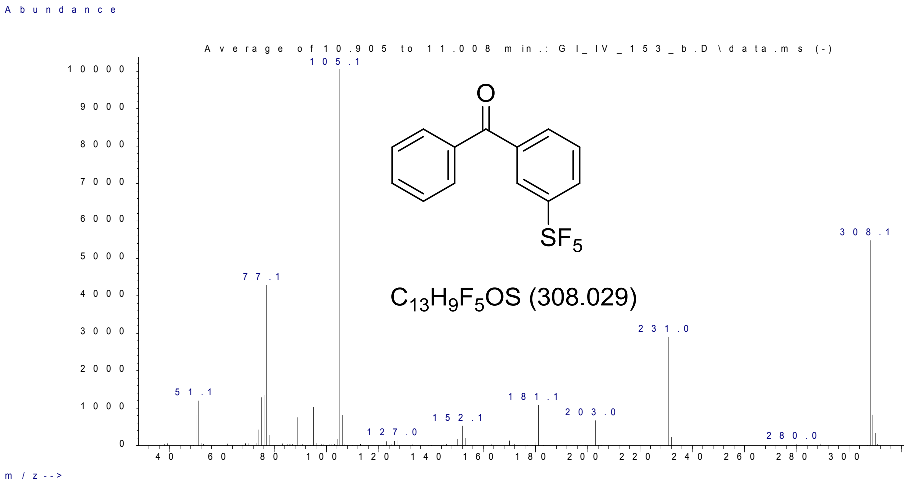


**Figure S3:** MS ($EI^+$) spectrum of **6i**.

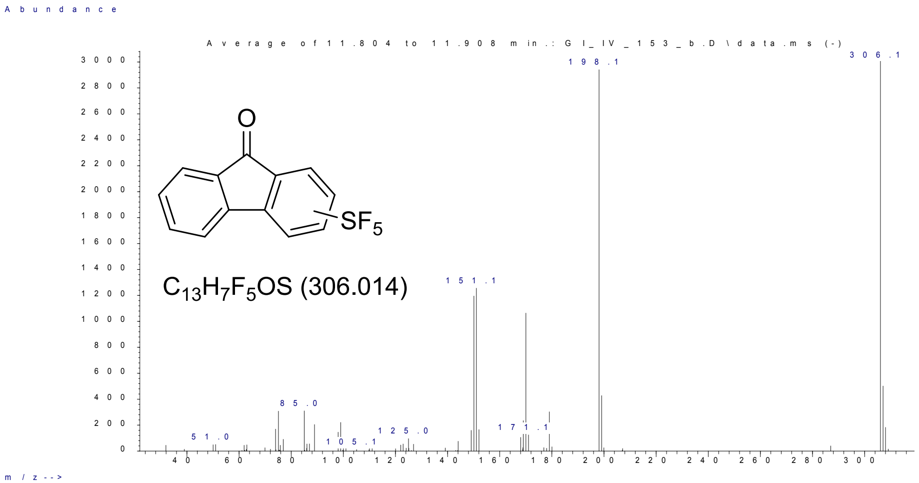


**Figure S5:** MS ($EI^+$) spectrum of **5a** isomer 2.

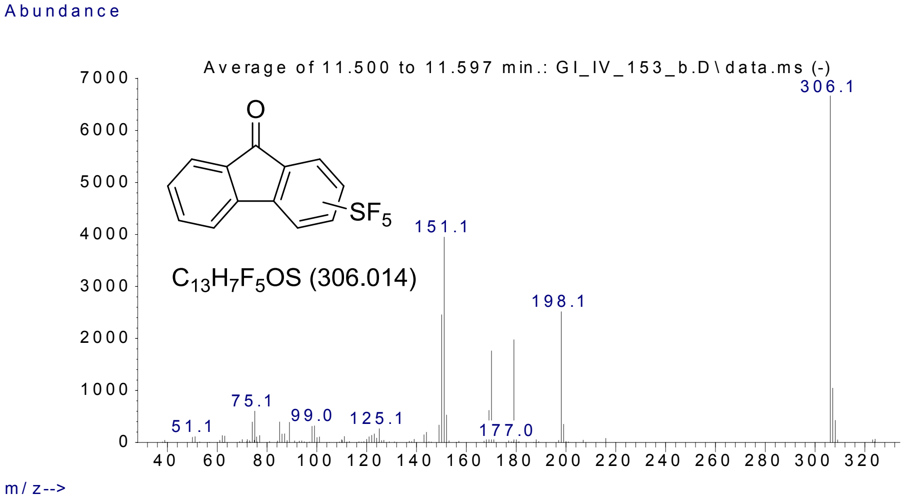


**Figure S4:** MS ($EI^+$) spectrum of **5a** isomer 1.

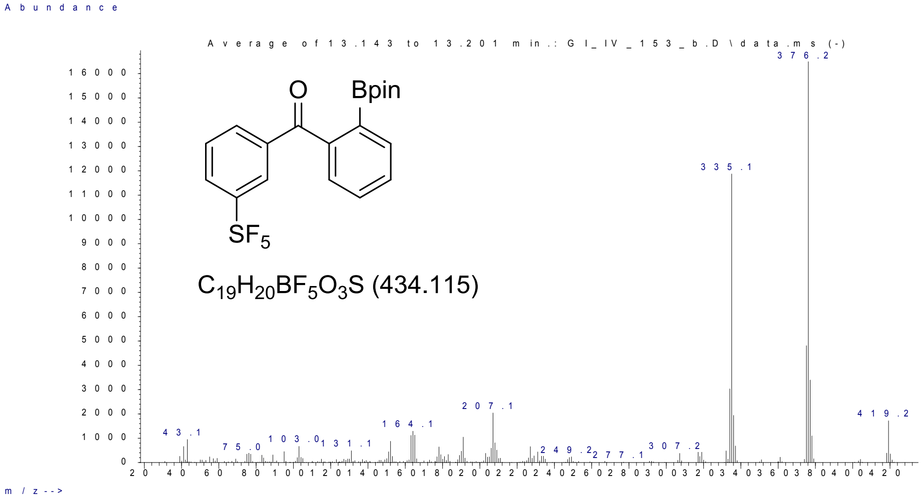


**Figure S6:** MS ($EI^+$) spectrum of **2i’**.

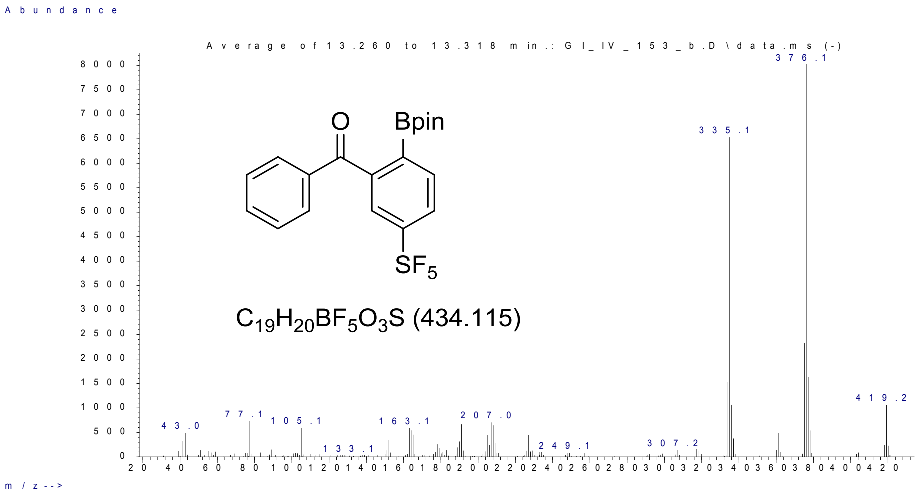


**Figure S7:** MS ($EI^+$) spectrum of **2i**.

**Synthesis of biaryls 7 by the Suzuki coupling reaction, General procedure**

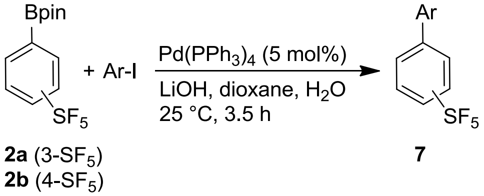


To a mixture of **2a** or **2b** (165 mg, 0.5 mmol), $Pd(Ph_3P)_4$ (29 mg, 0.025 mmol, 5 mol %), ArI (0.55 mmol, 1.1 equiv) in dry 1,4-dioxane (2 mL), a solution of $LiOH{\cdot}H_2O$ (84 mg, 2 mmol, 4 equiv) in water (1 mL) was added. The reaction mixture was stirred under argon at 50 °C for 3.5 h followed by addition of water (10 mL). Extraction into $Et_2O$ (3 × 10 mL), drying ($MgSO_4$) and solvent removal under reduced pressure provided crude product which was purified by column chromatography.

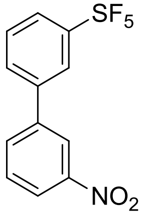


**7ah**: Prepared according to the general procedure from **2a** (165 mg, 0.5 mmol) and 3-nitroiodobenzene (137 mg, 0.55 mmol, 1.1 equiv) affording **7ah** as a white solid (89 mg, 55% yield): $R_f$ = 0.13 ($CH_2Cl_2$/hexane, 10:90); mp = 72–75 °C; FT-IR (film): $\nu_{max}$ ($cm^{-1}$) = 3092, 1600, 1532 (N=O), 1495, 1472, 1349 (N=O), 1117, 839 (SF), 738 (N=O), 597; $^1H$ NMR (400 MHz, $CDCl_3$): $\delta$ = 7.60–7.70 (m, 2H), 7.77–7.79 (m, 1H), 7.82–7.85 (m, 1H), 7.90–7.93 (m, 1H), 7.98–7.99 (m, 1H), 8.27–8.30 (m, 1H), 8.44–8.45 (m, 1H); $^{13}C$ NMR (101 MHz, $CDCl_3$): $\delta$= 122.1, 123.0, 124.8 (quint, $^3J_{C,F}$ = 4.6), 125.9 (quint, $^3J_{C,F}$ = 4.8), 129.6, 130.2, 130.3, 133.2, 139.8, 141.1, 148.8, 154.7 (quint, $^2J_{C,F}$ = 17.6); $^{19}F$ NMR (377 MHz, $CDCl_3$): $\delta$= 62.3 (d, $^2J_{F,F}$ = 149.7, 4F), 82.4–84.1 (m, 1F); MS ($EI^+$): *m/z* (%) = 326 (10), 325 (72) $[M]^+$, 295 (10), 170 (28), 153 (13), 152 (100), 151 (26), 150 (14); HRMS ($CI^+$): *m/z* = Calc. for $C_{12}H_9F_5NO_2S$ $[M + H]^+$, 326.0274; Found, 326.0278.

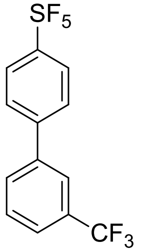


**7bc**: Prepared according to the general procedure from **2b** (165 mg, 0.50 mmol) and 3-(trifluoromethyl)iodobenzene (150 mg, 0.55 mmol, 1.1 equiv) affording **7bc** as a white solid (92 mg, 53% yield): $R_f$ = 0.39 (hexane); mp = 66–68 °C; FT-IR (film): $\nu_{max}$ ($cm^{-1}$) = 1602, 1486, 1446, 1439, 1399, 1337, 1262, 1170, 1131, 1102, 1077, 1038, 847 (SF), 837 (SF), 649, 598; $^1H$ NMR (400 MHz, $CDCl_3$): $\delta$= 7.58–7.69 (m, 4H), 7.75–7.76 (m, 1H), 7.82 (br s, 1H), 7.84–7.87 (m, 2H); $^{13}C$ NMR (101 MHz, $CDCl_3$): $\delta$= 125.3 (q, $^1J_{C,F}$ = 272.3), 124.1 (q, $^3J_{C,F}$ = 3.7), 125.1 (q, $^3J_{C,F}$ = 3.7), 126.7 (quint, $^3J_{C,F}$ = 4.6), 127.4, 129.6, 130.6, 131.6 (quint, $^2J_{C,F}$ = 32.0), 139.9, 143.0, 153.5 (quint, $^2J_{C,F}$ = 17.6); $^{19}F$ NMR (377 MHz, $CDCl_3$): $\delta$ = -63.2 (s, 3F), 62.5 (d, $^2J_{F,F}$ = 149.7, 4F), 82.8–84.5 (m, 1F); MS ($EI^+$): *m/z* (%) = 349 (15), 348 (100) $[M]^+$, 329 (17) , 241 (14), 240 (99), 221 (13), 219 (15),

201 (34), 171(11), 170 (24), 152 (27), 151 (13).; HRMS ($CI^+$): *m/z* = Calc. for $C_{13}H_9F_8S$ $[M + H]^+$, 349.0297; Found, 349.0298.

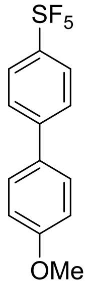


**7bd**: Prepared according to the general procedure from **2b** (165 mg, 0.5 mmol) and 4-(methoxy)iodobenzene (129 mg, 0.55 mmol, 1.1 equiv) affording **7bd** as a white solid (80 mg, 52% yield); $R_f$ = 0.17 (hexane); mp = 122–125 °C; FT-IR (film): $\nu_{max}$ ($cm^{-1}$)= 3067, 3030, 2979, 2949, 2850 ($CH_3$), 1610, 1582, 1528, 1493, 1461, 1445 ($OCH_3$), 1397, 1314, 1298, 1261, 1184, 1115, 1037, 846 (SF), 838, 820, 581; $^1$H NMR (400 MHz, $CDCl_3$): $\delta$ = 3.85 (s, 3H), 6.97–7.01 (m, 2H), 7.49–7.53 (m, 2H), 7.57–7.60 (m, 2H), 7.74–7.80 (m, 2H); $^{13}$C NMR (101 MHz, $CDCl_3$): $\delta$ = 55.3, 114.5, 126.3 (quint, $^3J_{C,F}$ = 4.6), 126.6, 128.4, 131.4, 144.1, 152.3 (quint, $^2J_{C,F}$ = 16.8), 160.1; $^{19}$F NMR (377 MHz, $CDCl_3$): $\delta$ = 62.8 (d, $^2J_{F,F}$ = 149.7, 4F), 83.7–85.5 (m, 1F); MS ($EI^+$): *m/z* (%) = 311 (15), 310 (100) $[M]^+$, 295 (9), 187 (24), 159 (26), 140 (10), 139 (26), 133 (12); HRMS ($Cl^+$): *m/z* = Calc. for $C_{13}H_{12}F_5OS$ $[M + H]^+$, 311.0529; Found, 311.0533.

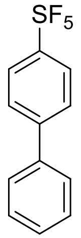


**7bh**:[10] Prepared according to the general procedure from **2b** (165 mg, 0.50 mmol) and iodobenzene (74 µL, 0.55 mmol, 1.1 equiv) affording **7bh** as a white solid (82 mg, 58% yield); $R_f$ = 0.56 (hexane); FT-IR (film): $\nu_{max}$ ($cm^{-1}$) = 3080, 3032, 1569, 1485, 1450, 1400, 841 (SF), 766, 721, 695, 586; $^1$H NMR (400 MHz, $CDCl_3$): $\delta$ = 7.38–7.49 (m, 3H), 7.56–7.58 (m, 2H), 7.63–7.65 (m, 2H), 7.80–7.82 (m, 2H); $^{13}$C NMR (101 MHz, $CDCl_3$): $\delta$ = 126.4 (quint, $^3J_{C,F}$ = 4.6), 127.2, 127.3, 128.4, 129.0, 139.1, 144.5, 152.9 (quint, $^2J_{C,F}$ = 17.6); $^{19}$F NMR (377 MHz, $CDCl_3$): $\delta$ = 62.7 (d, $^2J_{F,F}$ =150.3, 4F), 83.4–85.1 (m, 1F); HRMS ($Cl^+$): *m/z* = Calc. for $C_{12}H_{10}F_5S$ $[M + H]^+$, 281.0423; Found 281.0417.

**Synthesis of 4-(pentaflurosulfanyl)phenylboronic acid (8b)**

1. From boronic ester **2b**

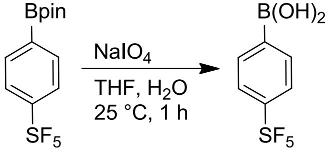


**2b** **8b**

**8b**: **2b** (330 mg, 1 mmol), THF (4 mL) and water (1 mL) were stirred at rt for 5 min. $NaIO_4$ (642 mg, 3.0 mmol, 3.0 equiv) was added in one portion. After stirring for additional 30 min, 1 M HCl (0.7 mL) was added and the mixture was left stirring overnight, followed by removal of solvent under reduced pressure, addition of water (15 mL), extraction with $Et_2O$ (3 × 10 mL), drying ($MgSO_4$), and solvent removal under reduced pressure. The resulting pale orange solid was sonicated for 10 min. with hexane (1 mL) and the hexane was carefully removed. Sonication with hexane was repeated twice and drying under reduced pressure at 30 °C provided a mixture of **8b** and 5% of its anhydride (trimer) as white solid (212 mg, 85% yield): mp > 250 °C (subl.); FT-IR (film): $\nu_{max}$ ($cm^{-1}$) = 1604, 1573, 1404, 1367, 1351, 1317, 1307, 1104, 1079, 1019, 842 (SF), 826, 598; $^1$H NMR (400 MHz, $CD_3CN$): $\delta$ = 6.25 (s, 2H), 7.81 (d, $^3J_{H,H}$ = 8.6, 2H), 7.92 (d, $^3J_{H,H}$ = 8.2, 2H); $^1$H NMR (400 MHz, [$D_6$]DMSO): $\delta$ = 7.89 (d, $^3J_{H,H}$ = 8.6, 2H), 8.05 (d, $^3J_{H,H}$ = 8.1, 2H); $^{11}$B NMR (128 MHz, [$D_6$]DMSO): $\delta$ = 28.7 (br s); $^{13}$C NMR (101 MHz, [$D_6$]DMSO): $\delta$ = 124.6–124.8 (m), 134.2, 142.8–143.4 (m), 153.7–154.4 (m); $^{19}$F NMR (377 MHz, $CD_3CN$): $\delta$ = 63.0 (d, $^2J_{F,F}$ = 147.0, 4F), 84.7–86.4 (m, 1F); HRMS ($ESI^-$): *m/z* = Calc. for $C_6H_5BF_5O_2S$ $[M - H]^-$, 247.00290; Found, 247.00273. Anhydride (trimer) of **8b** (5%): $^1$H NMR (400 MHz, $CD_3CN$) $\delta$ = 8.02 (d, $^3J_{H,H}$ = 8.5, 2H), 8.41 (d, $^3J_{H,H}$ = 8.0, 2H); $^{13}$C NMR (101 MHz, [$D_6$]DMSO) $\delta$ = 124.4–124.6 (m), 134.9; $^{19}$F NMR (377 MHz, $CD_3CN$) $\delta$ = 62.8 (d, $^2J_{F,F}$ = 147, 4F), 84.0–85.7 (m, 1F).

2. From diazonium salt **3b**[11]

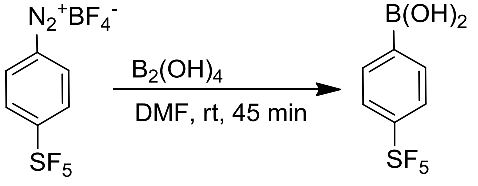

**3b** **8b**

To a solution of $B_2(OH)_4$ (180 mg, 2.0 mmol, 2.0 equiv) in DMF (4 mL), **3b** (318 mg, 1.0 mmol) was added portion-wise. The mixture was stirred for 45 min followed by addition of water (10 mL) and brine (4 mL), extraction of the product into EtOAc (3 × 10 mL). Combined organic phase was washed with brine (4 mL), water (2 × 4 mL) and brine (4 mL), dried ($MgSO_4$) and solvent was removed under reduced pressure. Column chromatography (EtOAc/hexane, 60:40 to 100:0) afforded **8b** as a mixture with estimated yield 25%, based on $^{19}$F NMR.

**Synthesis of potassium (pentafluorosulfanyl)phenyltrifluoroborates 9**

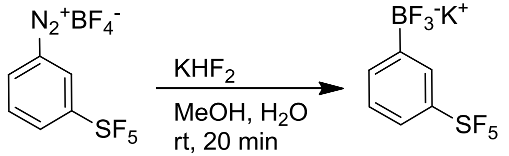

**3a** **9a**

**9a**: A solution of $KHF_2$ (780 mg, 10.0 mmol, 5 equiv) in water (3.6 mL) was added at rt to a solution of **3a** (660 mg, 2 mmol) in MeOH (8 mL). After stirring for 20 min, solvents were removed under reduced pressure (1 mm Hg, 70 °C, 5 min). The residuum was dissolved in MeCN (5 mL), filtered and the solid was washed with MeCN (2 × 2.5 mL). The filtrate was concentrated under reduced pressure and dissolved in EtOAc (1 mL). The product precipitated upon addition of $CH_2Cl_2$ (15 mL). Filtration, washing with $CH_2Cl_2$ (5 mL) and drying the solid under reduced pressure afforded **9a** as a white solid (520 mg, 84% yield): mp = 224–240 °C; FT-IR (film): $\nu_{max}$ (cm$^{-1}$) = 3064, 3021, 1713, 1702, 1598, 1479, 1412, 1371, 1233, 1219, 1112, 1099, 1002 (BF), 987 (BF), 899, 834 (SF); $^1$H NMR (400 MHz, [D$_6$]acetone) $\delta$ = 7.32 (t, $^3J_{H,H}$ = 7.7, 1H), 7.53 (ddd, $^3J_{H,H}$ = 8.2, $^4J_{H,H}$ = 2.5, 1.0, 1H), 7.68 (d, $^3J_{H,H}$ = 7.1, 1H), 7.90 (d, $^4J_{H,H}$ = 2.1, 1H); $^{11}$B NMR (128 MHz, [D$_6$]acetone) $\delta$ = 3.7 (quint, $^1J_{B,F}$ = 50.0); $^{13}$C NMR (101 MHz, [D$_6$]acetone) $\delta$ = 123.5 (quint, $^3J_{C,F}$ = 4.6), 127.9, 128.9–129.2 (m), 136.0; $^{19}$F NMR (377 MHz, [D$_6$]acetone) $\delta$ = -143.1 to -142.1 (m, 3F), 63.4 (d, $^2J_{F,F}$ = 147.4, 4F), 87.6–89.2 (m, 1F); Elem. anal.: Calc. for $C_6H_4BF_8KS$: C, 23.24, H, 1.30; S, 10.34%; Found: C, 23.27; H, 1.41; S, 10.44.

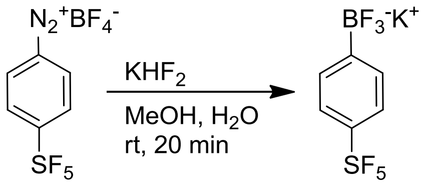

**3b** **9b**

**9b**: A solution of $KHF_2$ (780 mg, 10 mmol, 5 equiv) in water (3.6 mL) was added at rt to a solution of **3b** (660 mg, 2 mmol) in MeOH (8 mL). After stirring for 20 min, solvents were removed under reduced pressure (1 mm Hg, 70 °C, 5 min). The residue was dissolved in MeCN (10 mL) and filtered. The filtrate was concentrated under reduced pressure and dissolved in MeCN (5 mL). The product precipitated upon addition of $CH_2Cl_2$ (25 mL). Filtration, washing with $CH_2Cl_2$ (5 mL) and drying the solid under reduced pressure afforded **9b** as a white solid (579 mg, 93%): mp >220 °C (decomp.); FT-IR (film): $\nu_{max}$ (cm$^{-1}$) = 3097, 3055, 3037, 1714, 1389, 1371, 1234, 1225, 1024 (BF), 972 (BF), 922, 842 (SF), 819; $^1$H (400 MHz, [D$_6$]acetone): $\delta$= 7.56–7.66 (m, 4H); $^{11}$B NMR (128 MHz, [D$_6$]acetone) $\delta$ = 3.7 (br s); $^{13}$C NMR (101 MHz, [D$_6$]acetone) $\delta$ = 124.3 (quint, $^3J_{C,F}$ = 4.6), 132.8, 152.4–153.1 (m); $^{19}$F NMR (377 MHz, [D$_6$]acetone): $\delta$ = -142.8 to -142.1 (m, 3F), 63.7 (d, $^2J_{F,F}$ = 147.4, 4F), 87.6–89.1 (m, 1F); Elem. anal.: Calc. for $C_6H_4BF_8KS$: C, 23.24, H, 1.30; S, 10.34%; Found: C, 23.07; H, 1.58; S, 9.87.

## Trapping experiment, pyridine-promoted dediazoniation in the presence of iodobenzene

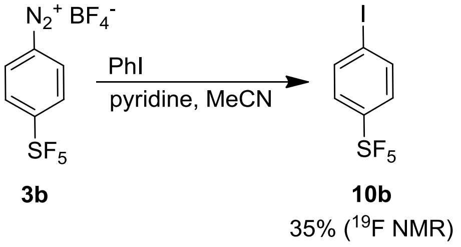


To a solution of **3b** (159 mg, 0.5 mmol) and PhI (408 mg, 2 mmol, 4 equiv) in MeCN (2 mL) cooled to −20 °C, pyridine (330 µL, 4.0 mmol, 4.0 equiv) was added dropwise. The mixture was slowly warmed to rt over 1.5 h and 4-nitro(pentafluorosulfanyl)benzene (0.5 mmol) was added as an internal standard. The yield was calculated based on $^{19}$F NMR spectroscopy.

## Competitive experiment, pyridine-promoted dediazoniation-borylation vs. -iodination in the presence of $B_2pin_2$ and iodobenzene

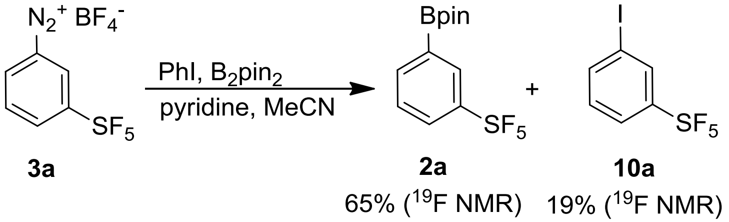


**3a** (318 mg, 1.0 mmol), $B_2pin_2$ (254 mg, 1 mmol, 1 equiv), PhI (204 mg, 1 mmol, 1 equiv) and MeCN (2 mL) were mixed and cooled to −30 °C. Pyridine (0.33 mL, 4 mmol, 4 equiv) was added to the stirred solution over half a minute and the mixture was slowly warmed to rt over 2 h. 4-Nitro(pentafluorosulfanyl)benzene (0.5 mmol) was then added as an internal standard and the yield was calculated based on $^{19}$F NMR spectroscopy.

## Synthesis of aryl iodides 10, General procedure

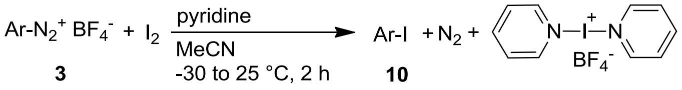


Pyridine (0.33 mL, 4.0 mmol, 4.0 equiv) was added over half a minute to a mixture of **3** (1.0 mmol), $I_2$ (279 mg, 1.1 mmol, 1.1 equiv) and MeCN (2 mL) cooled to −30 °C. The reaction mixture was warmed to rt over 2 h, solvent was removed under reduced pressure, $Et_2O$ (20 mL) was added. The ether phase was washed with aqueous $Na_2S_2O_3$ (1 M, 5 mL), brine (5 mL), dried ($MgSO_4$), and solvent was removed under reduced pressure. Purification by column chromatography afforded **10**.

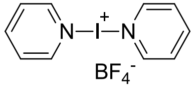


Bis(pyridine)iodonium tetrafluoroborate:[12] isolated as a pale brown solid by the addition of $Et_2O$ to the MeCN solution of the crude reaction mixture, filtration and drying under reduced pressure: $^1$H NMR (400 MHz, $CD_2Cl_2$): $\delta$ = 7.53–7.69 (m, 4H), 8.22 (tt, $^3J_{HH}$ = 7.6, $^4J_{HH}$ = 1.5, 2H), 8.70–8.82 (m, 4H); $^{11}$B NMR (128 MHz, $CD_2Cl_2$): $\delta$ = -1.3 (s); $^{13}$C NMR (101 MHz, $CD_2Cl_2$): $\delta$ = 128.4, 142.5, 149.9; $^{19}$F NMR (376 MHz, $CD_2Cl_2$): $\delta$ = -152.3 (d, $^1J_{BF}$ = 1.7); HRMS (ESI$^+$): $m/z$ = Calc. for $C_{10}H_{10}N_2I$ [M]$^+$, 284.98832; Found, 284.98846.

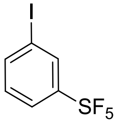


**10a**:[10] Prepared according to the general procedure from **3a** (318 mg, 1.0 mmol), $I_2$ (279 mg, 1.1 mmol, 1.1 equiv), MeCN (2 mL) and pyridine (0.33 mL, 4.0 mmol, 4.0 equiv) affording **10a** as a colorless liquid (278 mg, 84% yield): $R_f$ = 0.76 (hexane); $^1$H NMR (400 MHz, $CDCl_3$): $\delta$ = 7.21 (tt, $^3J_{H,H}$ = 8.0, $^5J_{H,H}$ = 0.8, 1H), 7.74 (ddd, $^3J_{H,H}$ = 8.3, $^4J_{H,H}$ = 2.1, 0.8, 1H), 7.83–7.88 (m, 1H), 8.08 (t, $^4J_{H,H}$ = 1.9, 1H); $^{13}$C NMR (101 MHz, $CDCl_3$): $\delta$ = 93.1, 125.2 (quint, $^3J_{C,F}$ = 4.7), 130.3, 134.7 (quint, $^3J_{C,F}$ = 4.7),

140.6; $^{19}$F NMR (377 MHz, $CDCl_3$) $\delta$ = 62.3 (d, $^2J_{F,F}$ = 150.3, 4F), 81.8–83.4 (m, 1F).

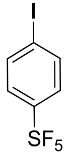

**10b**:[10] Prepared according to the general procedure from **3b** (318 mg, 1.0 mmol), $I_2$ (279 mg, 1.1 mmol, 1.1 equiv), MeCN (2 mL) and pyridine (0.33 mL, 4.0 mmol, 4.0 equiv) affording **10b** as a colorless solid (279 mg, 85% yield), or on 2 mmol scale (580 mg, 88% yield), or on 1 mmol scale using collidine (0.67 mL, 4 mmol, 4 equiv) instead of pyridine (269 mg, 82% yield): $R_f$ = 0.76 (hexane); $^1$H NMR (400 MHz, $CDCl_3$): $\delta$ = 7.46–7.49 (m, 2H), 7.80–7.83 (m, 2H); $^{13}$C NMR (101 MHz, $CDCl_3$): $\delta$ = 98.2, 127.5 (quint, $^3J_{C,F}$ = 4.8), 137.9; $^{19}$F NMR (377 MHz, $CDCl_3$) $\delta$ = 62.3 (d, $^2J_{F,F}$ = 150.3, 4F), 82.2–83.8 (m, 1F).

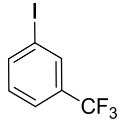

**10c**:[13] Prepared according to the general procedure from **3c** (260 mg, 1.0 mmol), $I_2$ (279 mg, 1.1 mmol, 1.1 equiv), MeCN (2 mL) and pyridine (0.33 mL, 4.0 mmol, 4.0 equiv) affording **10c** as a colorless liquid (142 mg, 52% yield), or on 1 mmol scale using collidine (0.67 mL, 4 mmol, 4 equiv) instead of pyridine (162 mg, 60% yield): $R_f$ = 0.73 (hexane); $^1$H NMR (400 MHz, $CDCl_3$): $\delta$ = 7.20–7.24 (m, 1H), 7.58–7.60 (m, 1H), 7.88–7.90 (m, 1H), 7.96–7.96 (m, 1H); $^{13}$C NMR (101 MHz, $CDCl_3$): $\delta$ = 93.8, 122.9 (q, $^1J_{F,C}$ = 273), 124.5 (q, $^3J_{F,C}$ = 3.9), 130.4, 132.4 (q, $^2J_{F,C}$ = 32.9), 134.3 (q, $^3J_{F,C}$ = 3.7), 140.9 (q, $^5J_{F,C}$ = 1.5); $^{19}$F NMR (376 MHz, $CDCl_3$): $\delta$ = -63.4 (s).

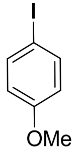

**10d**:[14] Prepared according to the general procedure from **3d** (222 mg, 1.0 mmol), $I_2$ (279 mg, 1.1 mmol, 1.1 equiv), MeCN (2 mL) and pyridine (0.33 mL, 4.0 mmol, 4.0 equiv) affording **10d** as a white solid (20 mg, 9% yield), or using collidine (0.67 mL, 4 mmol, 4 equiv) instead of pyridine (26 mg, 11% yield): $R_f$ = 0.30 (hexane); $^1$H NMR (400 MHz, $CDCl_3$): $\delta$ = 3.77 (s, 3H), 6.66–6.69 (m, 2H), 7.53–7.57 (m, 2H); $^{13}$C NMR (101 MHz, $CDCl_3$): $\delta$ = 55.3, 82.6, 116.3, 138.1, 159.4.

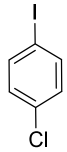

**10e**:[13] Prepared according to the general procedure from **3e** (226 mg, 1.0 mmol), $I_2$ (279 mg, 1.1 mmol, 1.1 equiv), MeCN (2 mL) and pyridine (0.33 mL, 4.0 mmol, 4.0 equiv) affording **10e** as a white solid (110 mg, 46% yield): $R_f$ = 0.77 (hexane); $^1$H NMR (400 MHz, $CDCl_3$): $\delta$ = 7.07–7.10 (m, 2H), 7.59–7.63 (m, 2H); $^{13}$C NMR (101 MHz, $CDCl_3$): $\delta$ = 91.1, 130.5, 134.2, 138.7.

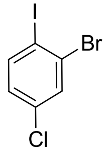

**10f**:[15] Prepared according to the general procedure from **3f** (305 mg, 1.0 mmol), $I_2$ (279 mg, 1.1 mmol, 1.1 equiv), MeCN (2 mL) and pyridine (0.33 mL, 4.0 mmol, 4.0 equiv) affording **10f** as a colorless liquid (198 mg, 63% yield): $R_f$ = 0.85 (hexane); $^1$H NMR (400 MHz, $CDCl_3$): $\delta$ = 6.99 (dd, $^3J_{H,H}$ = 8.5, $^4J_{H,H}$ = 2.4, 1H), 7.62 (d, $^4J_{H,H}$ = 2.4, 1H), 7.76 (d, $^3J_{H,H}$ = 8.5, 1H); $^{13}$C NMR (101 MHz, $CDCl_3$): $\delta$ = 98.6, 128.8, 130.4, 132.4, 135.0, 140.8.

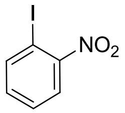

**10g**:[16] Prepared according to the general procedure from **3g** (237 mg, 1.0 mmol), $I_2$ (279 mg, 1.1 mmol, 1.1 equiv), MeCN (2 mL) and pyridine (0.33 mL, 4.0 mmol, 4.0 equiv) affording **10f** as a yellow liquid (222 mg, 89% yield): $R_f$ = 0.29 (EtOAc/hexane, 5:95); $^1$H NMR (400 MHz, $CDCl_3$): $\delta$ = 7.26–7.30 (m, 1H), 7.48–7.52 (m, 1H), 7.85 (dd, $^3J_{H,H}$ = 8.1, $^4J_{H,H}$ = 1.6, 1H), 8.04 (dd, $^3J_{H,H}$ = 7.8, $^4J_{H,H}$ = 1.4, 1H); $^{13}$C NMR (101 MHz, $CDCl_3$): $\delta$ = 86.2, 125.4, 129.0, 133.3, 141.9.

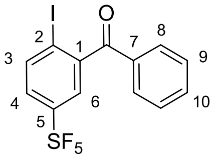

**10i**: Prepared according to the general procedure from **3i** (422 mg, 1.0 mmol), $I_2$ (279 mg, 1.1 mmol, 1.1 equiv), MeCN (2 mL) and pyridine (0.33 mL, 4.0 mmol, 4.0 equiv) affording **10i** as a white solid (398 mg, 92% yield): $R_f$ = 0.27 ($Et_2O$/hexane, 20:80); mp = 107–110 °C; FT-IR (film): $\nu_{max}$ ($cm^{-1}$) = 3091, 3072, 3007, 1668 (C=O), 1595, 1584, 1458, 145, 1392, 1315, 1292, 1258, 1170, 1022, 842 (SF); $^1$H NMR (400 MHz, $CDCl_3$): $\delta$ = 7.49–7.53 (m, 2H, C9H), 7.56 (dd, $^3J_{H,H}$ = 8.6, $^4J_{H,H}$ = 2.5, 1H, C4H), 7.63–7.69 (m, 2H, C6H, C10H), 7.79–7.83 (m, 2H, C8H), 8.05 (d, $^3J_{H,H}$ = 8.8, 1H; C3H); $^{13}$C NMR (101 MHz, $CDCl_3$): $\delta$ = 96.4 (C2), 125.6 (quint, $^3J_{C,F}$ = 4.6, C6), 128.0 (quint, $^3J_{C,F}$ = 4.7, C4), 129.0, 130.4, 134.4 (C10), 134.6 (C7), 140.3 (C3), 145.1 (C1), 153.5 (quint, $^2J_{C,F}$ = 18.3, C5), 195.2 (CO); $^{19}$F NMR (377 MHz, $CDCl_3$): $\delta$ = 62.5 (d, $^2J_{F,F}$ =150.9, 4F), 81.1–82.8 (m, 1F); MS ($EI^+$): $m/z$ (%) = 435 (15), 434 (100) $[M]^+$, 357 (32) $[M - Ph]^+$, 152 (11), 105 (91), 94 (10), 77 (43), 75 (14), 51 (11); HRMS ($ESI^+$): $m/z$ = Calc. for $C_{13}H_8F_5INaOS$ $[M + Na]^+$, 456.91529; Found, 456.91527.

**Synthesis of 1-bromo-4-(pentafluorosulfanyl)benzene**

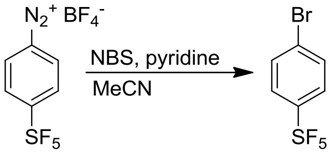

3b

35%

To a mixture of **3b** (318 mg, 1 mmol) and NBS (178 mg, 1 mmol, 1 equiv) in MeCN (2 mL), pyridine (0.33 mL, 4 mmol, 4 equiv) was added at rt and the reaction was stirred overnight. Solvent was removed under reduced pressure, water (10 mL) was added and product was extracted into $Et_2O$ (3 × 10 mL). Combined organic phase was washed with 1 M HCl (5 mL), brine (5 mL), dried ($MgSO_4$), and solvent was removed under reduced pressure. Column chromatography (hexane) provided product as a colorless liquid (99 mg, 35% yield). Characterization corresponds to literature.[17]

**Synthesis of (pentafluorosulfanyl)benzenes 6 by hydrodediazoniation of aryldiazonium tetrafluoroborates, General procedure**

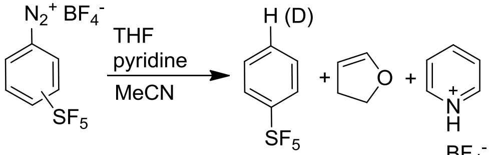

3a (3-$SF_5$)
3b (4-$SF_5$)
6 (6-D) 11

A solution of **3** (159 mg, 0.5 mmol) in THF (0.50 mL) and MeCN (2 mL) was cooled to −25 °C and pyridine (165 µL, 2.0 mmol, 4.0 equiv) as added over half a minute. The reaction mixture was warmed to rt over 2 h. 4-Nitro(pentafluorosulfanyl)benzene (ca 0.5 mmol) was then added as an internal standard and the yield was calculated based on $^{19}$F NMR. Side products dihydrofuran (**11**) was observed by GCMS: MS ($EI^+$): $m/z$ (%) = 70 (100), 69 (52), 42 (25), 41(85), 40 (23), 39 (73), 38 (13) and pyridinium tetrafluoroborate by $^{11}$B and $^{19}$F NMR.

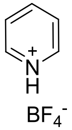

Prepared independently by mixing $H_3BO_3$ (248 mg, 4.0 mmol, 1 equiv) with aqueous 40% HF (800 mg, 16.0 mmol, 4 equiv) followed by addition of pyridine (411 mg, 5.2 mmol, 1.3 equiv) at rt. Addition of MeCN (4 mL), MeOH (1 mL) and $Et_2O$ (25 mL) filtration and drying the solid under reduced pressure provided product as white solid (528 mg, 60% yield): $^{11}$B NMR (128 MHz, $CD_3CN$): $\delta$ = -0.5 (s); $^{19}$F NMR (376 MHz, $CD_3CN$): $\delta$ = -150.2 (s).

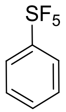

**6**: Prepared according to the general procedure from **3a** (159 mg, 0.5 mmol), THF (0.50 mL), MeCN (2 mL) and pyridine (165 µL, 2.0 mmol, 4.0 equiv) in 75% $^{19}$F NMR yield: $^{19}$F NMR (377 MHz, $CDCl_3$): $\delta$ 63.2 (d, $^2J_{F,F}$ = 147.7, 4F), 85.0–86.6 (m, 1F); MS ($EI^+$): $m/z$ (%) = 204 (71), 96 (94), 89(22), 77 (100), 74 (10), 70 (10), 51 (44), 50 (23); or from **3b** (159 mg, 0.5 mmol), THF (0.50 mL), MeCN (2 mL) and pyridine (165 µL, 2.0 mmol, 4.0 equiv) in 70% $^{19}$F NMR yield.

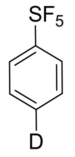


**6**-D: Prepared according to the general procedure from **3b** (159 mg, 0.5 mmol), [$D_8$]THF (0.50 mL), MeCN (2 mL) and pyridine (165 µL, 2.0 mmol, 4.0 equiv) in 48% $^{19}$F NMR yield and 77-82% deuterium enrichment determined by GCMS; $^{19}$F (377 MHz, $CDCl_3$): $\delta = 63.2$ (d, $^2J_{F,F}$ = 148.0, 4F), 85.0–86.6 (m, 1F); MS (EI$^+$): *m/z* (%) (**6** + **6**-D) = 205 (100), 204 (22), 127 (11), 97 (99), 96 (25), 89 (30), 78 (100), 77 (28), 52 (24), 51 (33), 50 (18).

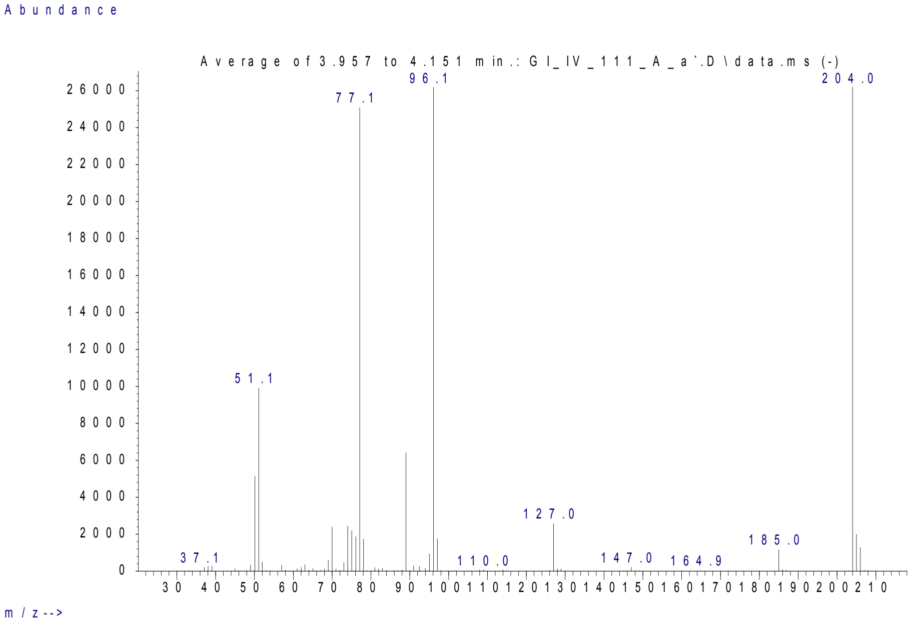


**Figure S8:** MS (EI$^+$) spectrum of **6**.

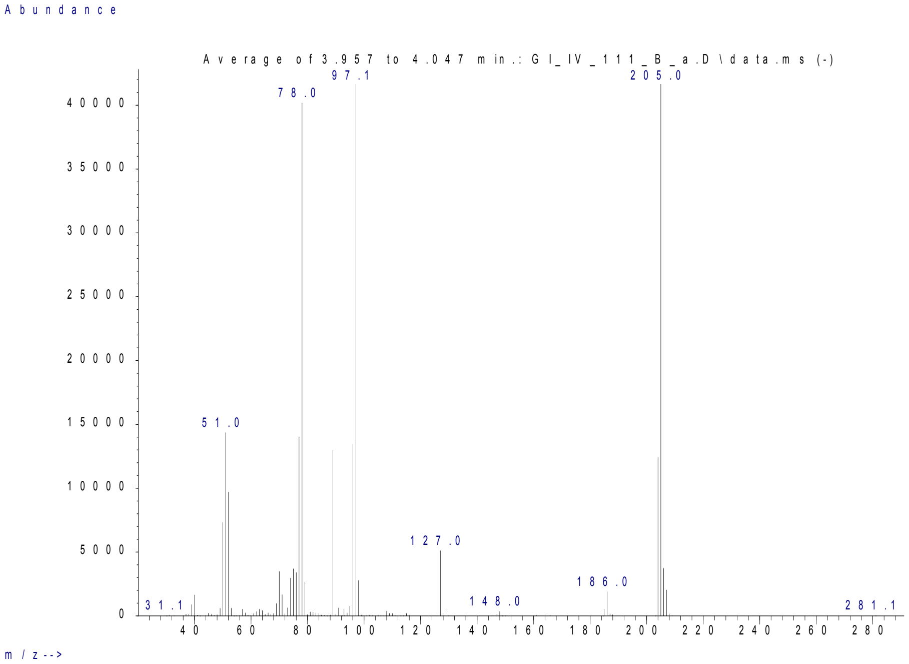


**Figure S9:** MS (EI$^+$) spectrum of a mixture of **6** and **6**-D in ca 20:80 ratio.

## KIE experiment

A solution of **3b** (159 mg, 0.5 mmol) in a mixture THF (0.25 mL), [$D_8$]THF (0.25 mL) and MeCN (2 mL) was cooled to −25 °C. Pyridine (165 µL, 2.0 mmol, 4.0 equiv) was added over half a minute and the reaction mixture was warmed to rt over 2 h. 4-Nitro(pentafluorosulfanyl)benzene (0.5 mmol) was then added as an internal standard and the yield was calculated based on $^{19}$F NMR. A mixture of **6** and **6**-D formed in 62% $^{19}$F NMR yield and **6**:**6**-D ratio of 5.5 (determined by GC–MS, Figures S10 and S11).

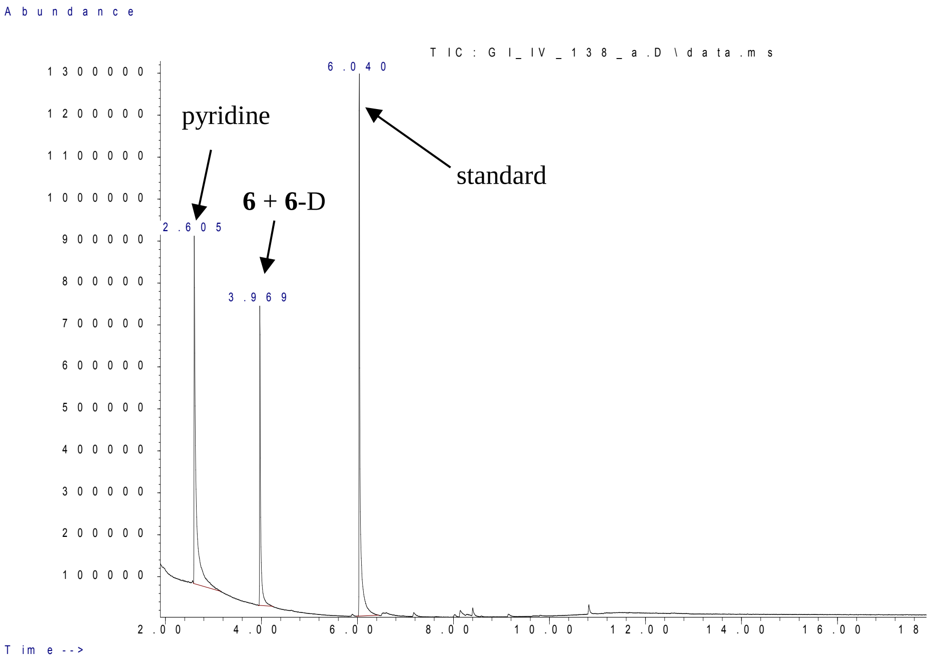


**Figure S10:** GC chromatogram of the crude product mixture after the KIE experiment.

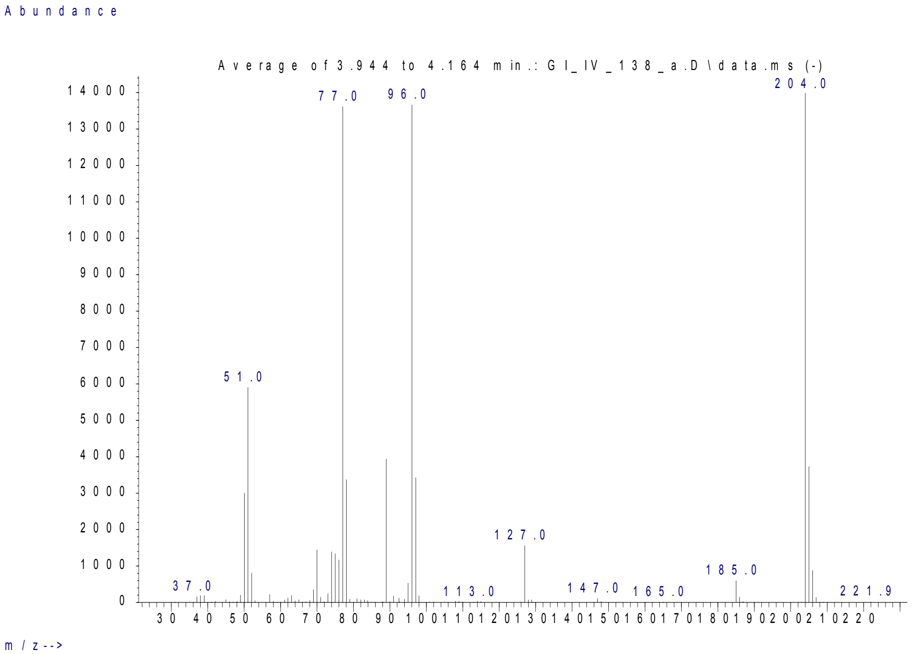


**Figure S11:** MS ($EI^+$) spectrum of a mixture of **6** + **6**-D.

**3a:** $^1$H NMR (400 MHz, $CD_3CN$)

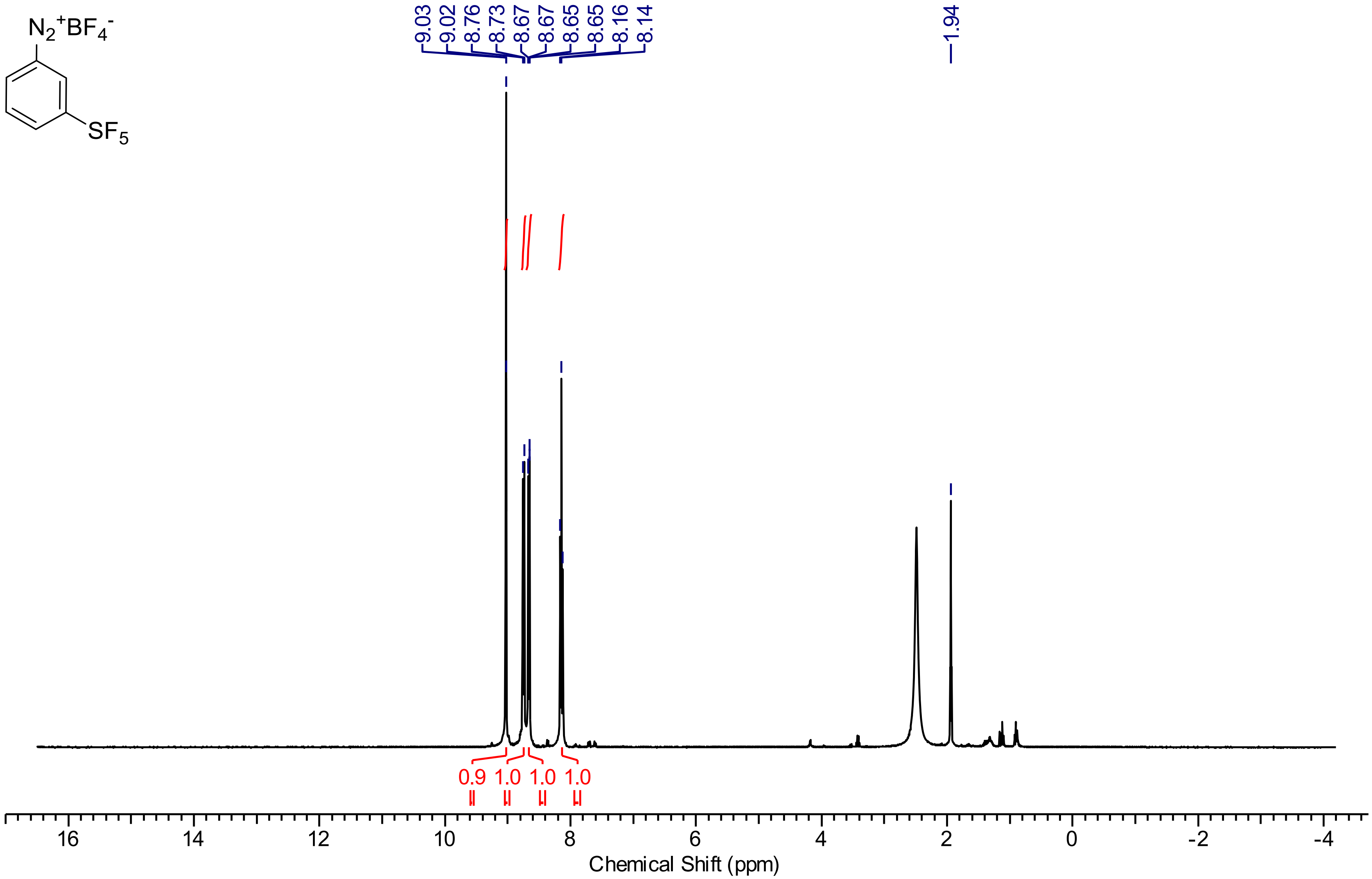

**3a:** $^{1}$H NMR (400 MHz, [D$_6$]acetone)

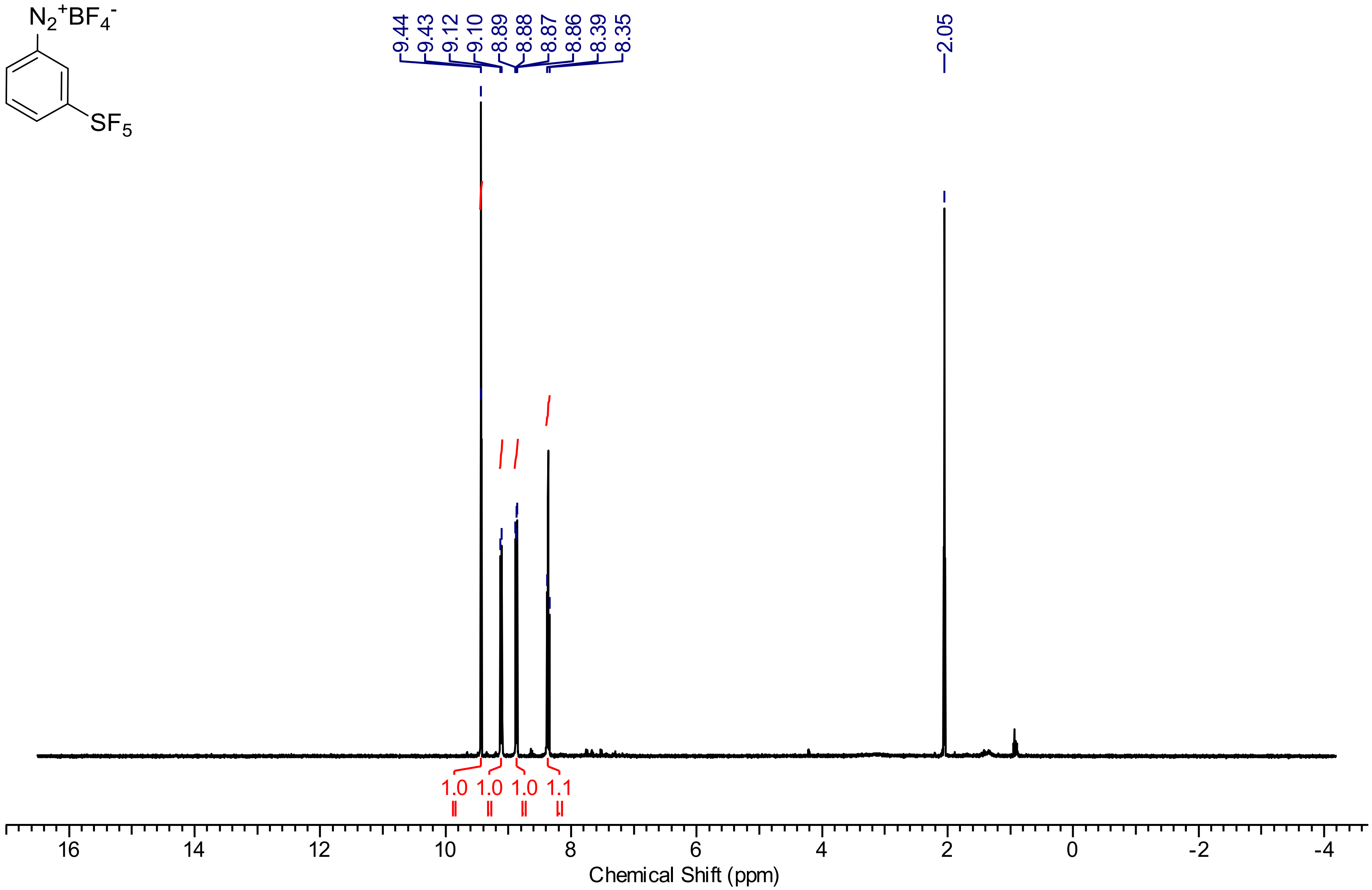

**3a**: $^{11}B$ NMR (128 MHz, $CD_3CN$)

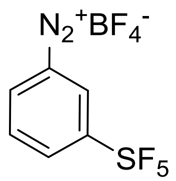


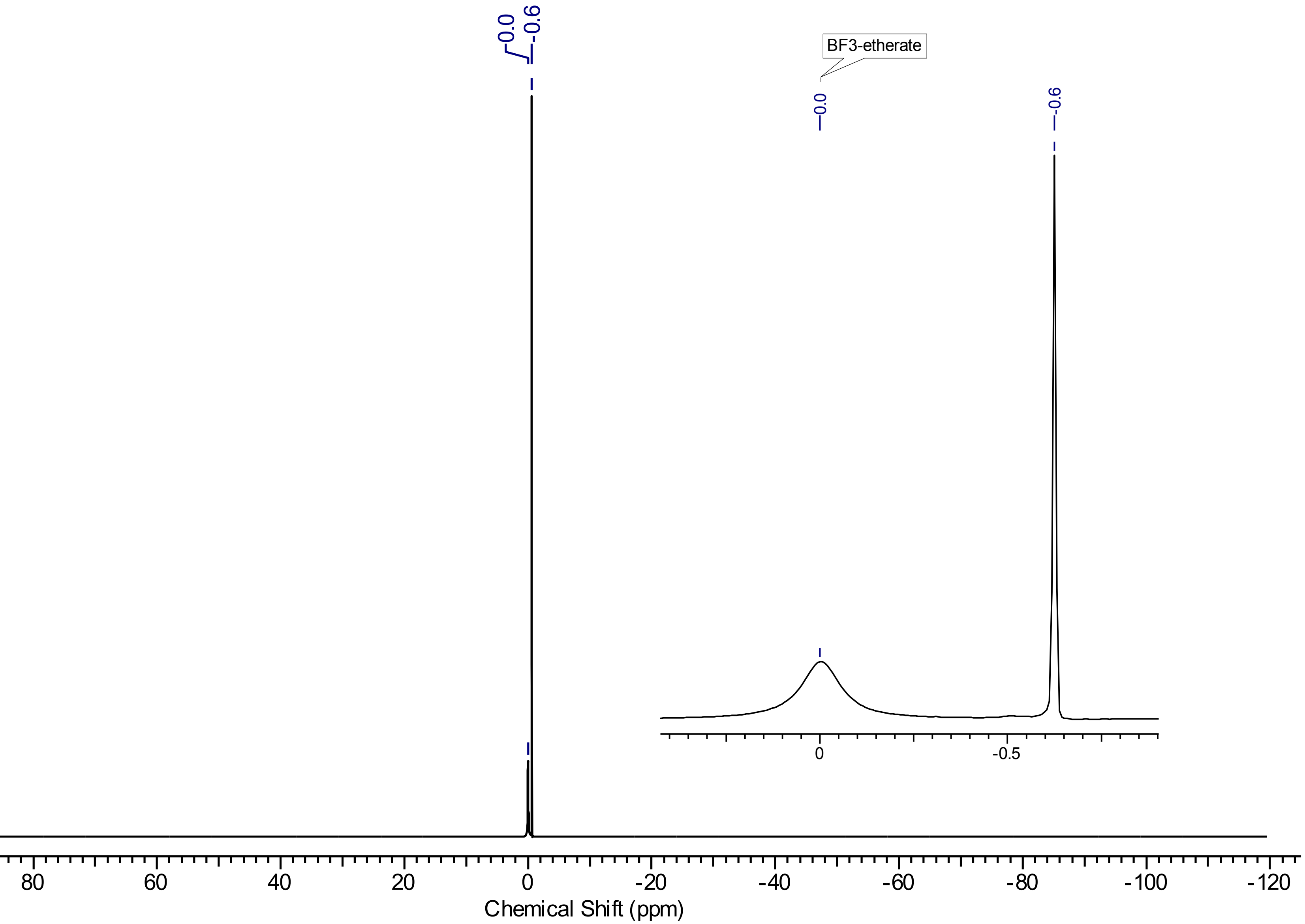

**3a**: $^{13}$C NMR (101 MHz, $CD_3CN$)

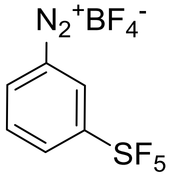


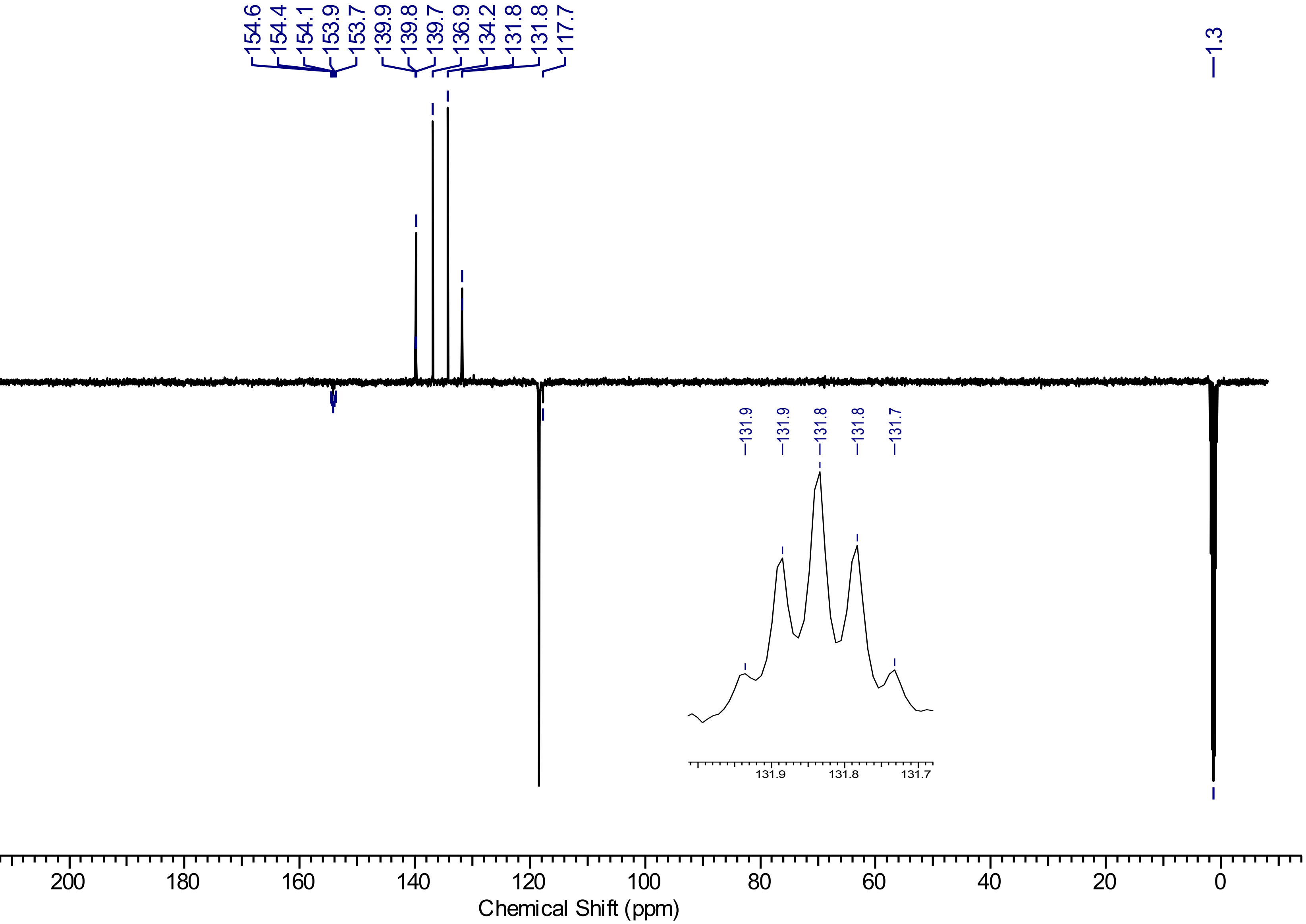

**3a**: $^{19}$F NMR (377 MHz, [D$_6$]acetone)

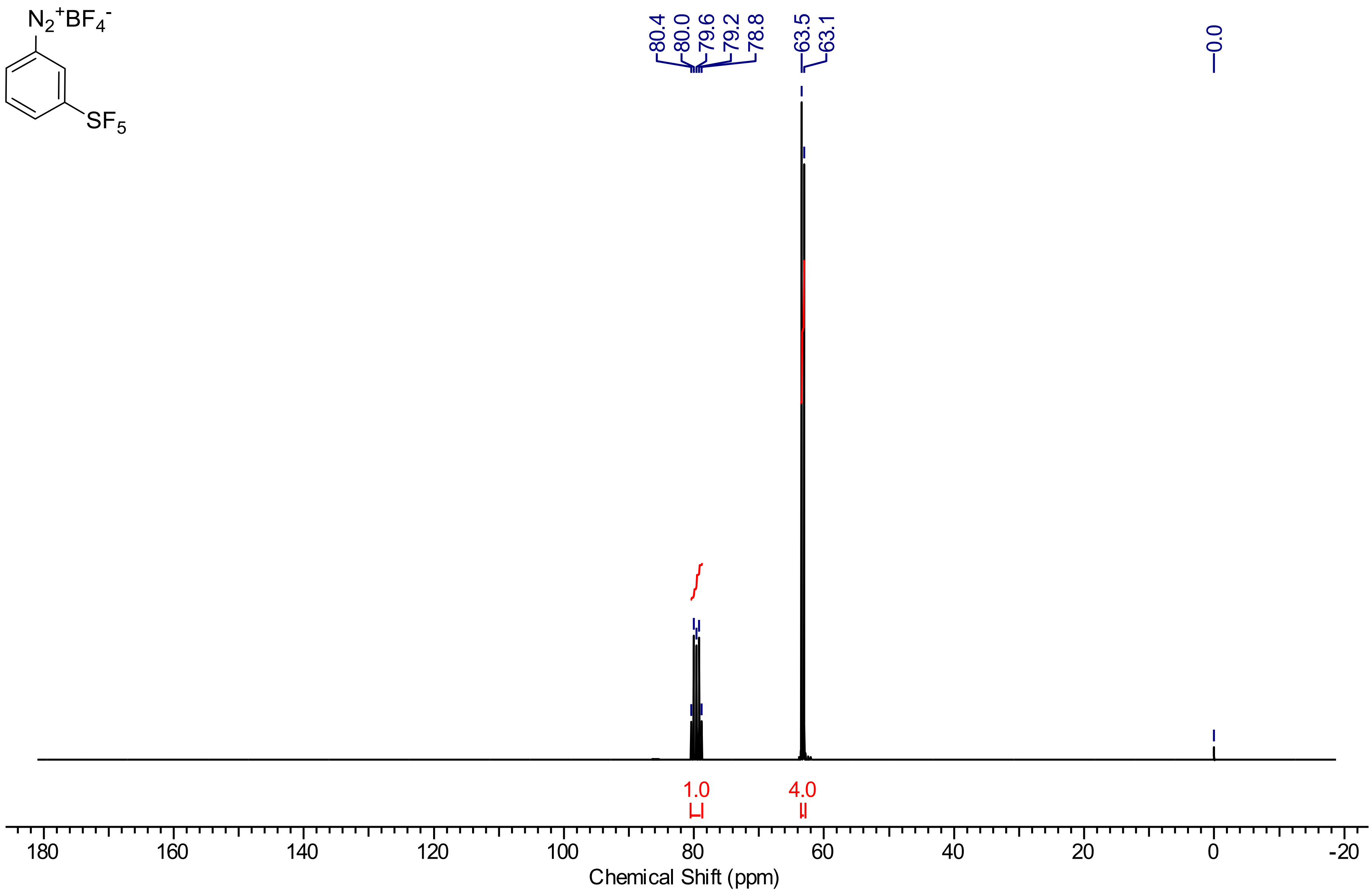

**3a**: $^{19}F$ NMR (377 MHz, $[D_6]$acetone)

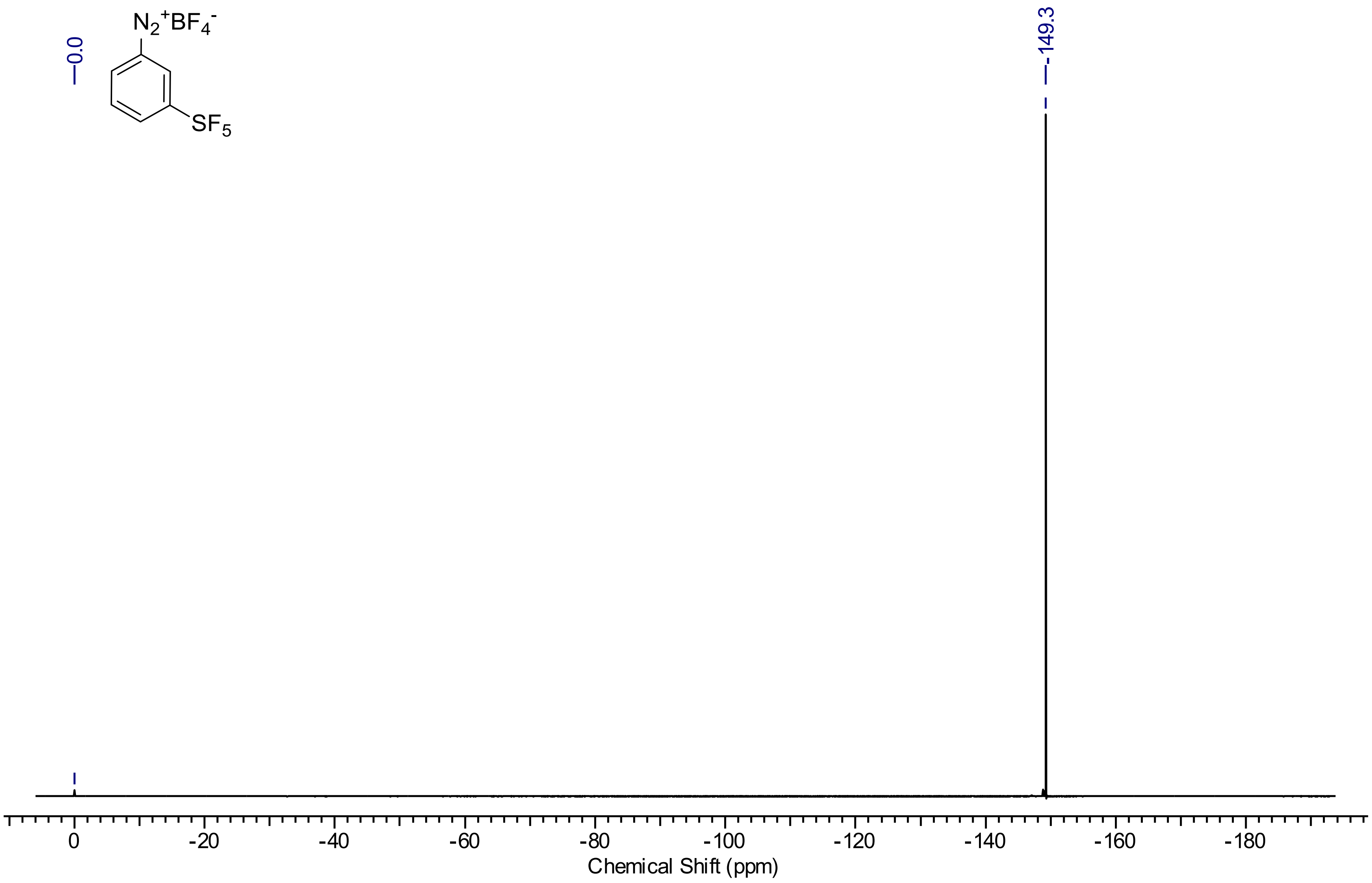

**3b**: $^{1}H$ NMR (400 MHz, $CD_3CN$)

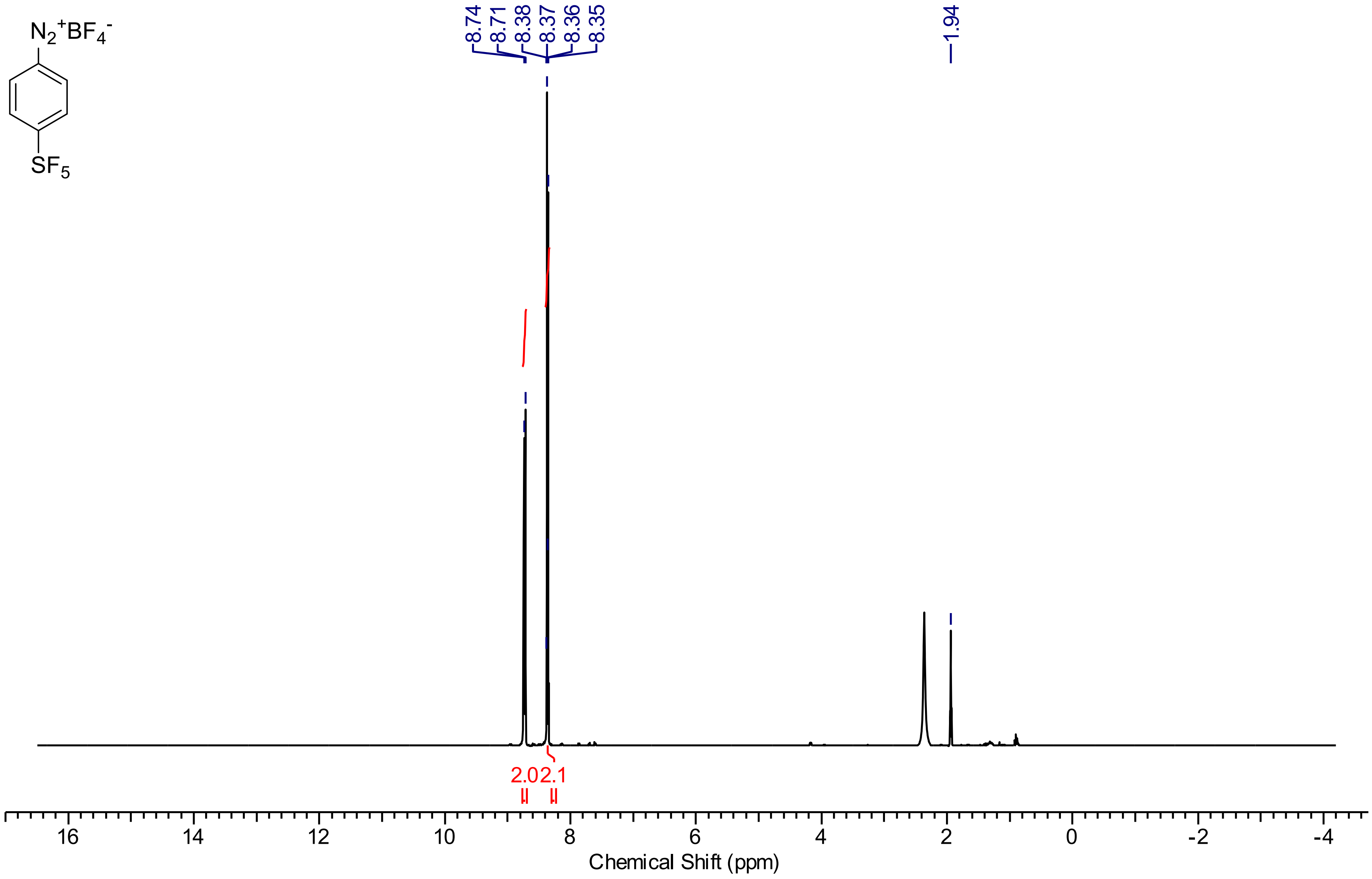

**3b**: $^{1}$H NMR (400 MHz, [D$_6$]acetone)

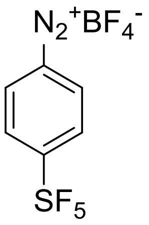


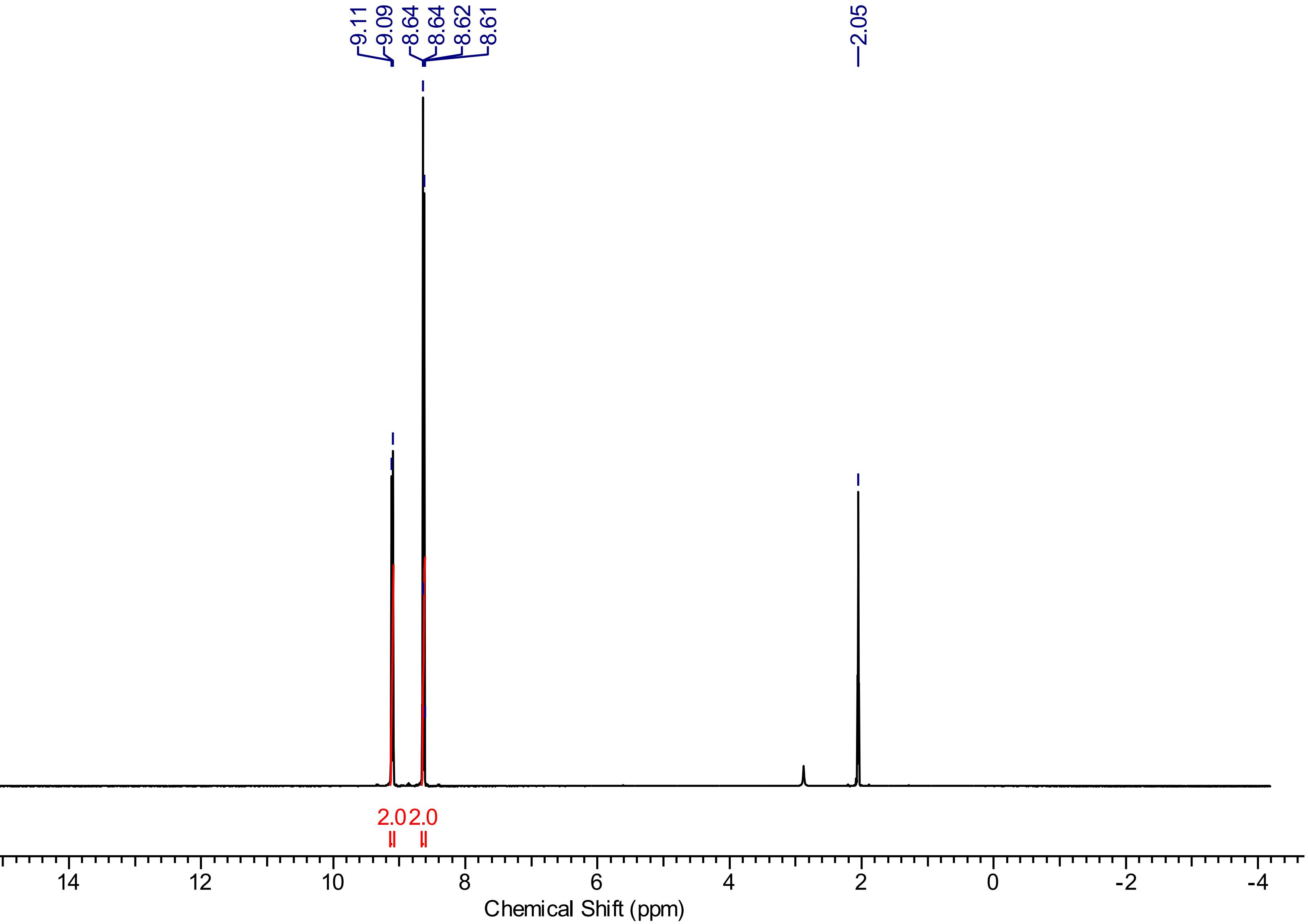

**3b**: $^{11}B$ NMR (128 MHz, $[D_6]$acetone)

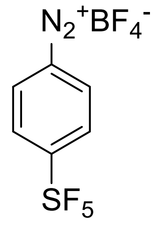

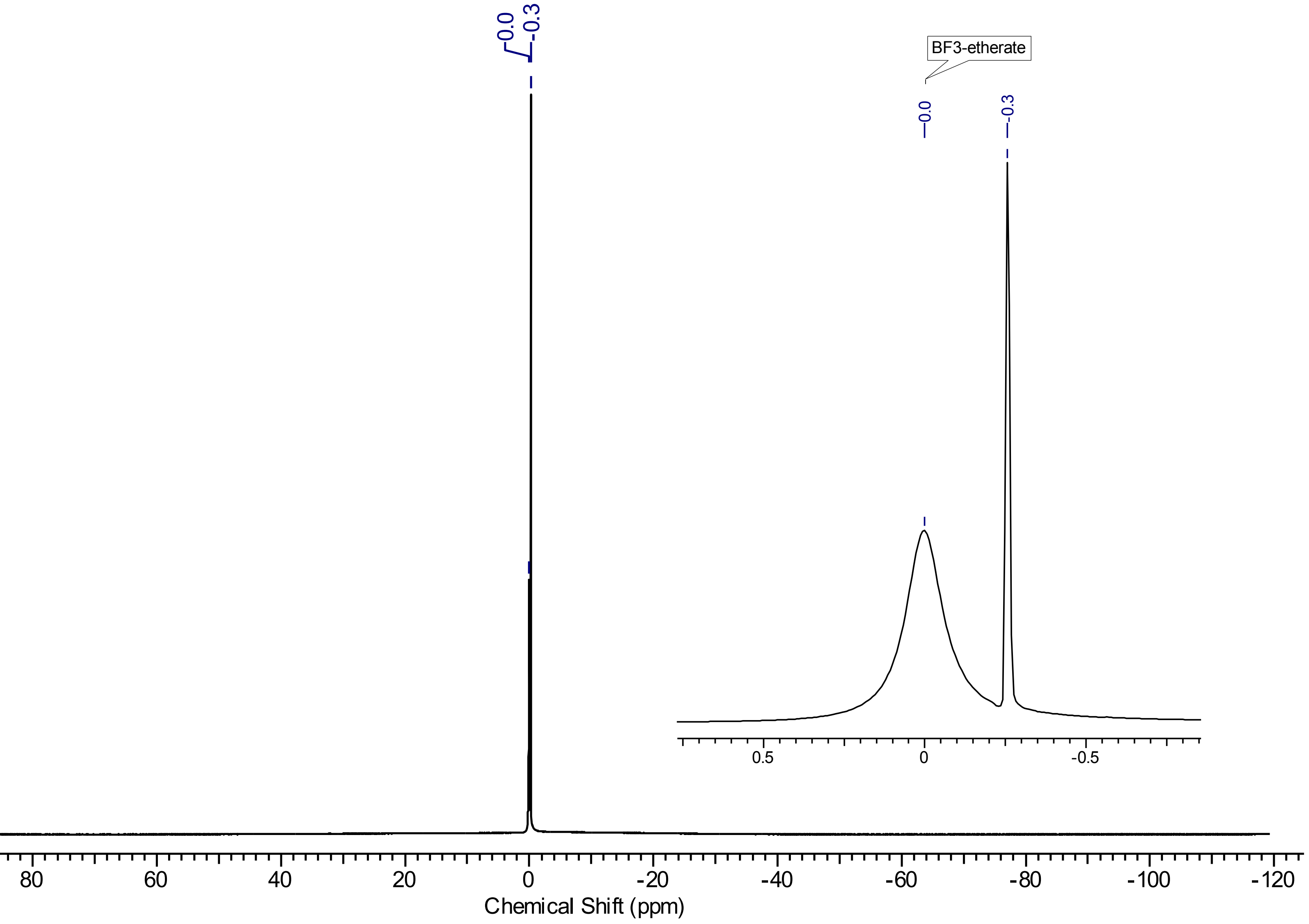

**3b**: $^{13}$C NMR (101 MHz, $CD_3CN$)

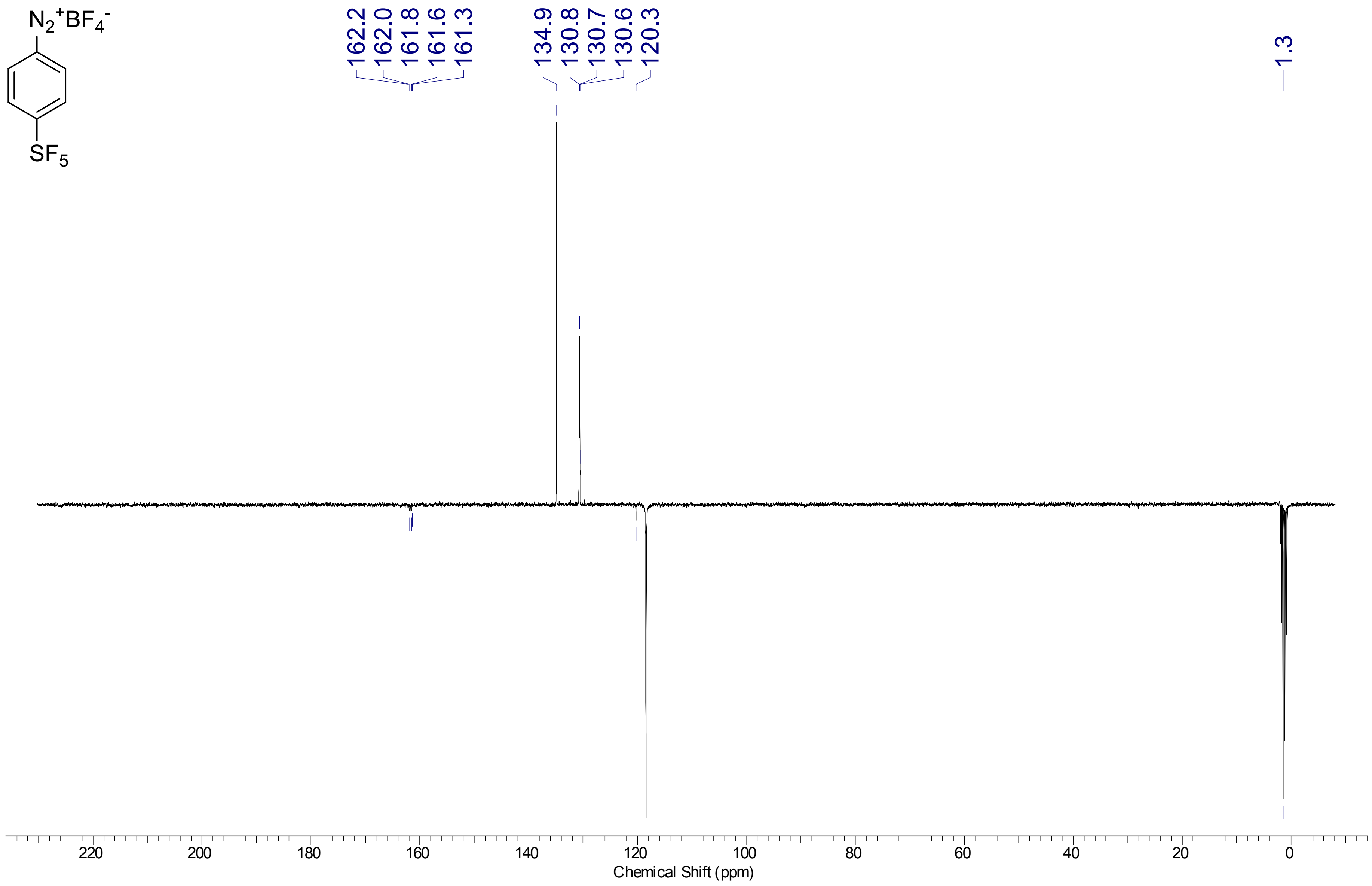

**3b**: $^{19}$F NMR (377 MHz, $CD_3CN$)

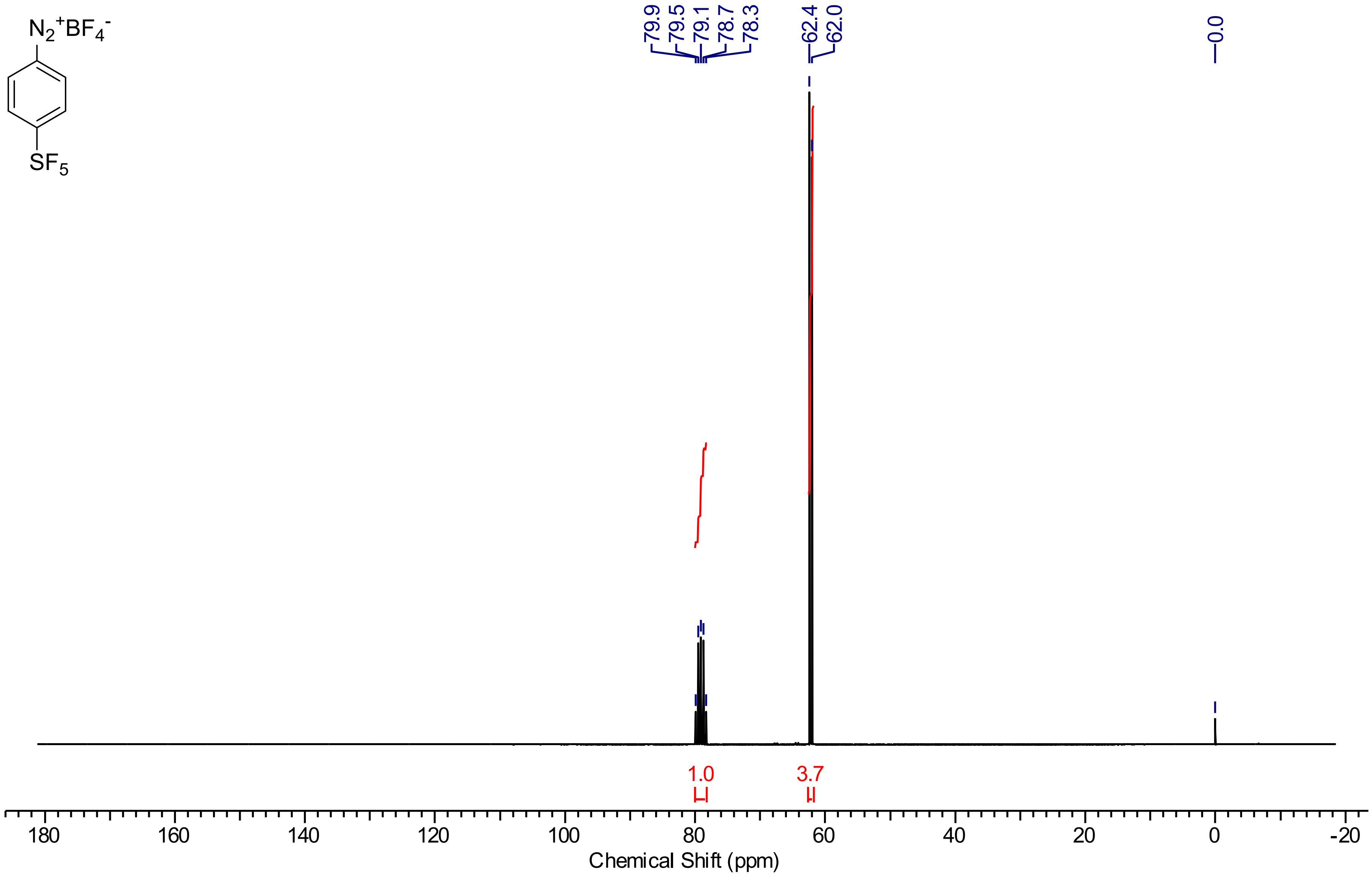

**3b**: $^{19}$F NMR (377 MHz, $CD_3CN$)

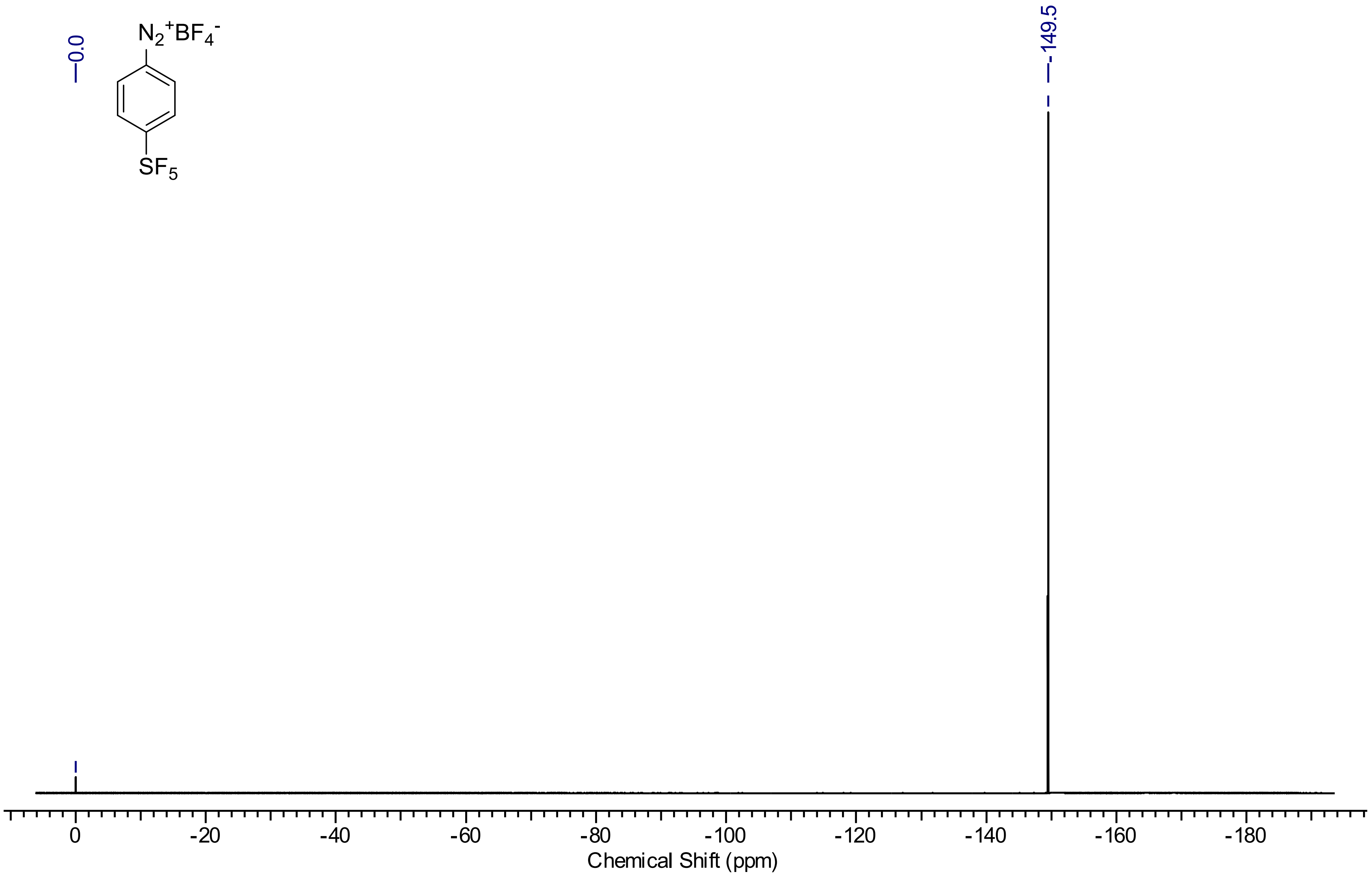

**3b**: $^{19}$F NMR (377 MHz, [D$_6$]acetone)

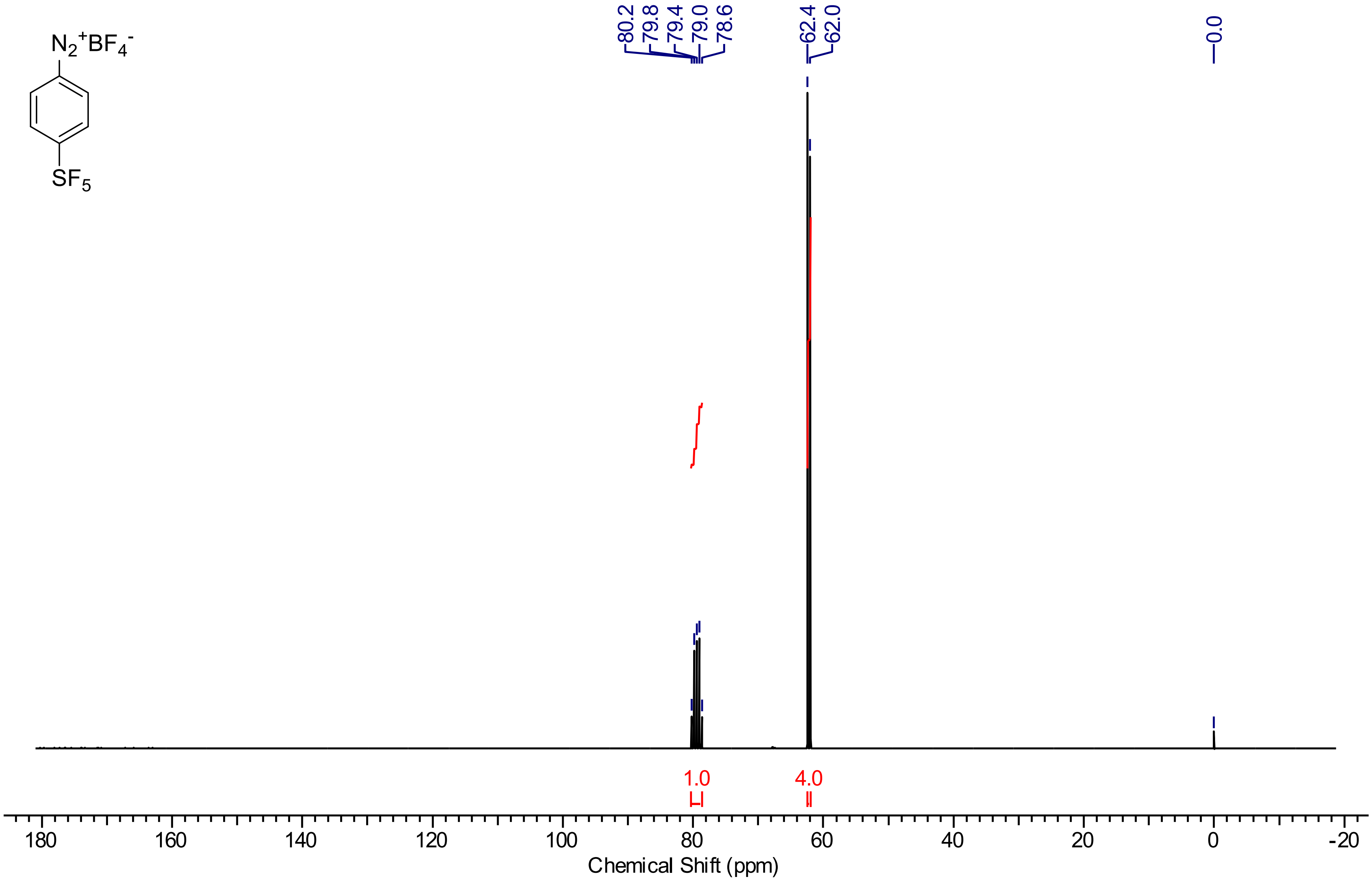

**3b**: $^{19}$F NMR (376 MHz, [D$_6$]acetone)

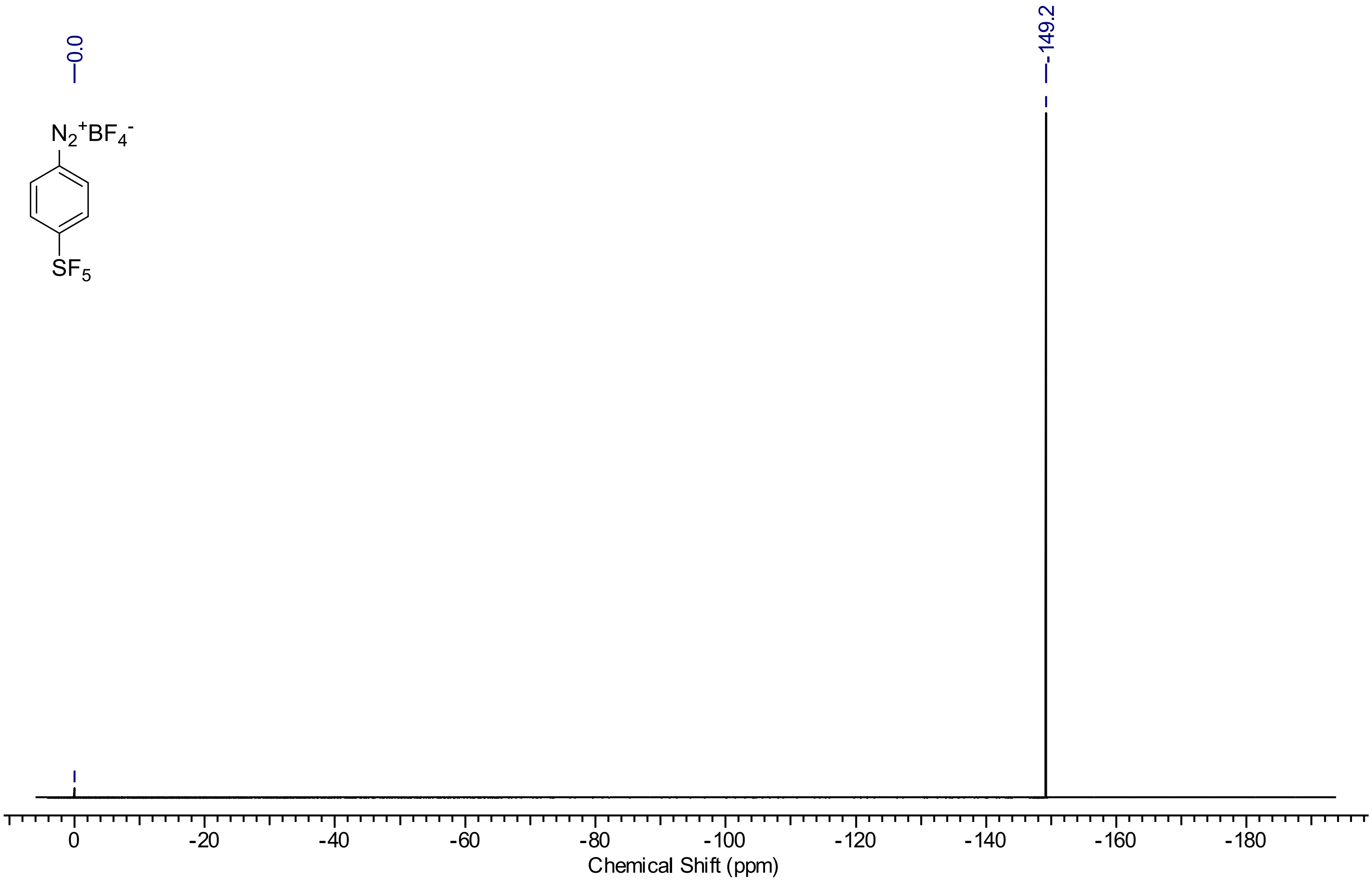

**3c**: $^{1}$H NMR (400 MHz, $CD_3CN$)

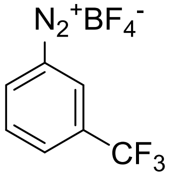


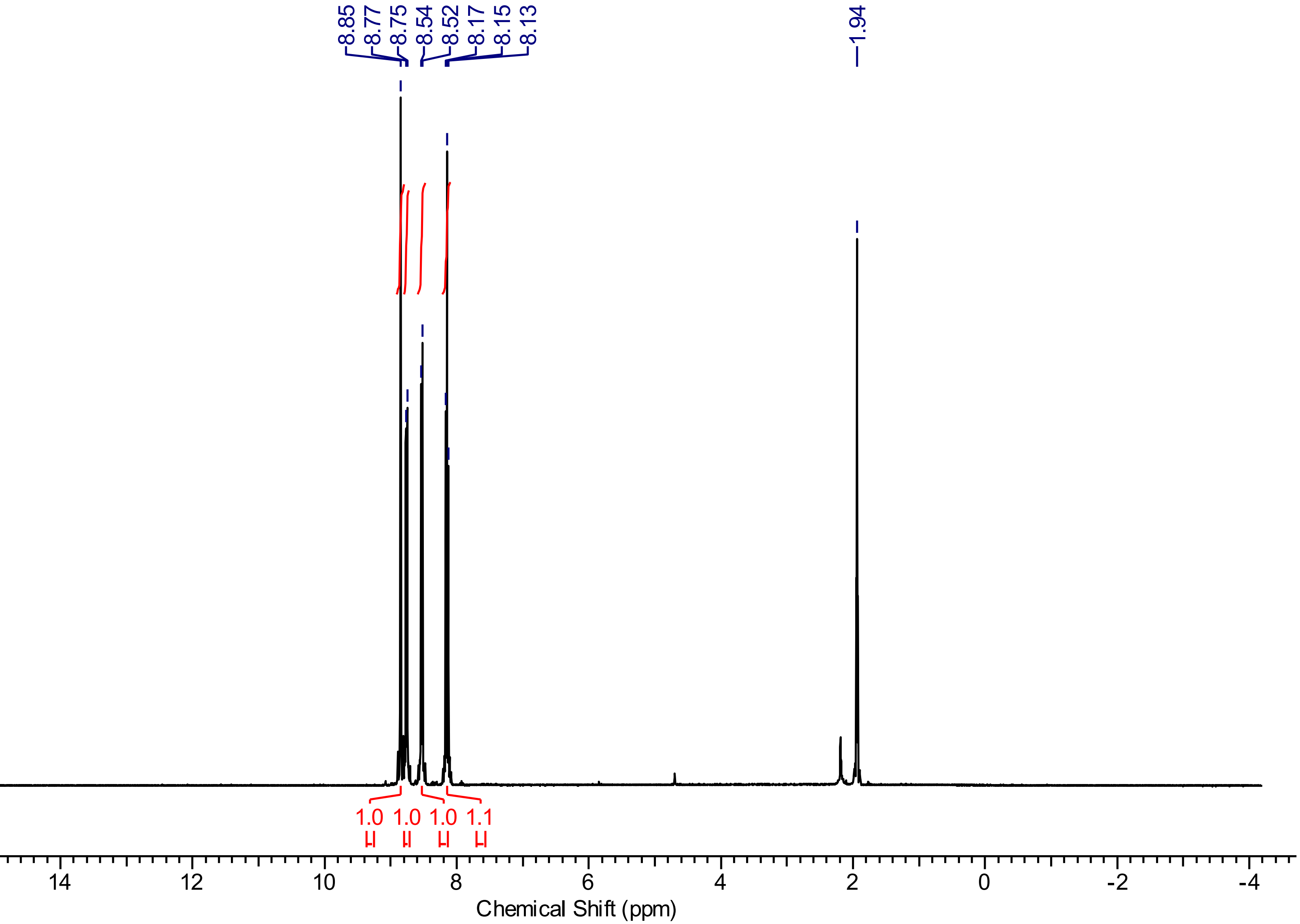

**3c**: $^{13}$C NMR (101 MHz, $CD_3CN$)

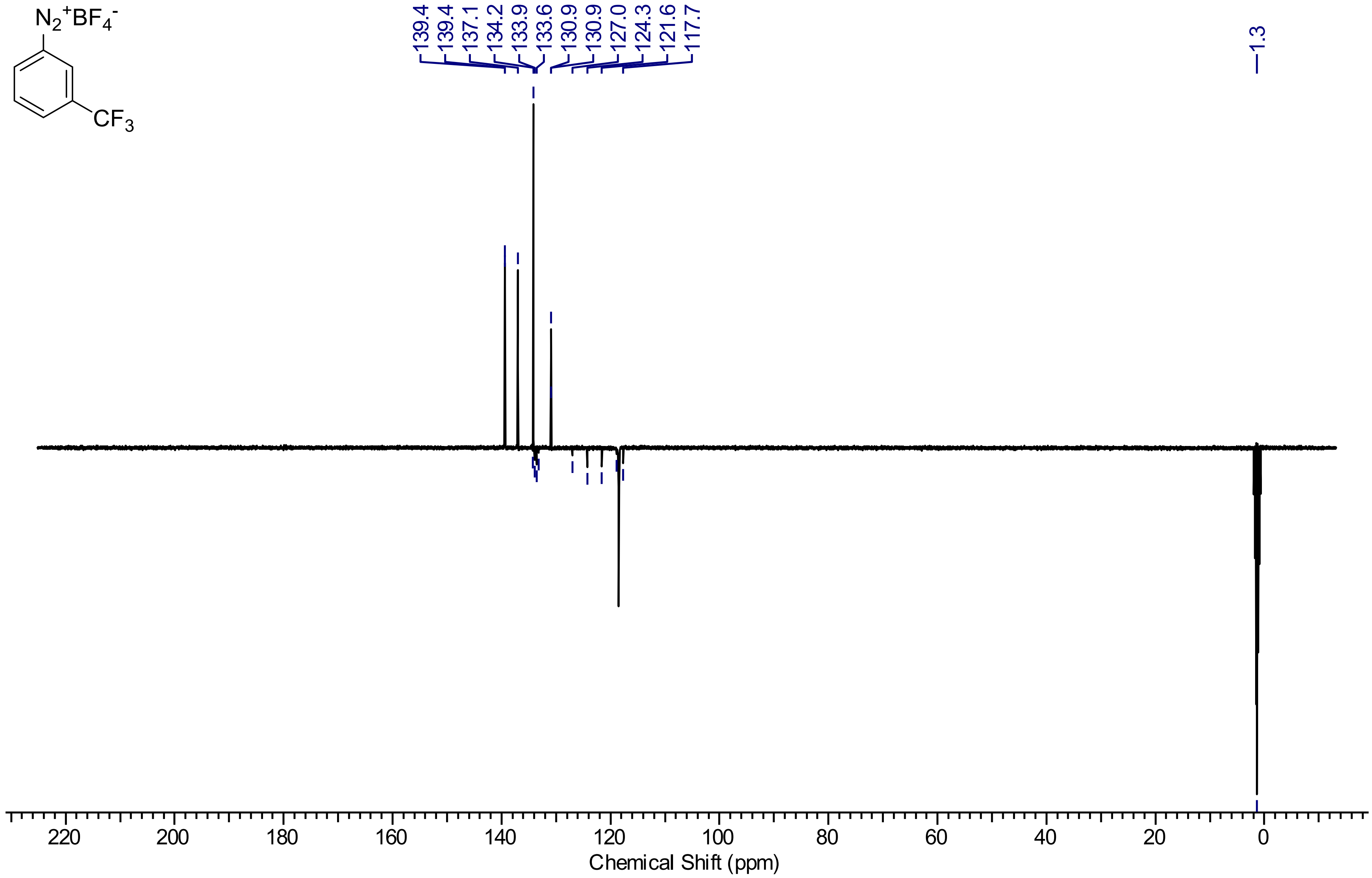

**3c**: $^{1}$H-$^{1}$H COSY NMR

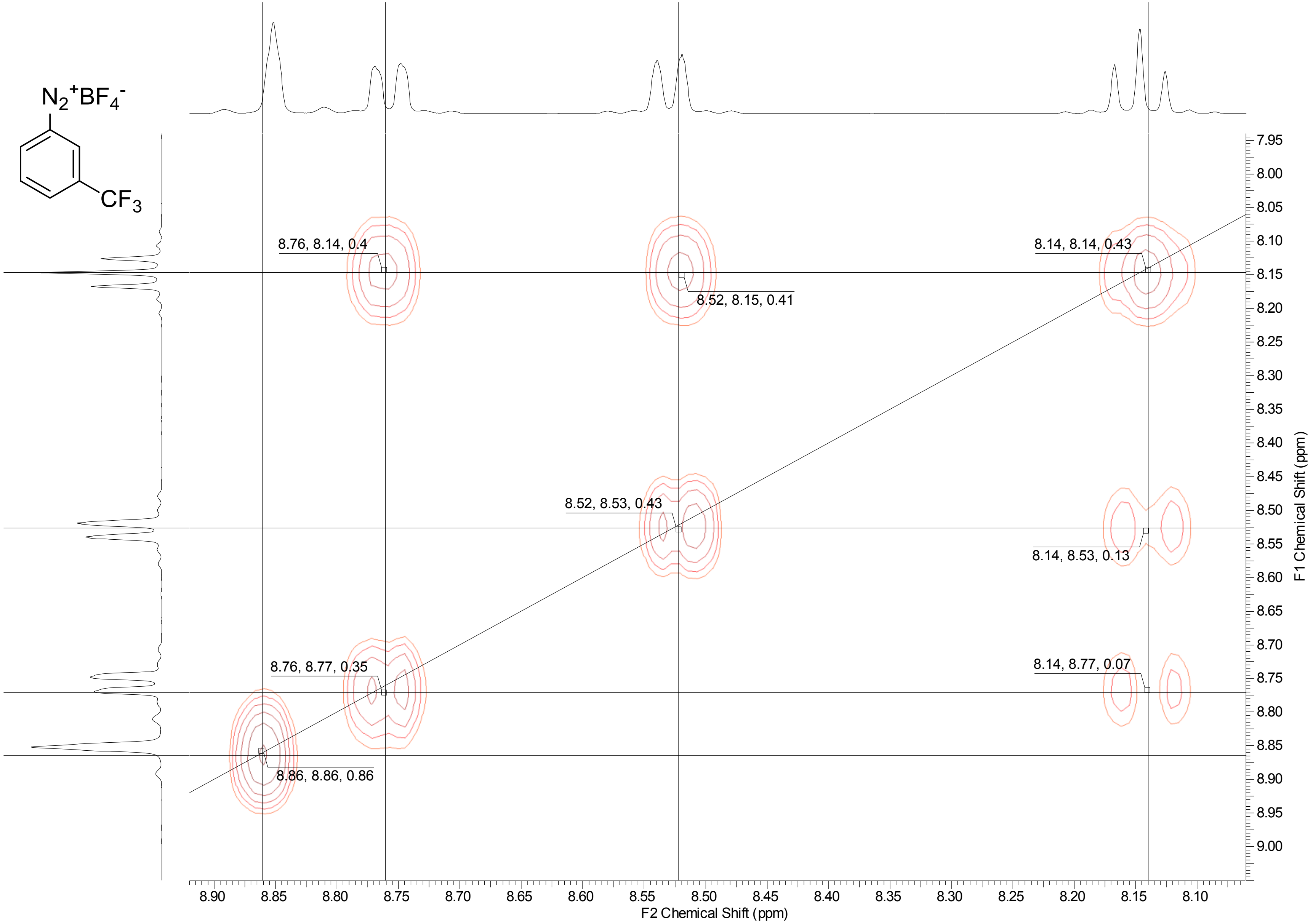

**3c**: $^{19}$F NMR (376 MHz, $CD_3CN$)

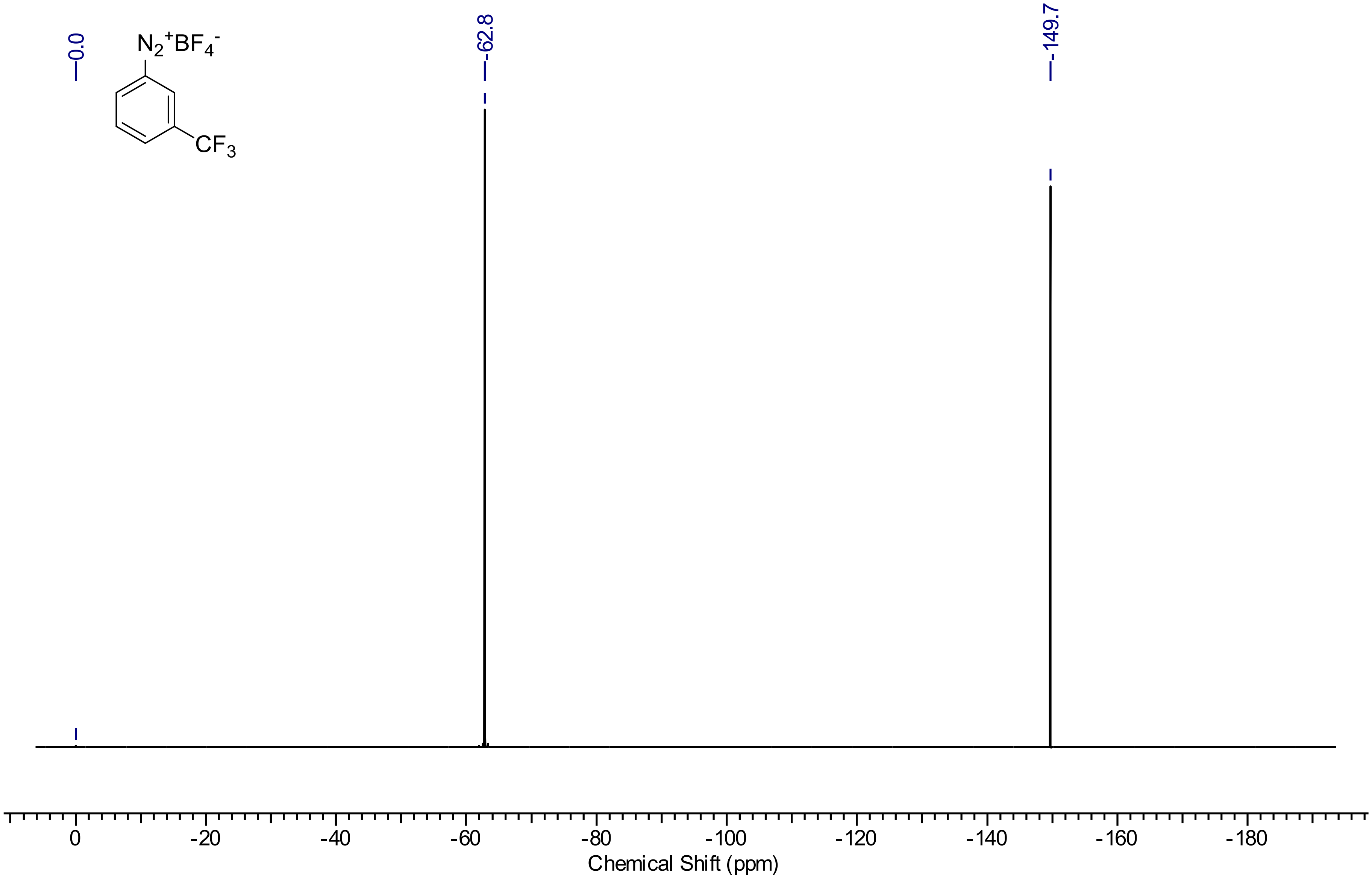

**3d**: $^1$H NMR (400 MHz, $CD_3CN$)

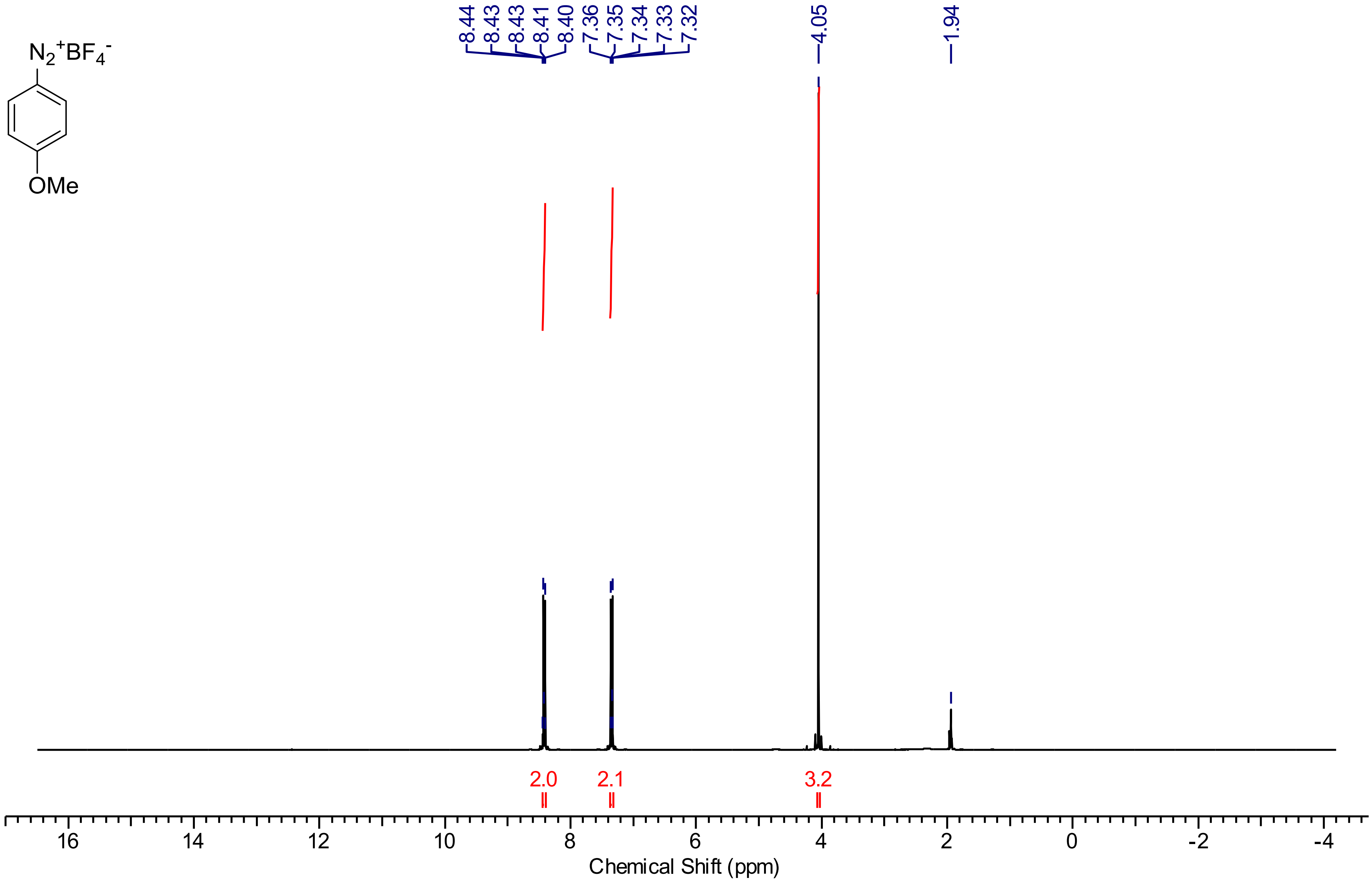

**3d**: $^{13}C$ NMR (101 MHz, $CD_3CN$)

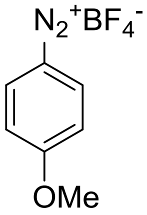

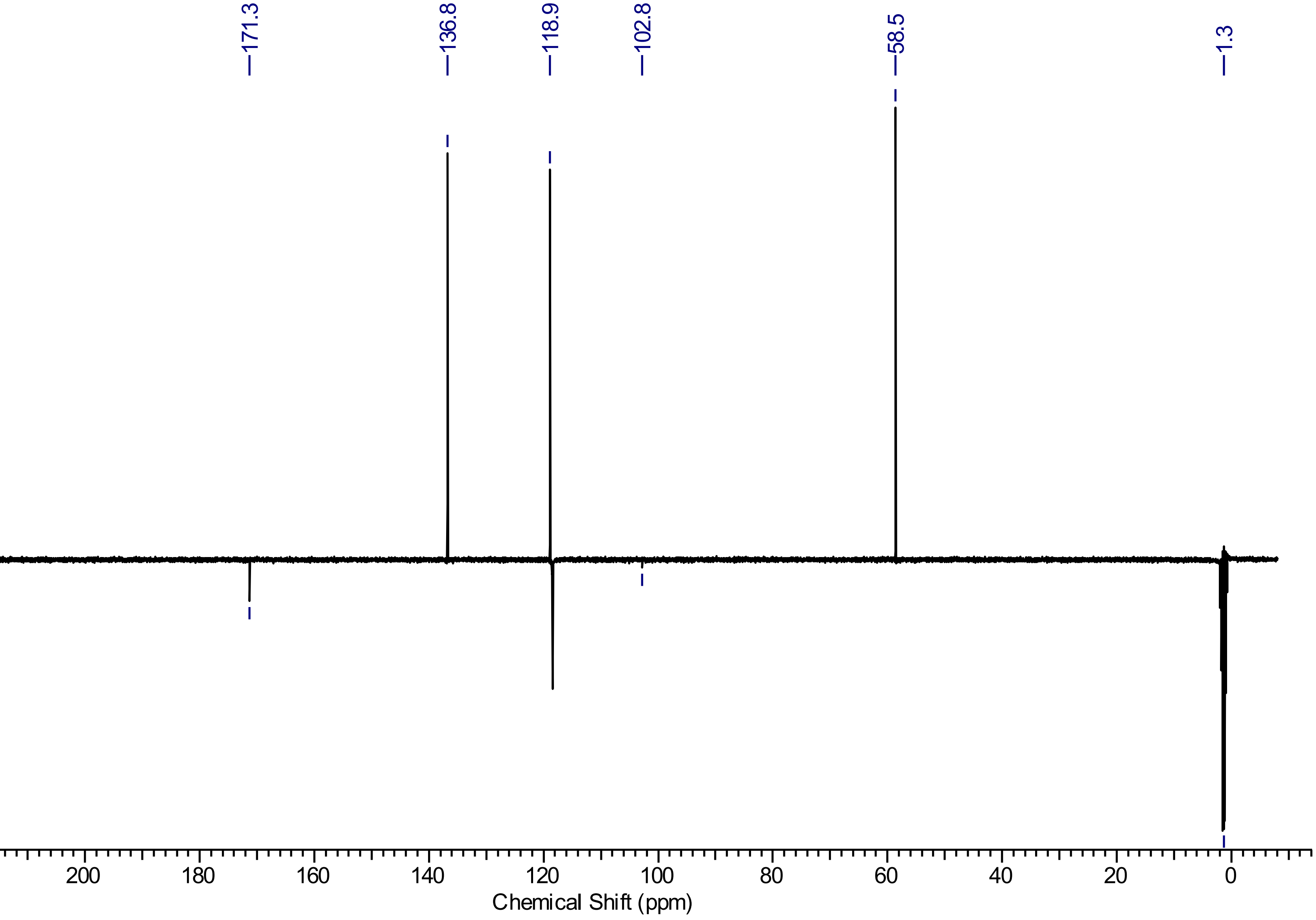

**3d**: $^{19}F$ NMR (376 MHz, [$D_6$]acetone)

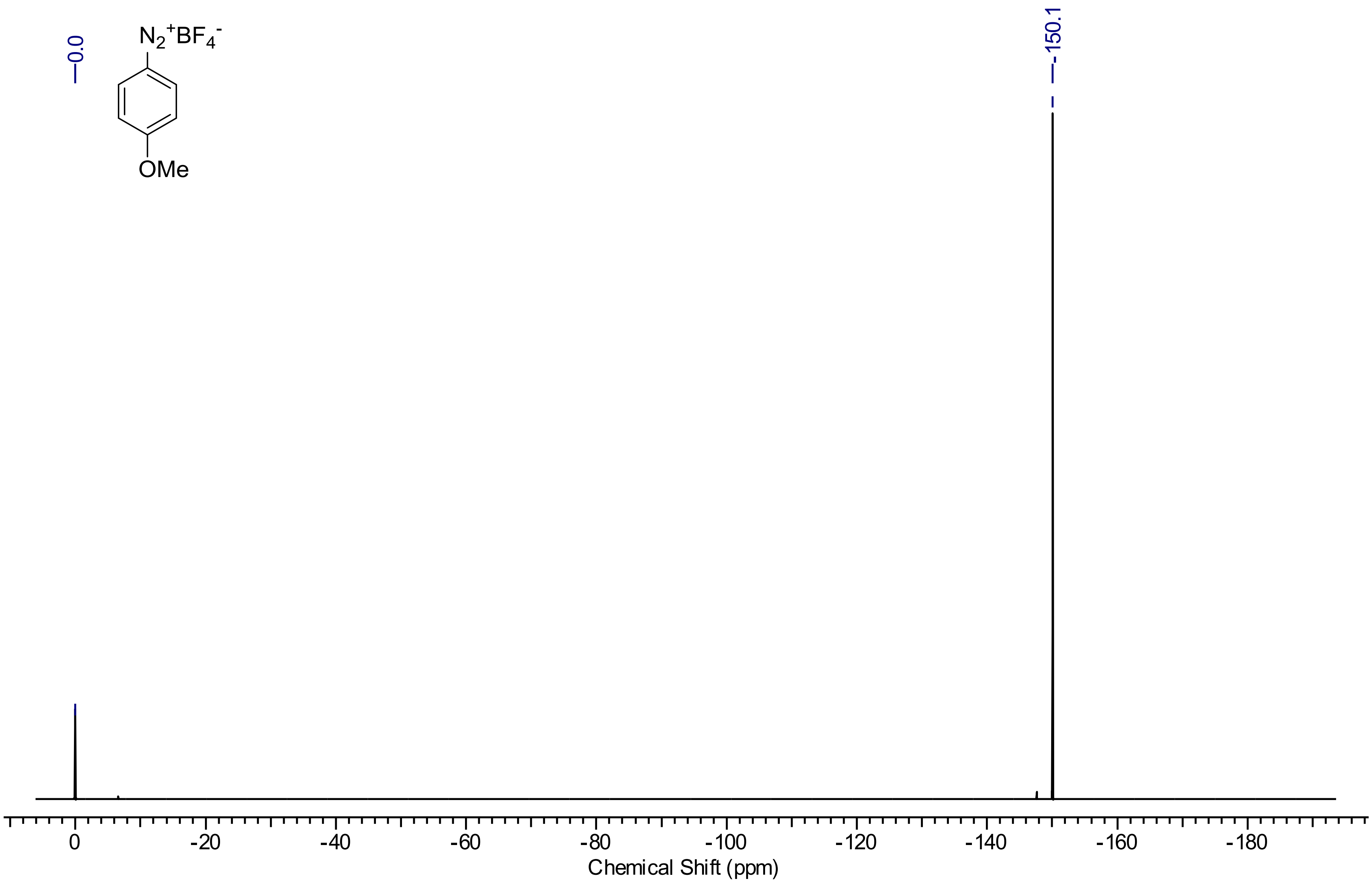

**3e**: $^{1}$H NMR (400 MHz, $CD_3CN$)

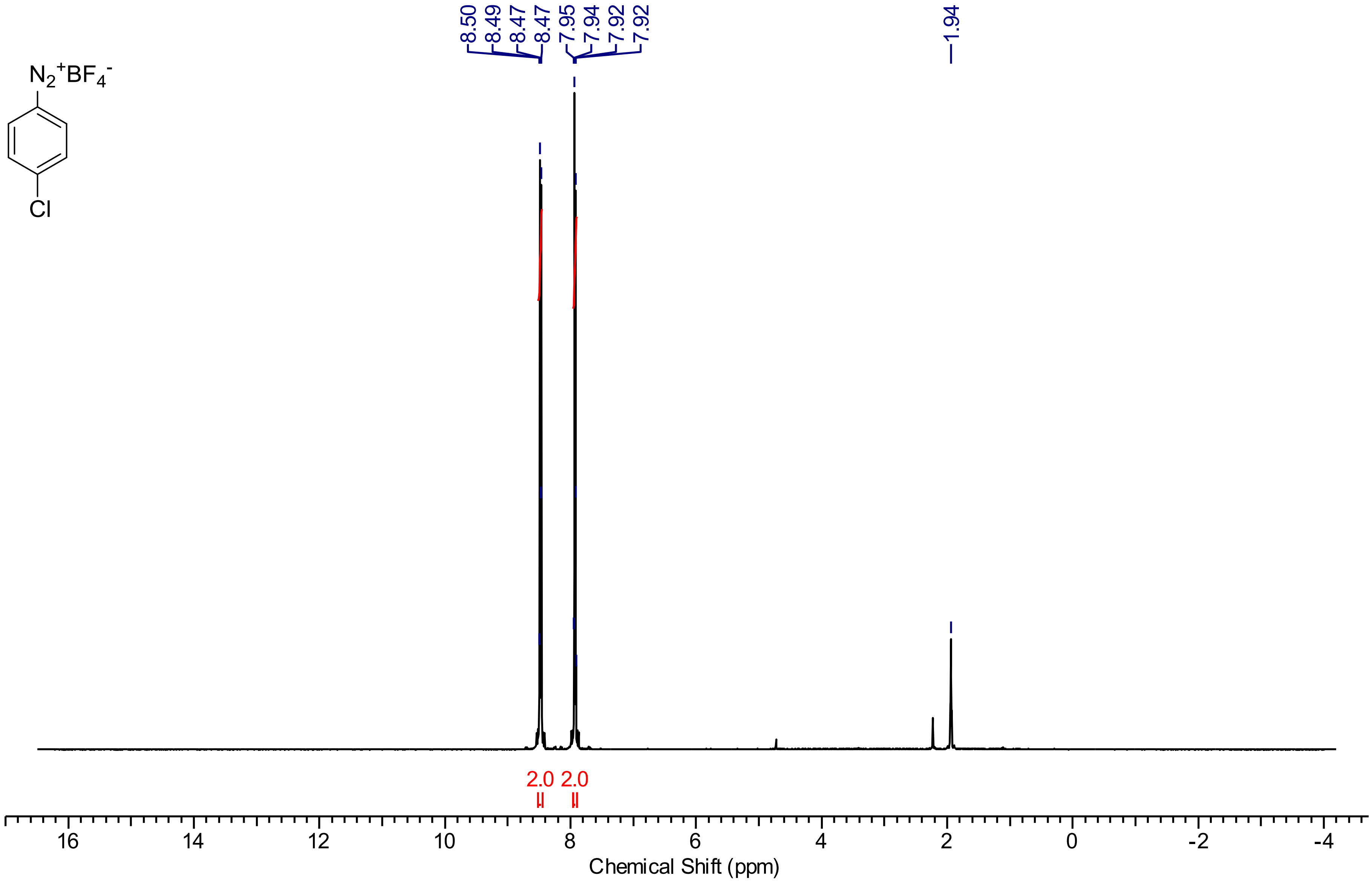

**3e**: $^{13}$C NMR (101 MHz, $CD_3CN$)

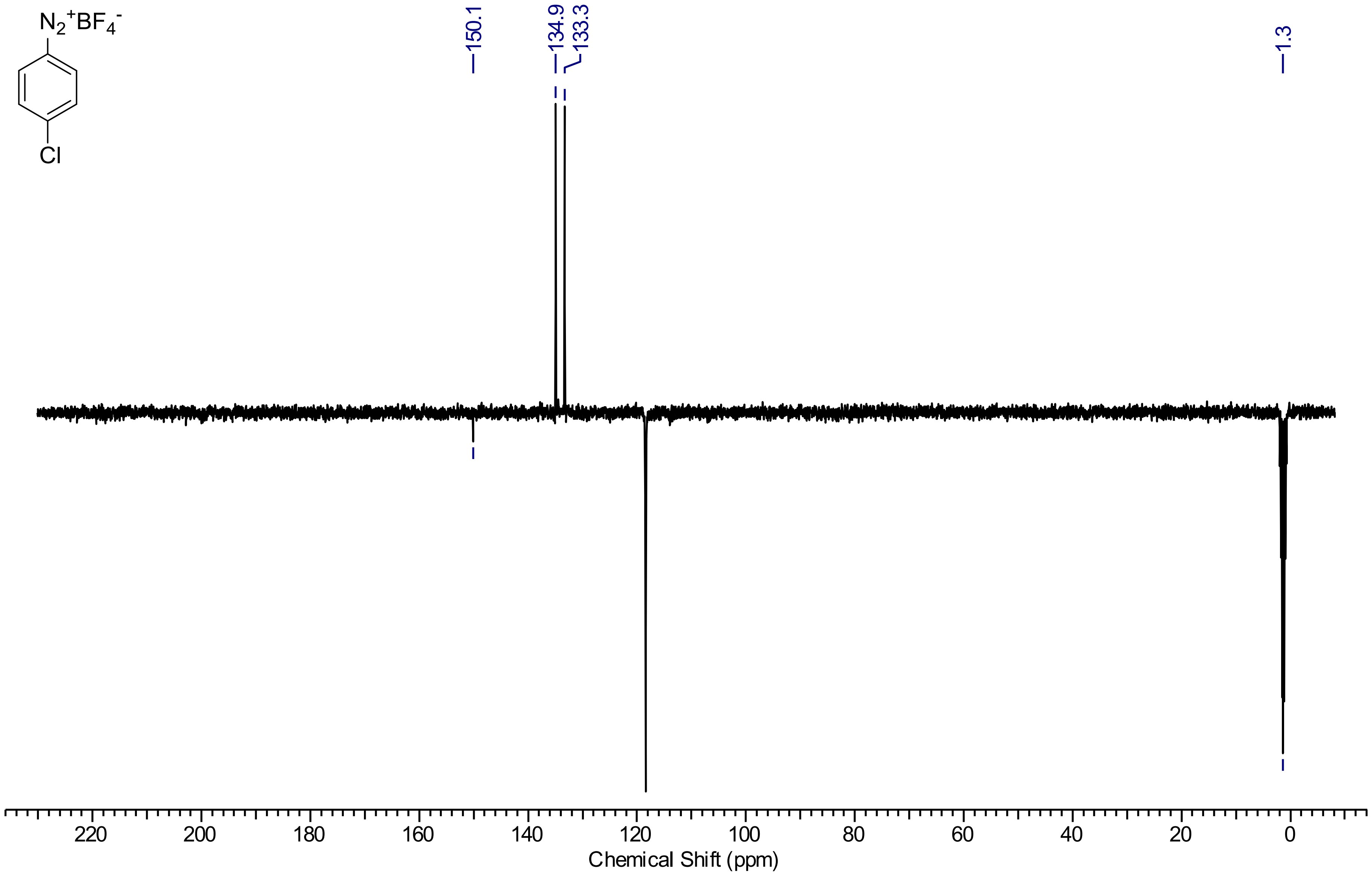

**3e**: $^{19}$F NMR (376 MHz, $CD_3CN$)

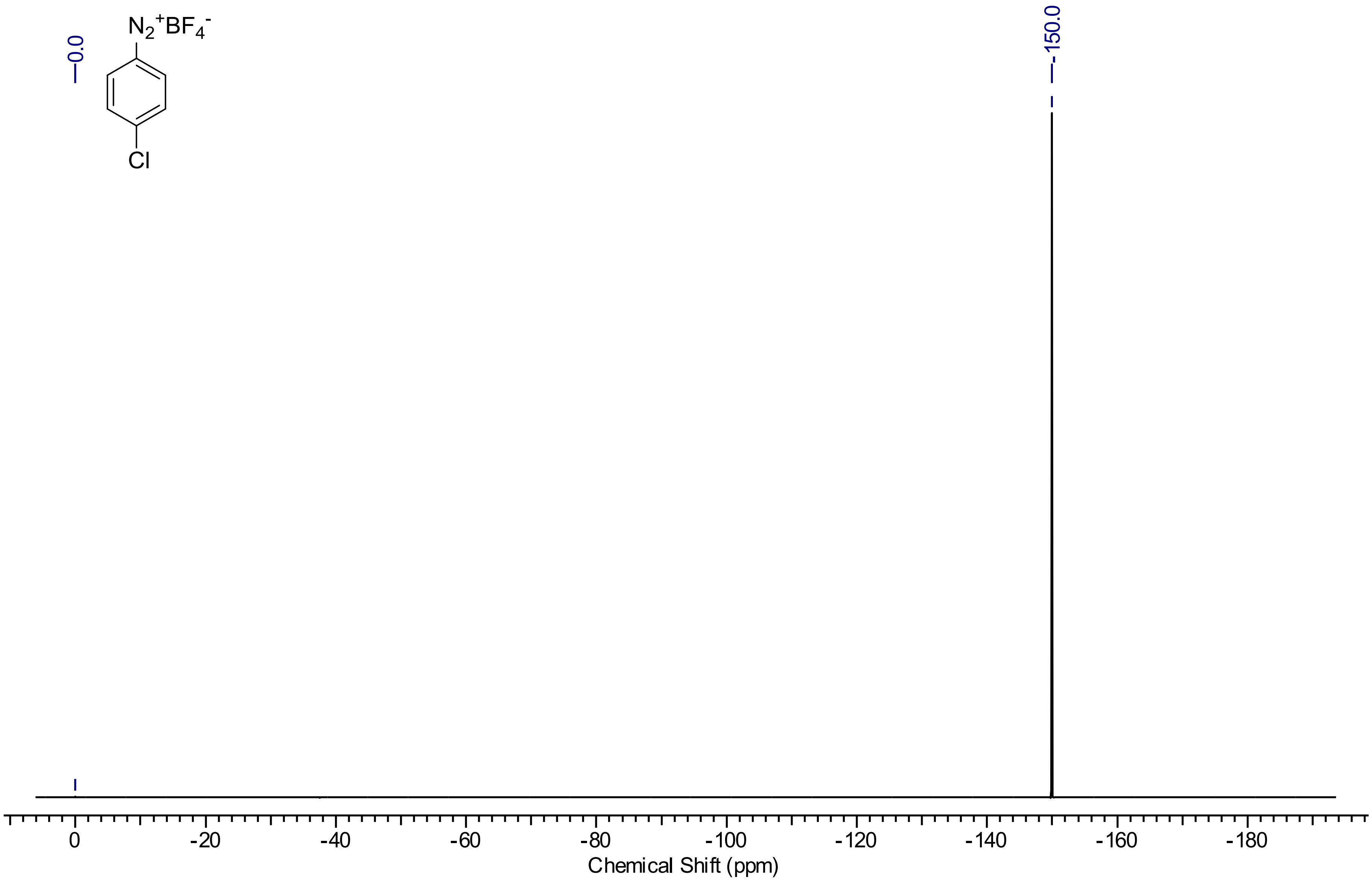

**3f**: $^1$H NMR (400 MHz, $CD_3CN$)

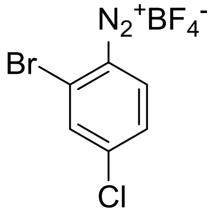


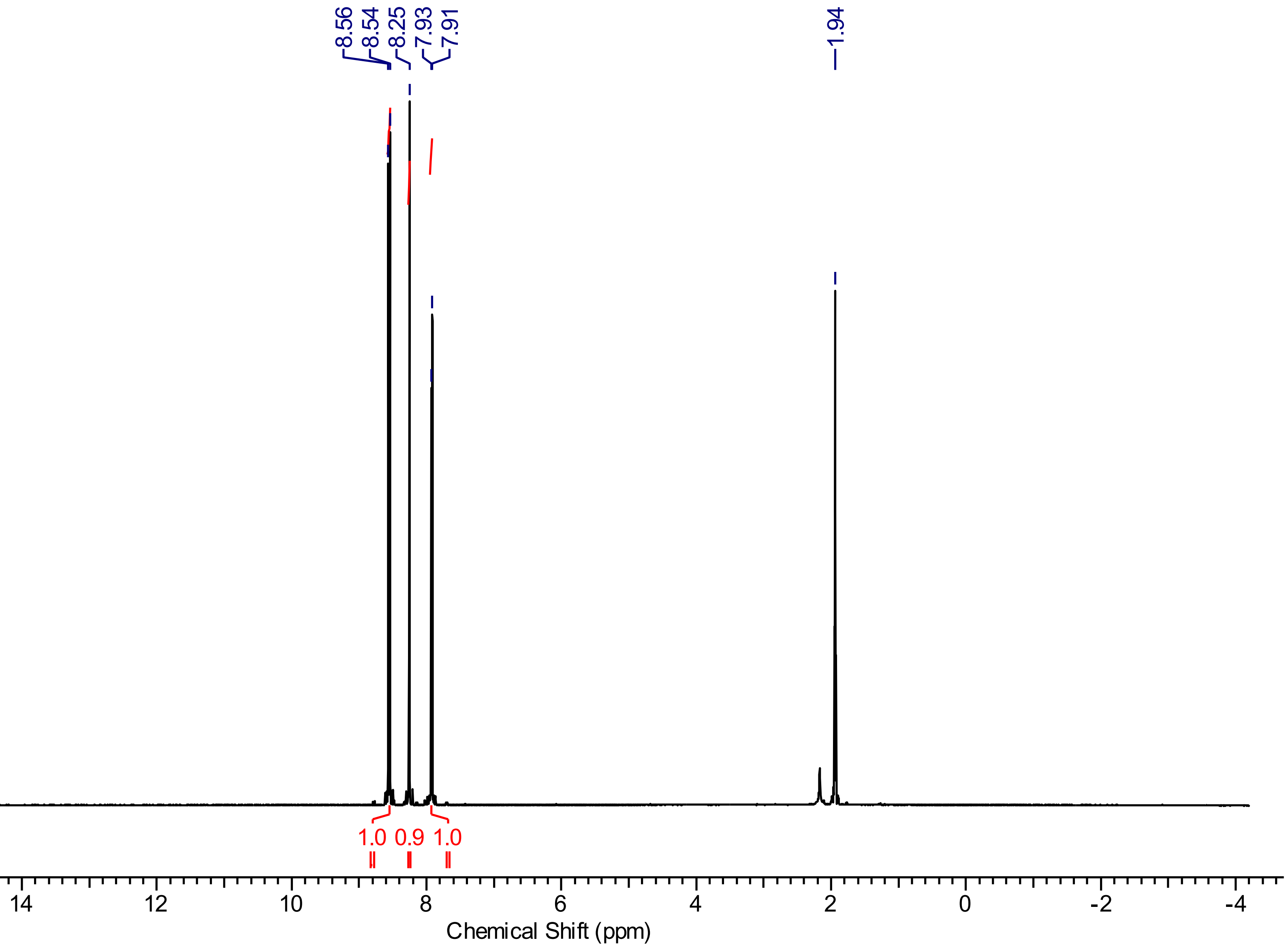

**3f**: $^{13}$C NMR (101 MHz, $CD_3CN$)

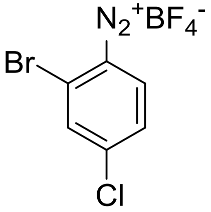


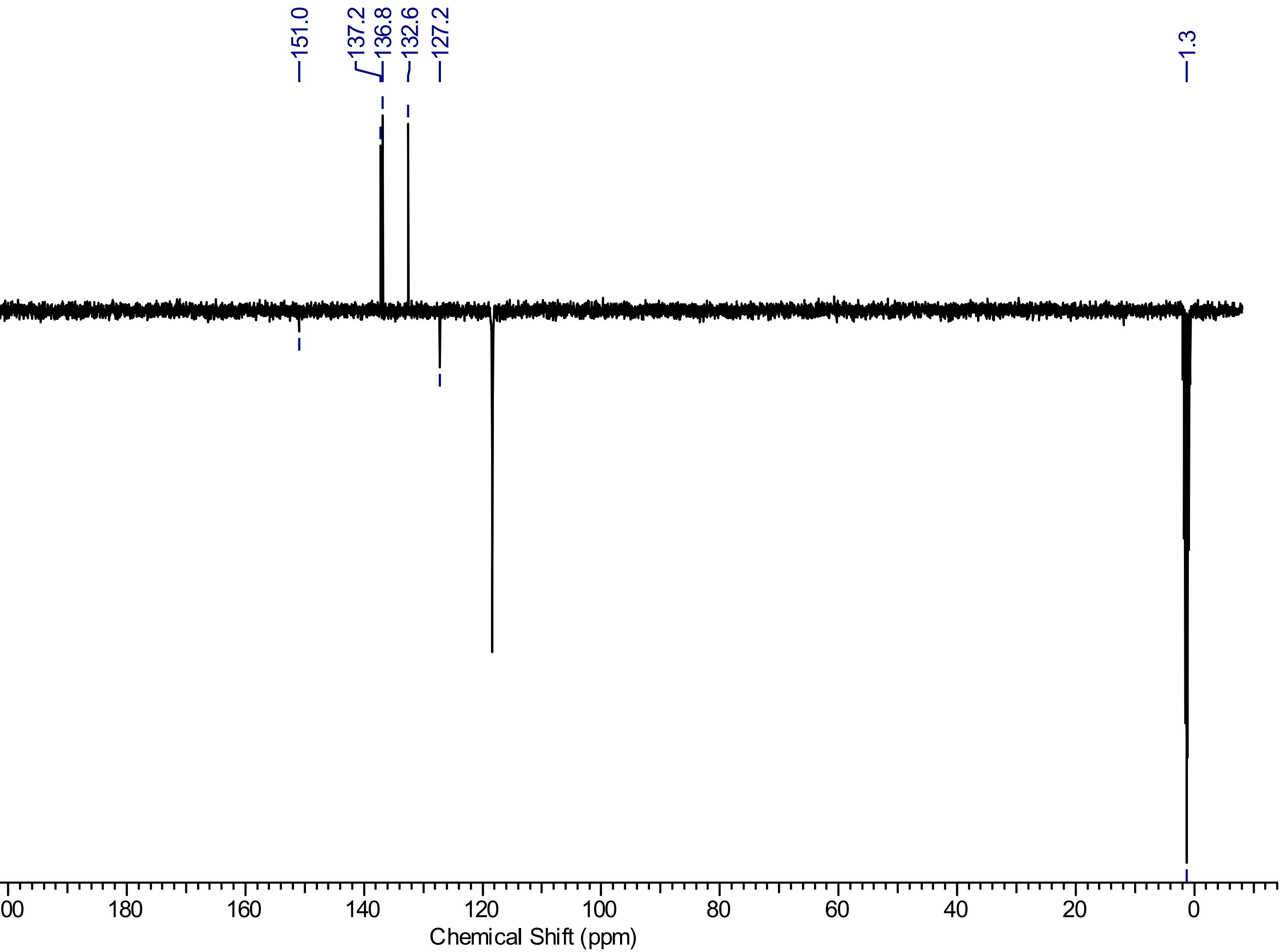

**3f**: $^{19}$F NMR (376 MHz, $CD_3CN$)

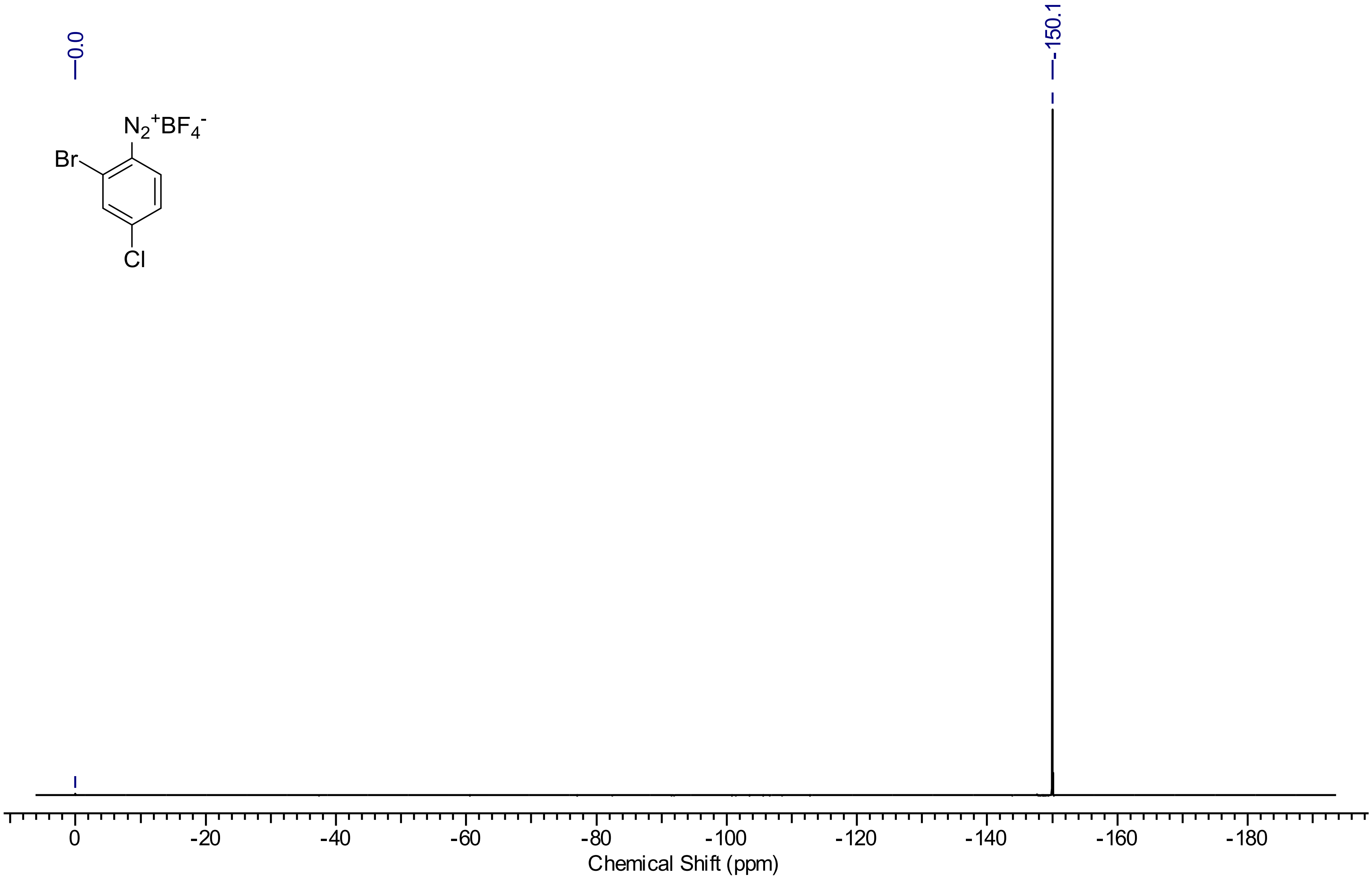

**3g**: $^{1}$H NMR (400 MHz, $CD_3CN$)

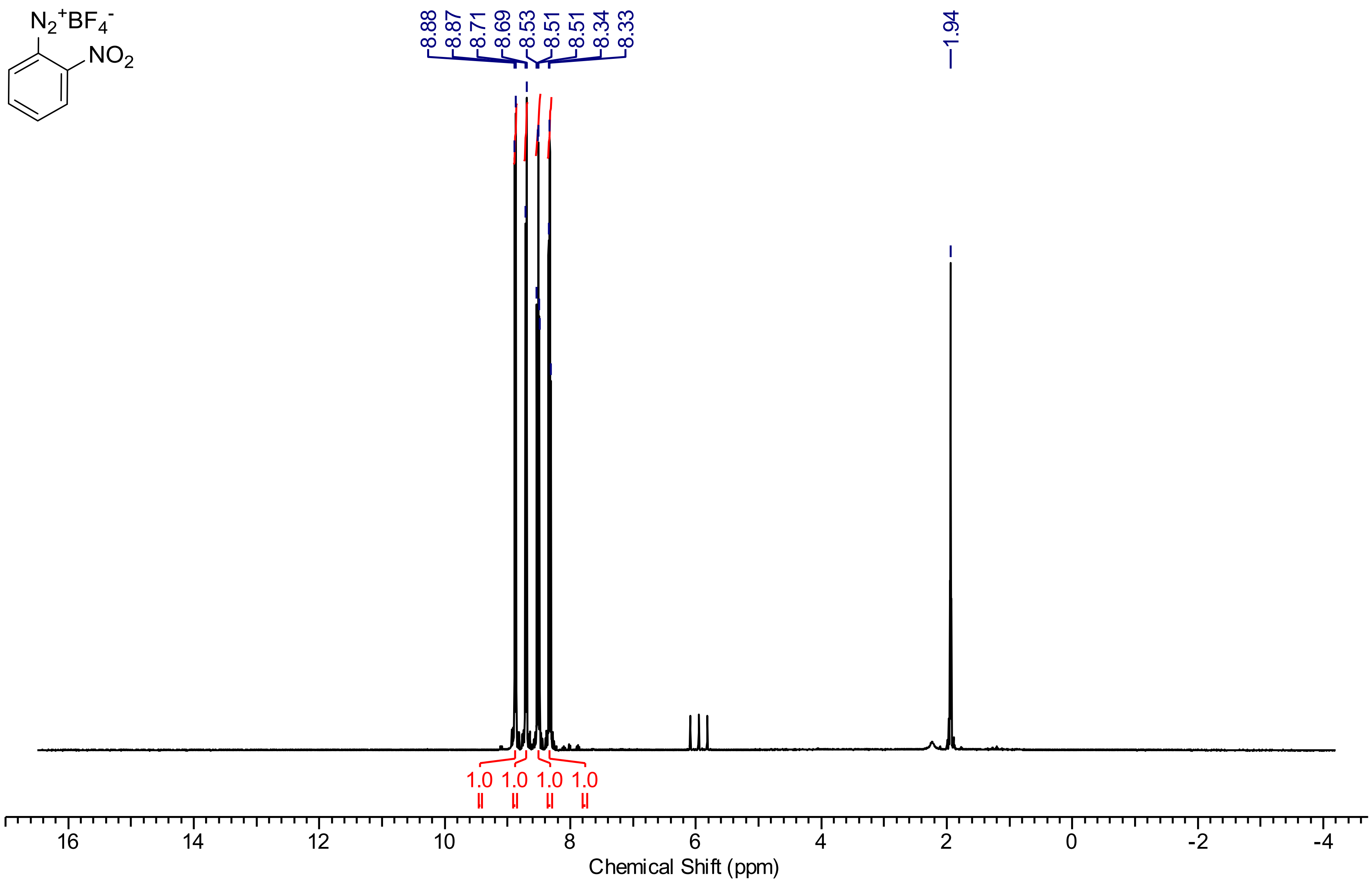

**3g**: $^{13}C$ NMR (101 MHz, $CD_3CN$)

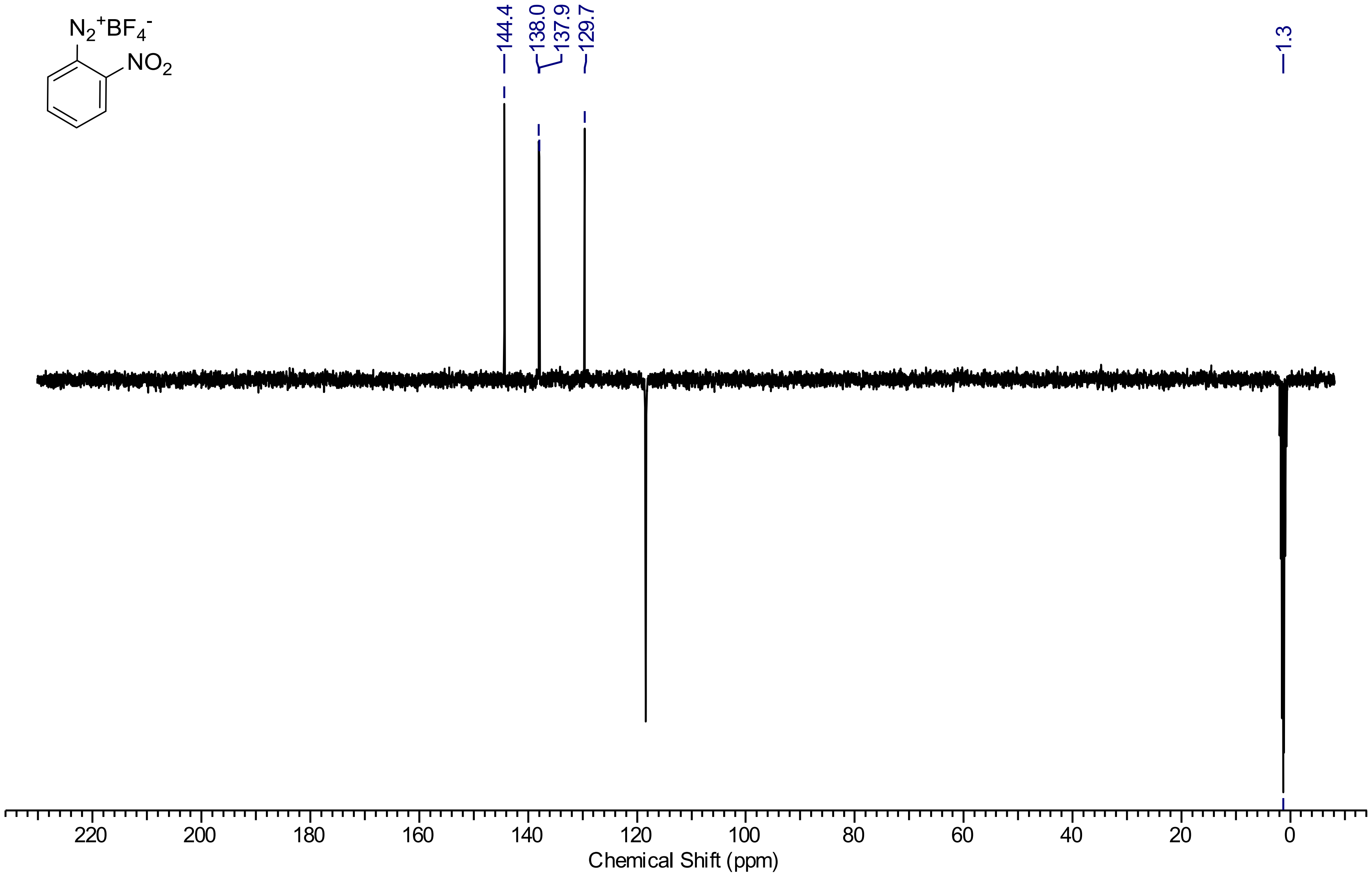

**3g**: $^{19}$F NMR (376 MHz, $CD_3CN$)

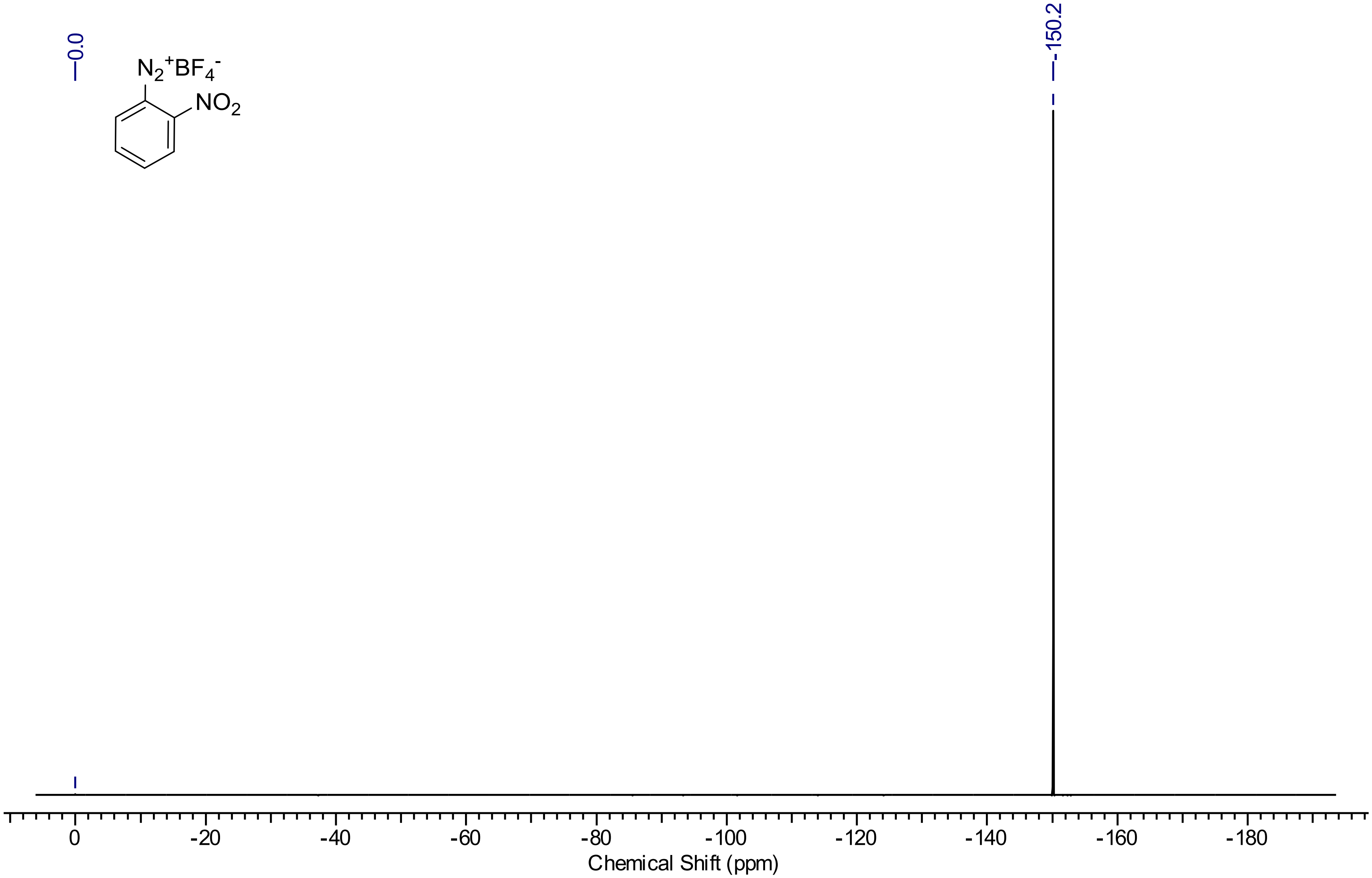

**3h**: $^{1}H$ NMR (400 MHz, $CD_3CN$)

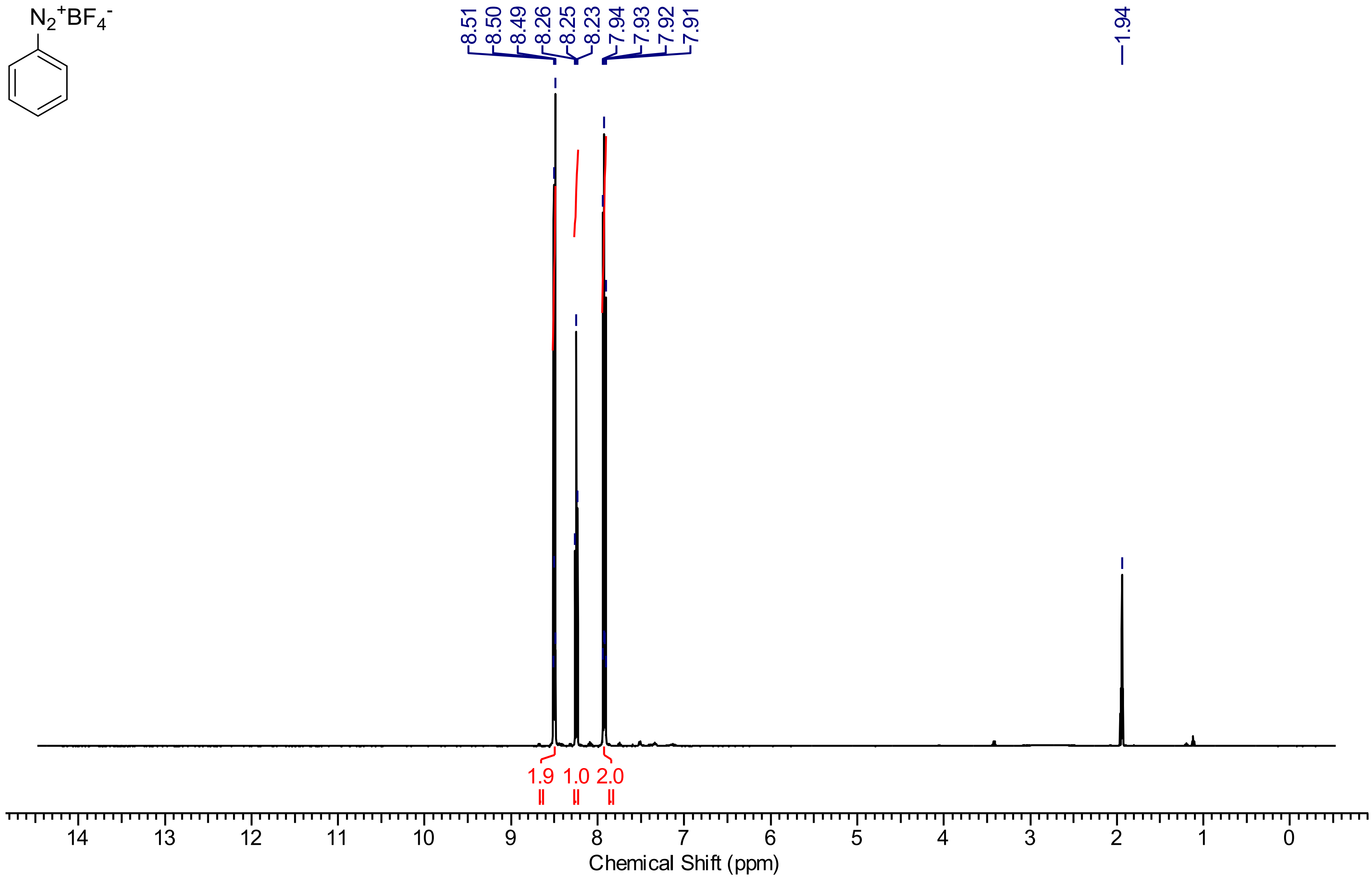

**3h**: $^{13}C$ NMR (126 MHz, $CD_3CN$)

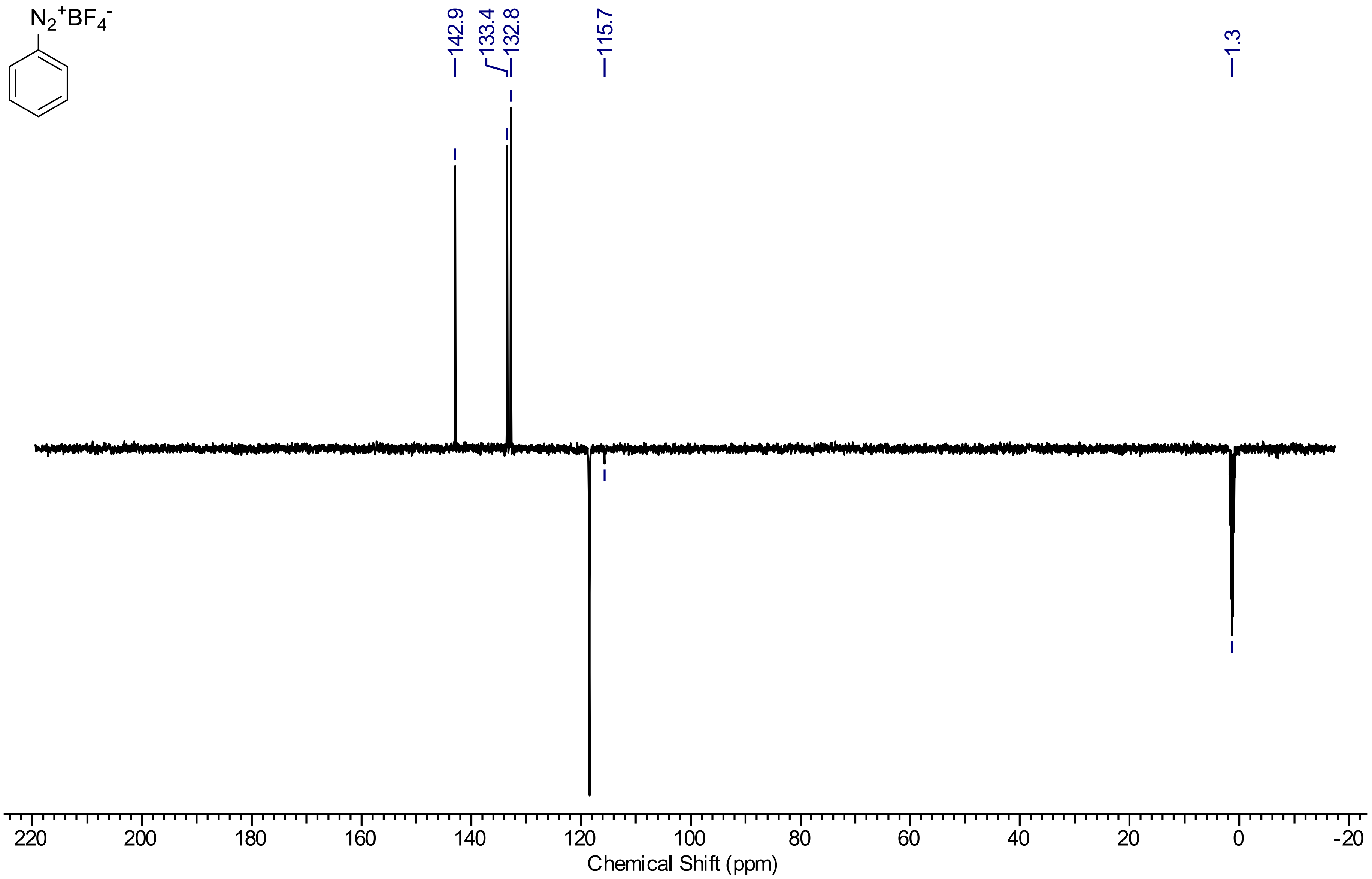

**3h**: $^{19}F$ NMR (470 MHz, $CD_3CN$)

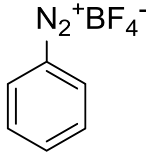

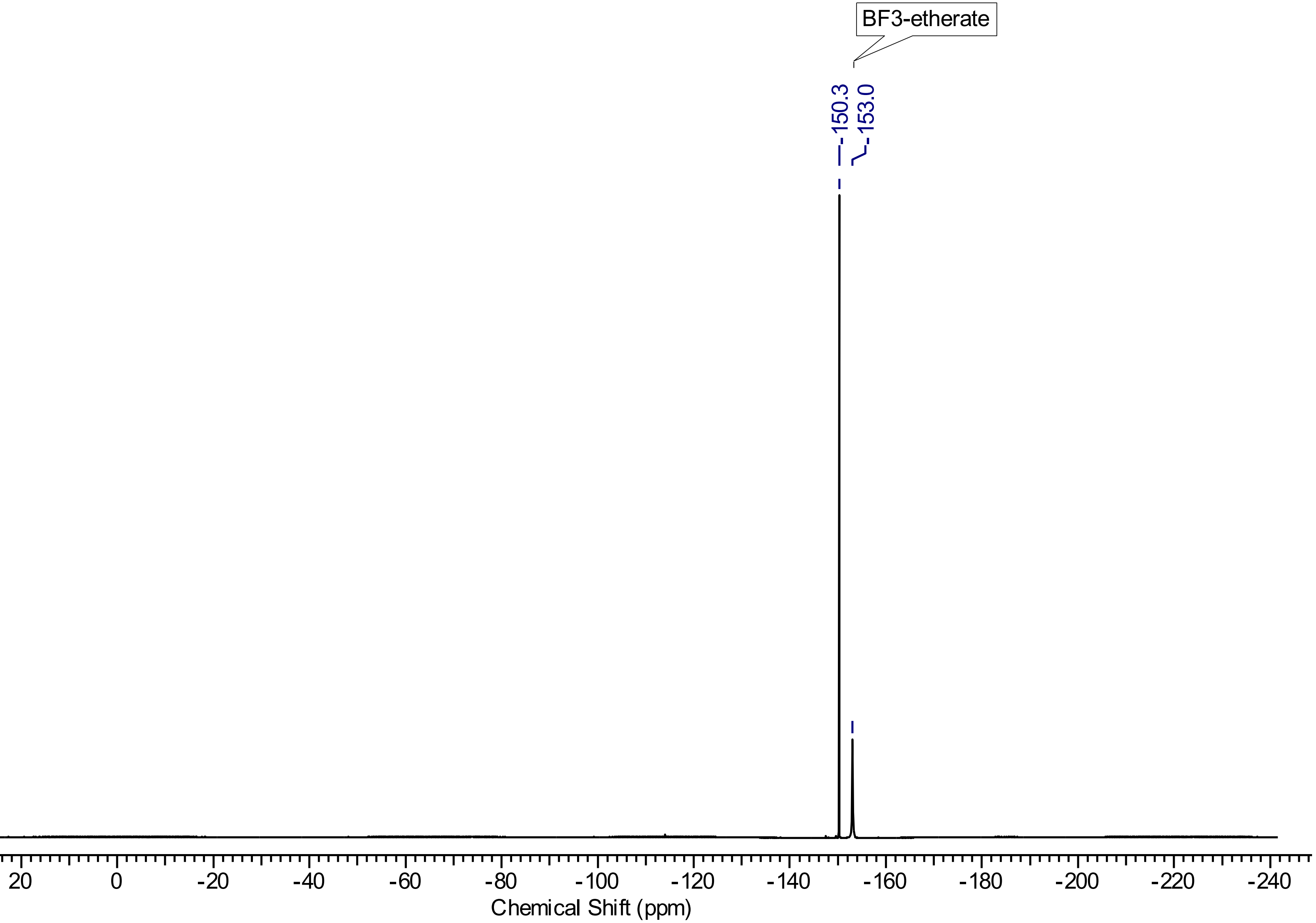

**3i**: $^{1}H$ NMR (500 MHz, $CD_3CN$)

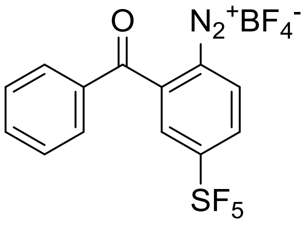


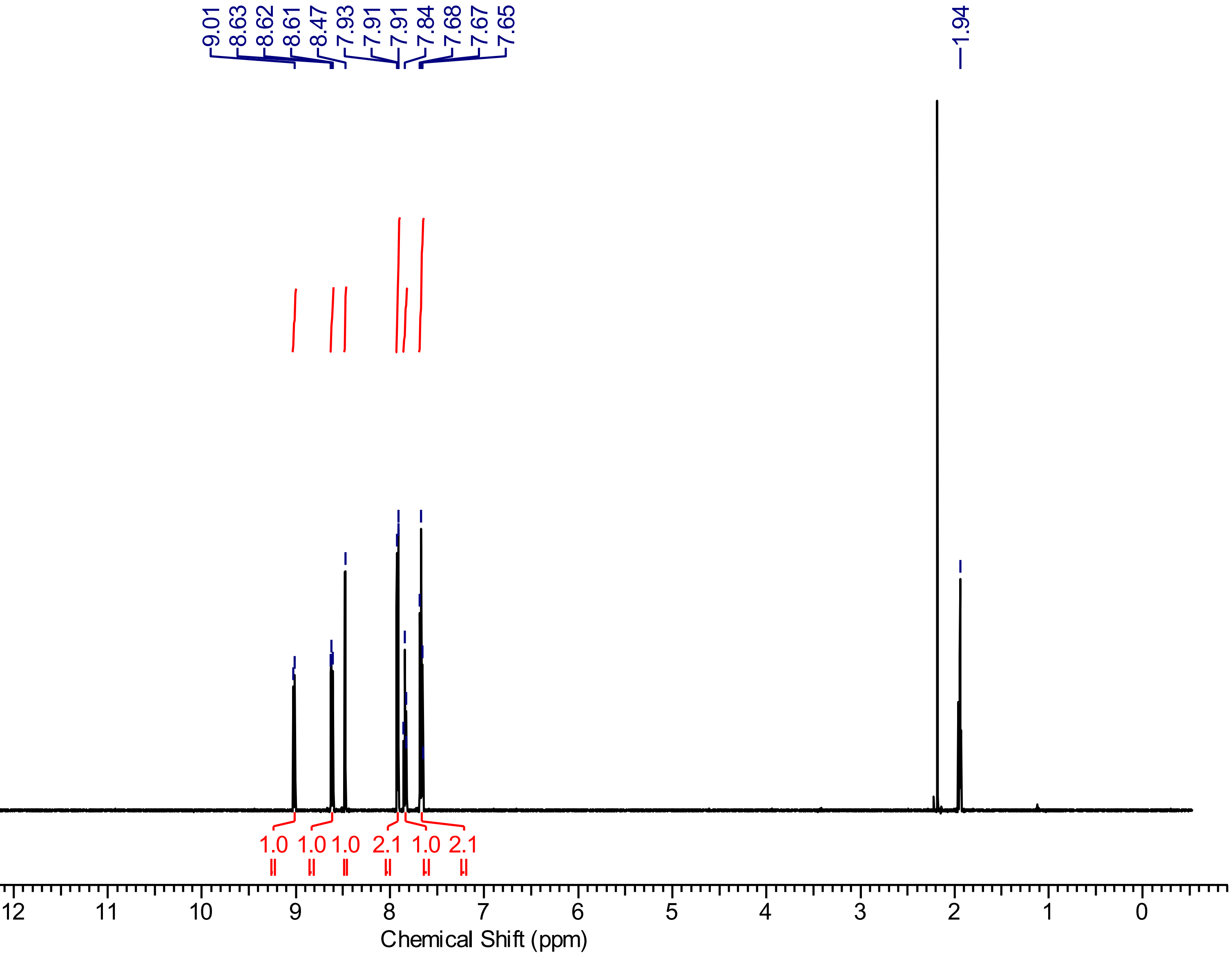

**3i**: $^{11}$B NMR (160 MHz, $CD_3CN$)

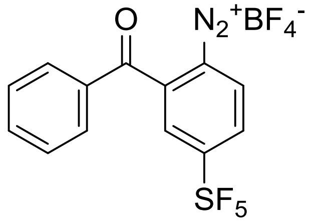


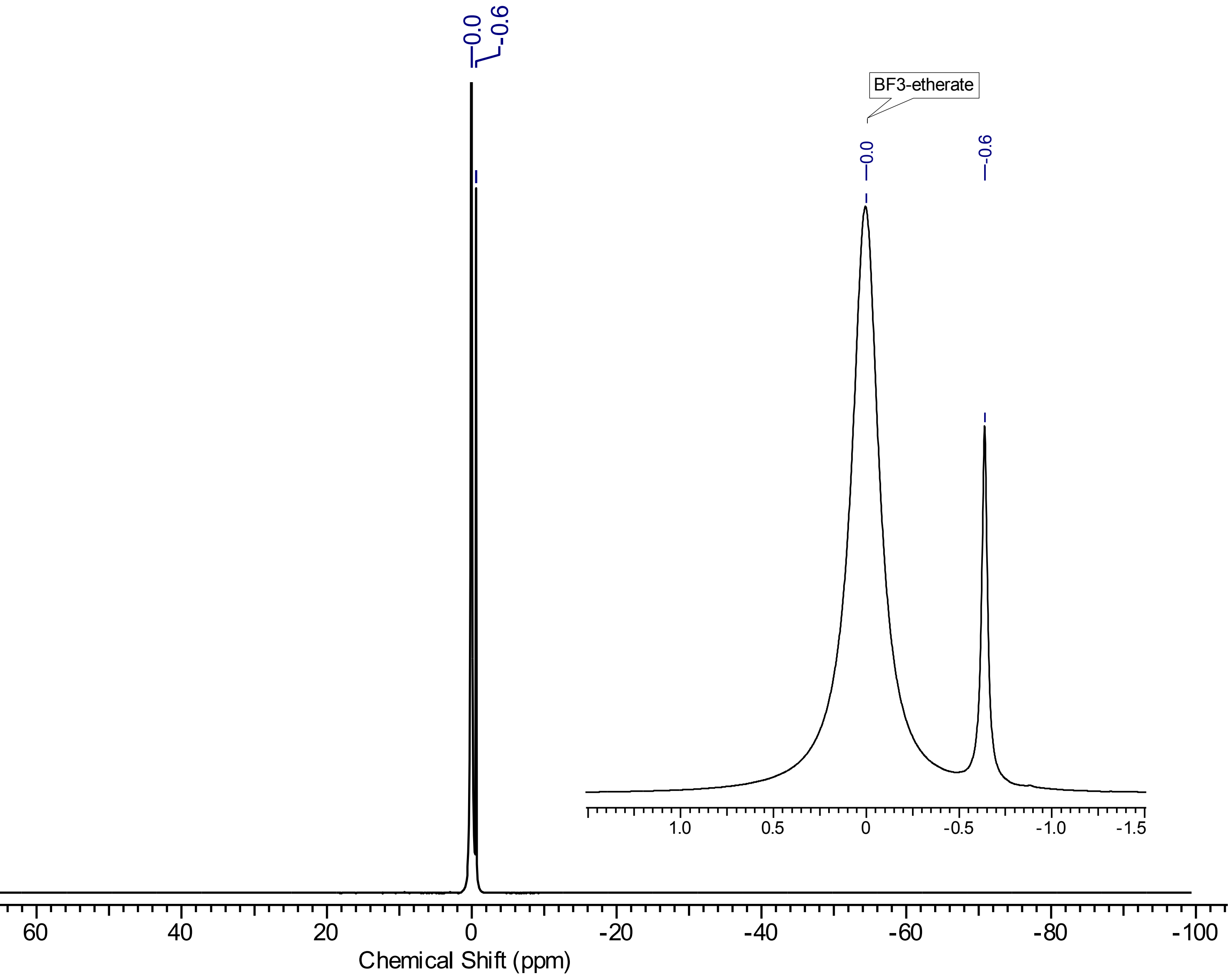

**3i**: $^{13}$C NMR (126 MHz, $CD_3CN$)

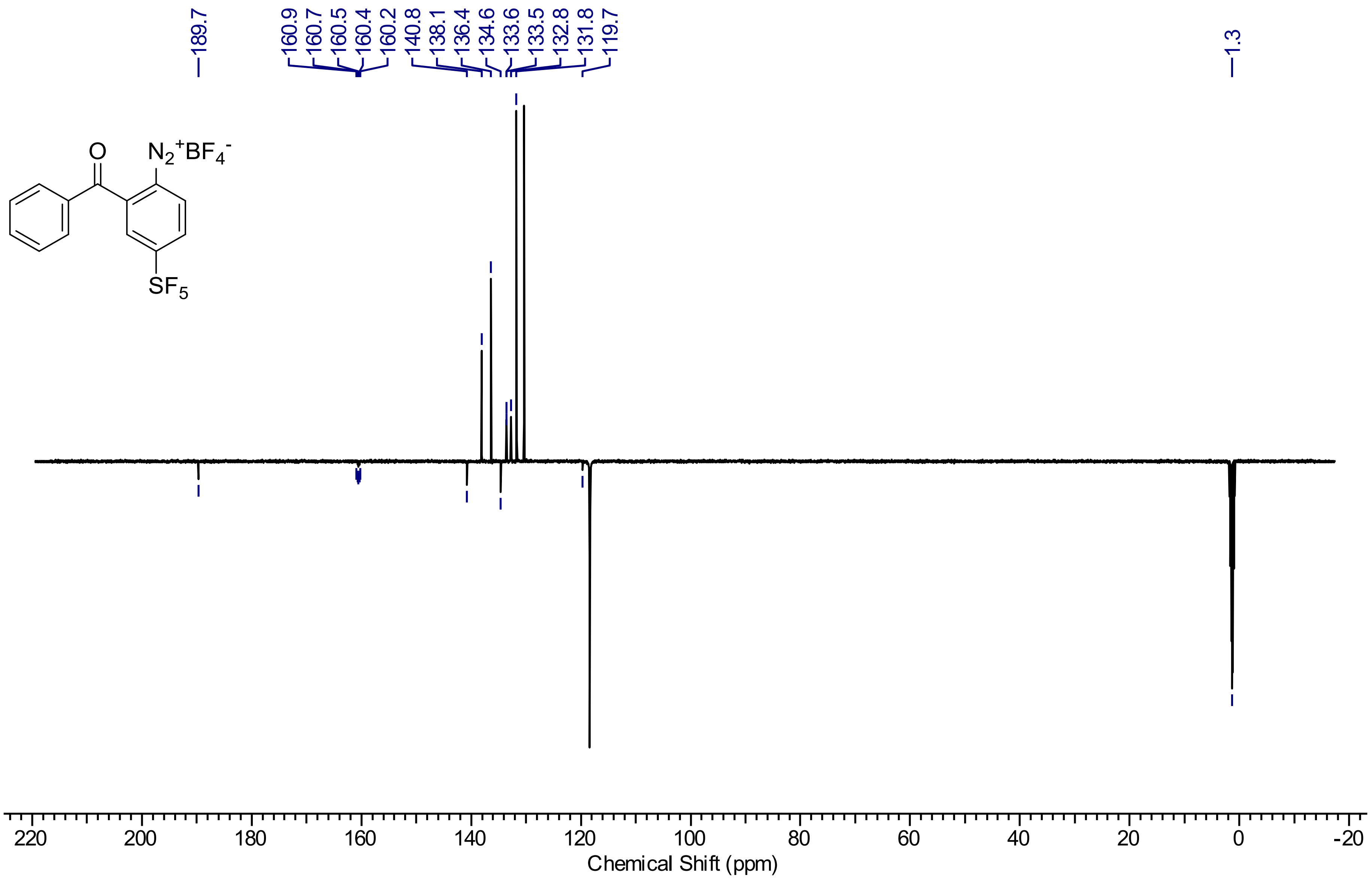

**3i**: $^{19}F$ NMR (377 MHz, $CD_3CN$)

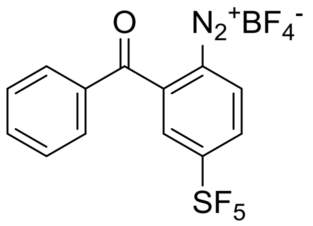

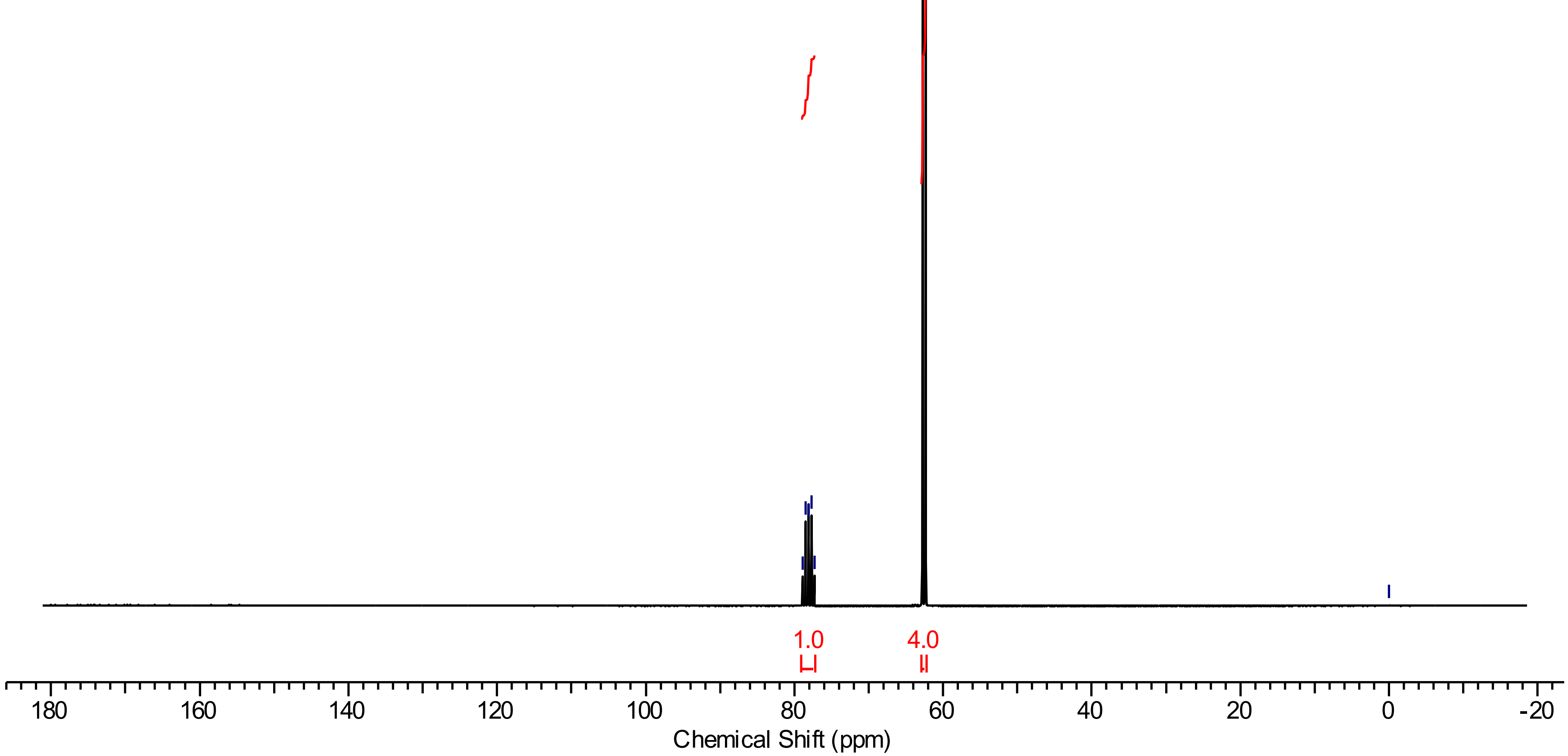

**3i**: $^{19}$F NMR (377 MHz, $CD_3CN$)

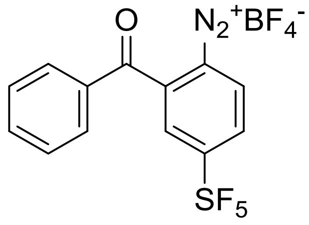


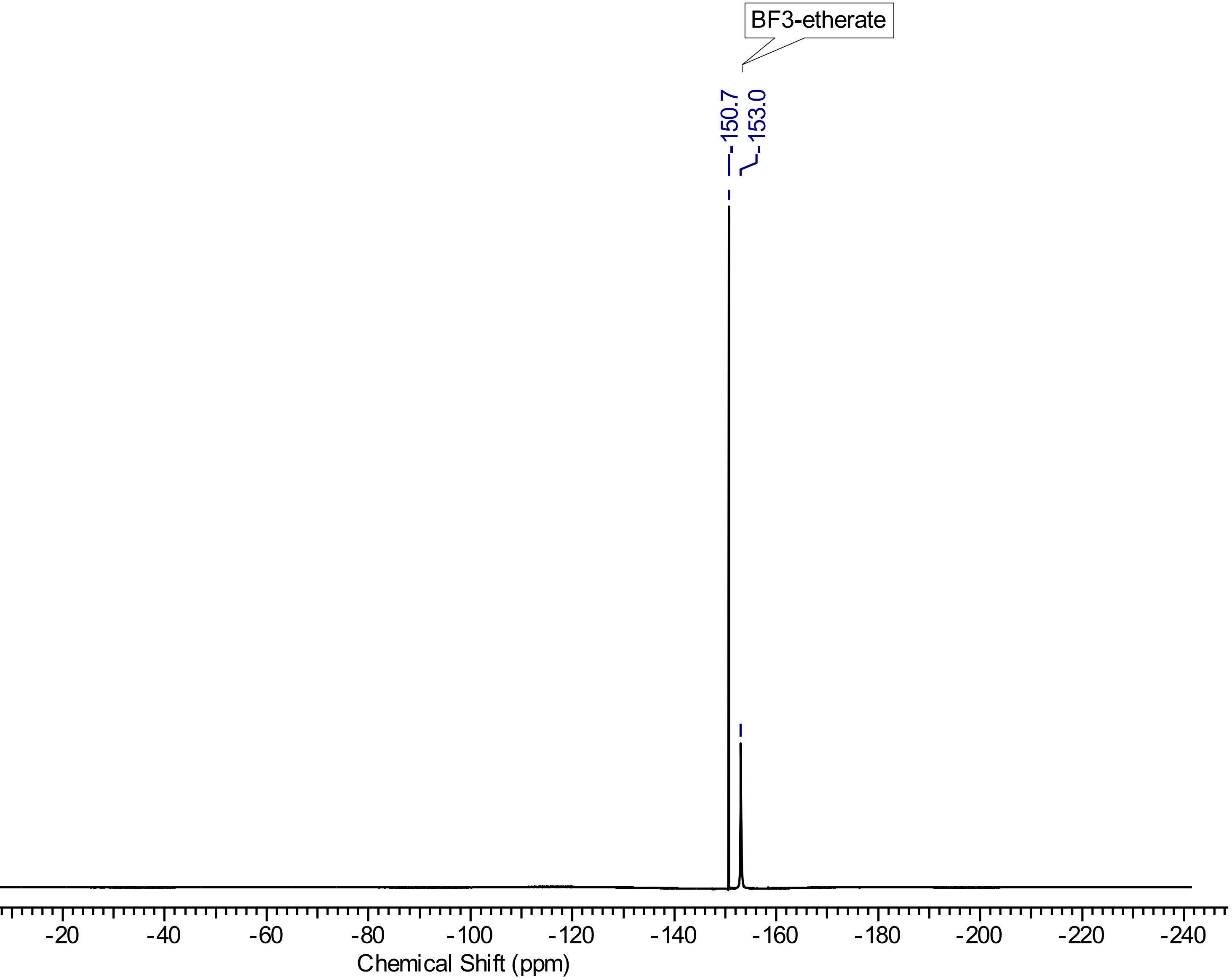

**pyridine-BF$_3$ complex**: $^{11}$B NMR (128 MHz, $CD_3CN$)

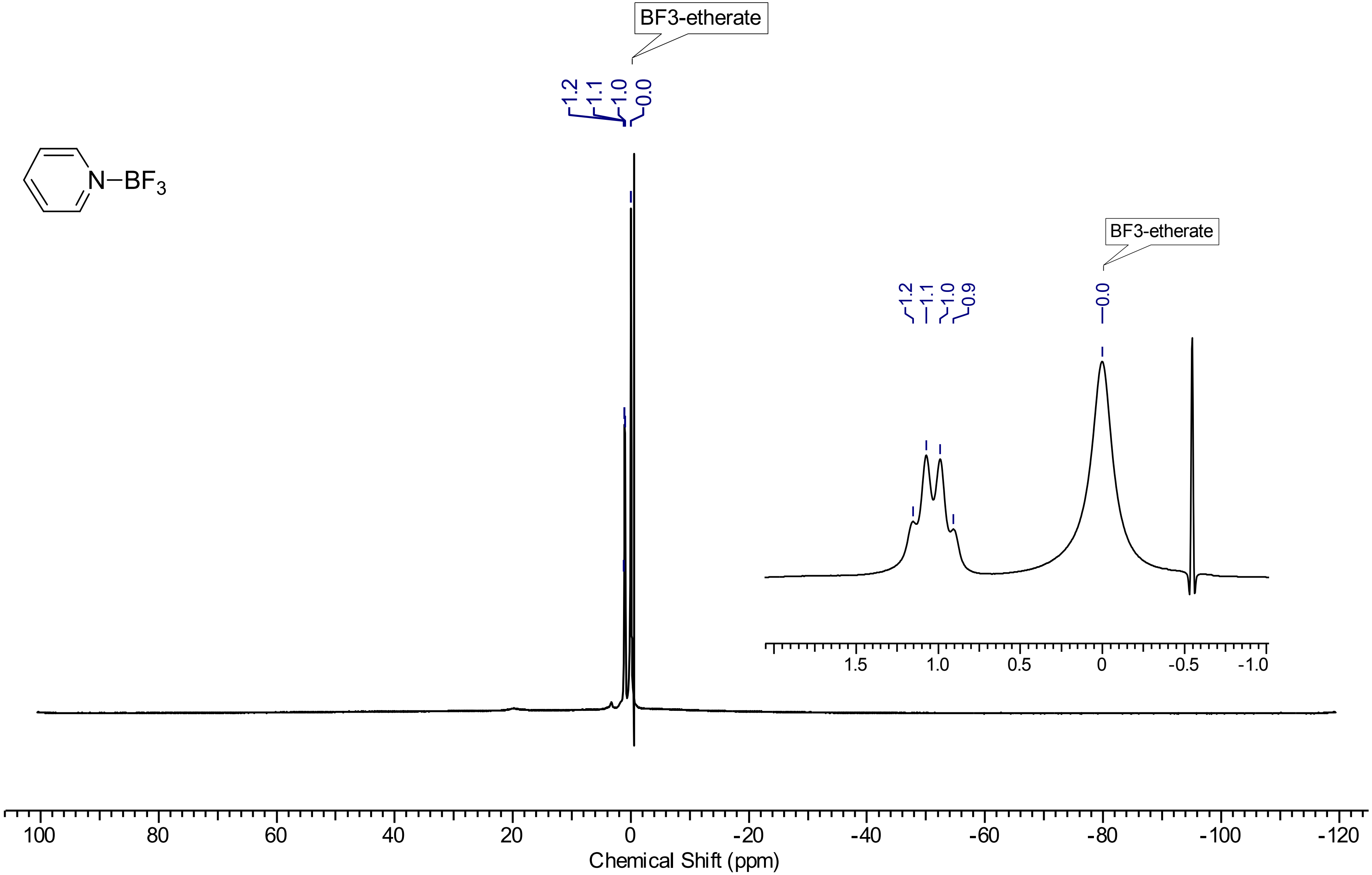

**pyridine-$BF_3$ complex**: $^{19}F$ NMR **(**376 MHz, $CD_3CN$**)**

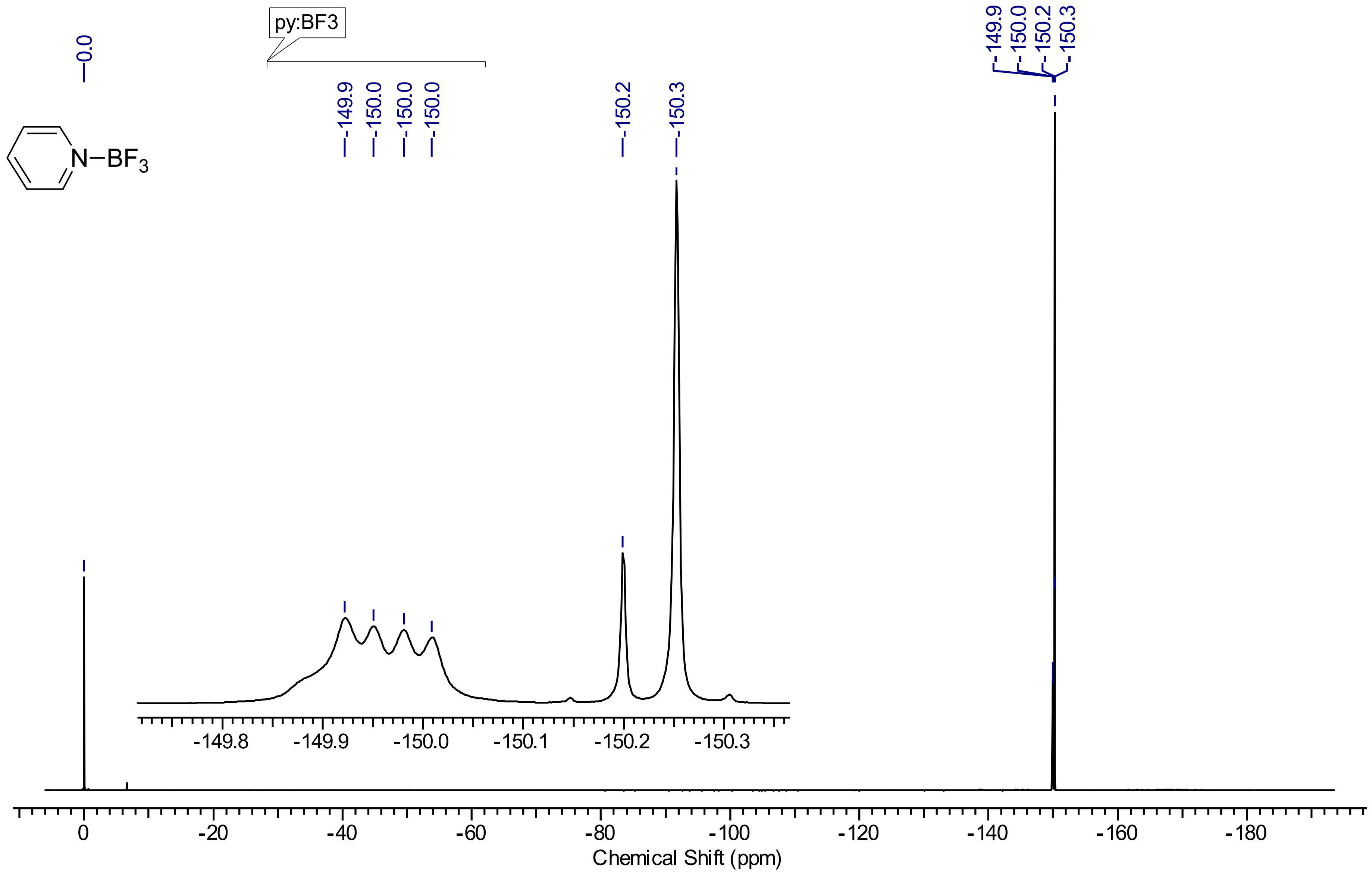

**F-Bpin**: $^{11}$B NMR (128 MHz, $CD_3CN$)

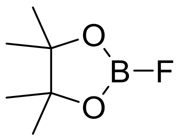


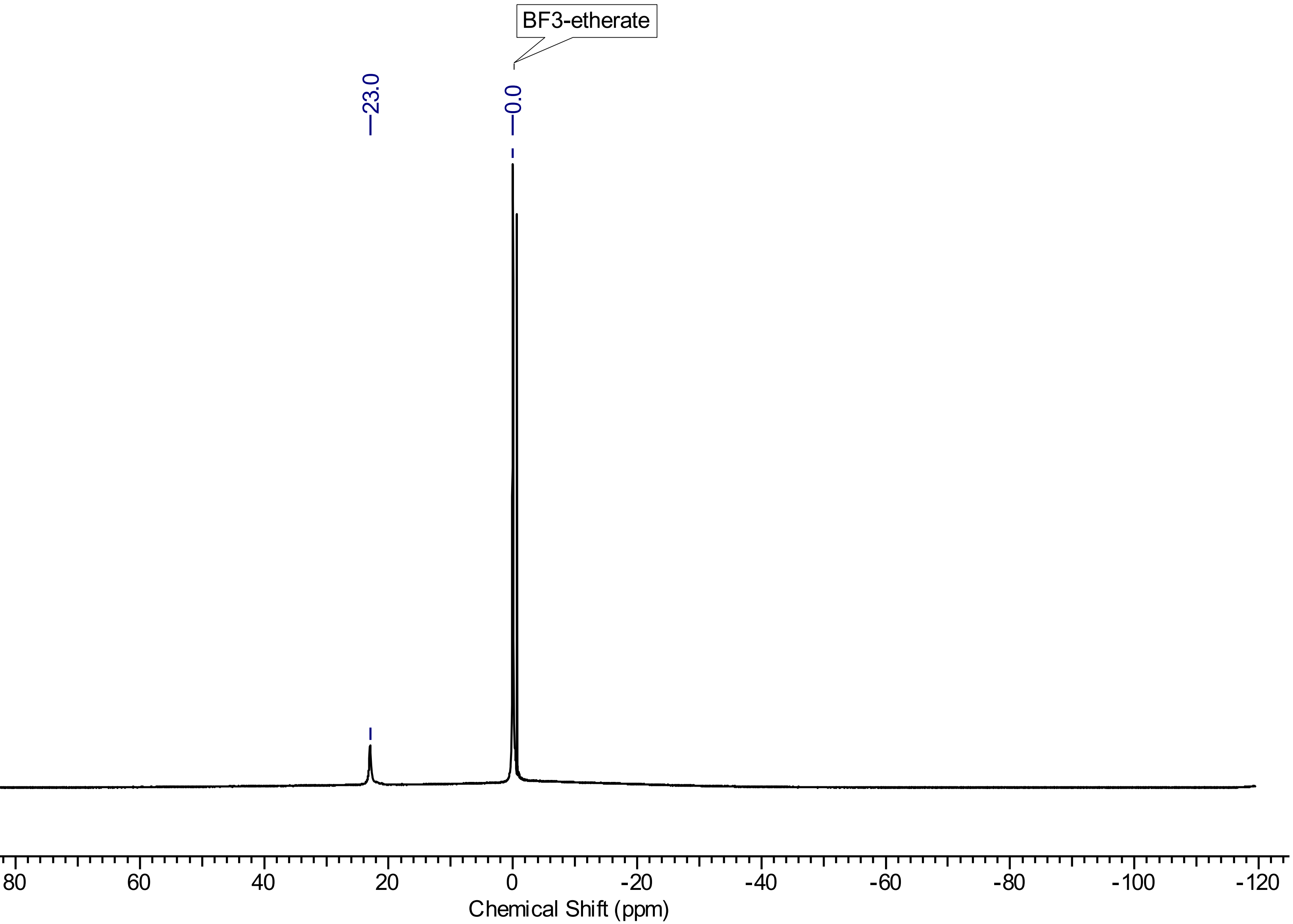

**F-Bpin**: $^{19}$F NMR (376 MHz, $CD_3CN$)

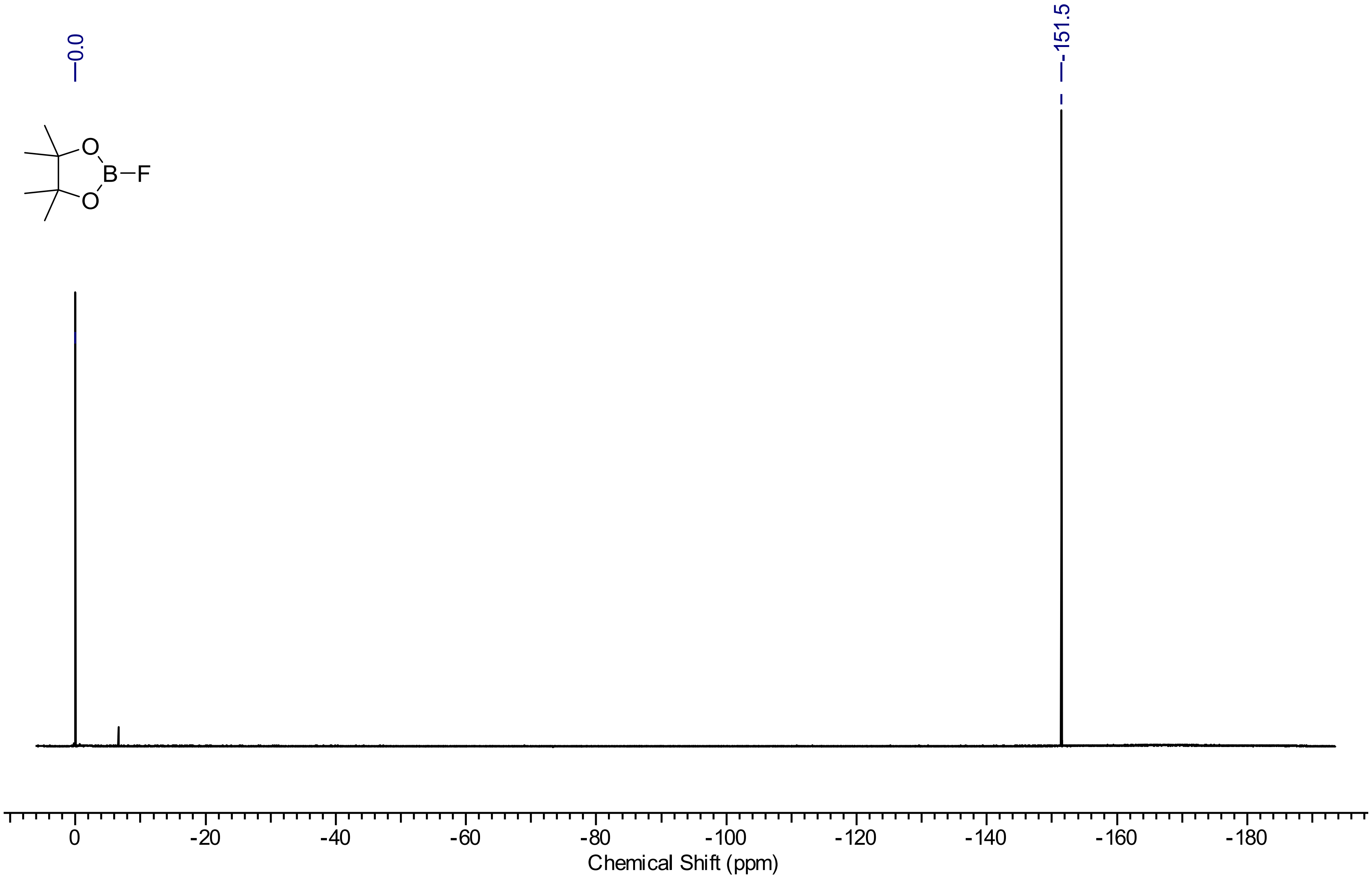

**2a**: $^{1}$H NMR (400 MHz, $CDCl_3$)

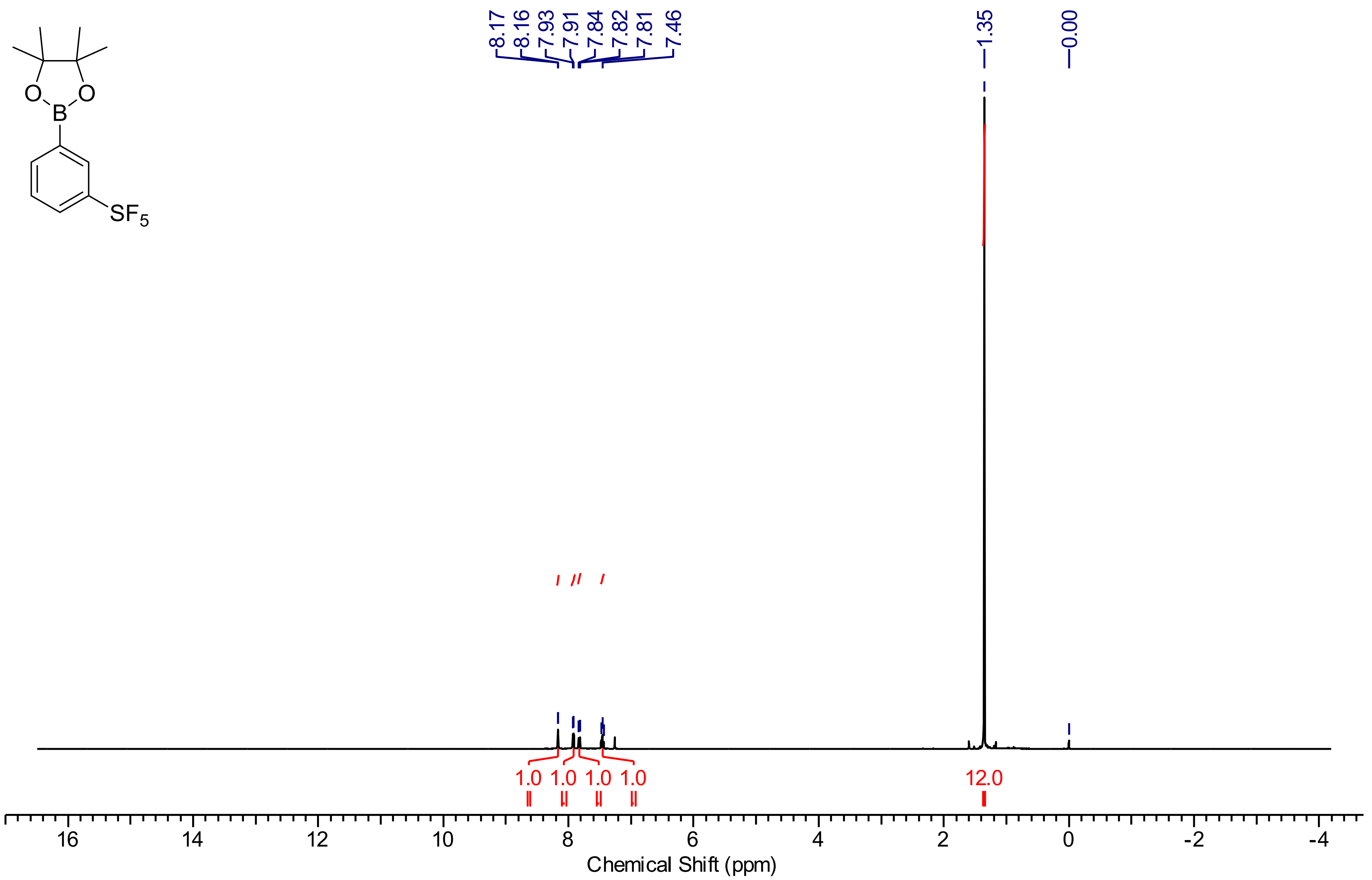

**2a**: $^{11}$B NMR (128 MHz, $CDCl_3$)

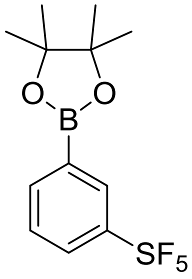


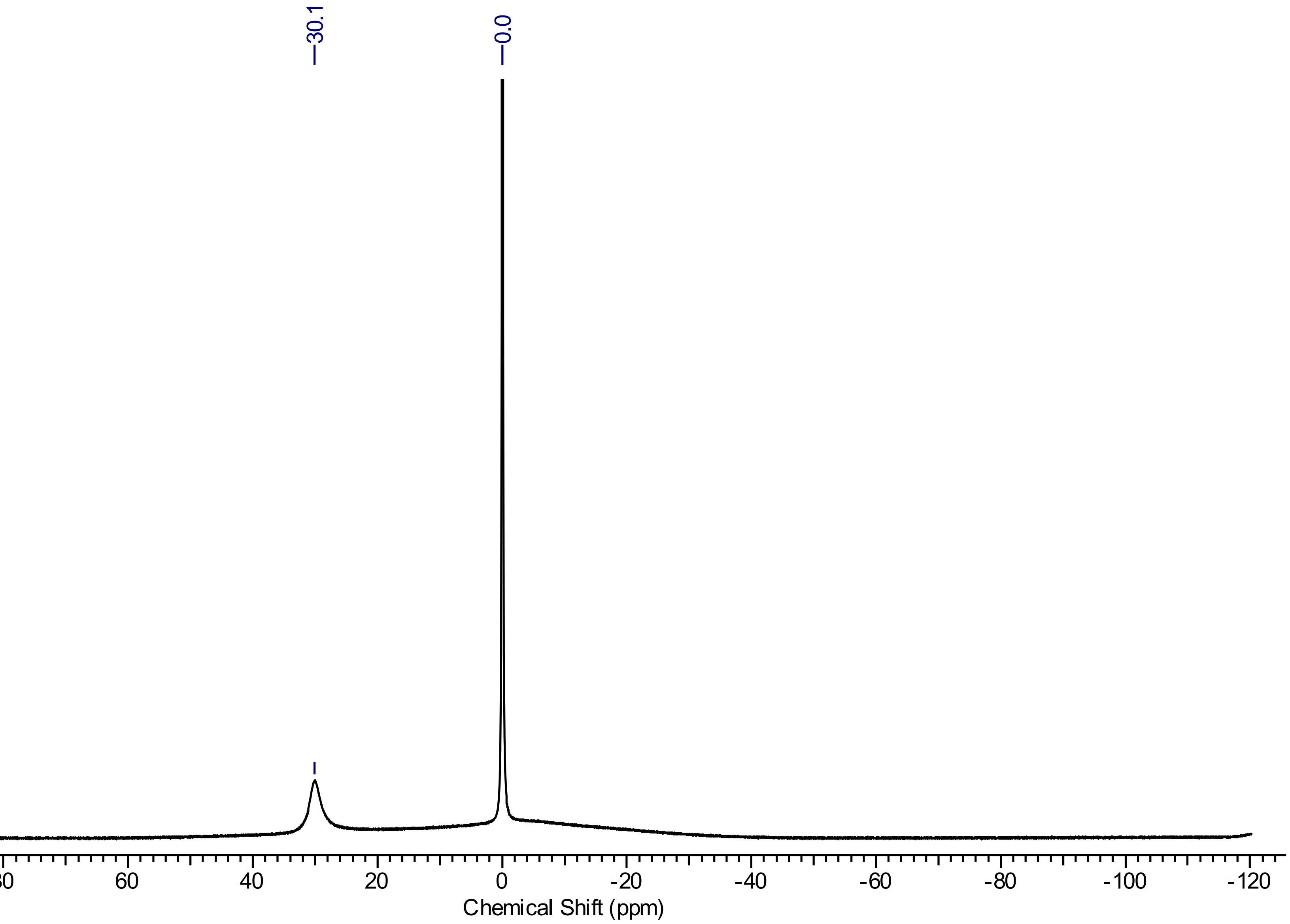

**2a**: $^{13}$C NMR (101 MHz, $CDCl_3$)

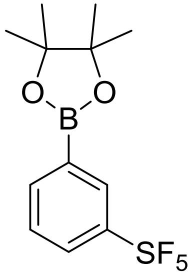


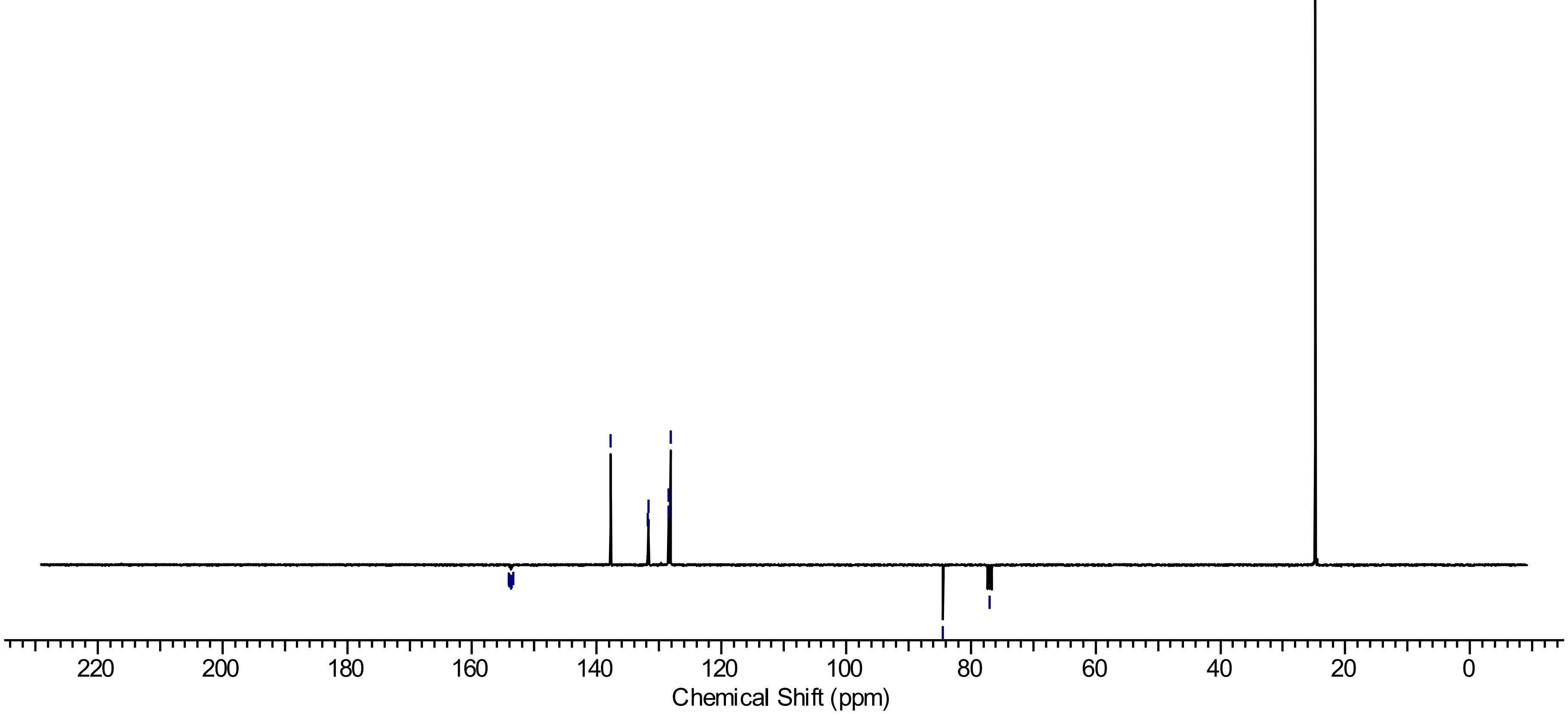

**2a**: $^{19}$F NMR (377 MHz, $CDCl_3$)

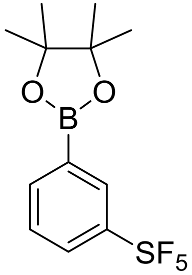


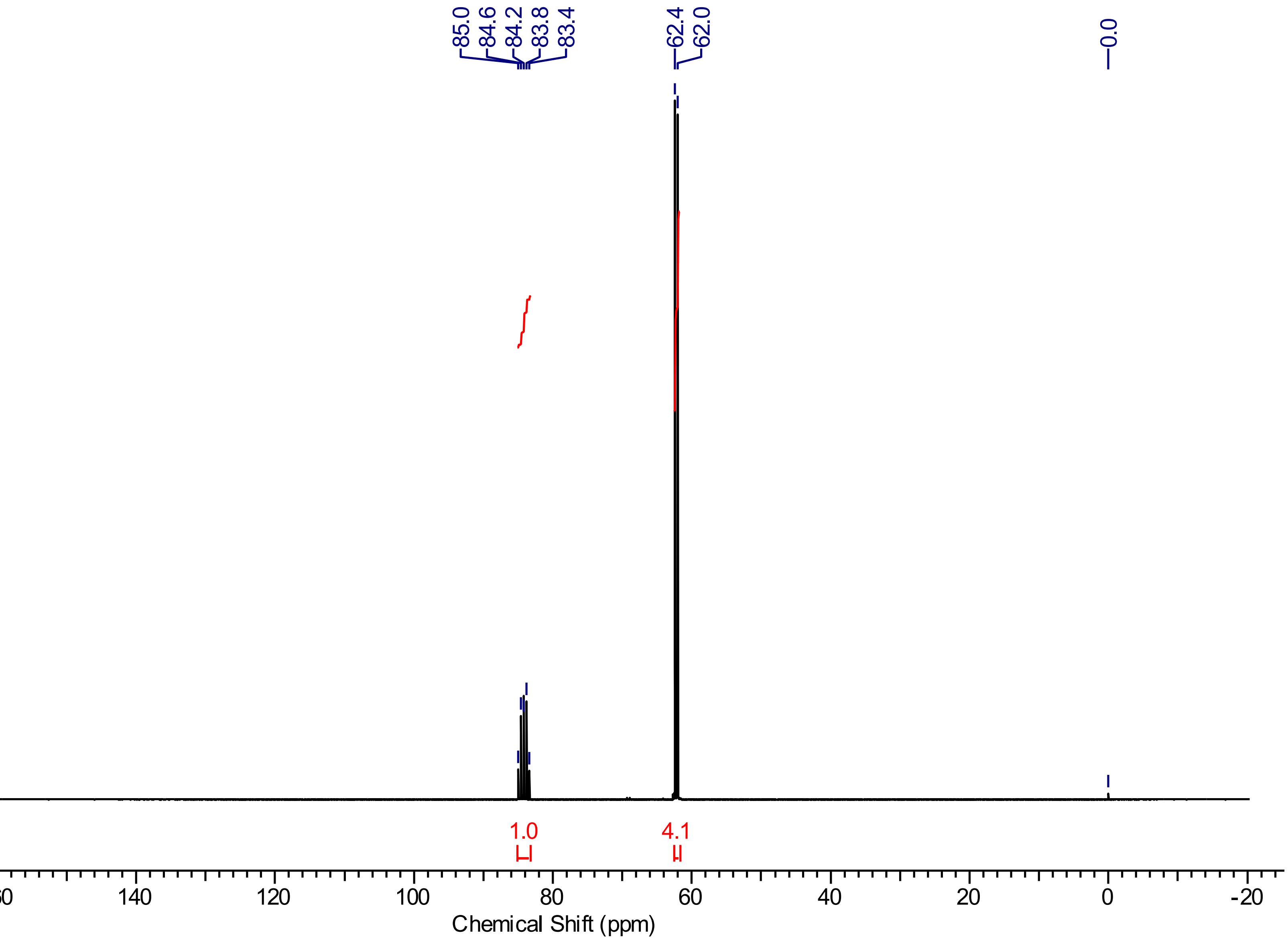

**2b**: $^{1}$H NMR (400 MHz, $CDCl_3$)

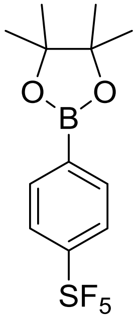

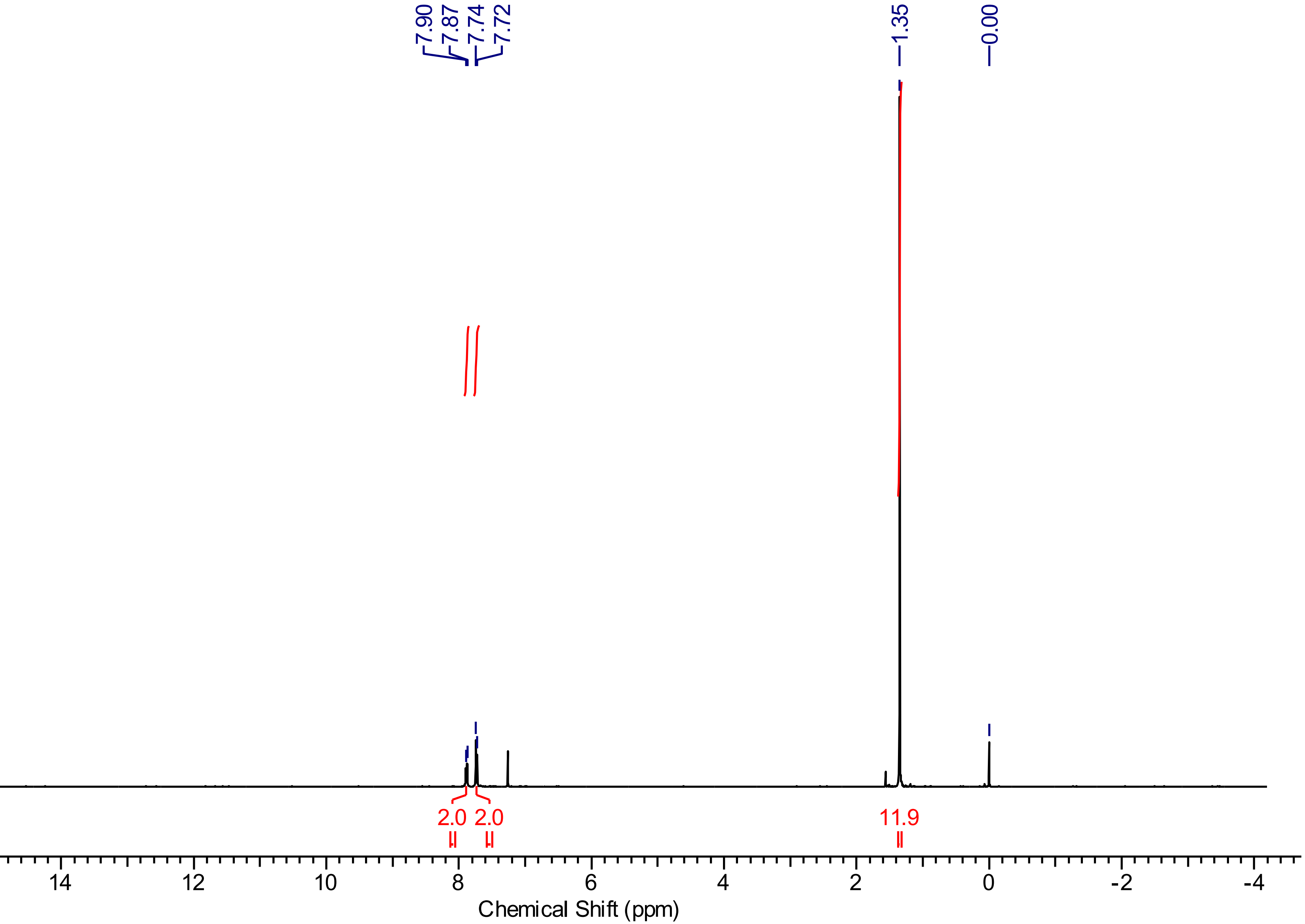

**2b**: $^{11}$B NMR (128 MHz, $CDCl_3$)

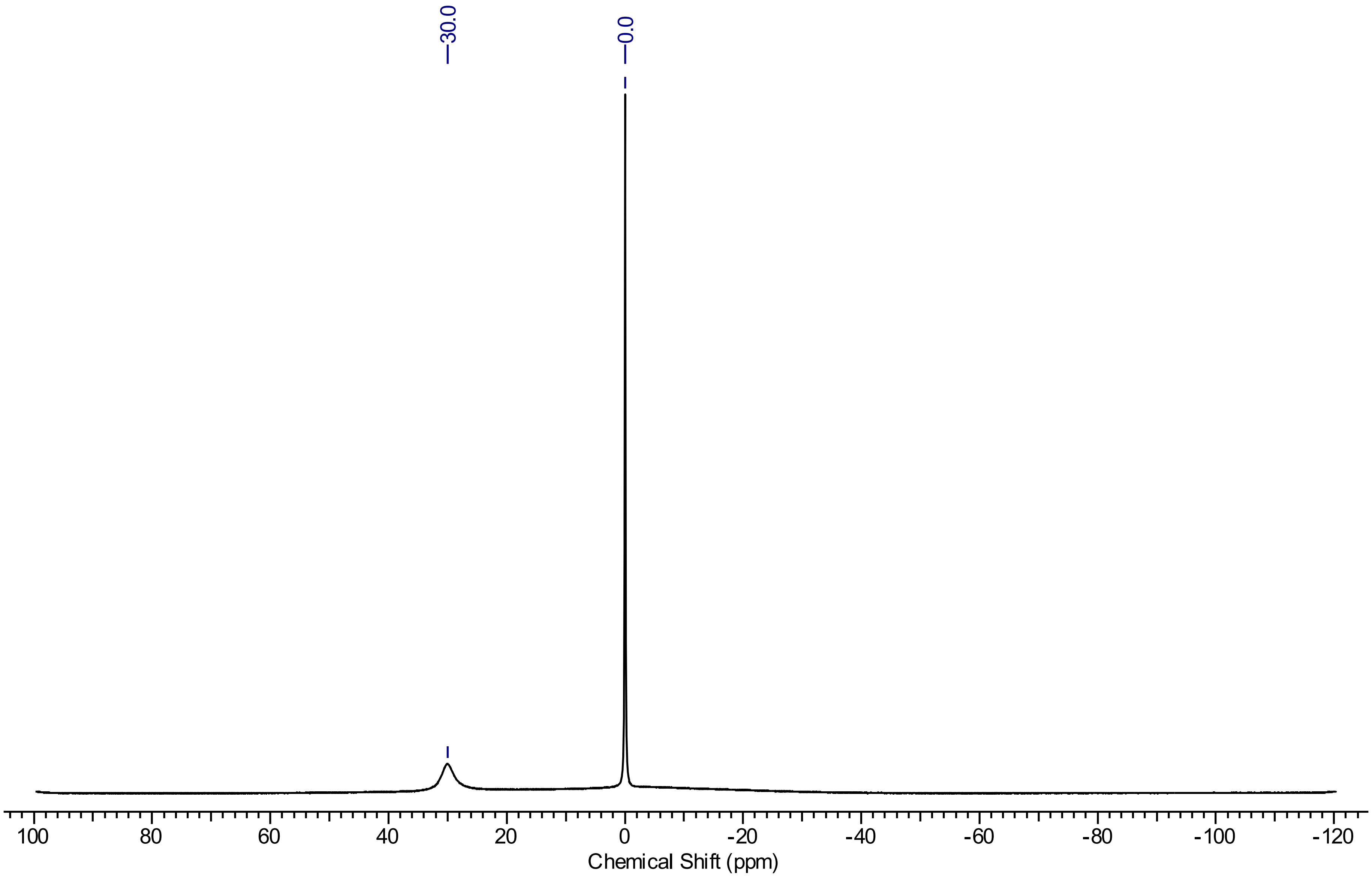

**2b**: $^{13}$C NMR (101 MHz, $CDCl_3$)

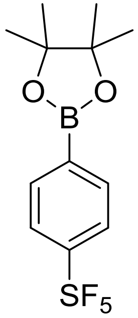


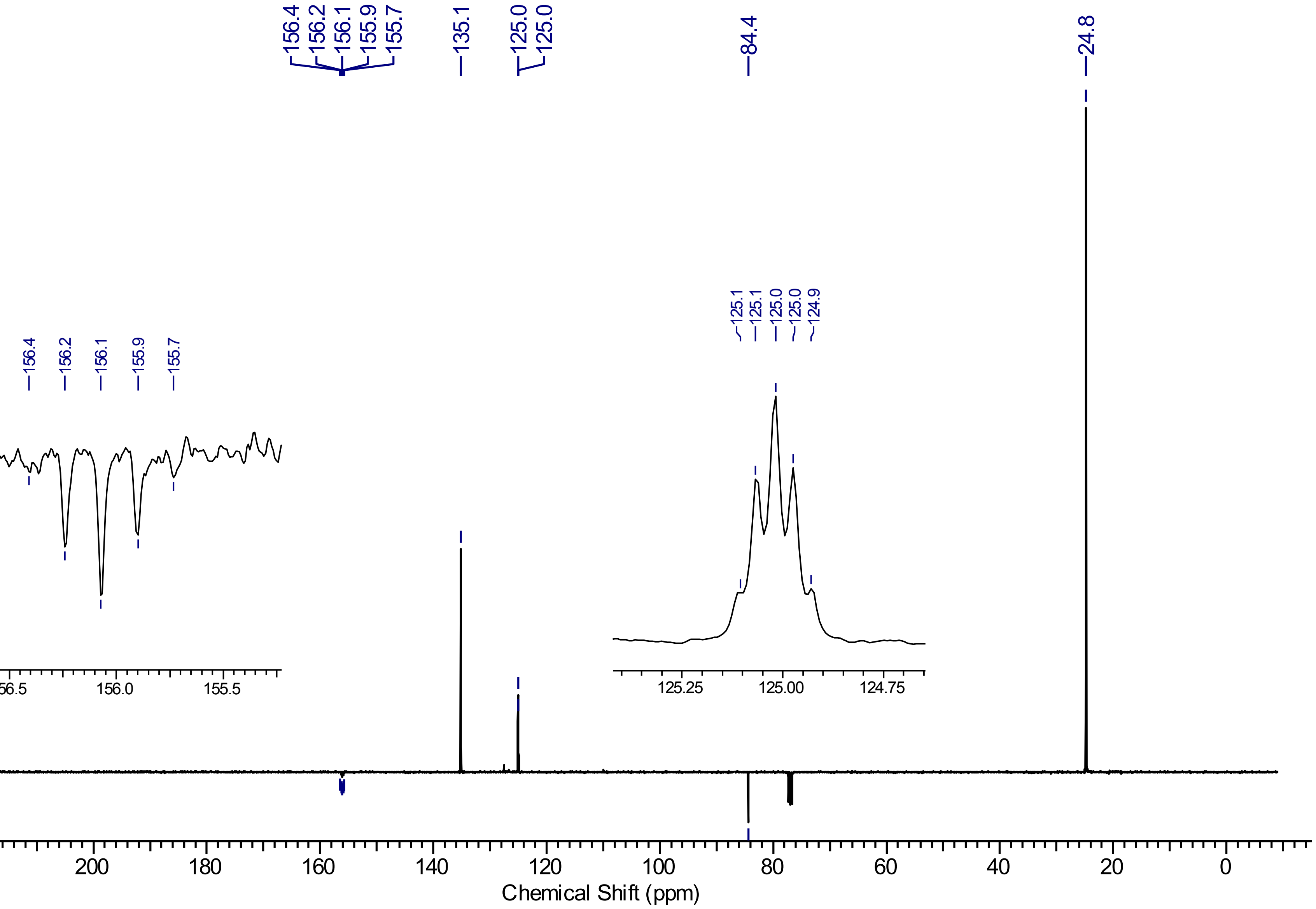

**2b**: $^{19}$F NMR (377 MHz, $CDCl_3$)

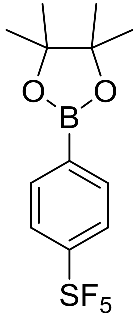

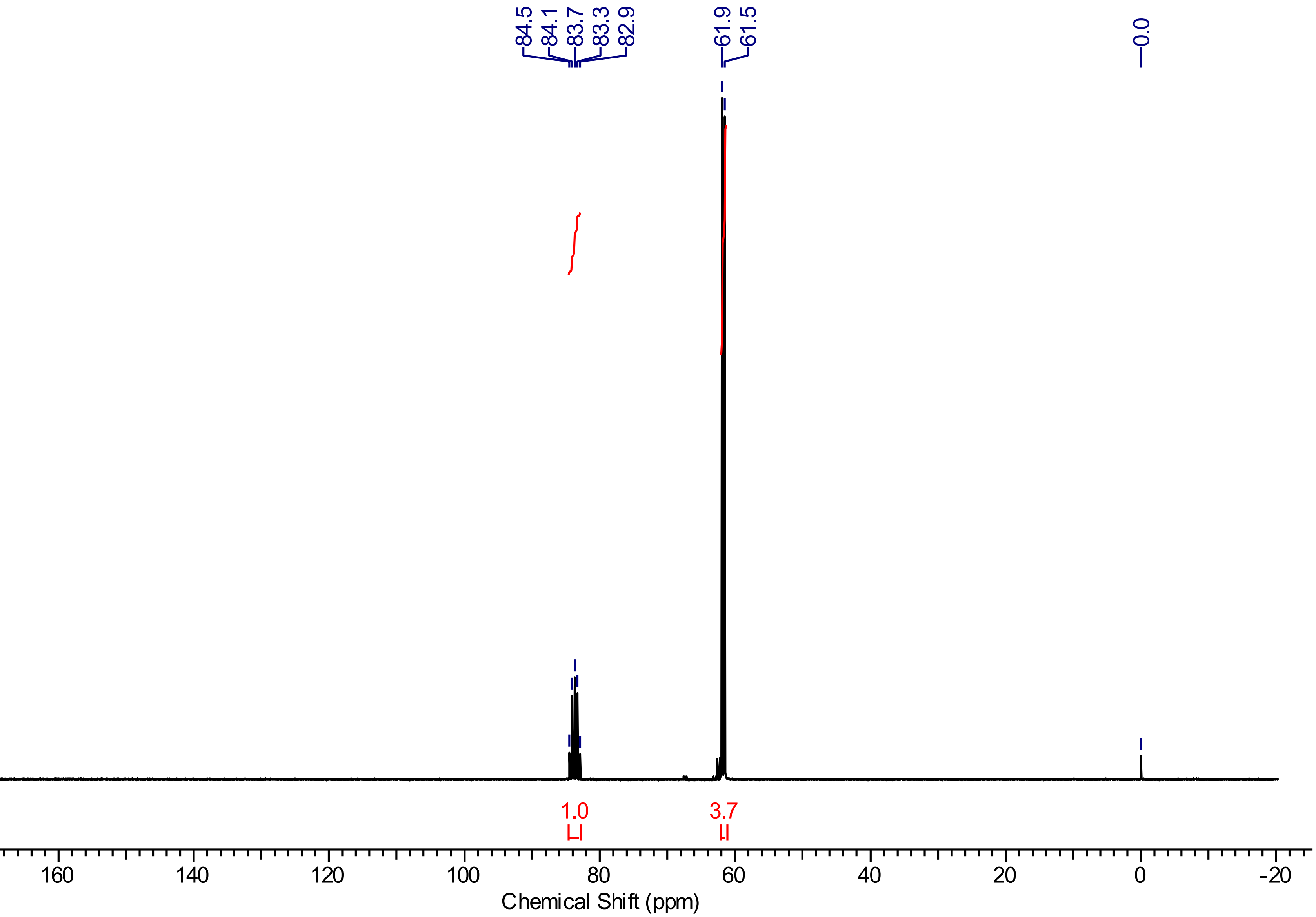

**2c**: $^{1}H$ NMR (400 MHz, $CDCl_3$)

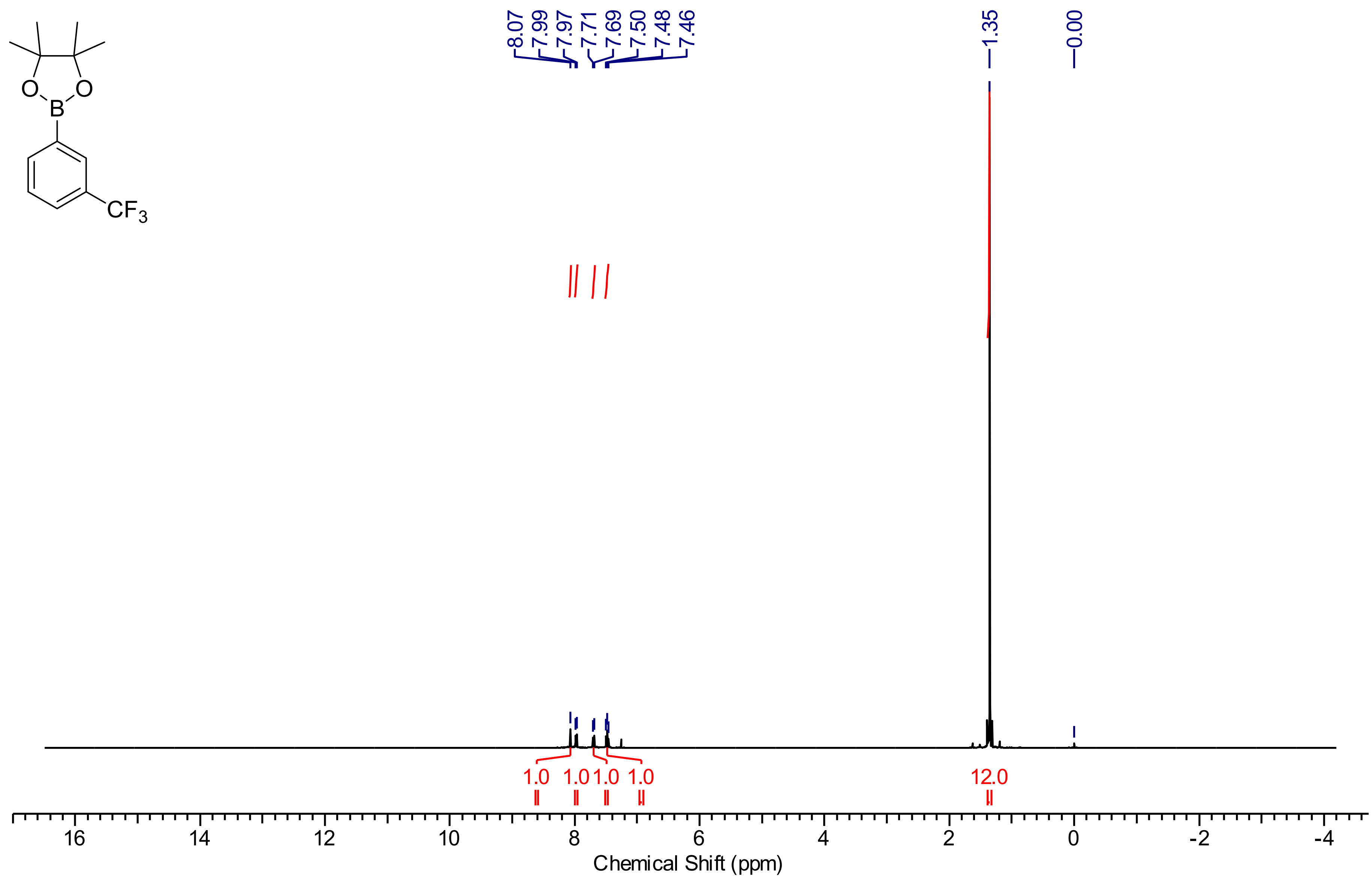

**2c**: $^{13}$C NMR (101 MHz, $CDCl_3$)

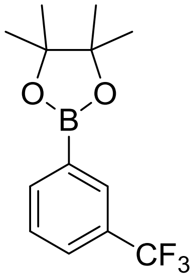

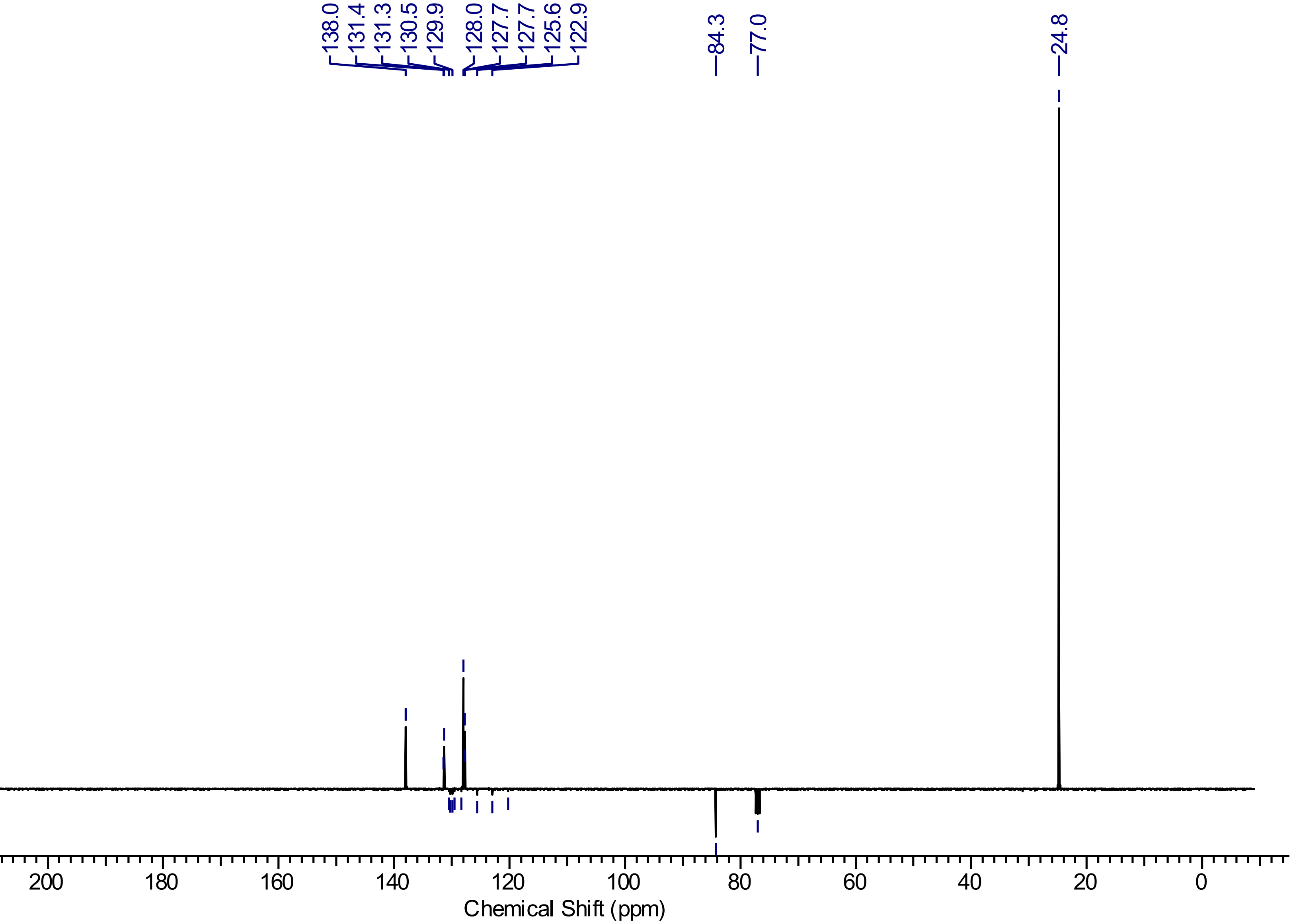

**2c**: $^{19}$F NMR (376 MHz, $CDCl_3$)

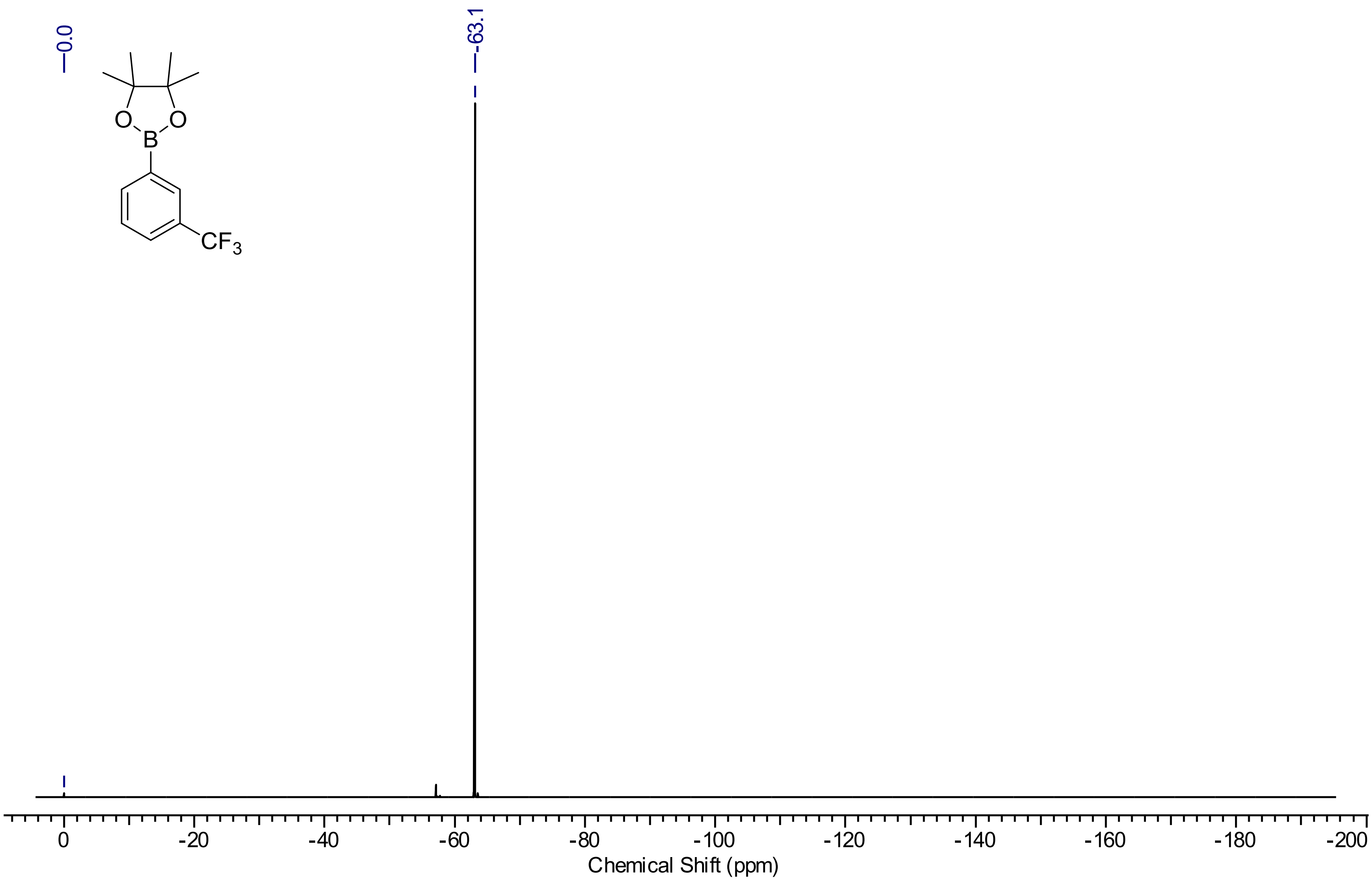

**2d**: $^{1}H$ NMR (400 MHz, $CDCl_3$)

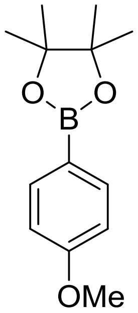

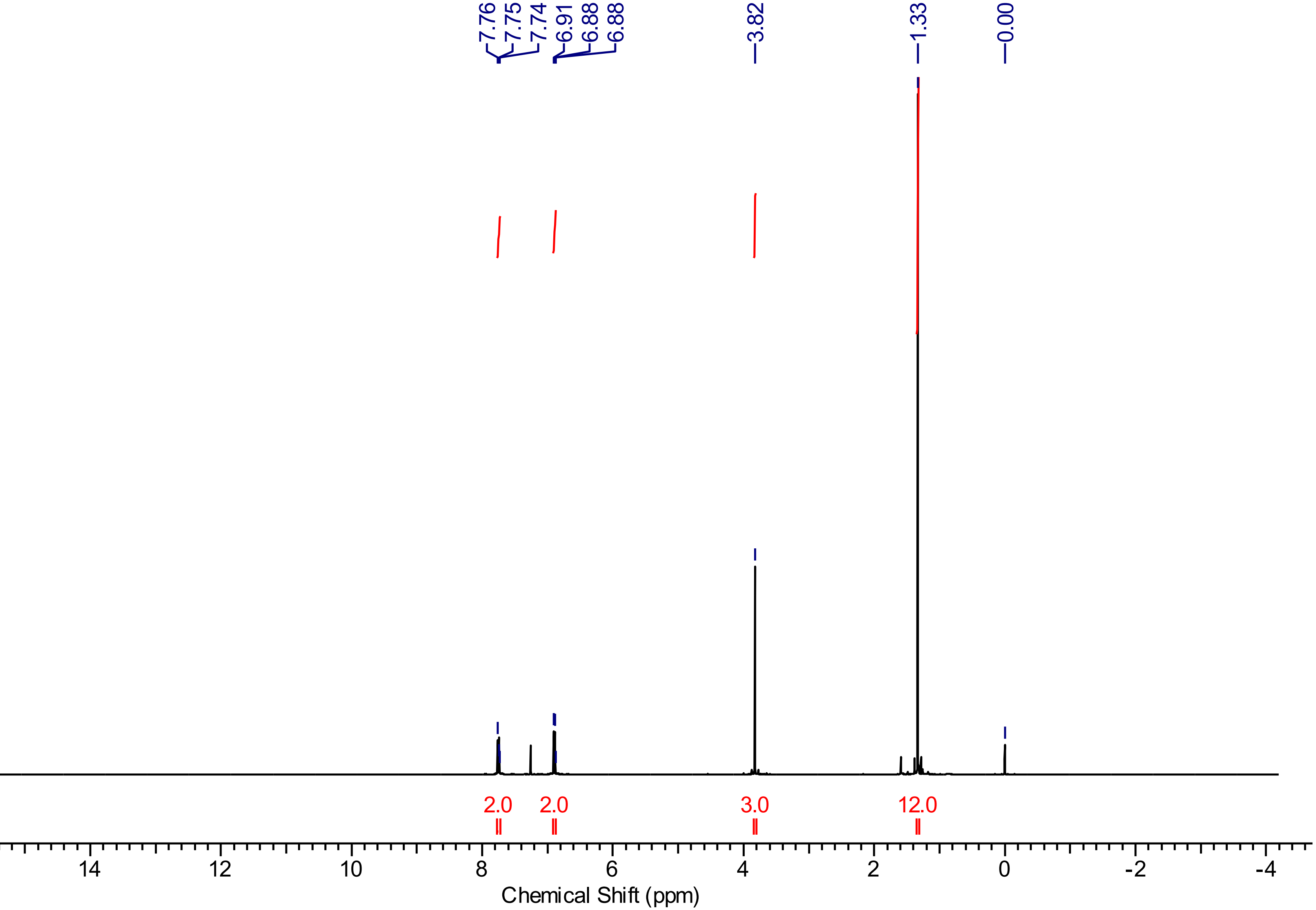

**2d**: $^{13}C$ NMR (101 MHz, $CDCl_3$)

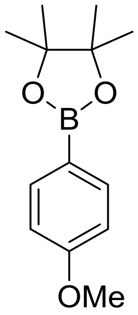


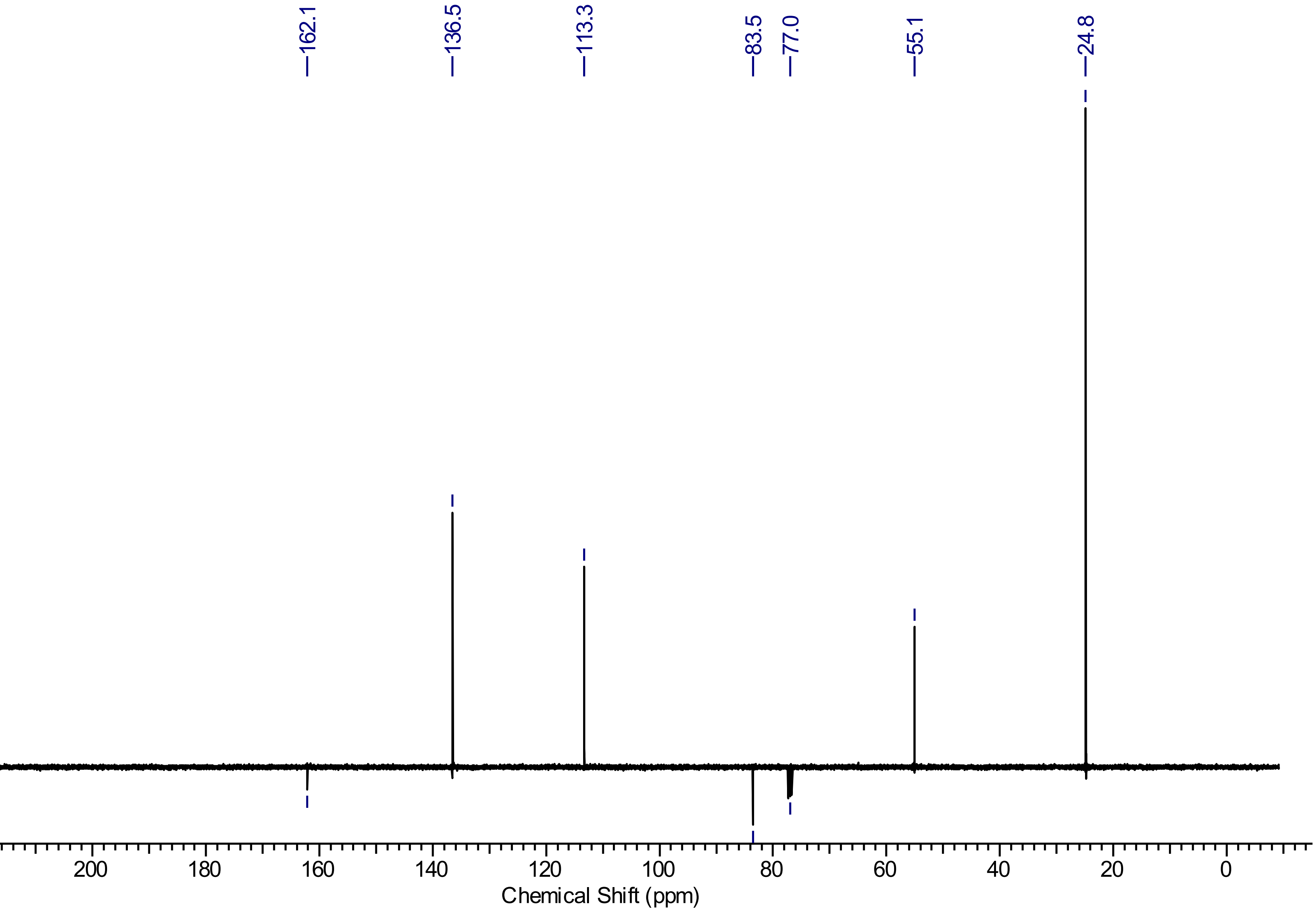

**2e**: $^{1}$H NMR (400 MHz, $CDCl_3$)

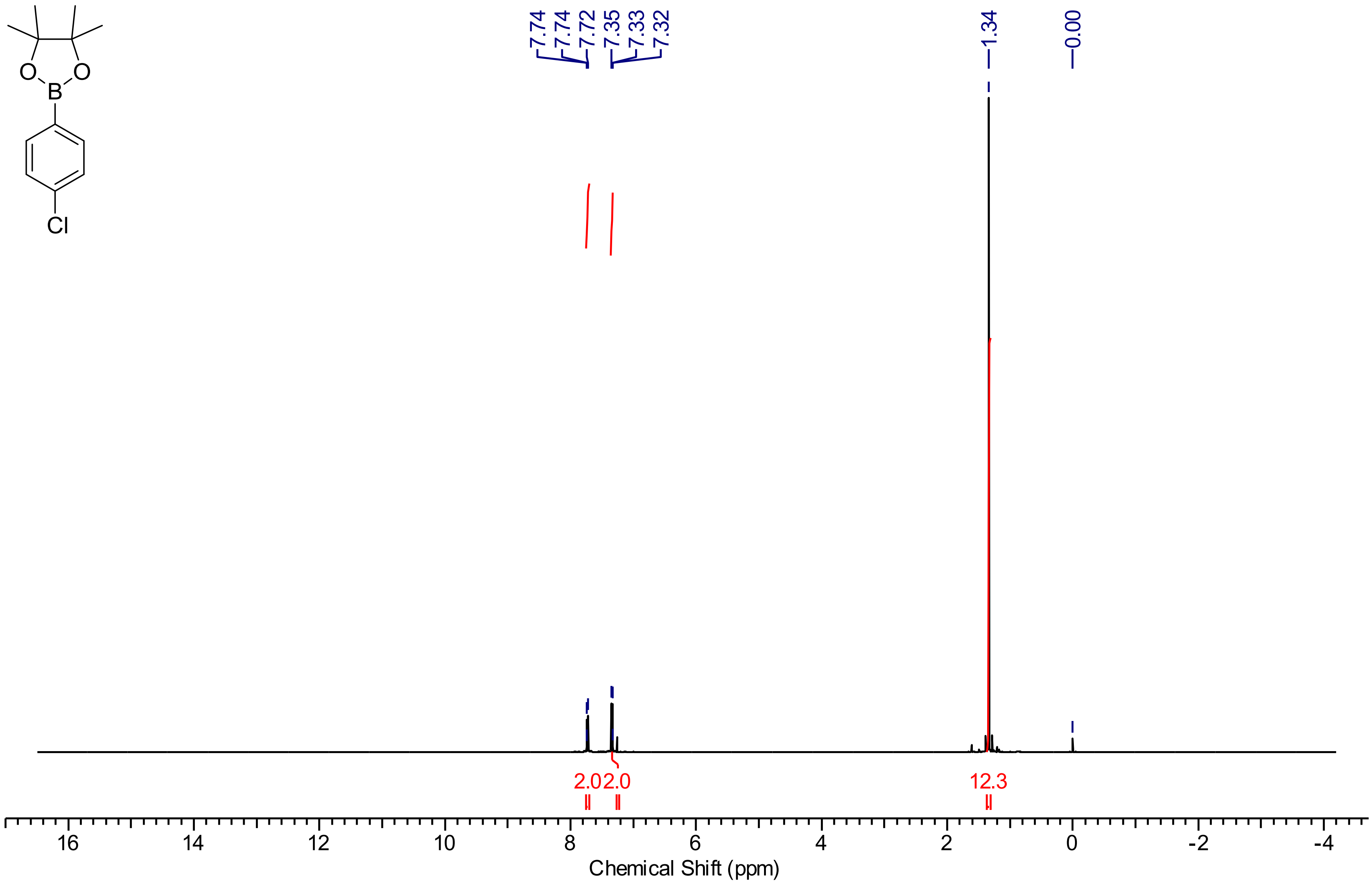

**2e**: $^{13}$C NMR (101 MHz, $CDCl_3$)

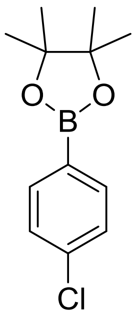


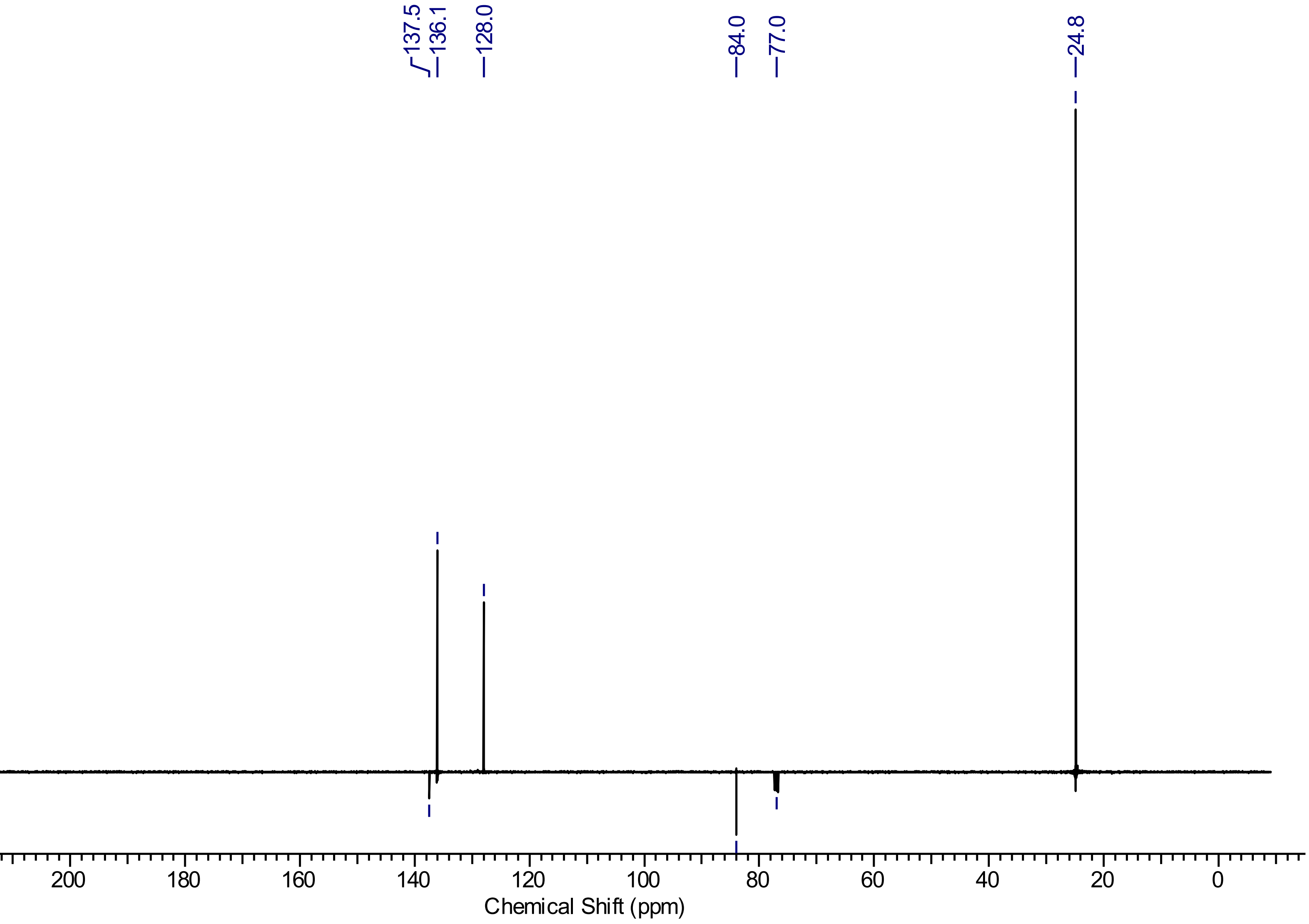

**2f**: $^1$H NMR (400 MHz, $CDCl_3$)

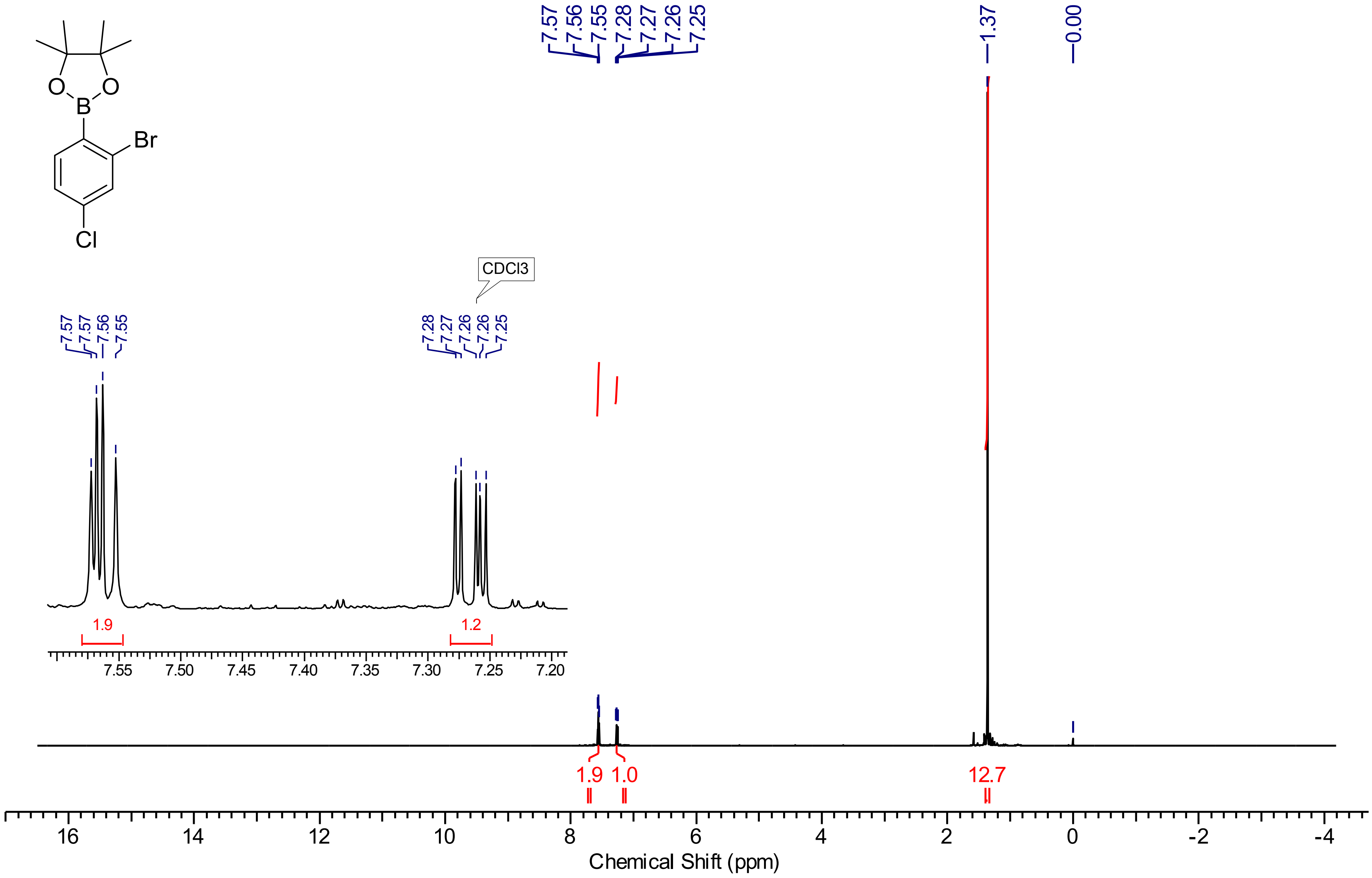

**2f**: $^{13}$C NMR (101 MHz, $CDCl_3$)

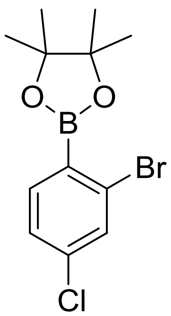

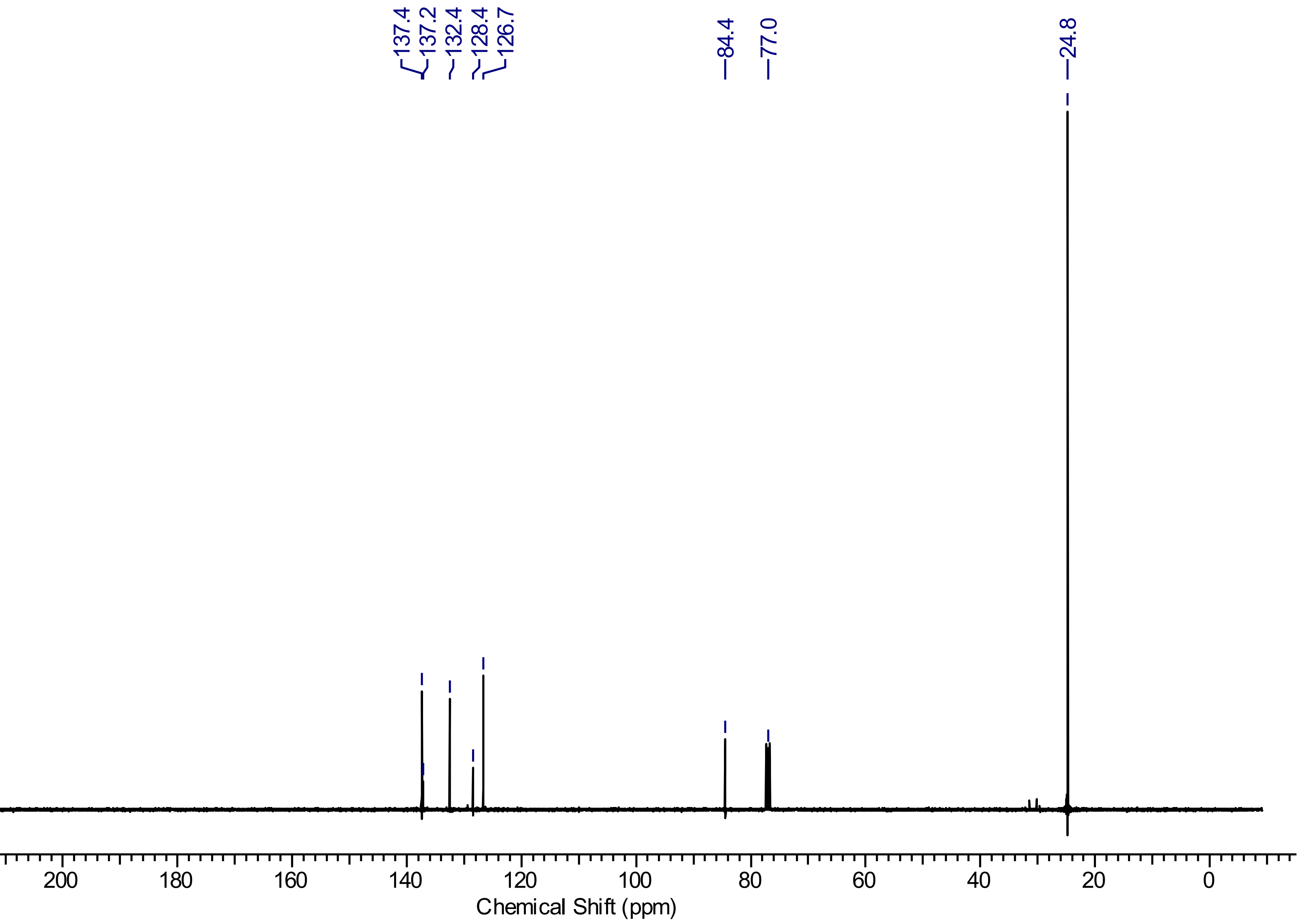

**2h**: $^{1}H$ NMR (400 MHz, $CDCl_3$)

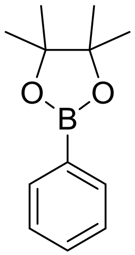

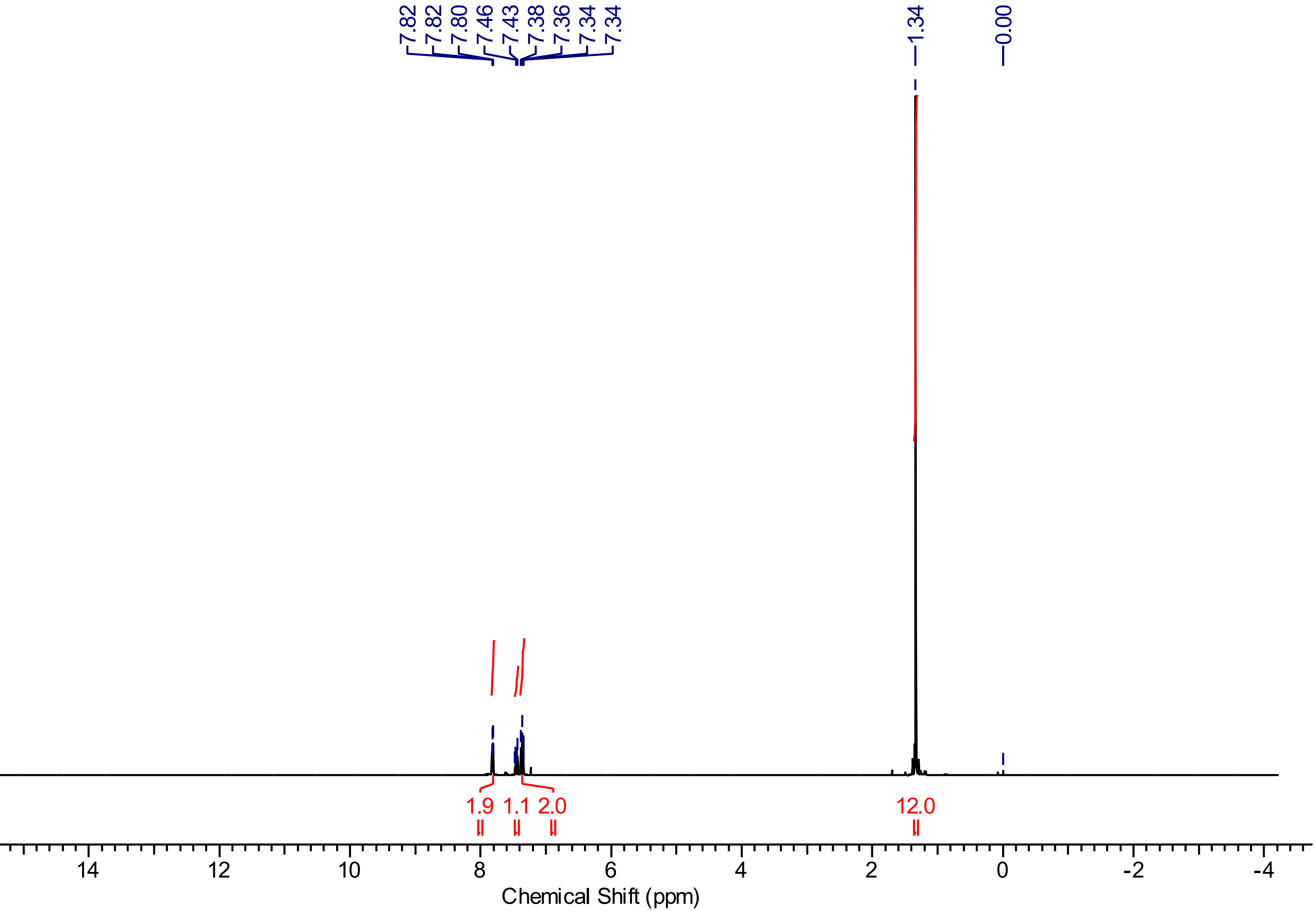

**2h**: $^{13}C$ NMR (101 MHz, $CDCl_3$)

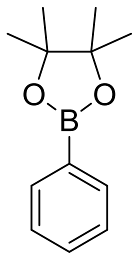

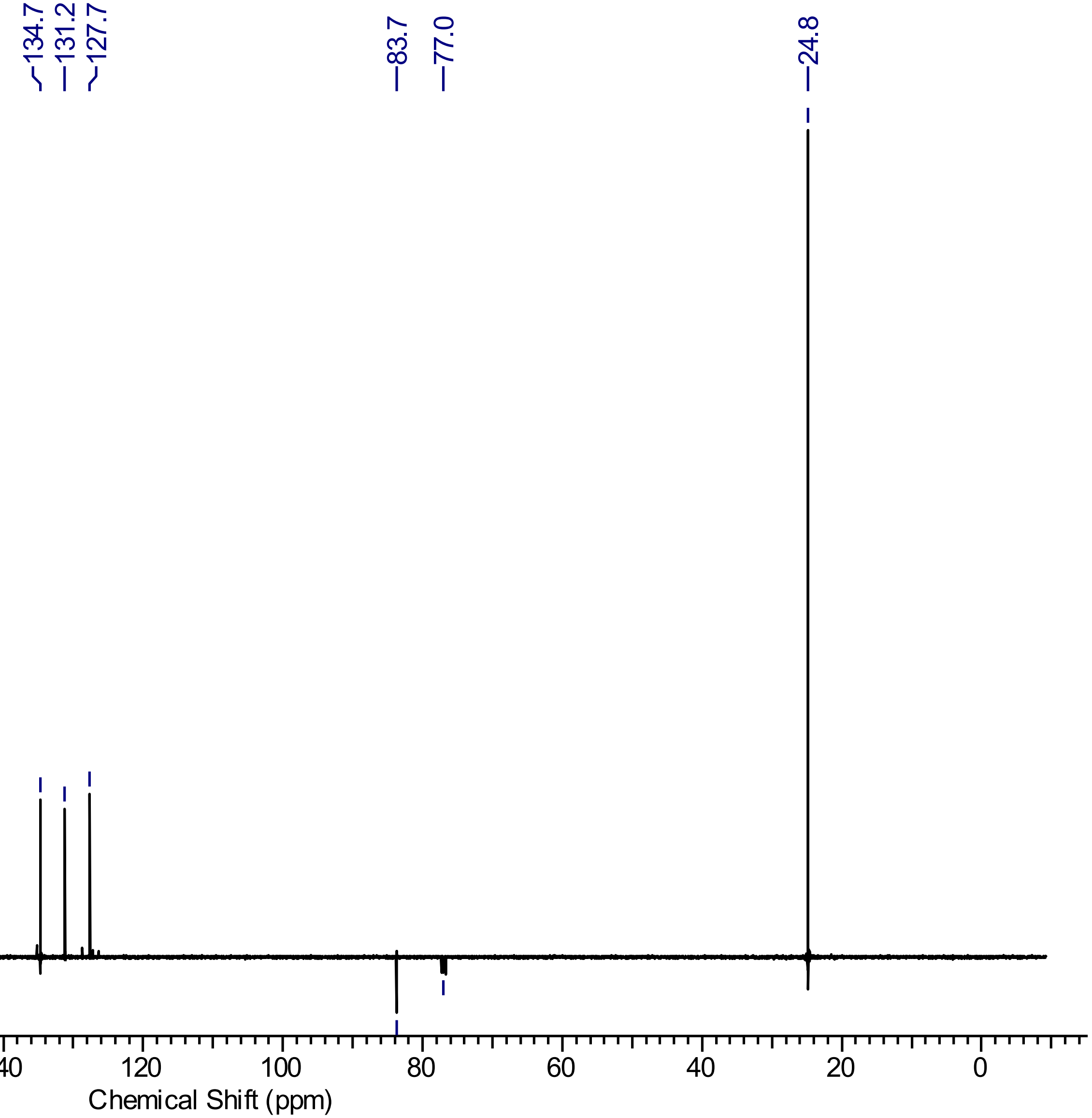

**7ah**: $^{1}$H NMR (400 MHz, $CDCl_3$)

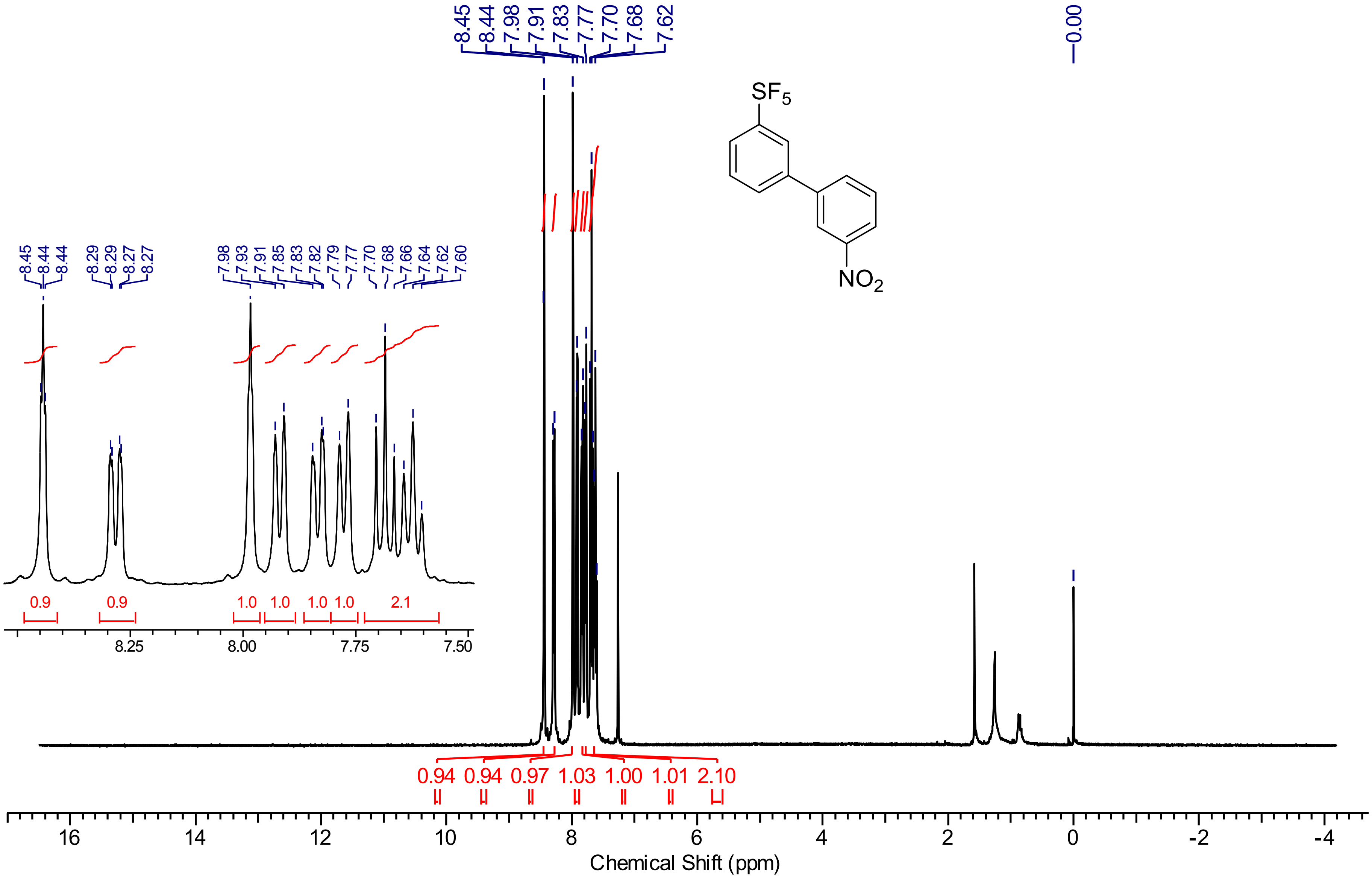

**7ah**: $^{13}$C NMR (101 MHz, $CDCl_3$)

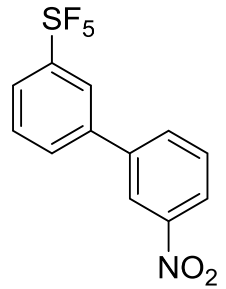

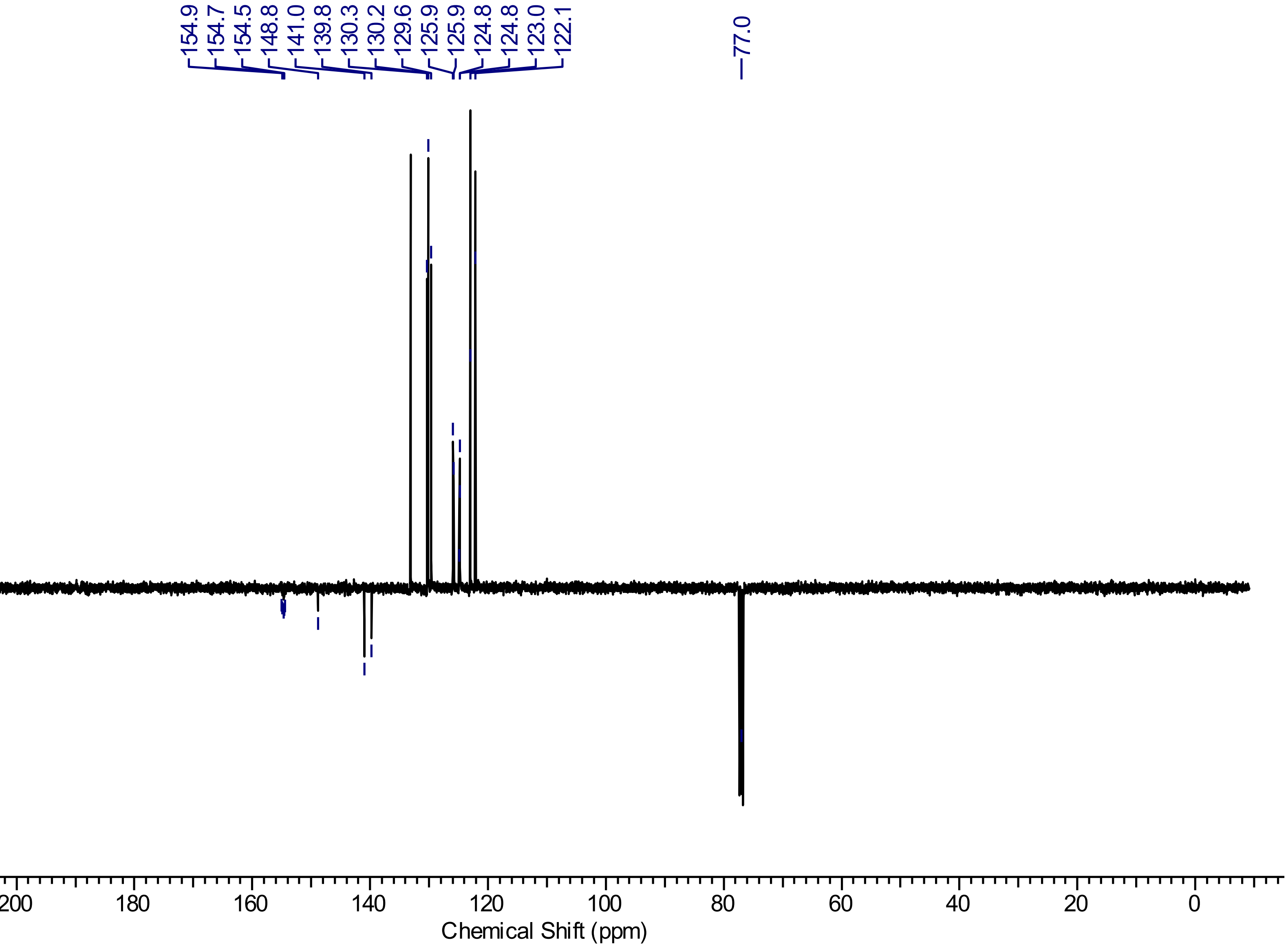

**7ah**: $^{19}$F NMR (377 MHz, $CDCl_3$)

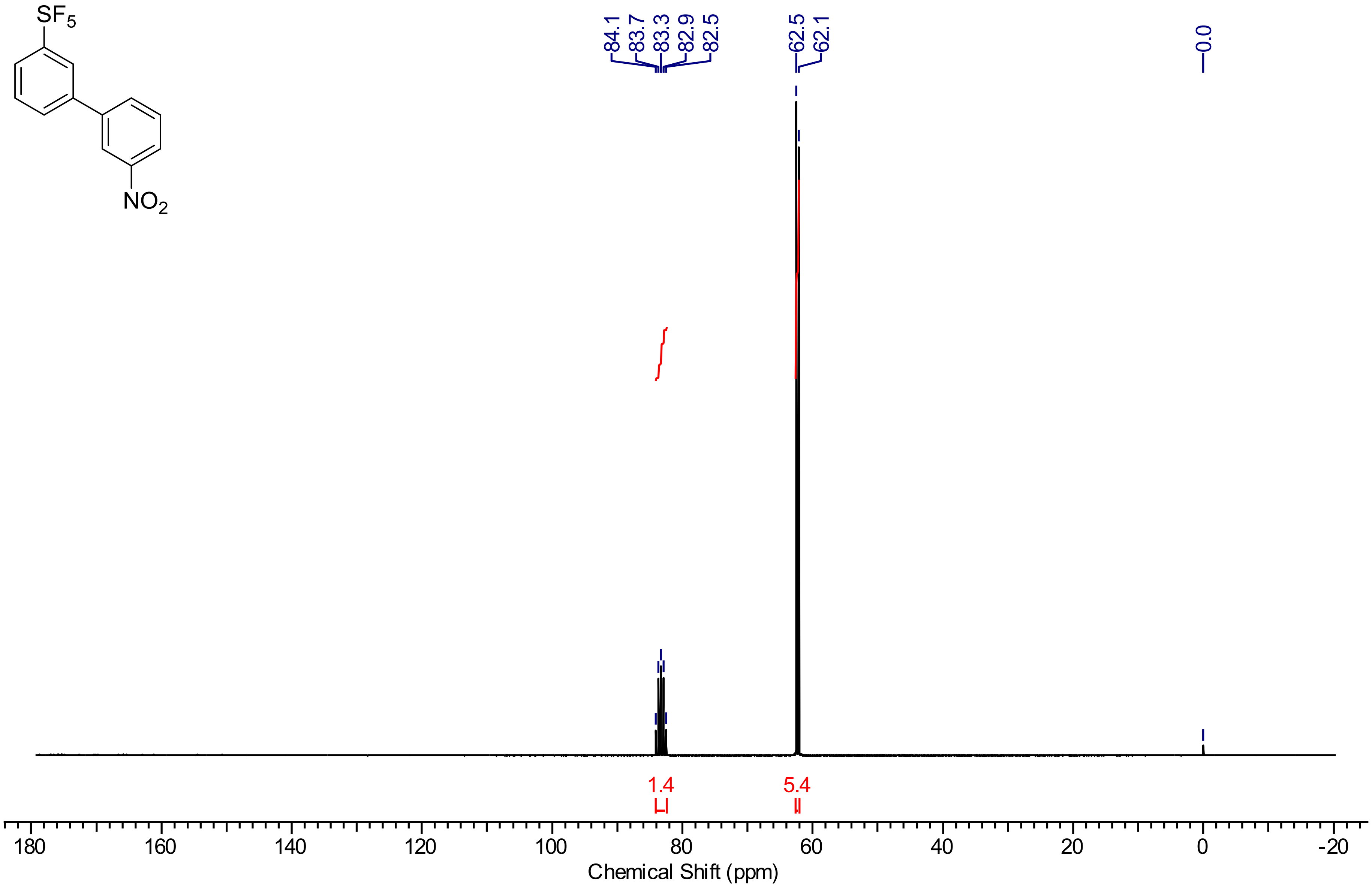

**7bc**: $^1$H NMR (400 MHz, $CDCl_3$)

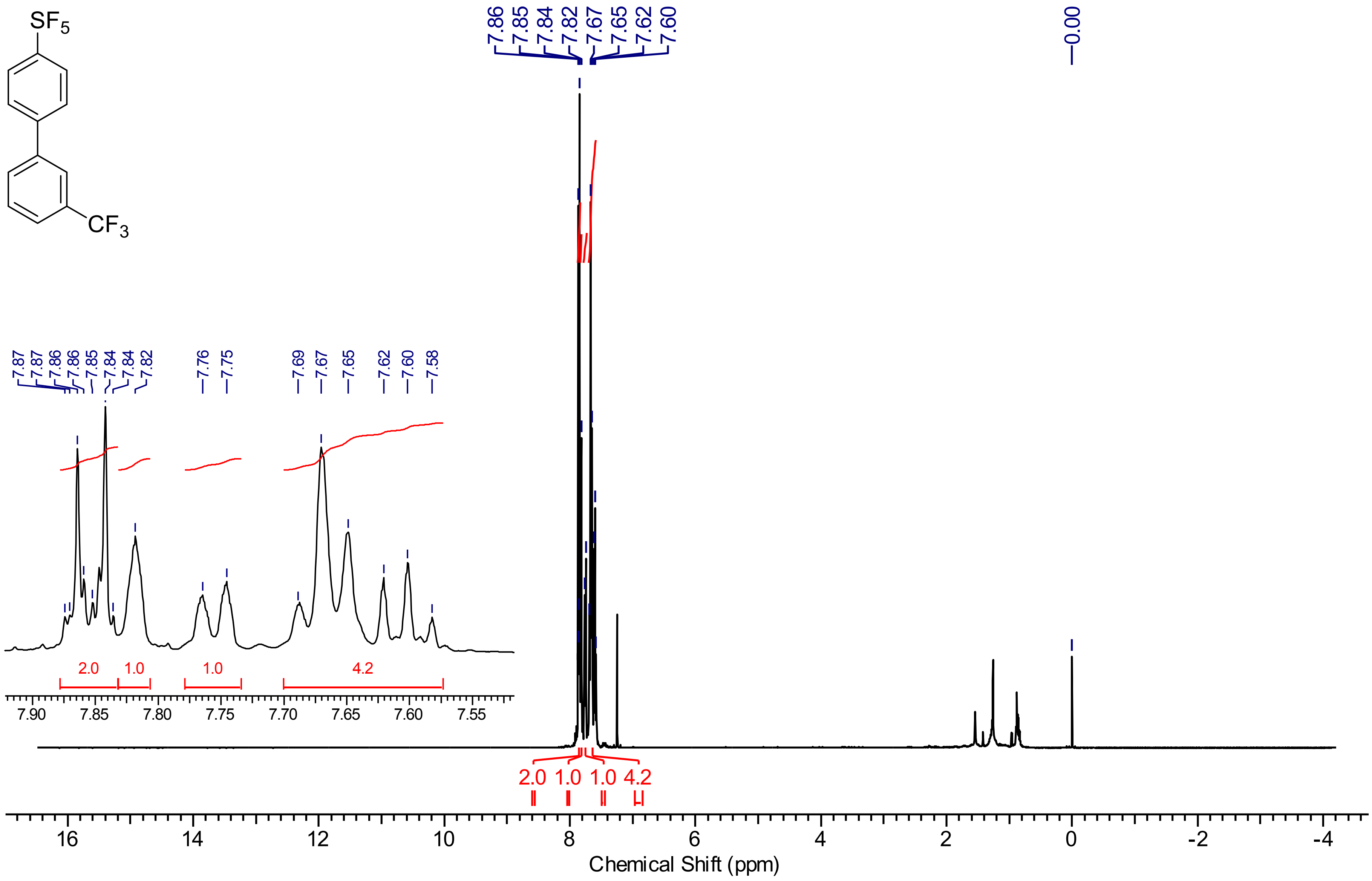

**7bc**: $^{13}C$ NMR (101 MHz, $CDCl_3$)

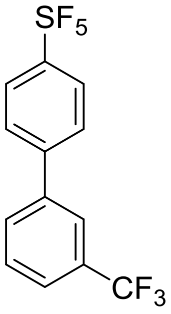


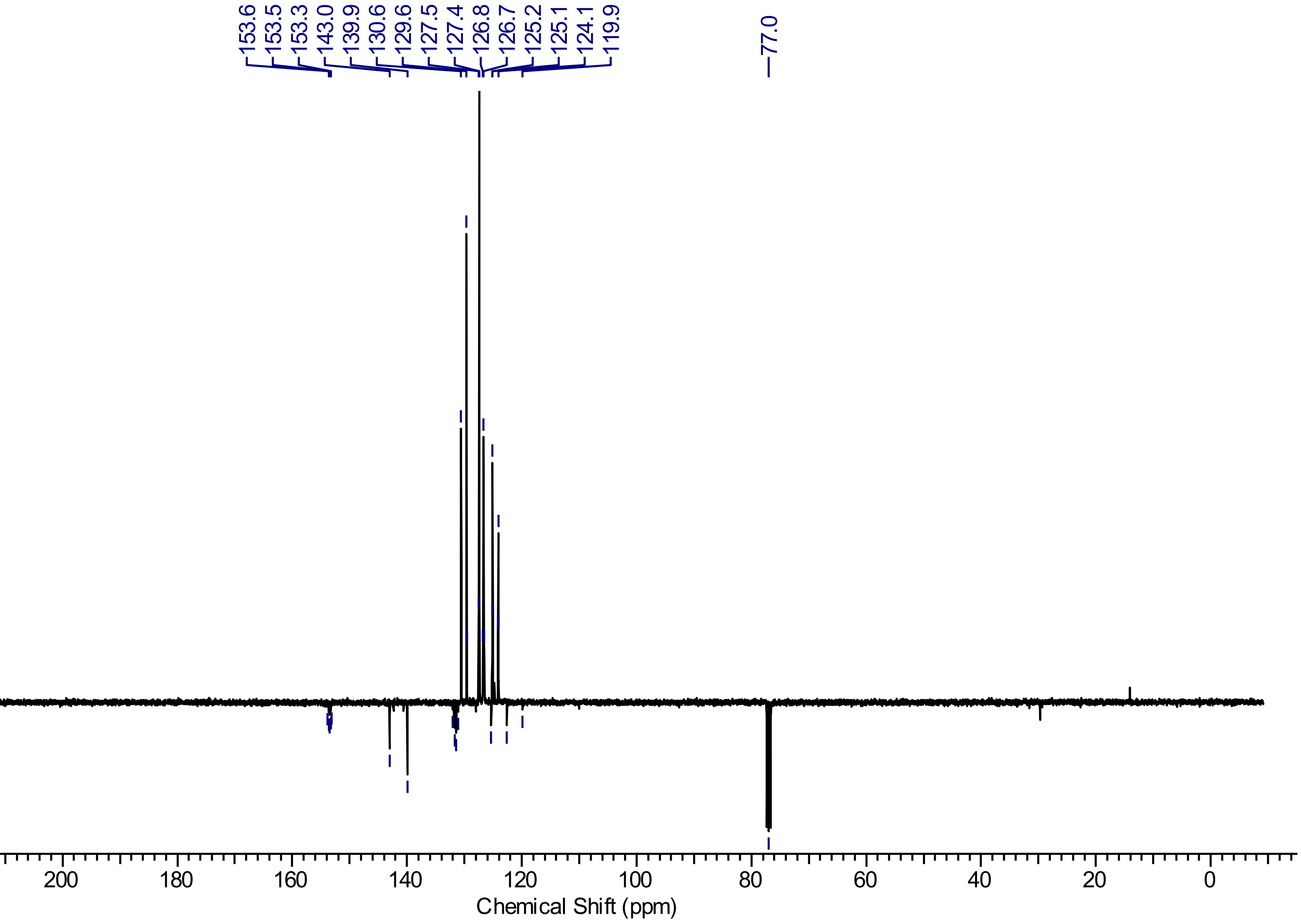

**7bc**: $^{19}$F NMR (377 MHz, $CDCl_3$)

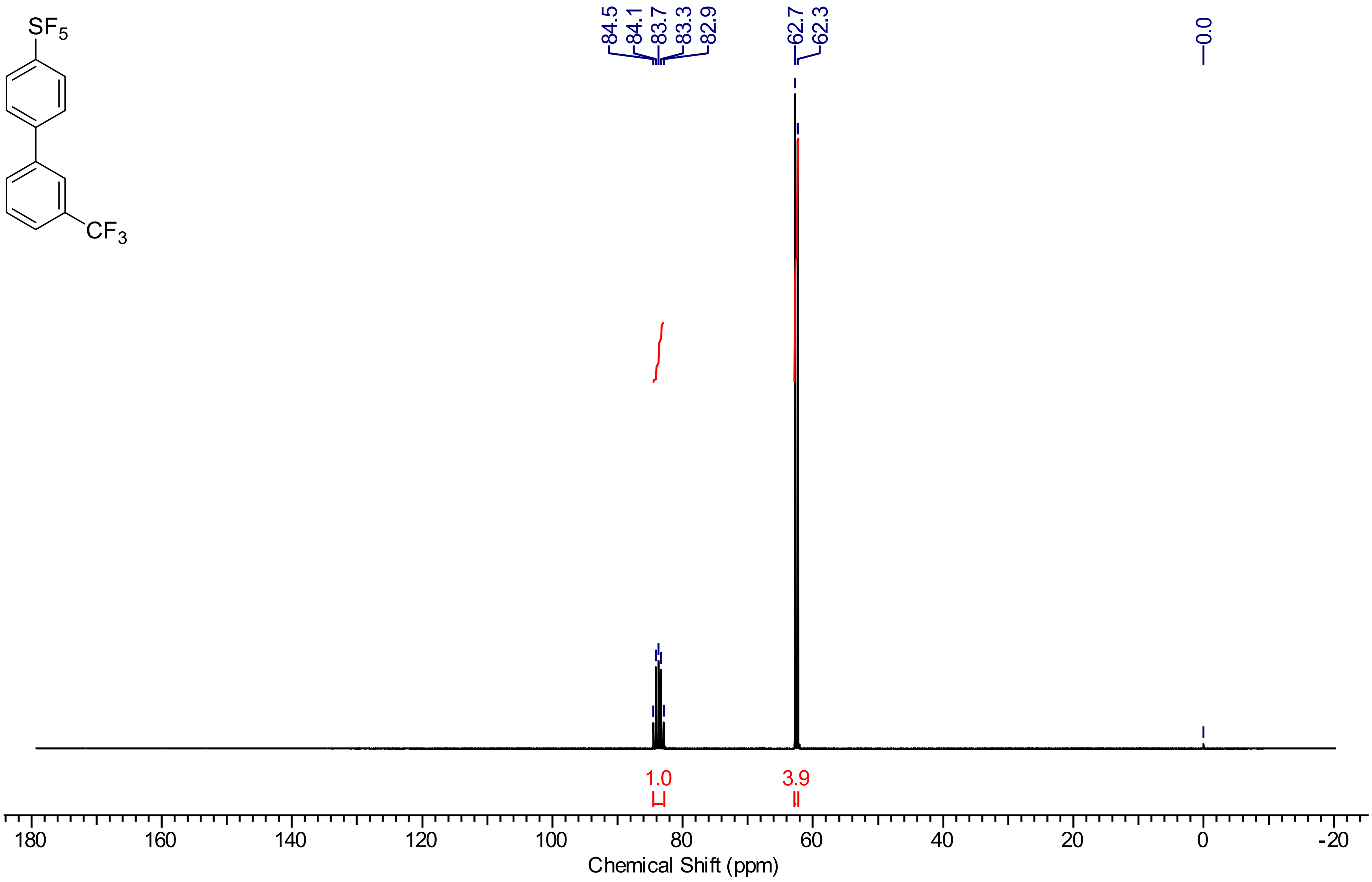

**7bc**: $^{19}$F NMR (377 MHz, $CDCl_3$)

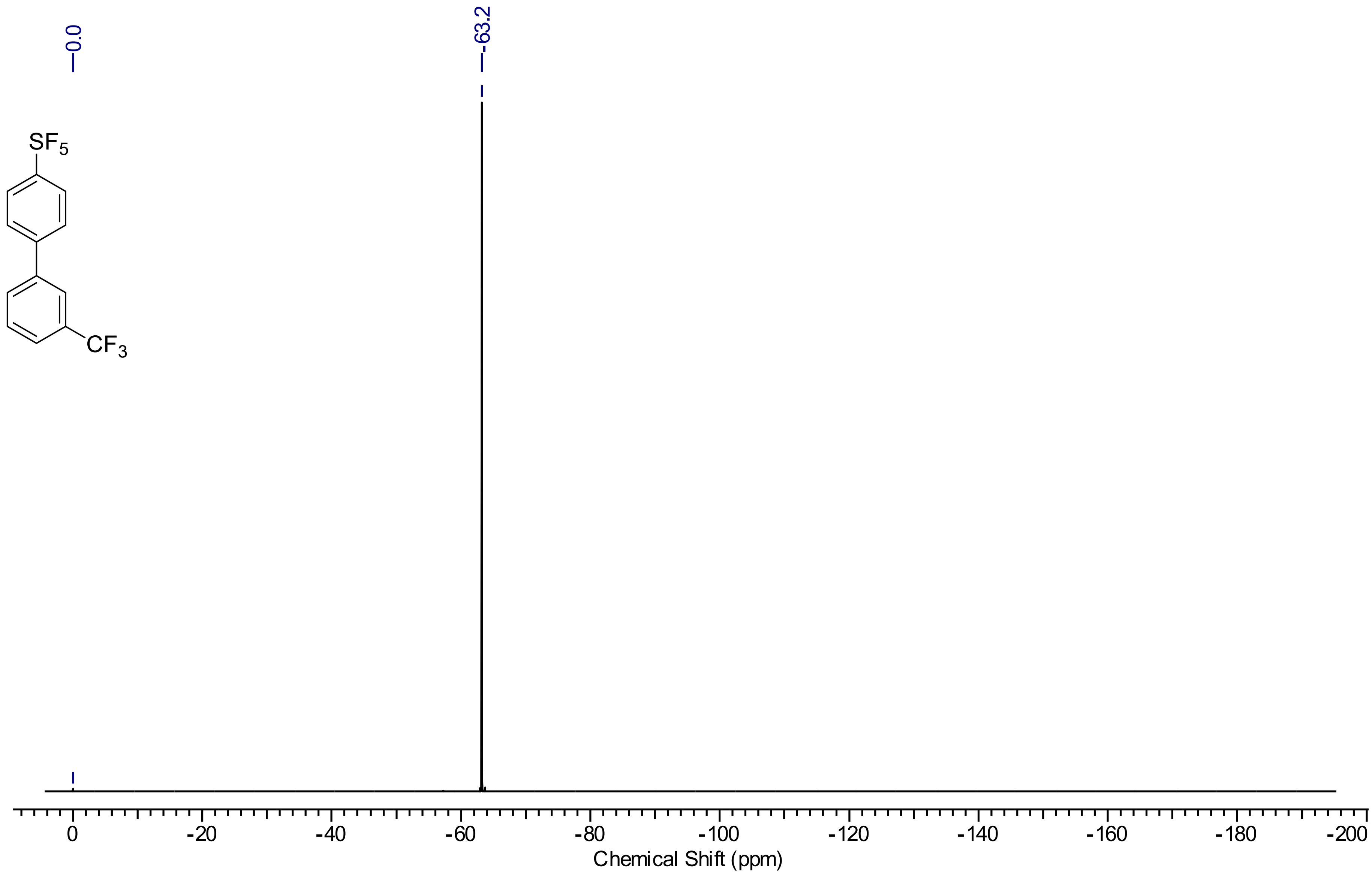

**7bd**: $^1$H NMR (400 MHz, $CDCl_3$)

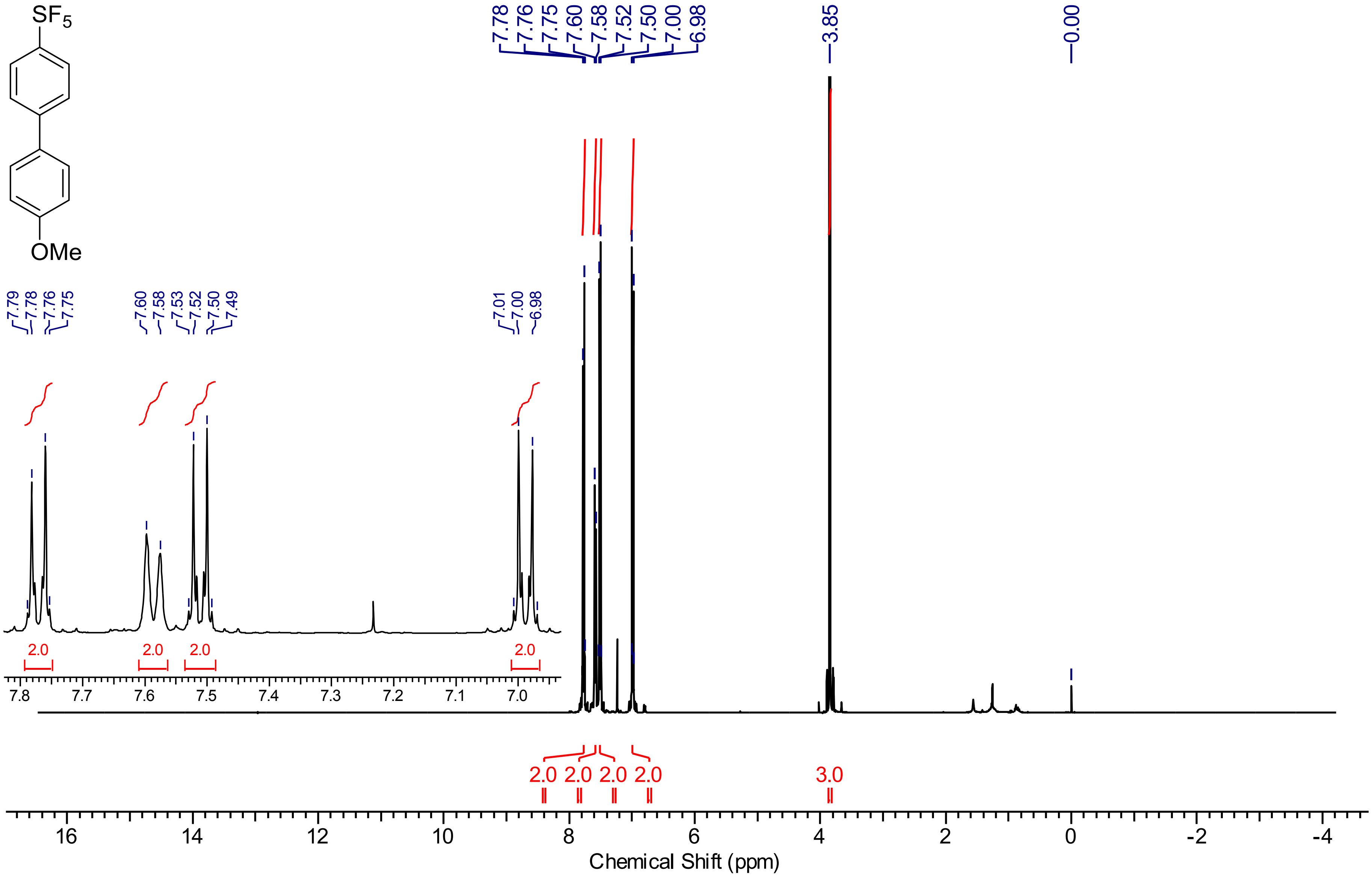

**7bd**: $^{13}C$ NMR (101 MHz, $CDCl_3$)

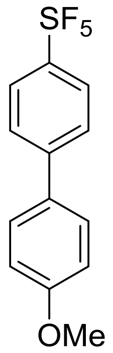


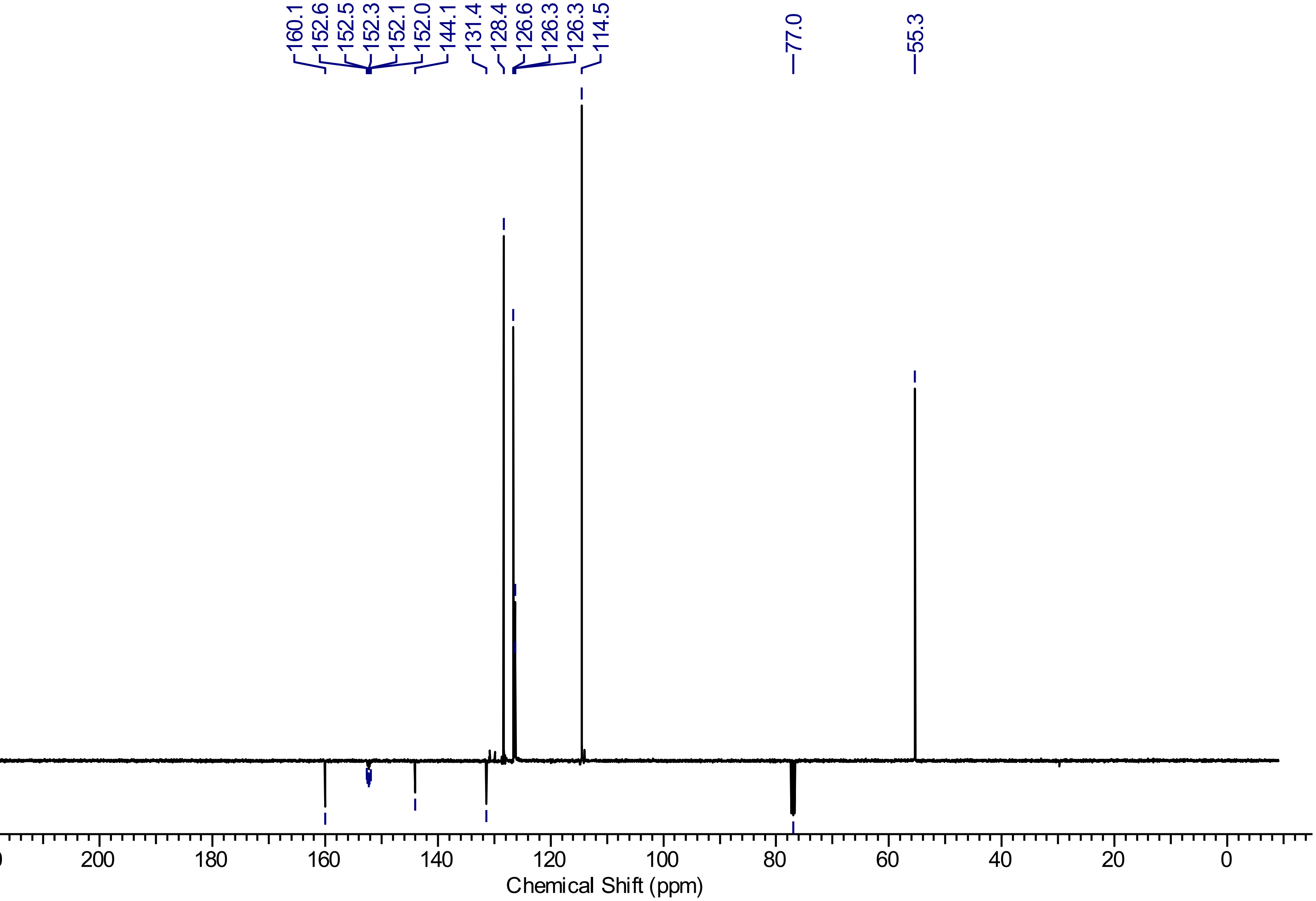

**7bd**: $^{19}$F NMR (377 MHz, $CDCl_3$)

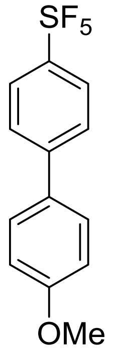


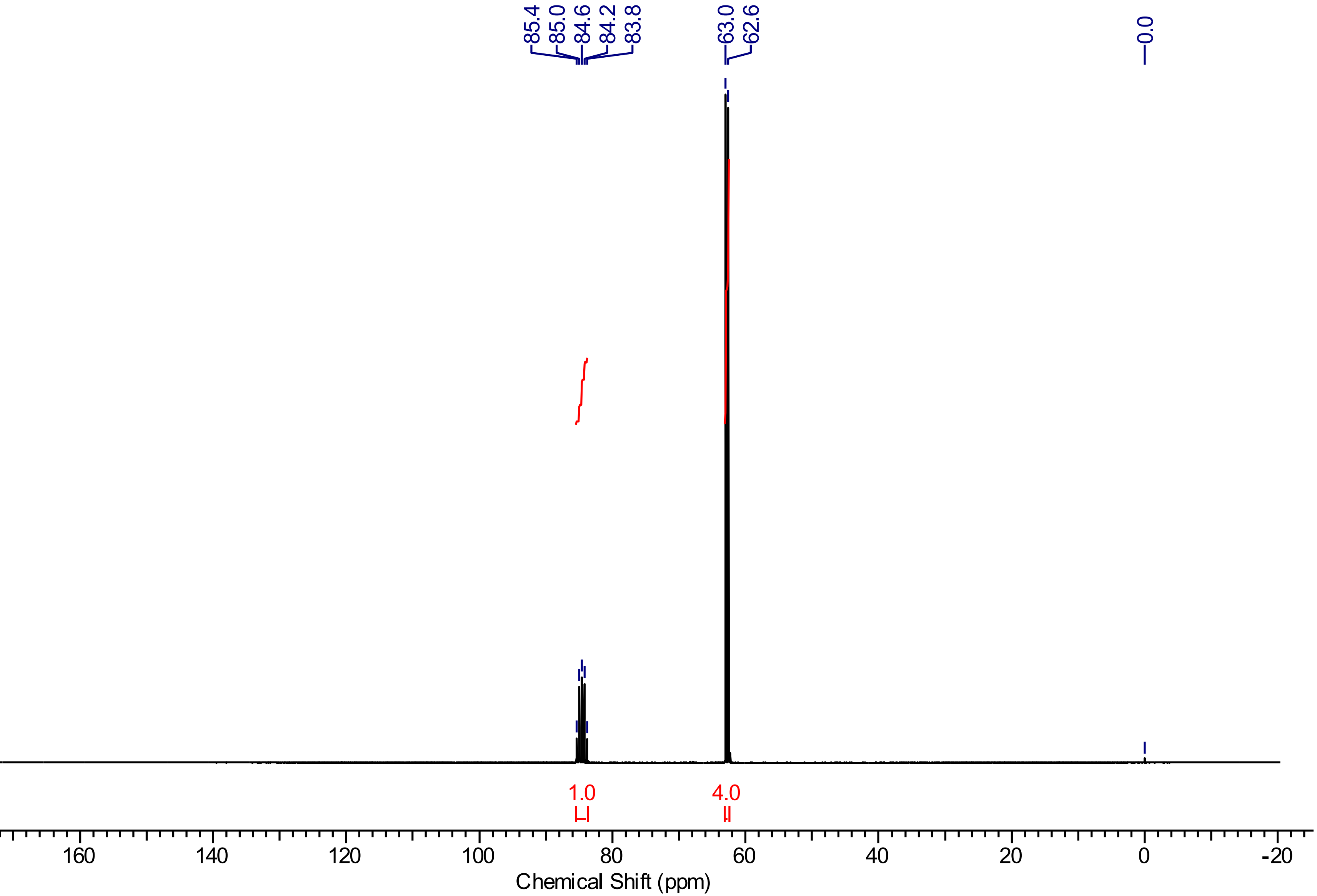

**7bh**: $^1$H NMR (400 MHz, $CDCl_3$)

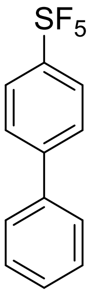


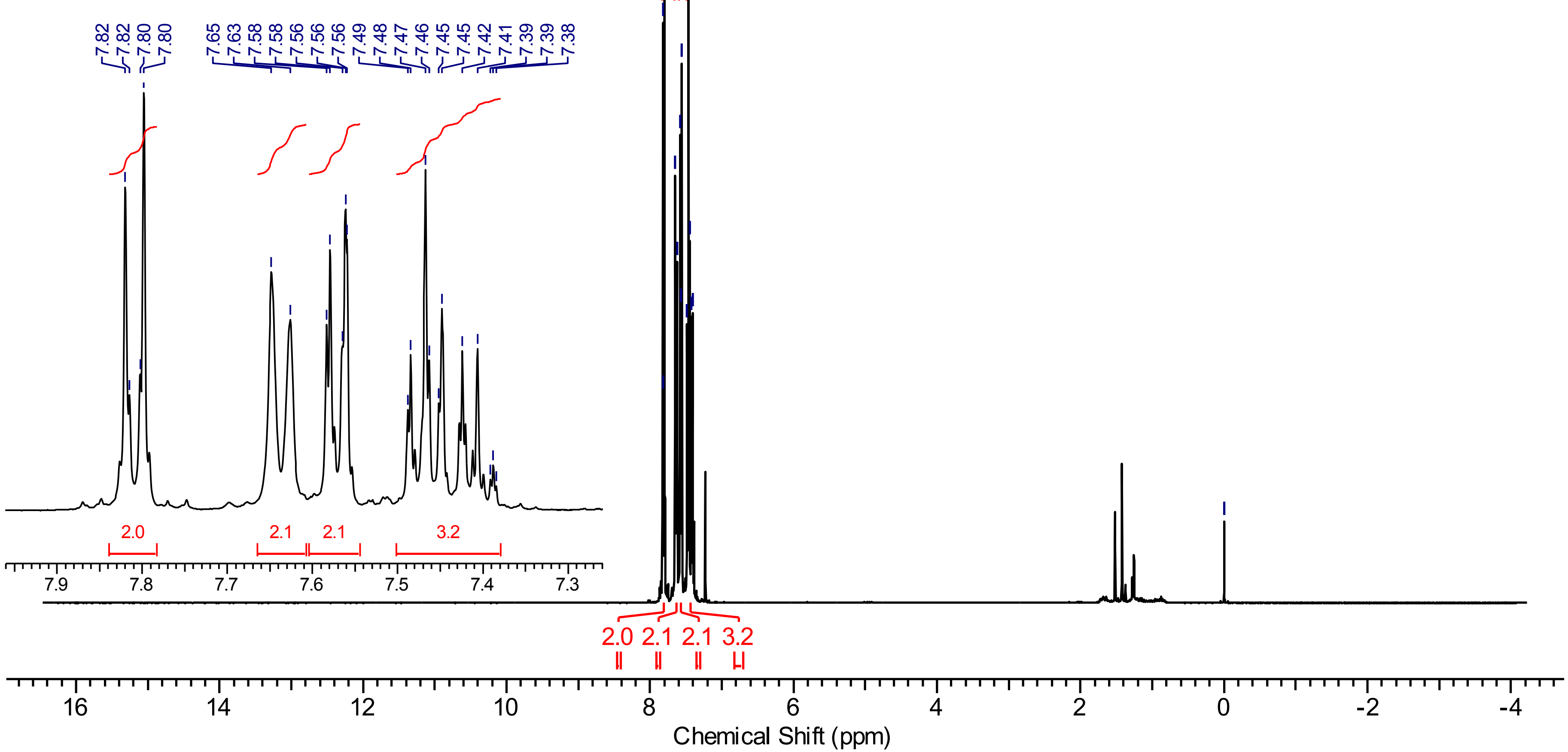

**7bh**: $^{13}$C NMR (101 MHz, $CDCl_3$)

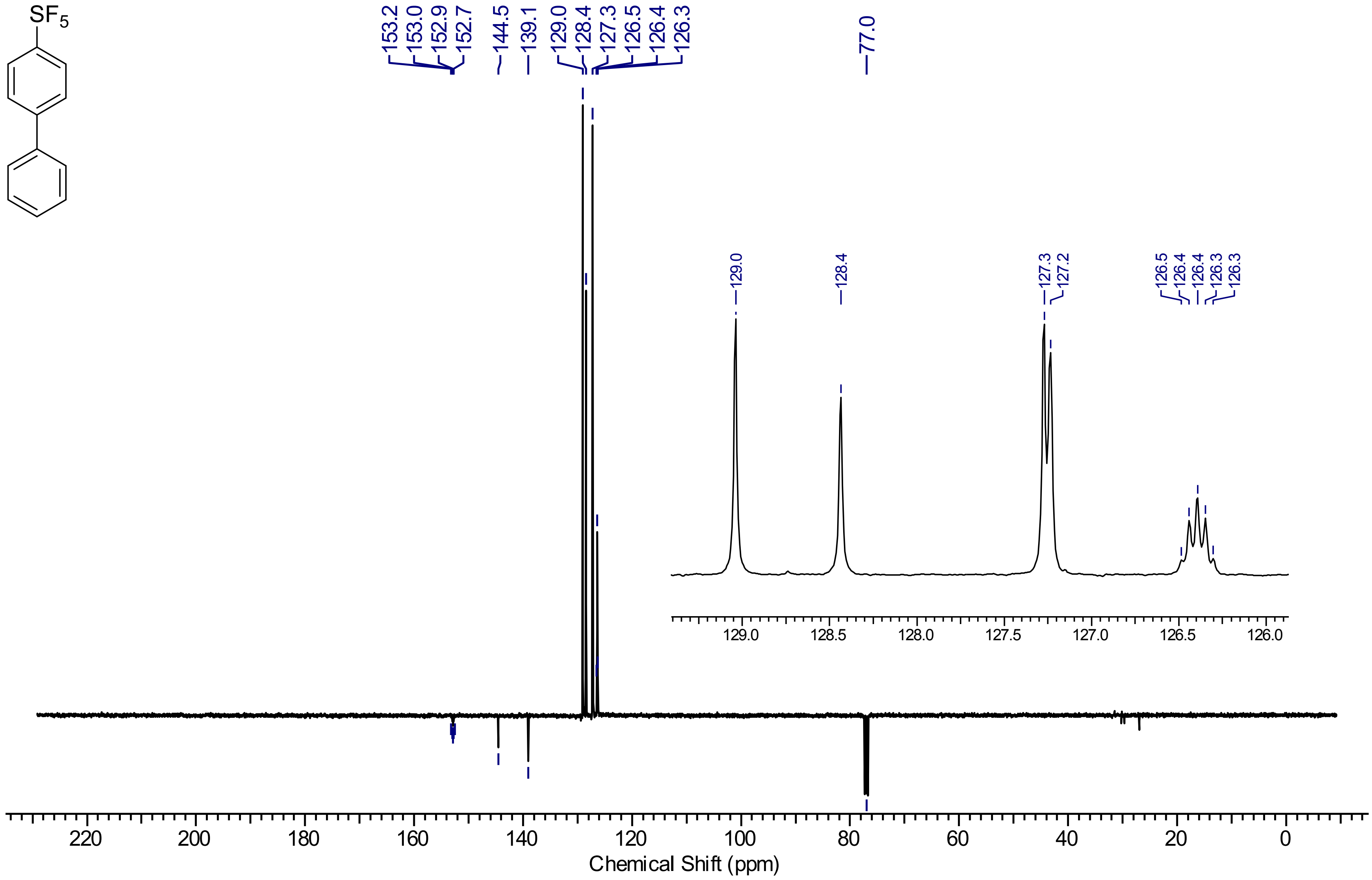

**7bh**: $^{19}F$ NMR (377 MHz, $CDCl_3$)

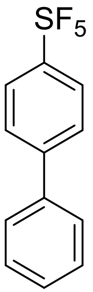

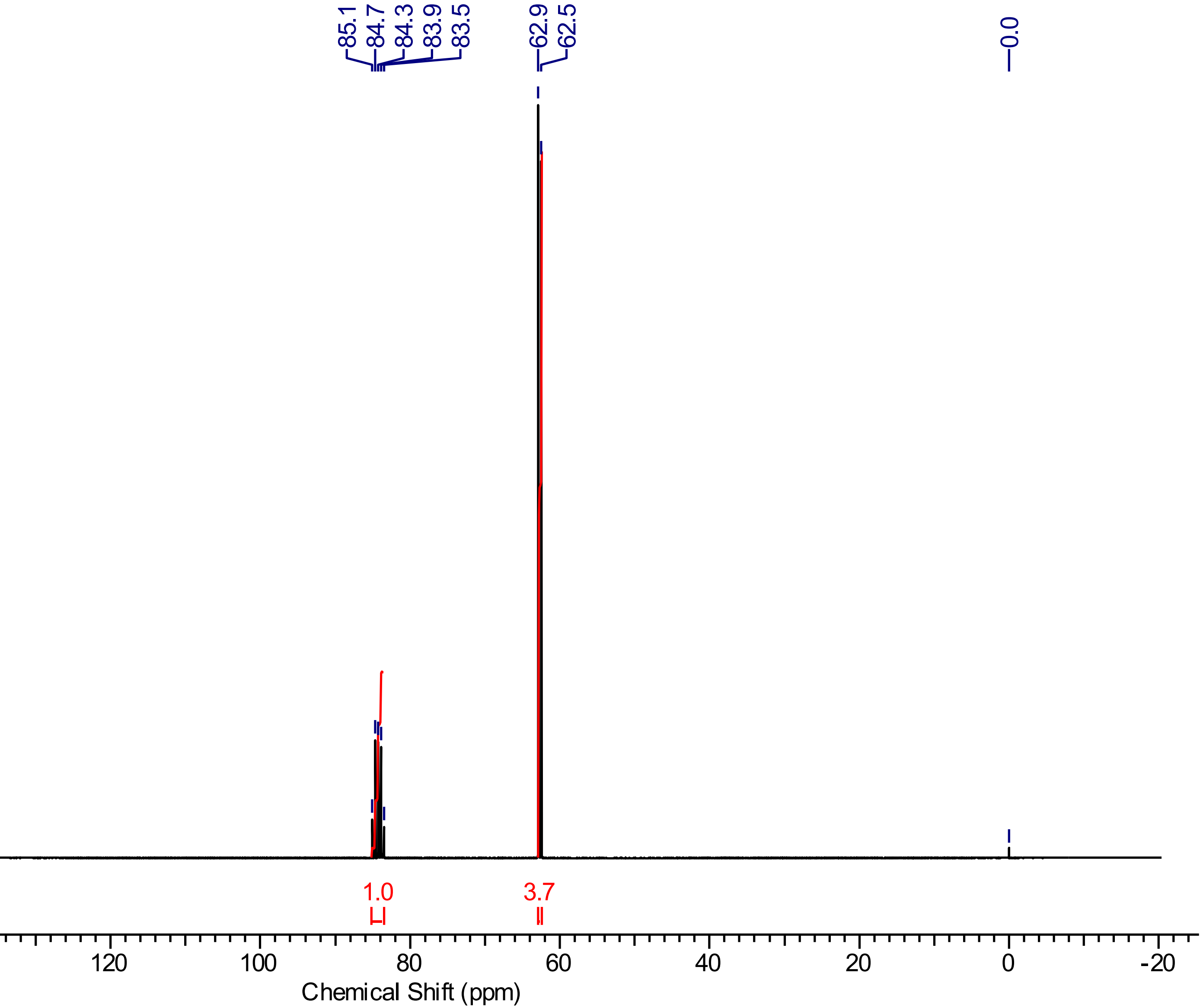

**8b** + **8b anhydride**: $^{1}$H NMR (400 MHz, [D$_6$]DMSO)

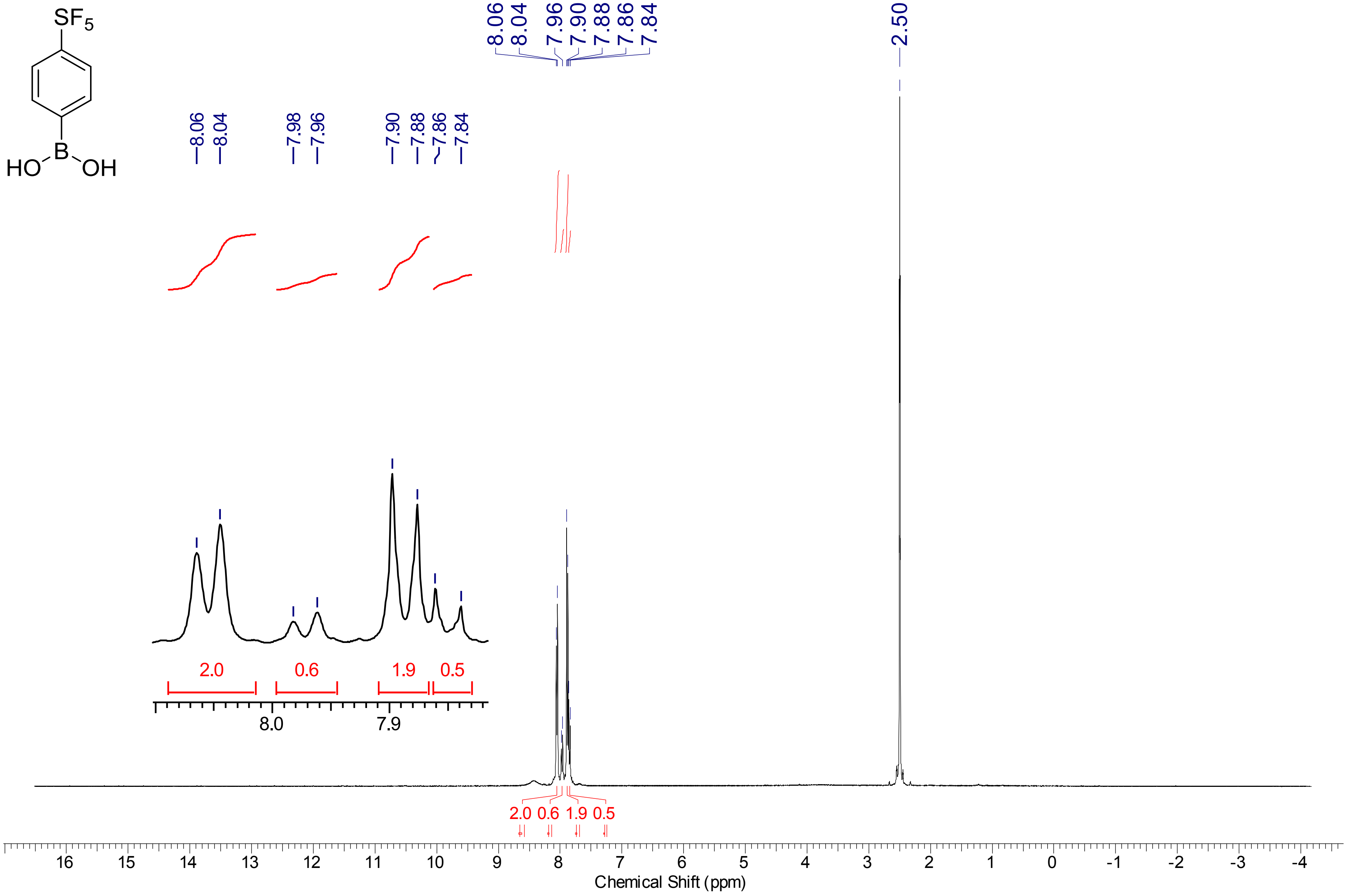

**8b**: $^{11}B$ NMR (128 MHz, [D$_6$]DMSO)

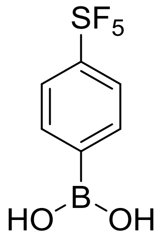


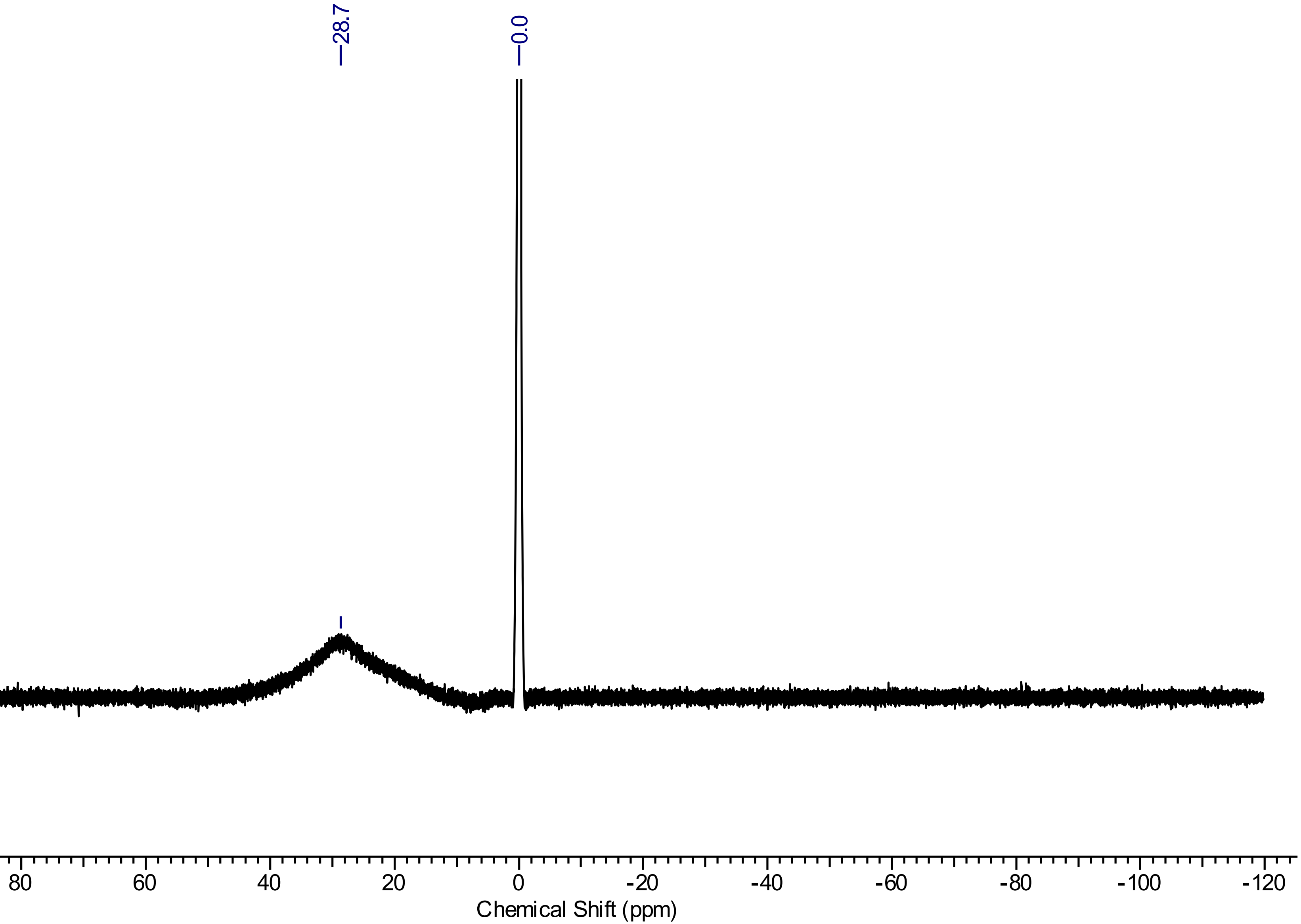

**8b**: $^{13}$C NMR (101 MHz, [D$_6$]DMSO)

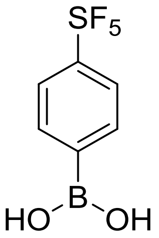


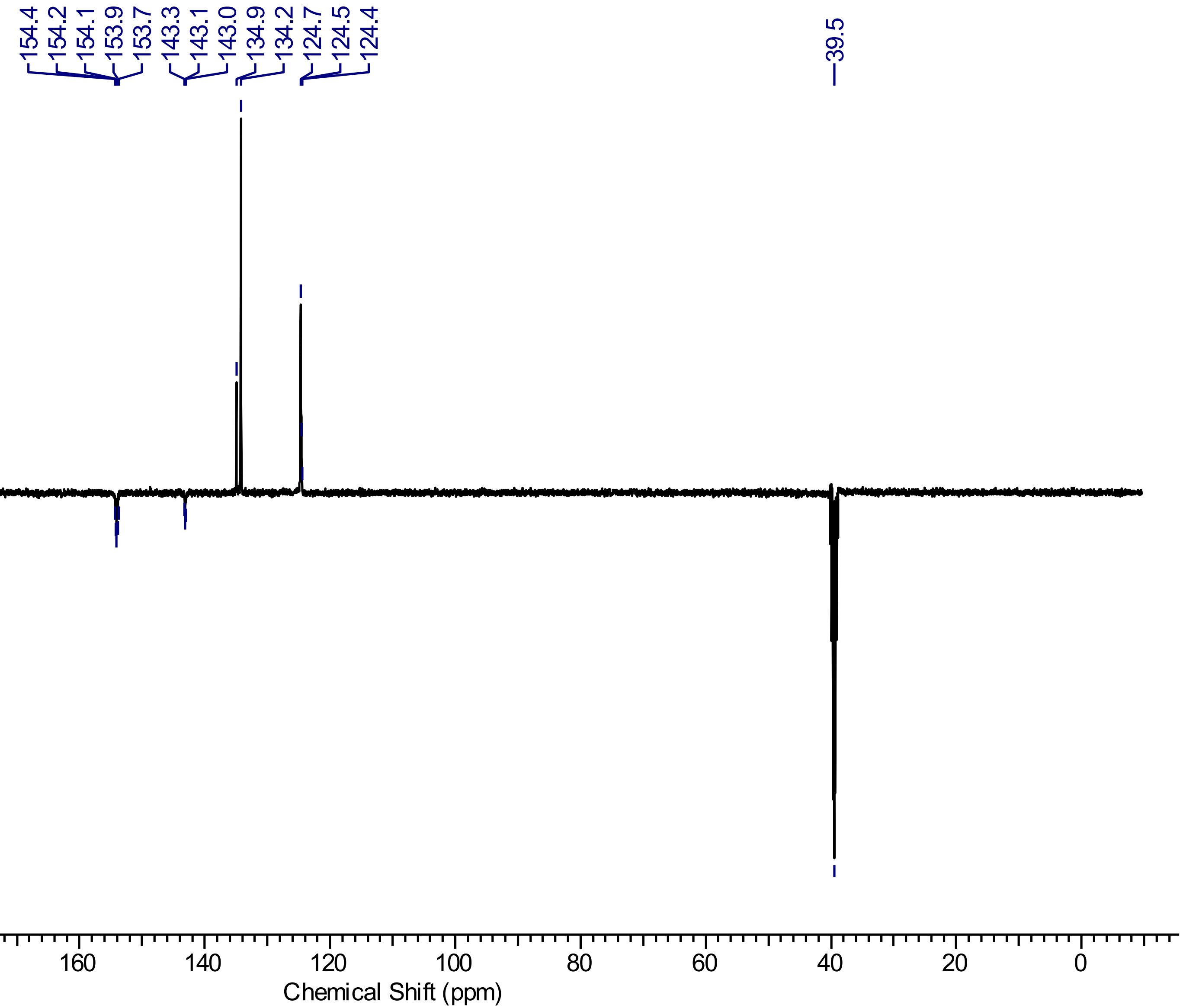

**8b**: $^{19}$F NMR (377 MHz, $CD_3CN$)

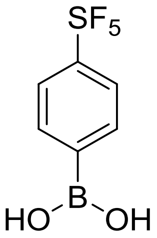


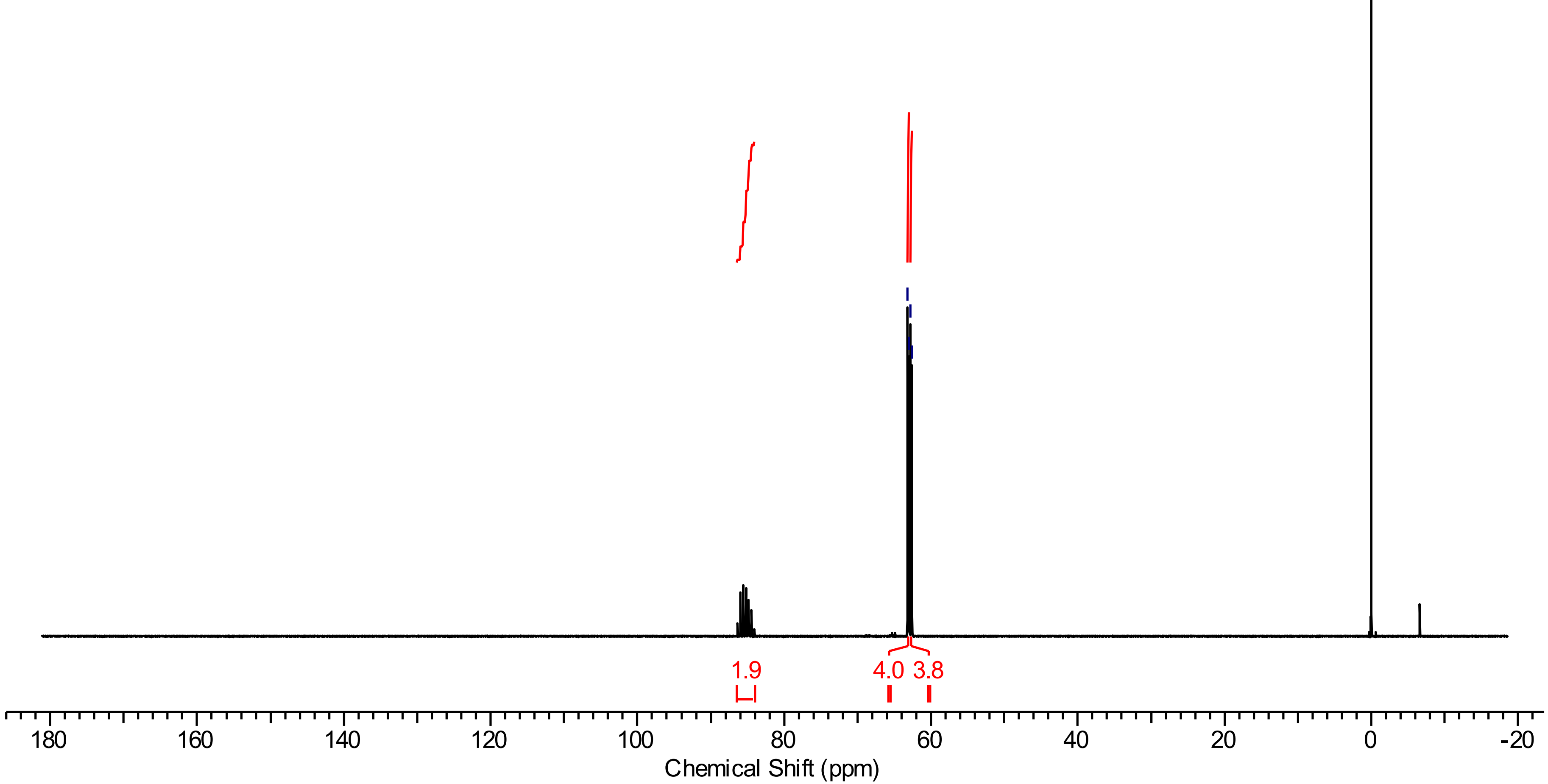

**9a**: $^1$H NMR (400 MHz, $[D_6]$acetone)

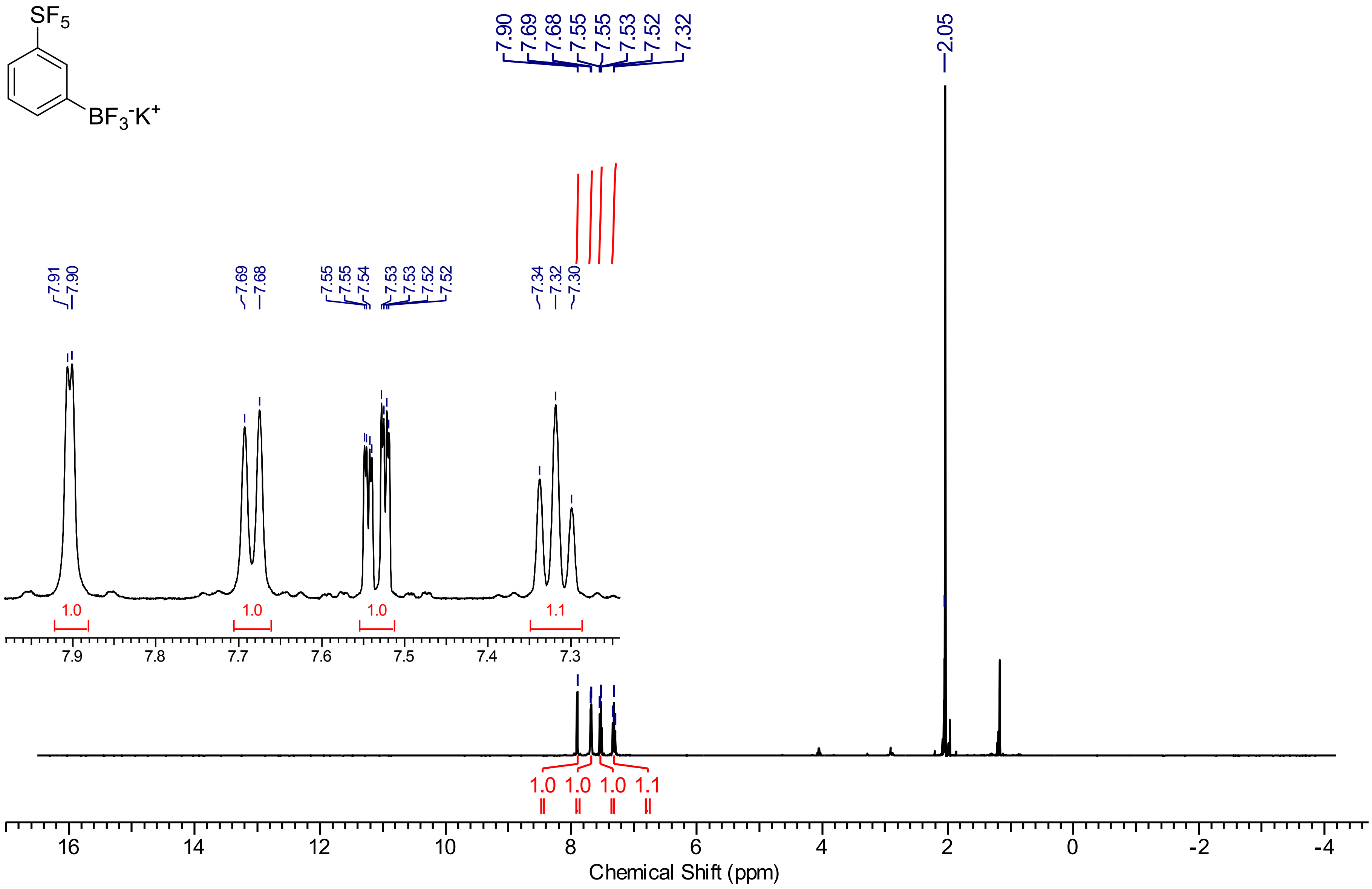

**9a**: $^{11}$B NMR (128 MHz, [D$_6$]acetone)

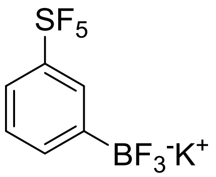

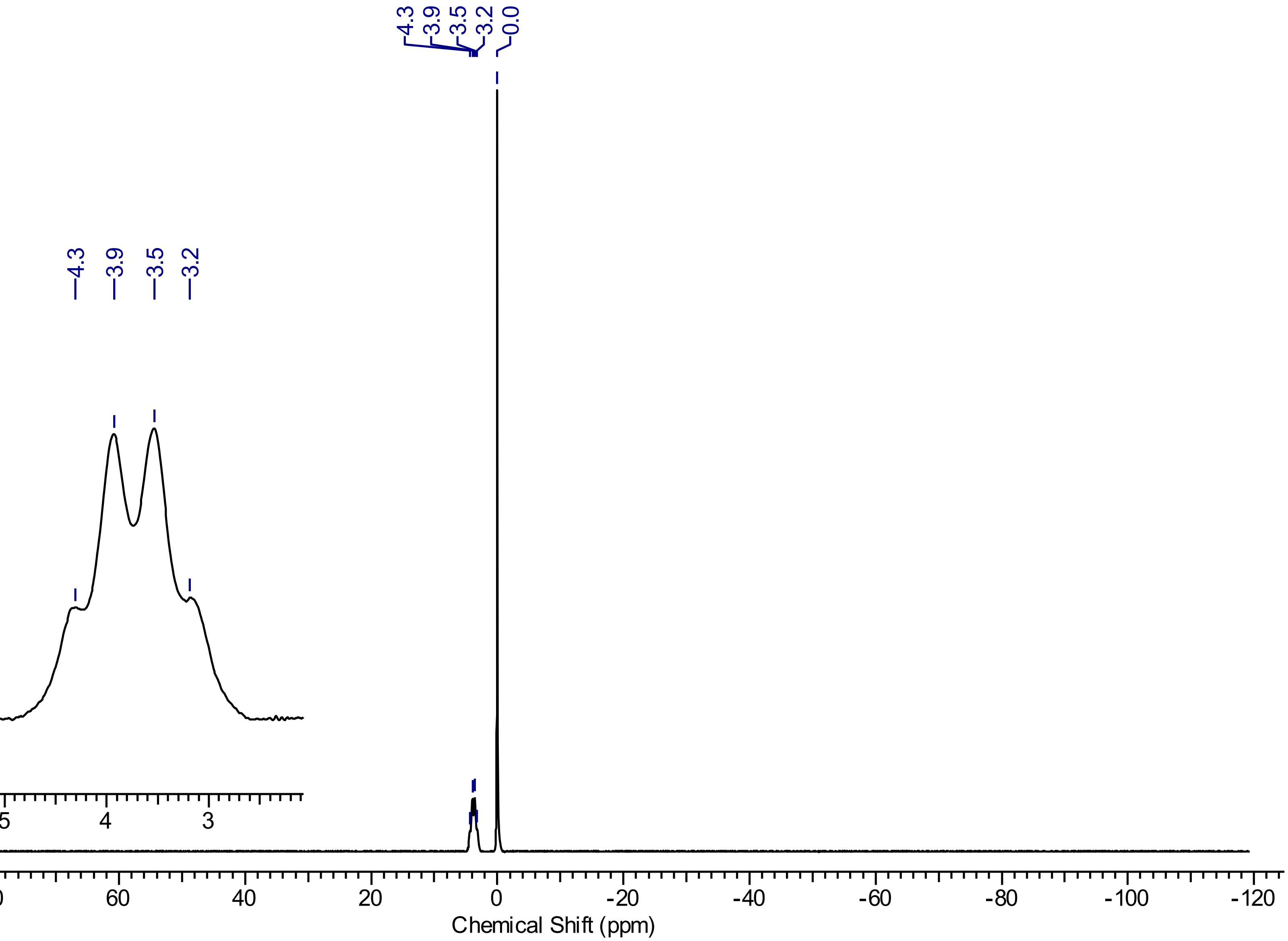

**9a**: $^{13}C$ NMR (101 MHz, [$D_6$]acetone)

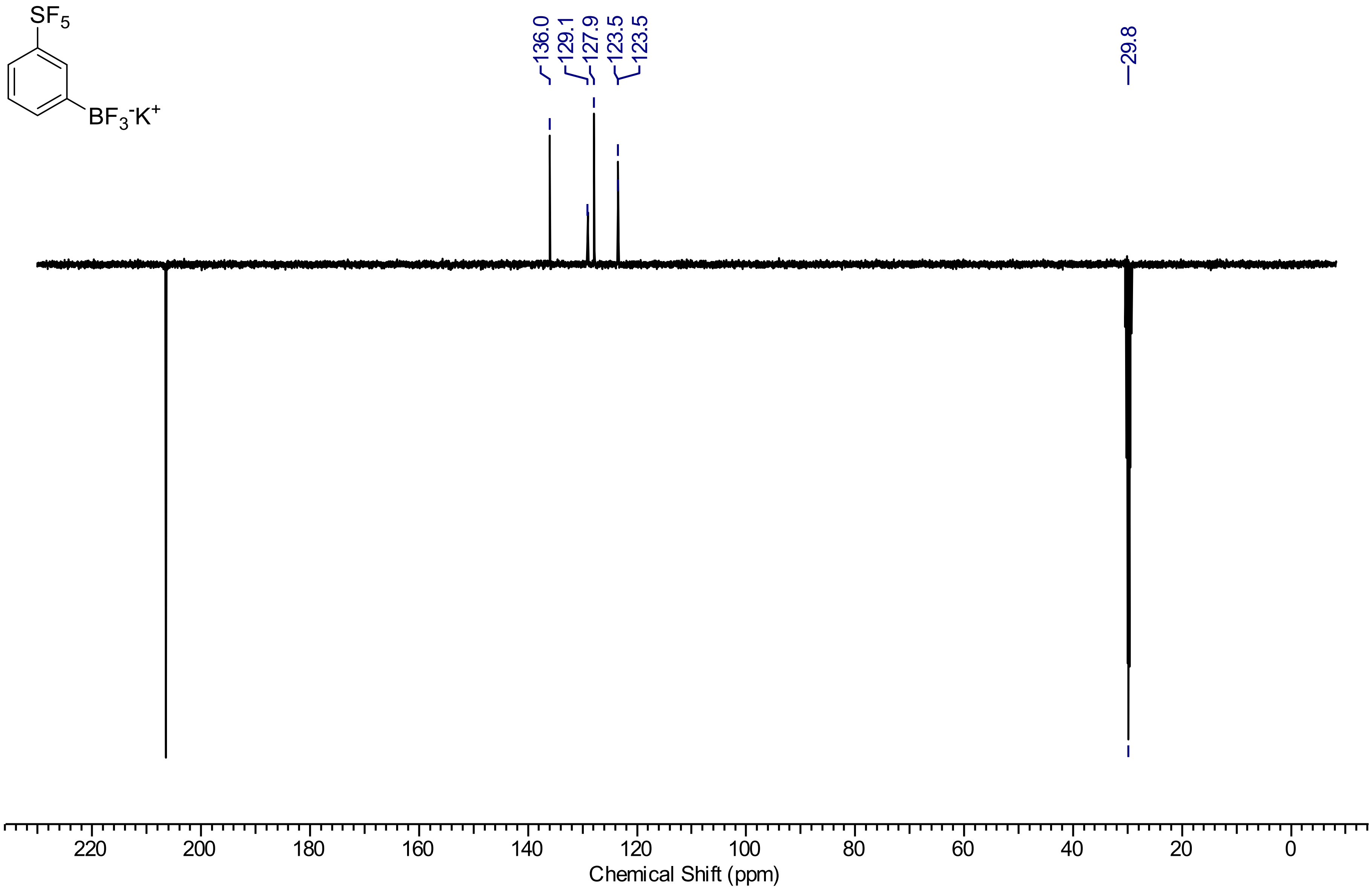

**9a**: $^{19}F$ NMR (377 MHz, [$D_6$]acetone)

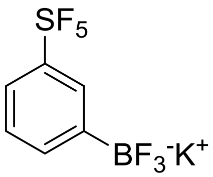

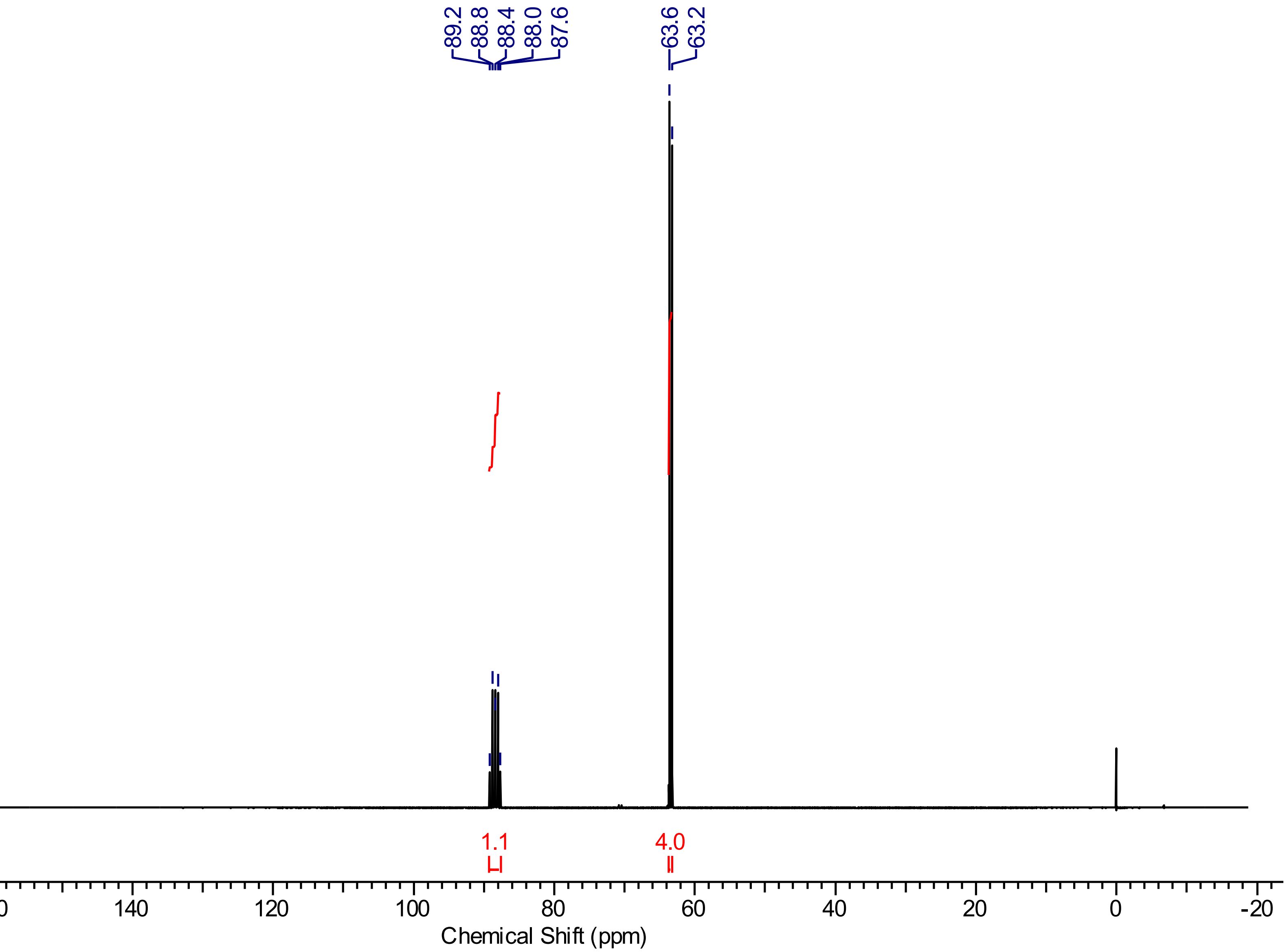

**9b**: $^{1}H$ NMR (400 MHz, [$D_6$]acetone)

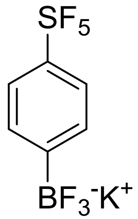


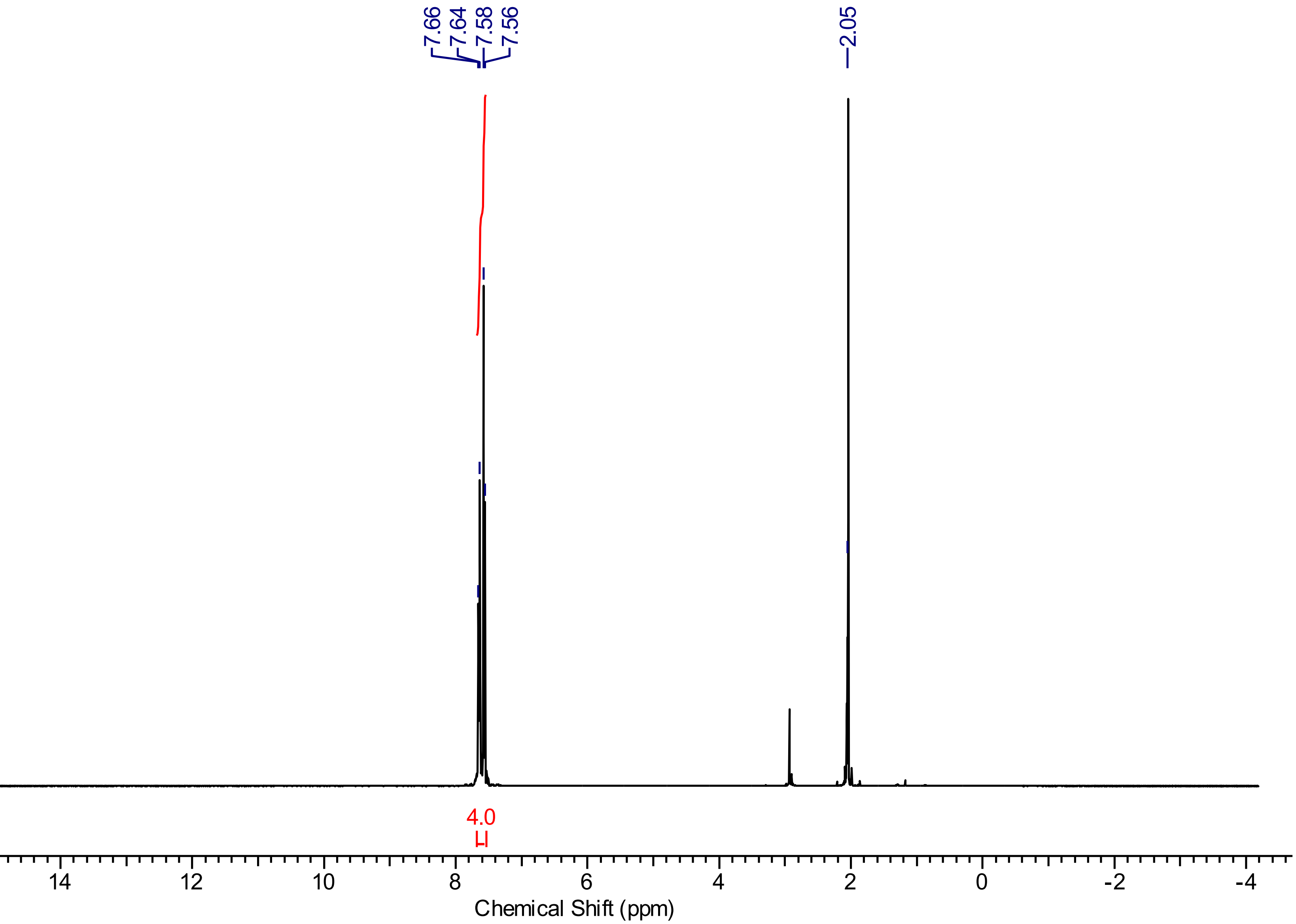

**9b**: $^{11}$B NMR (128 MHz, [D$_6$]acetone)

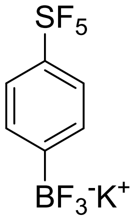


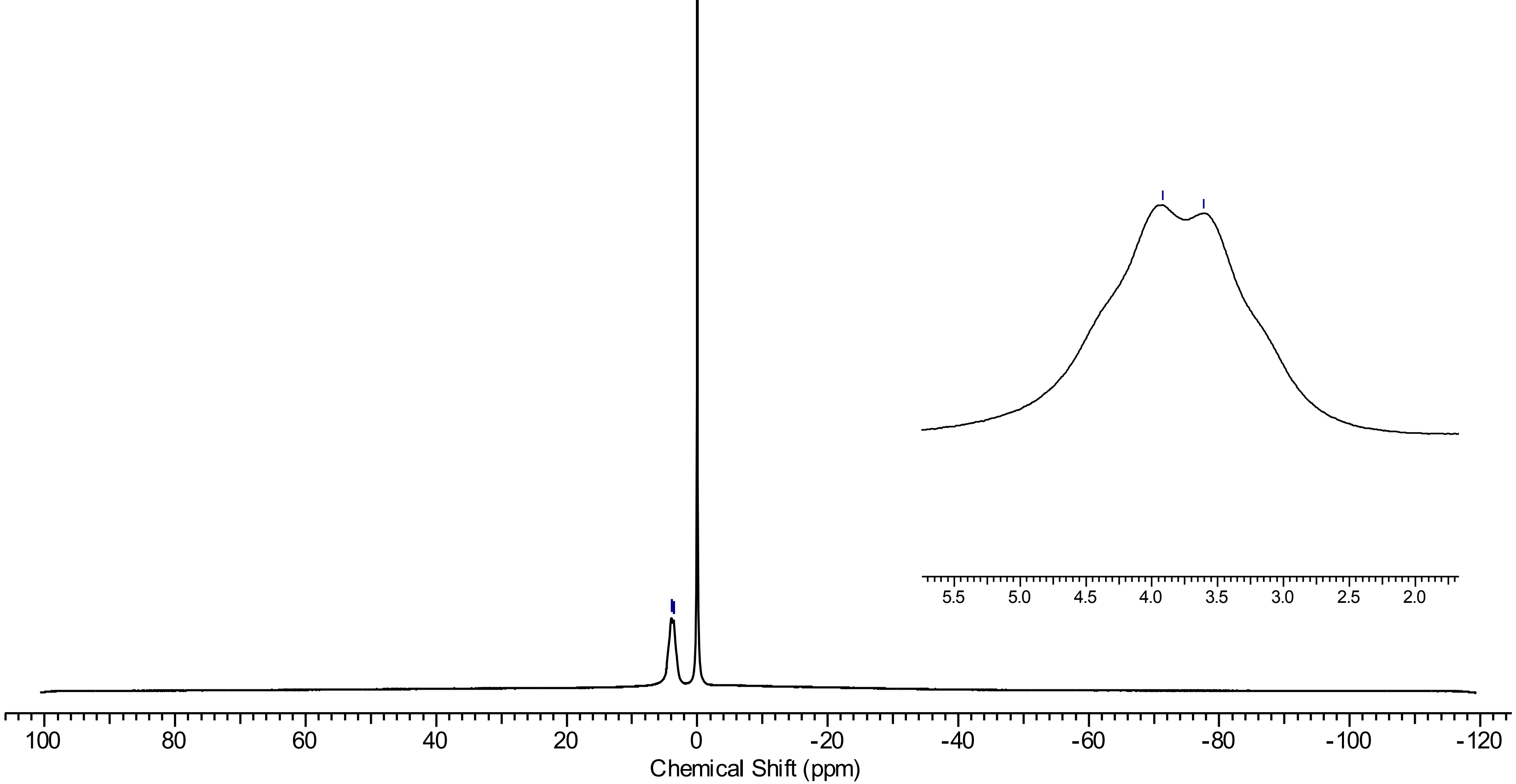

**9b**: $^{13}C$ NMR (101 MHz, $[D_6]$acetone)

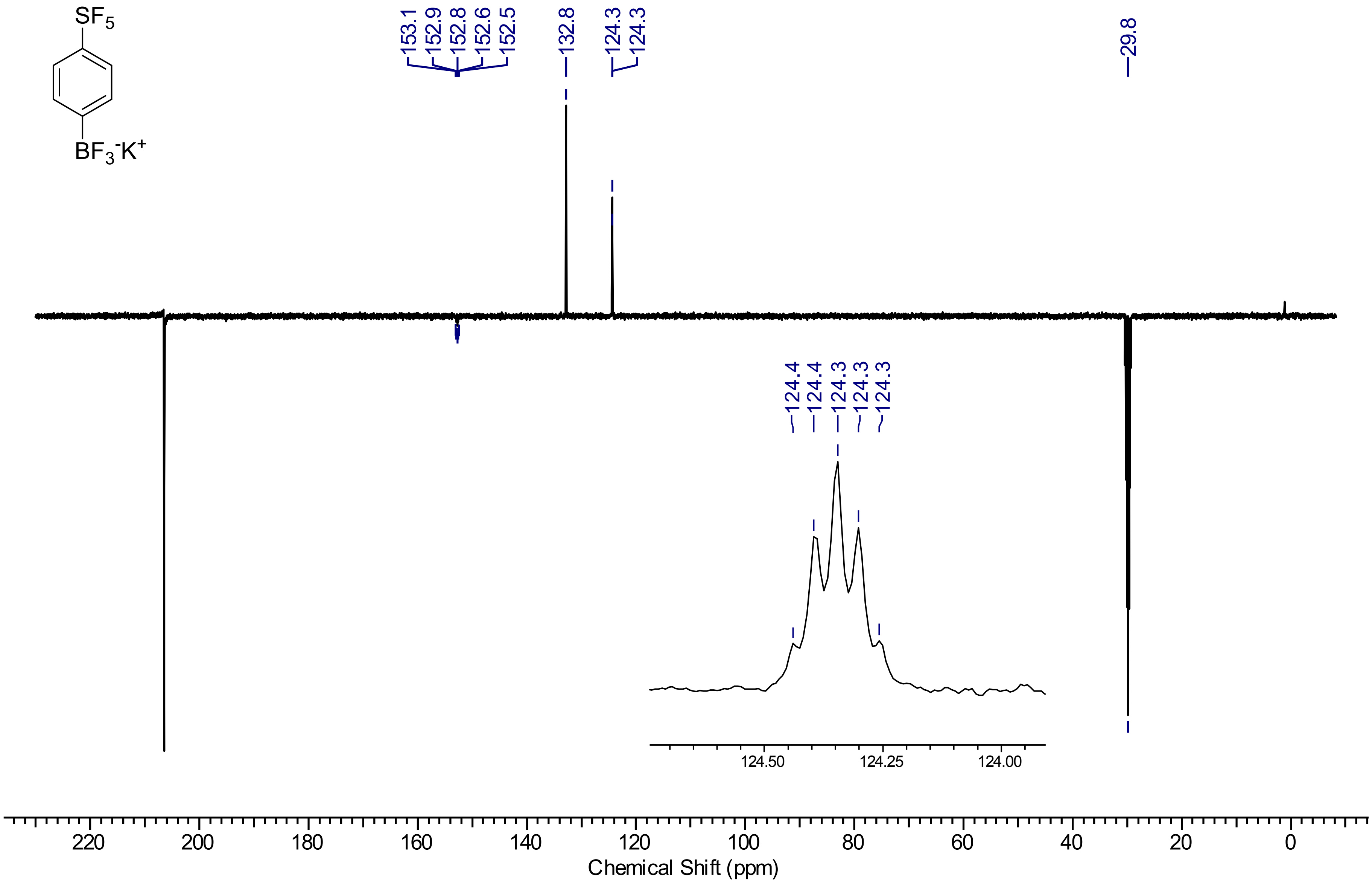

**9b**: $^{19}$F NMR (377 MHz, [D$_6$]acetone)

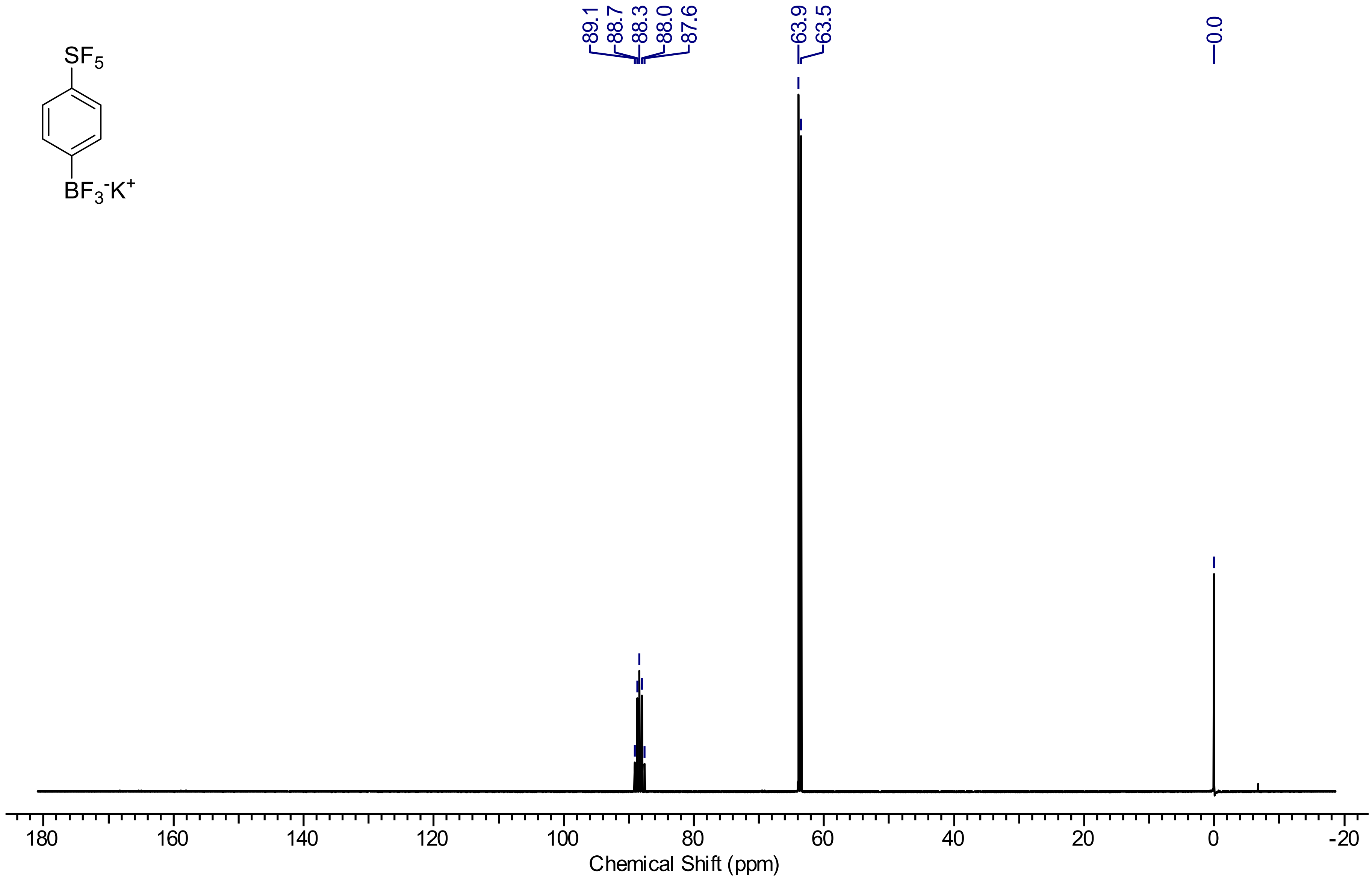

**9b**: $^{19}F$ NMR (376 MHz, [D$_6$]acetone)

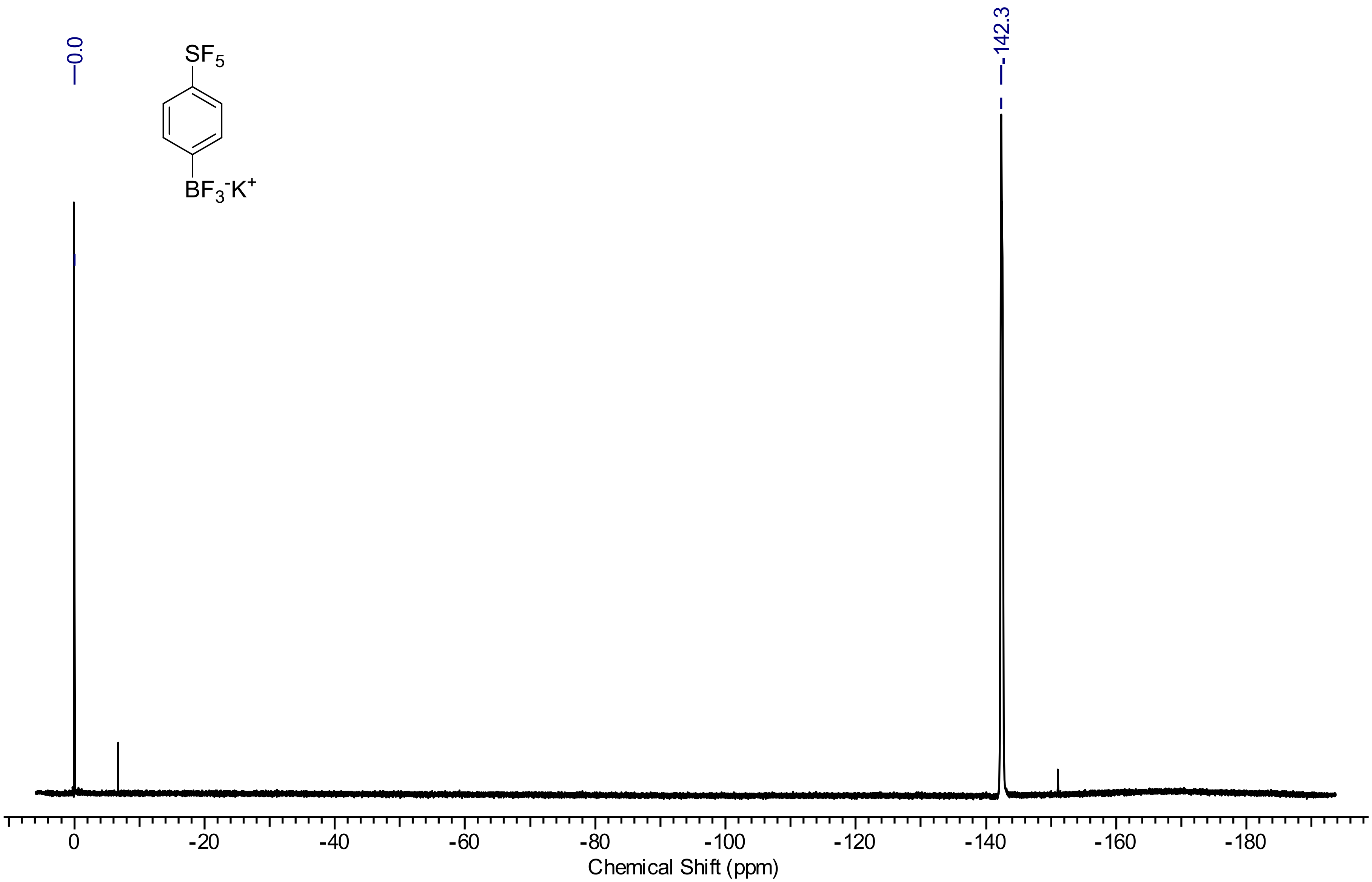

**Bis(pyridine)iodonium tetrafluoroborate**: $^{1}H$ NMR (400 MHz, $CD_2Cl_2$)

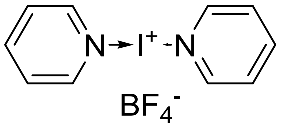

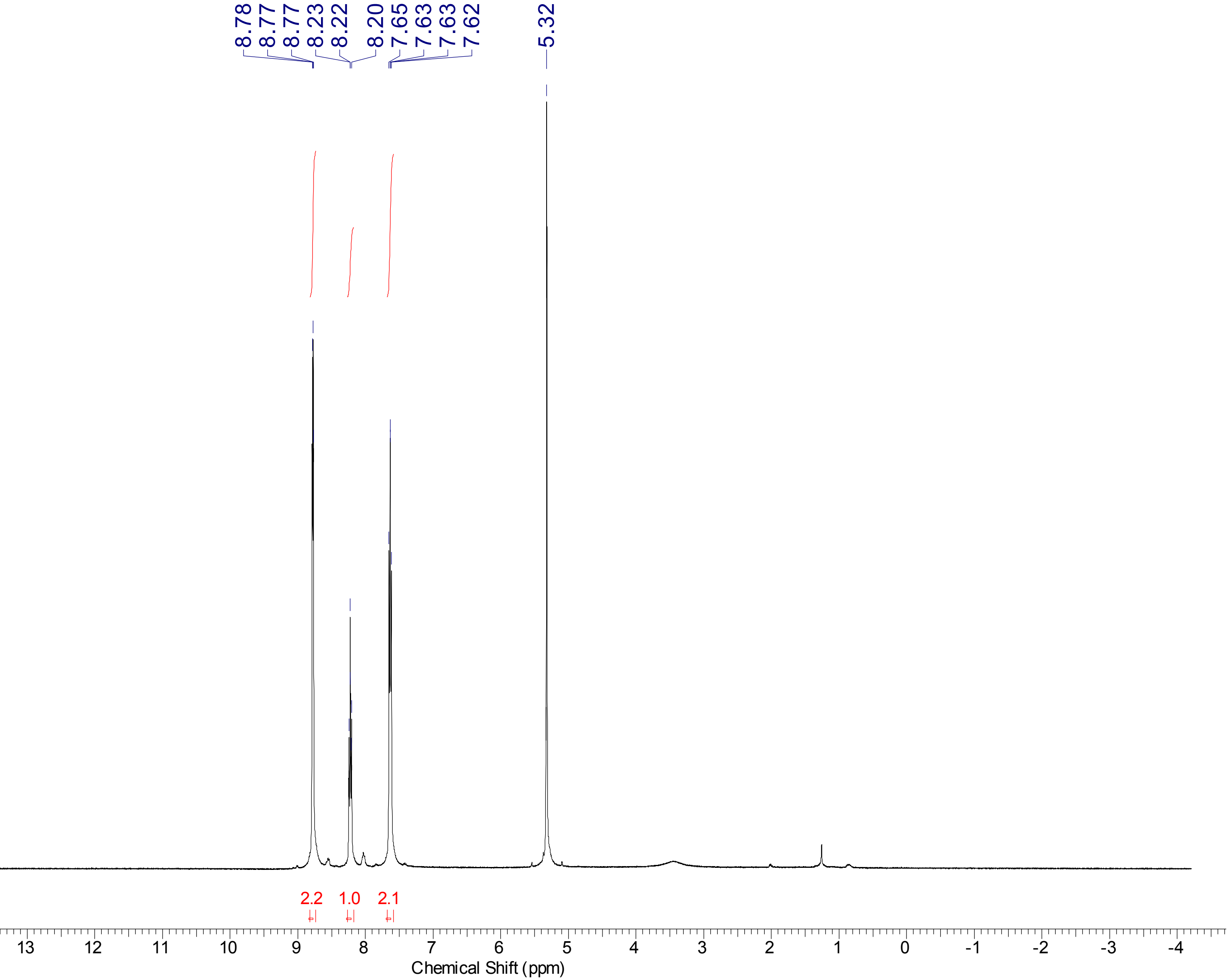

**Bis(pyridine)iodonium tetrafluoroborate**: $^{11}B$ NMR (128 MHz, $CD_2Cl_2$)

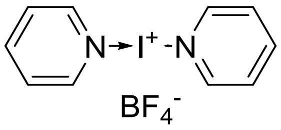

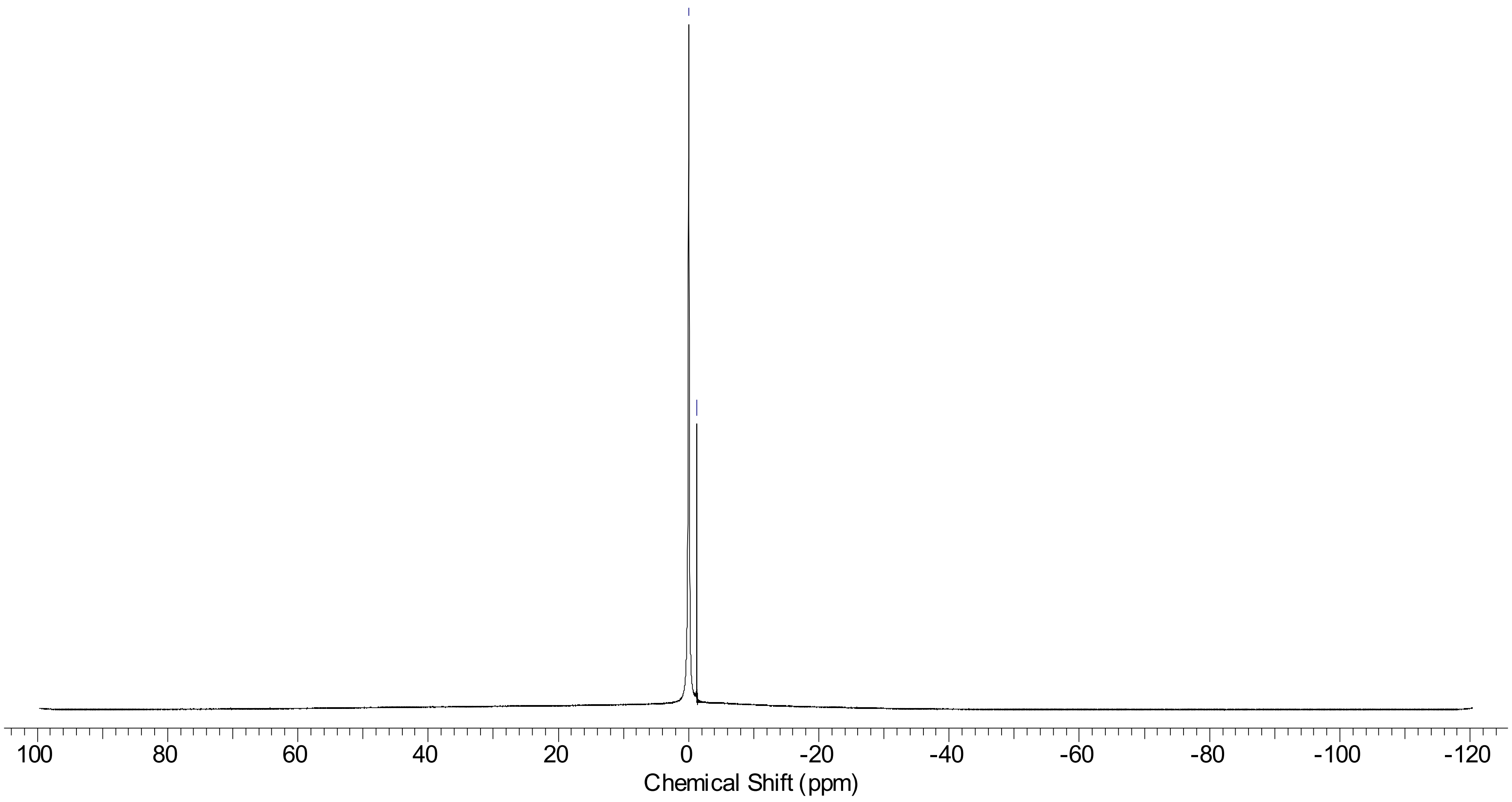

**Bis(pyridine)iodonium tetrafluoroborate**: $^{13}C$ NMR (101 MHz, $CD_2Cl_2$)

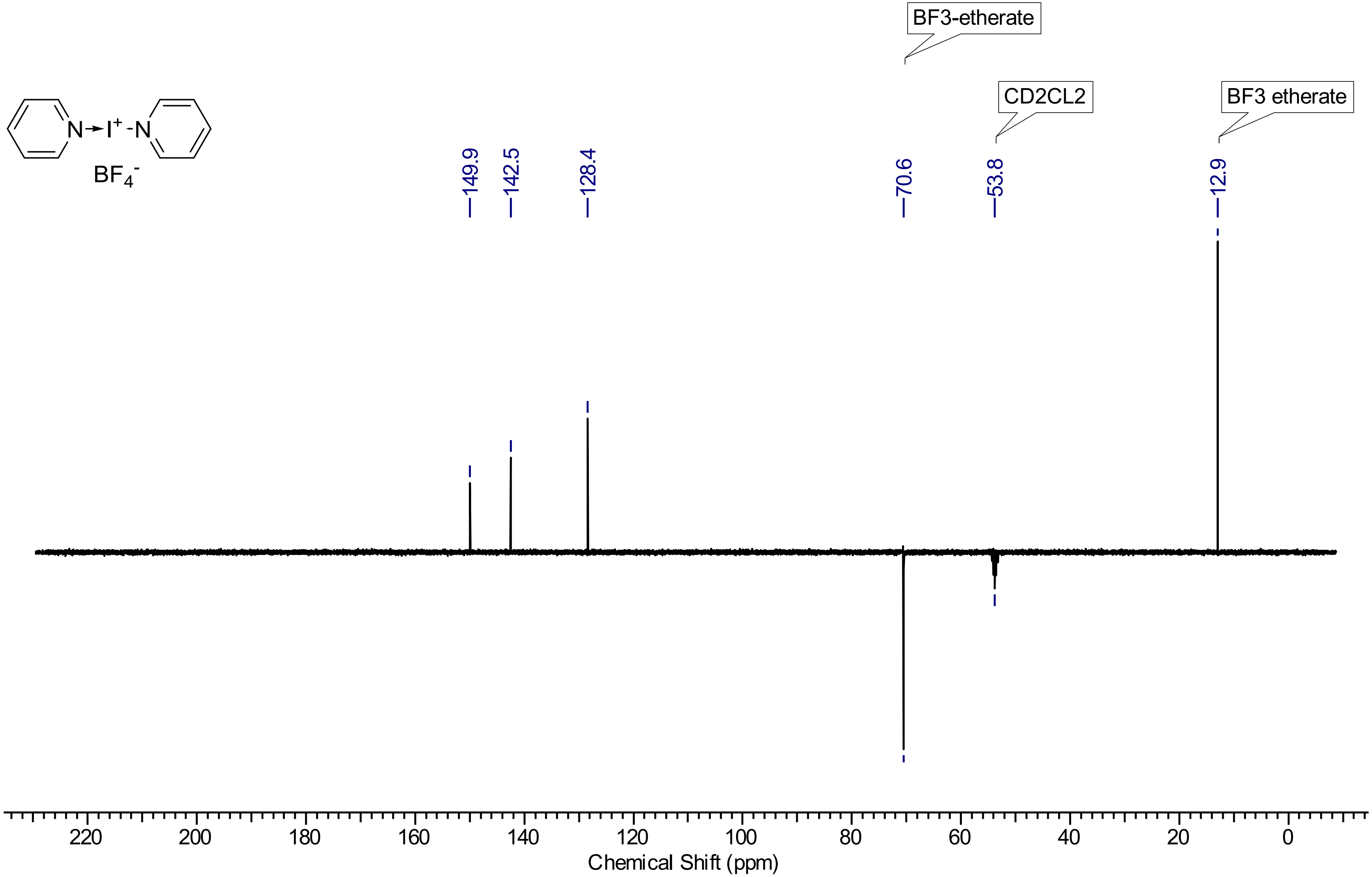

**Bis(pyridine)iodonium tetrafluoroborate**: $^{19}$F NMR (376 MHz, $CD_2Cl_2$)

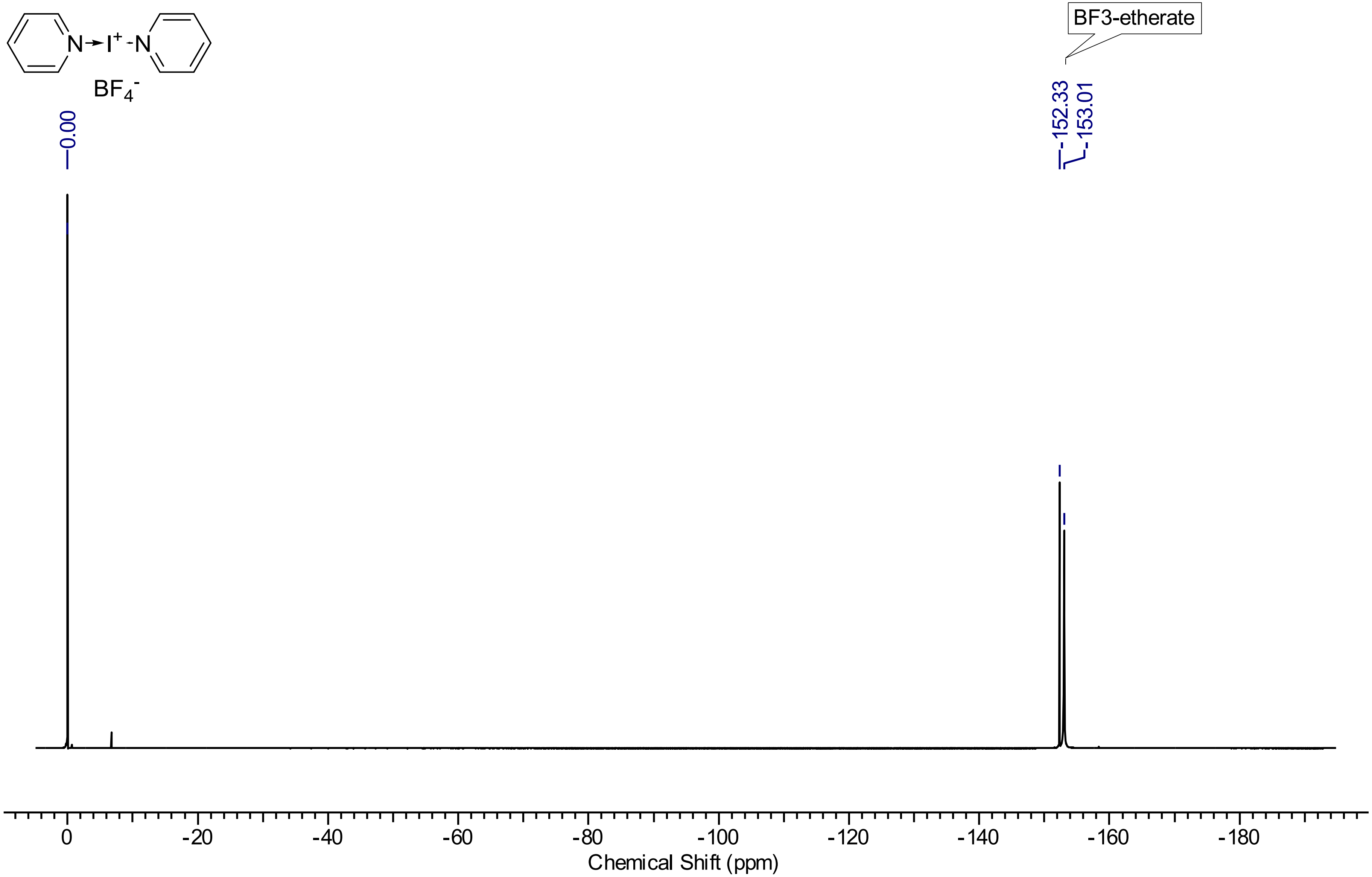

**10a**: $^1$H NMR (400 MHz, $CDCl_3$)

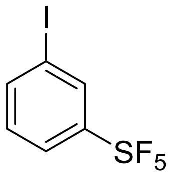

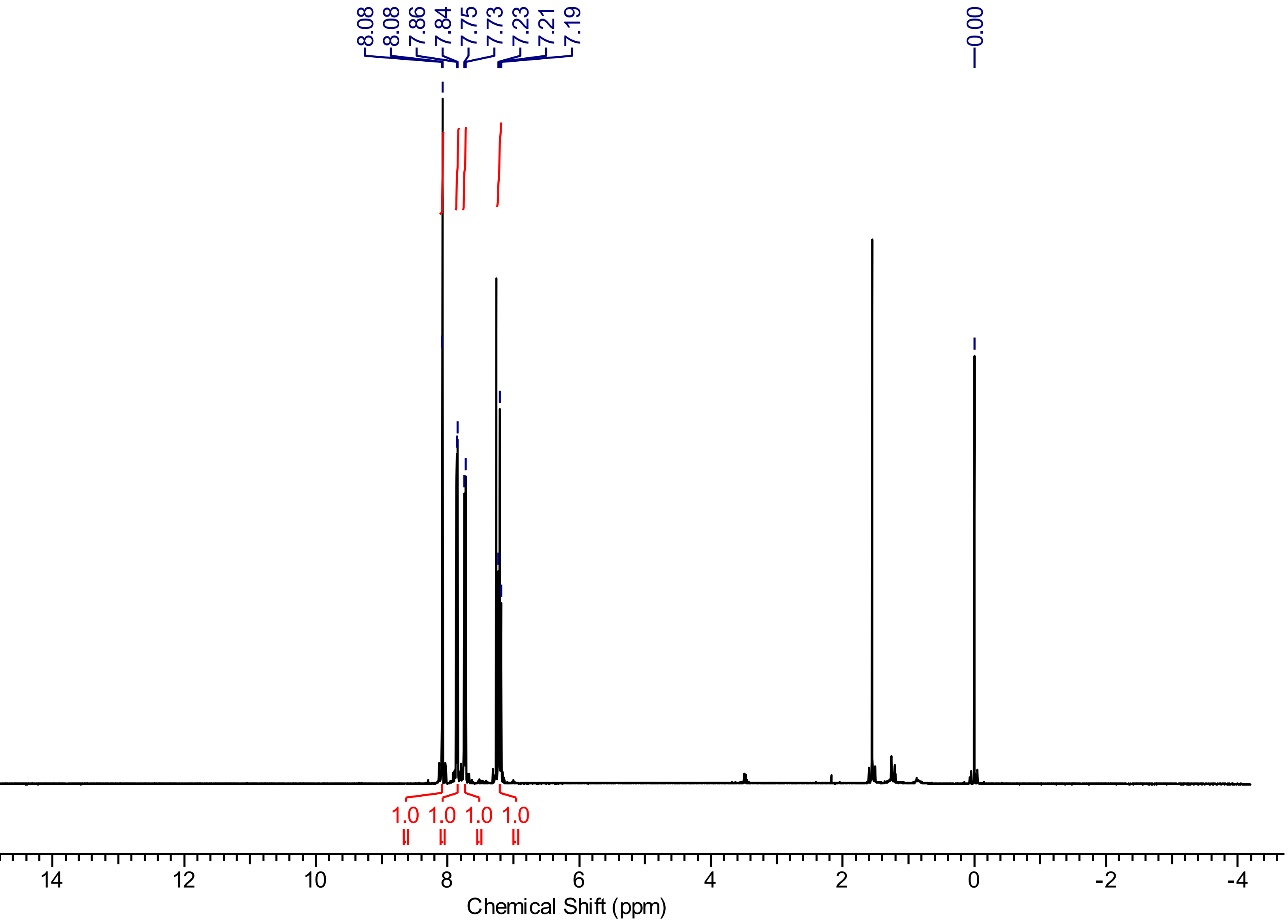

**10a**: $^{13}C$ NMR (101 MHz, $CDCl_3$)

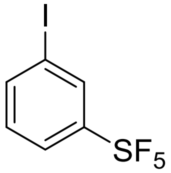


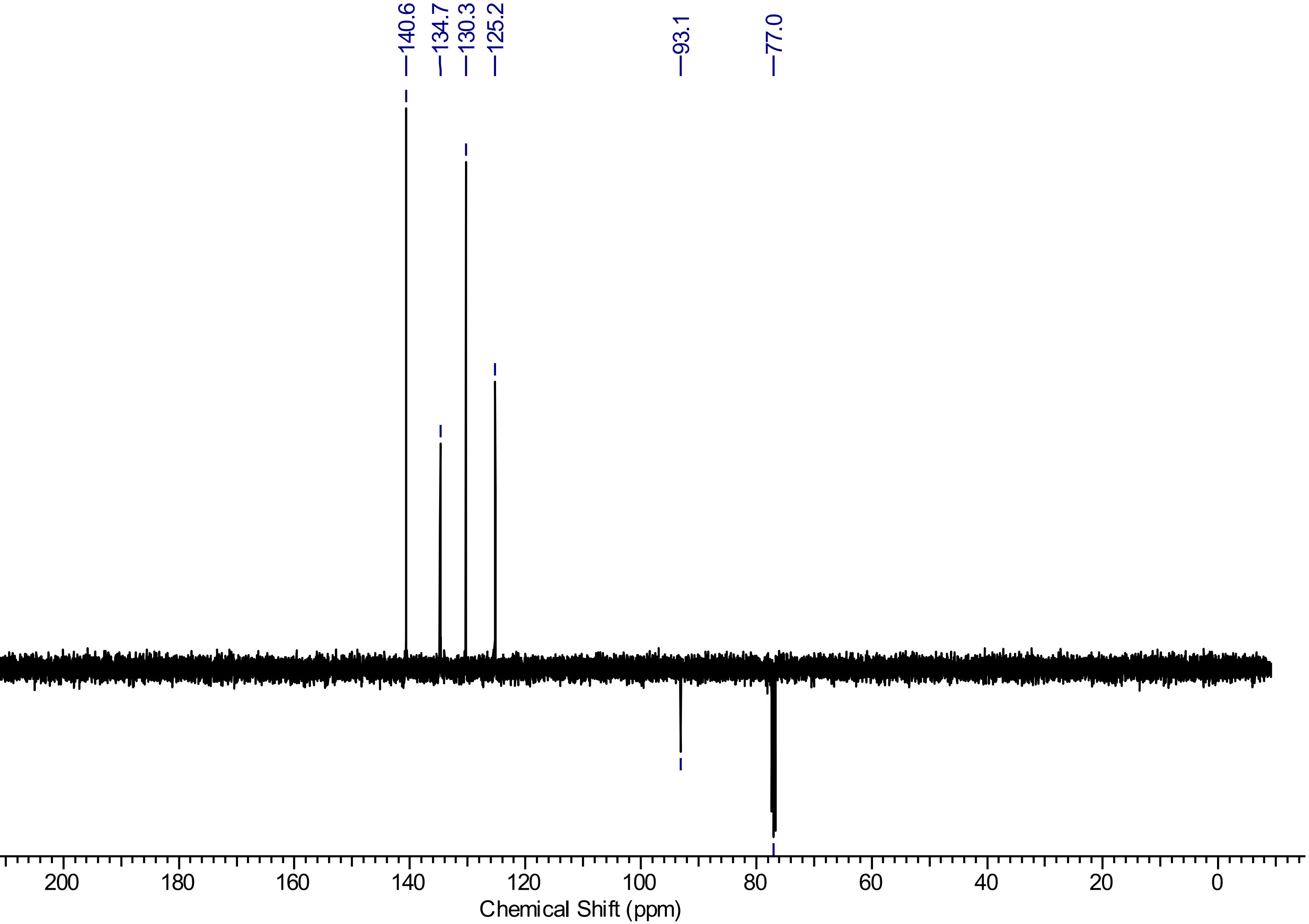

**10a**: $^{19}F$ NMR (377 MHz, $CDCl_3$)

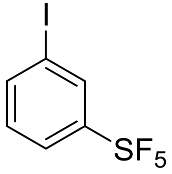


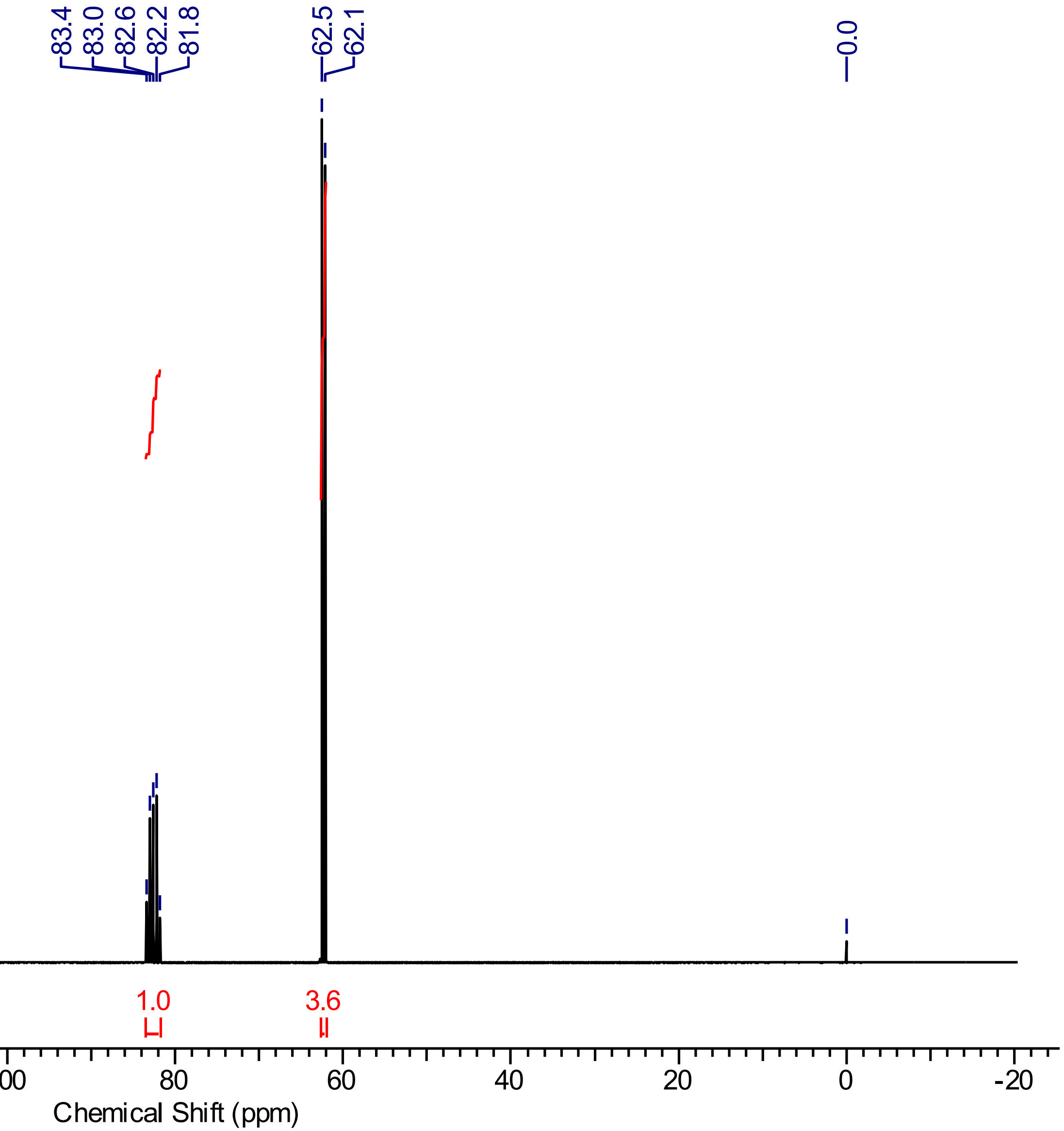

**10b**: $^{1}H$ NMR (400 MHz, $CDCl_3$)

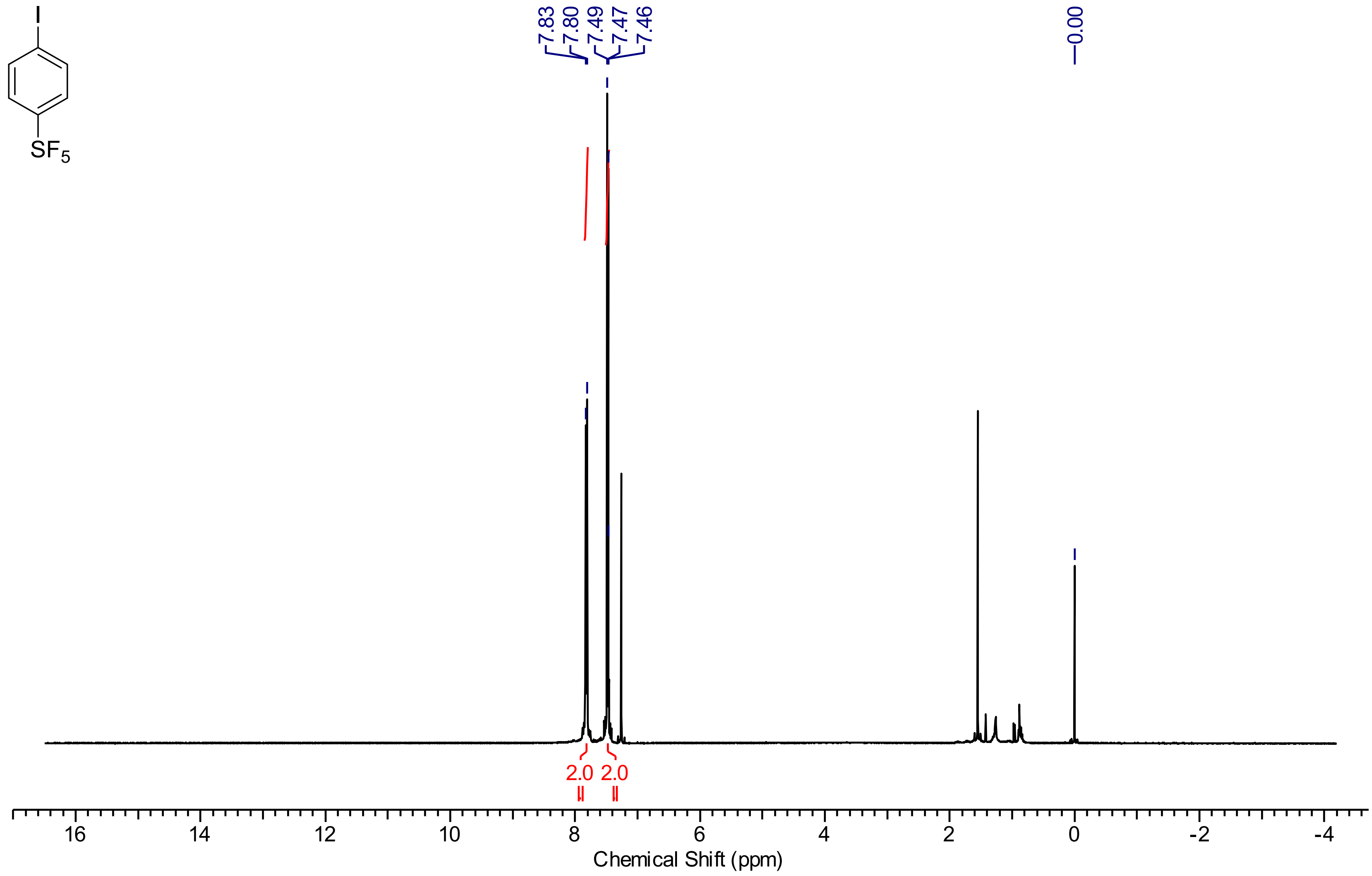

**10b**: $^{13}C$ NMR (101 MHz, $CDCl_3$)

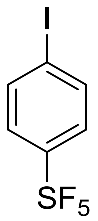


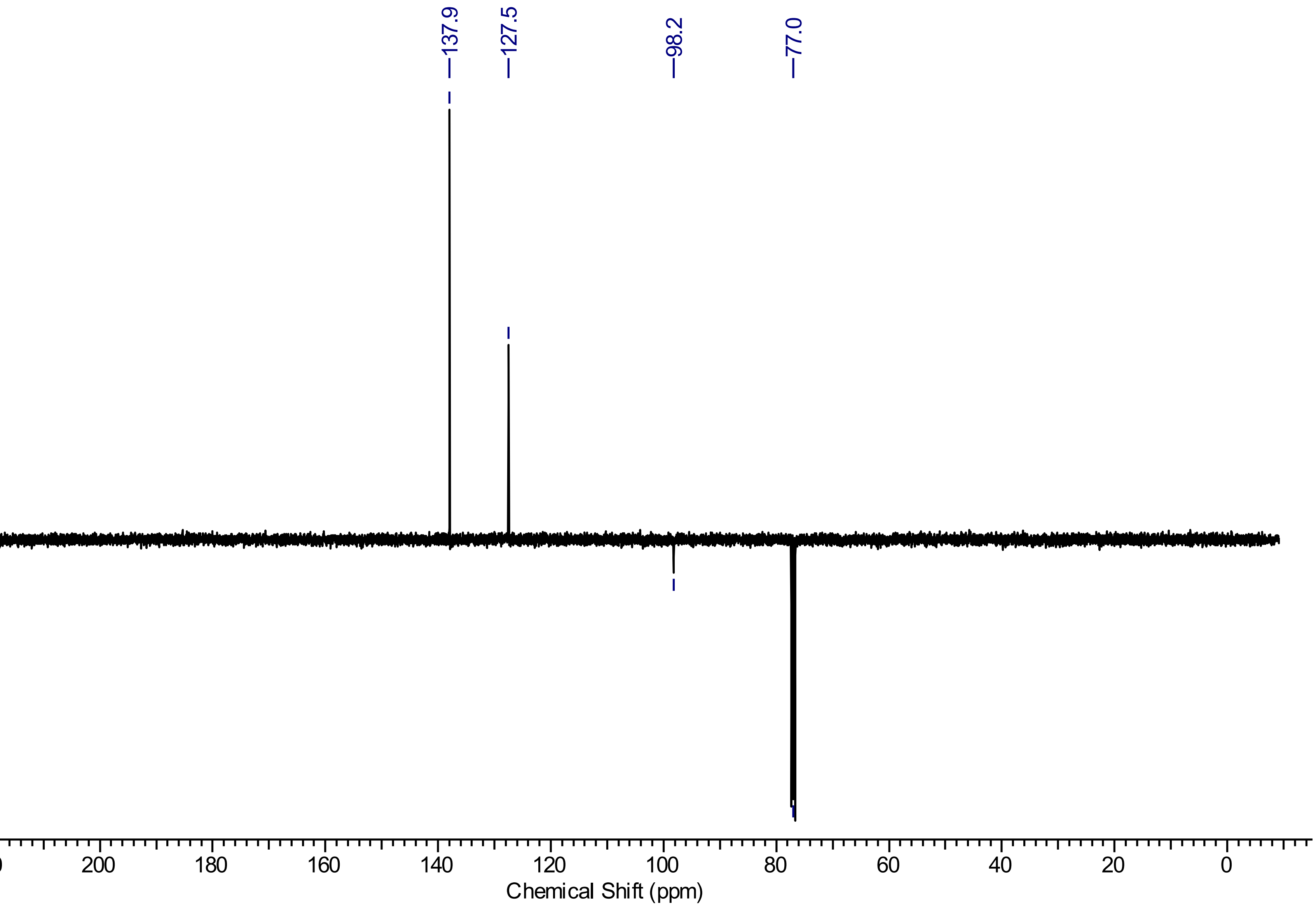

**10b**: $^{19}$F NMR (377 MHz, $CDCl_3$)

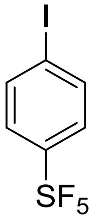


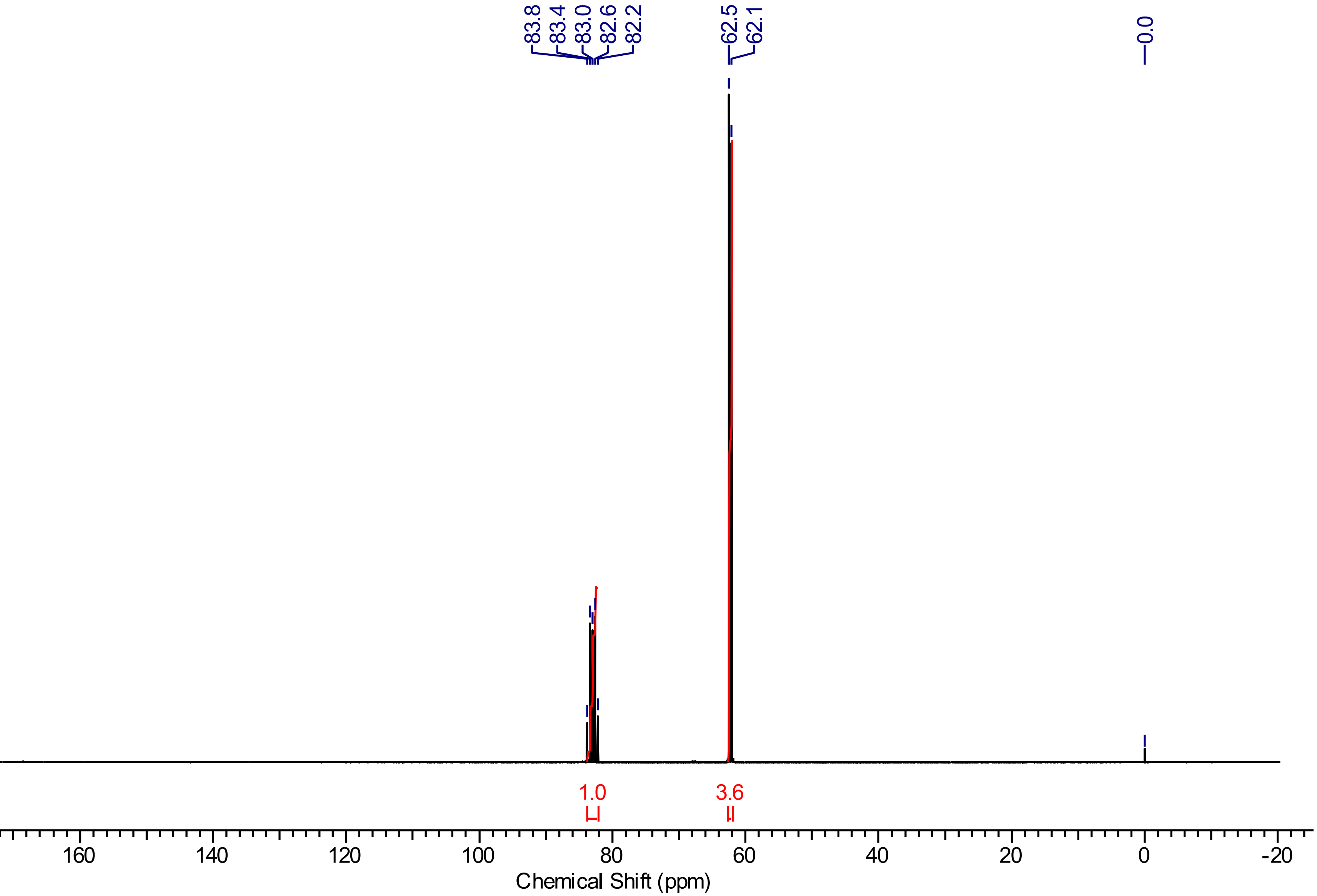

**10c**: $^{1}$H NMR (400 MHz, $CDCl_3$)

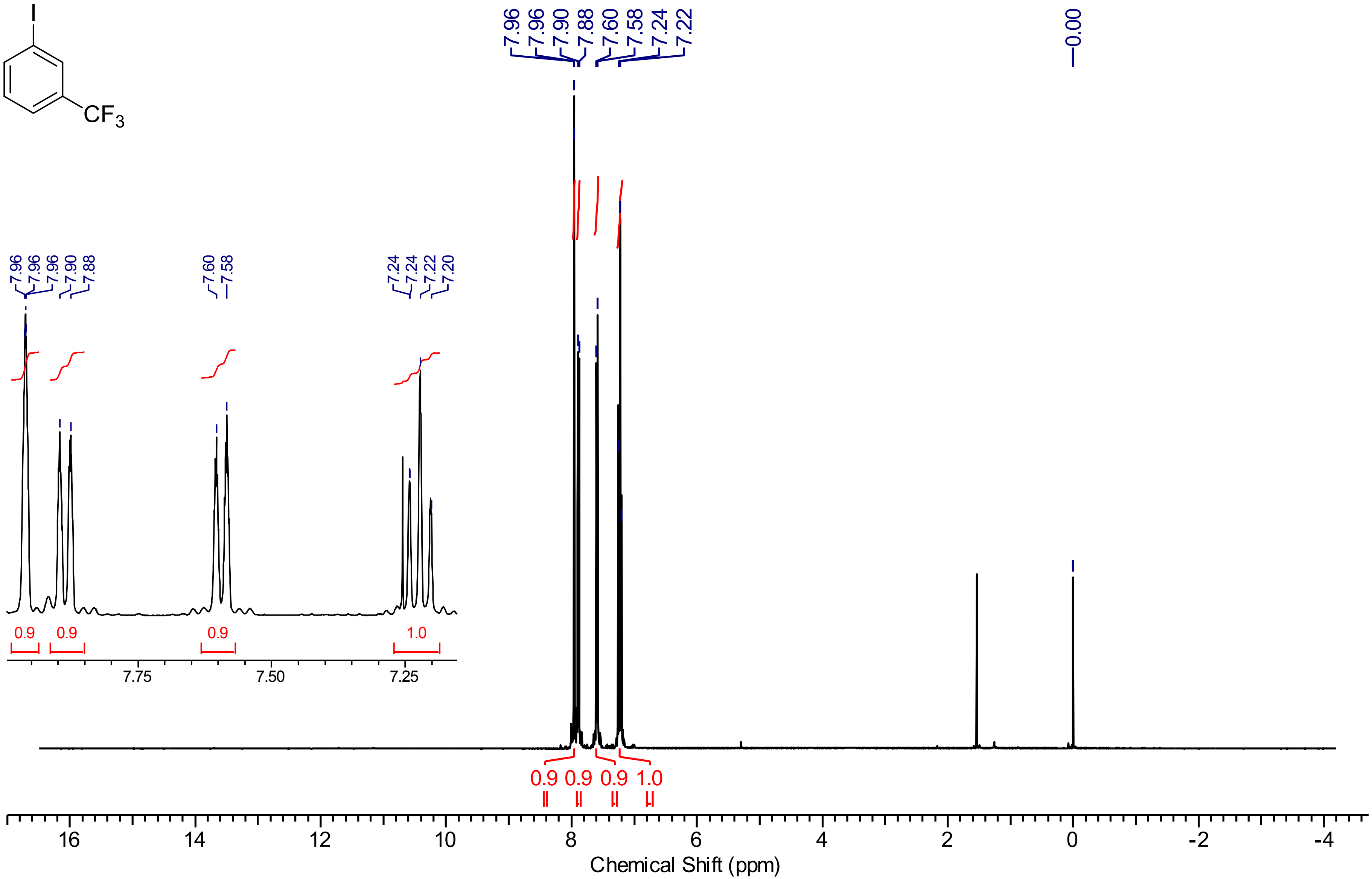

**10c**: $^{13}$C NMR (101 MHz, $CDCl_3$)

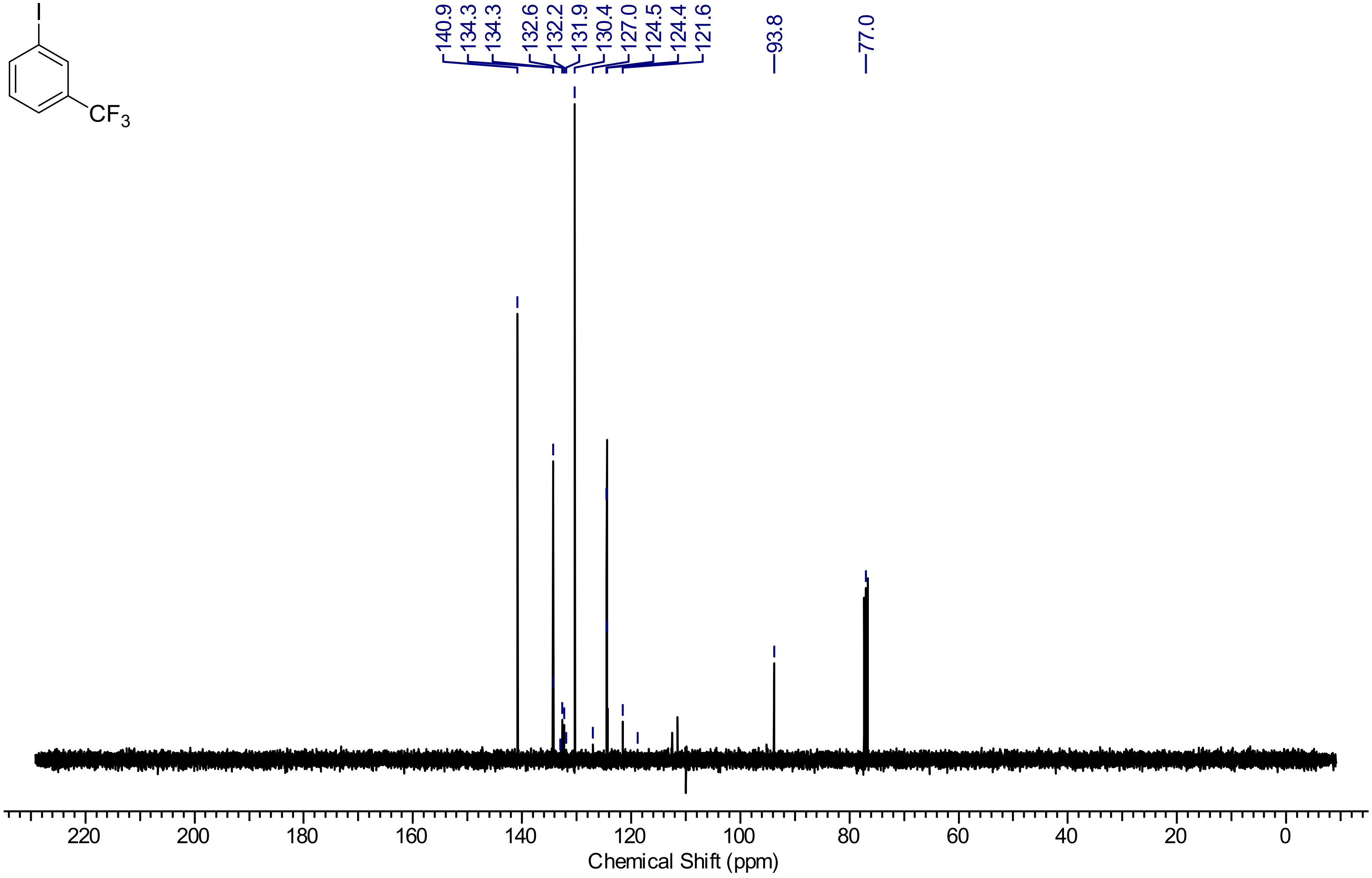

**10c**: $^{19}$F NMR (376 MHz, $CDCl_3$)

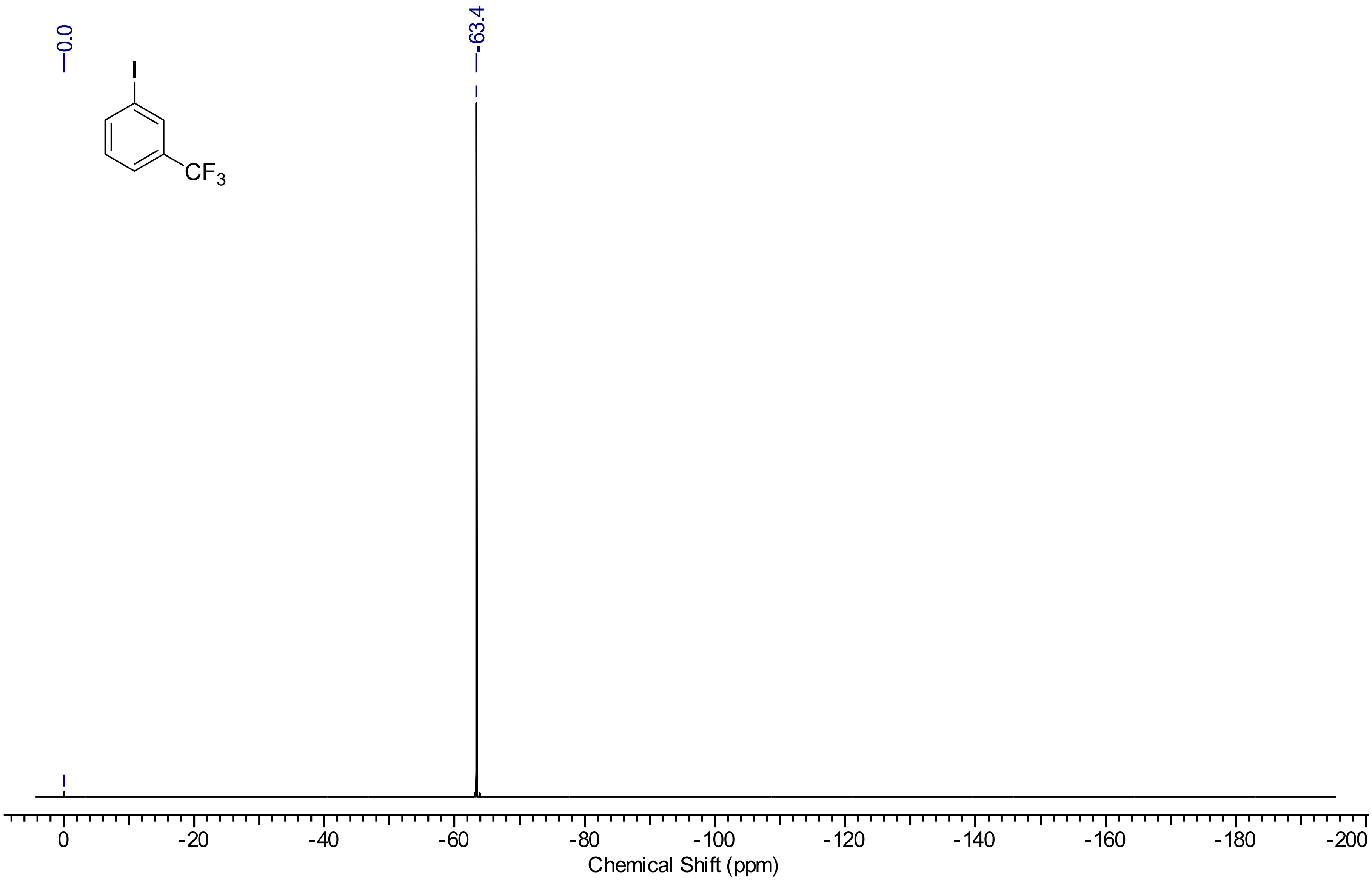

**10d**: $^{1}$H NMR (400 MHz, $CDCl_3$)

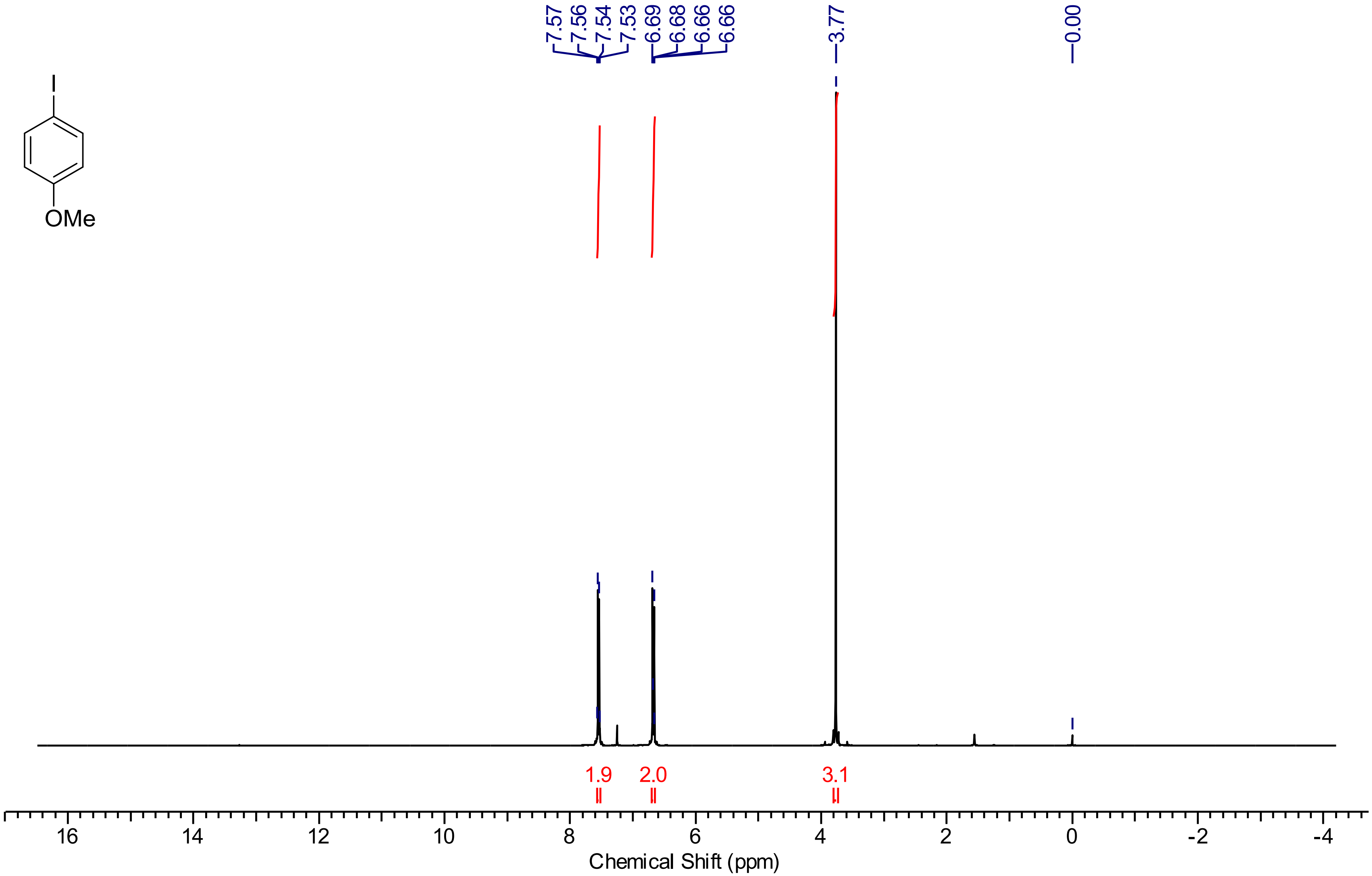

**10d**: $^{13}$C NMR (101 MHz, $CDCl_3$)

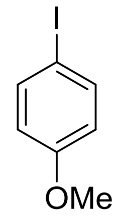

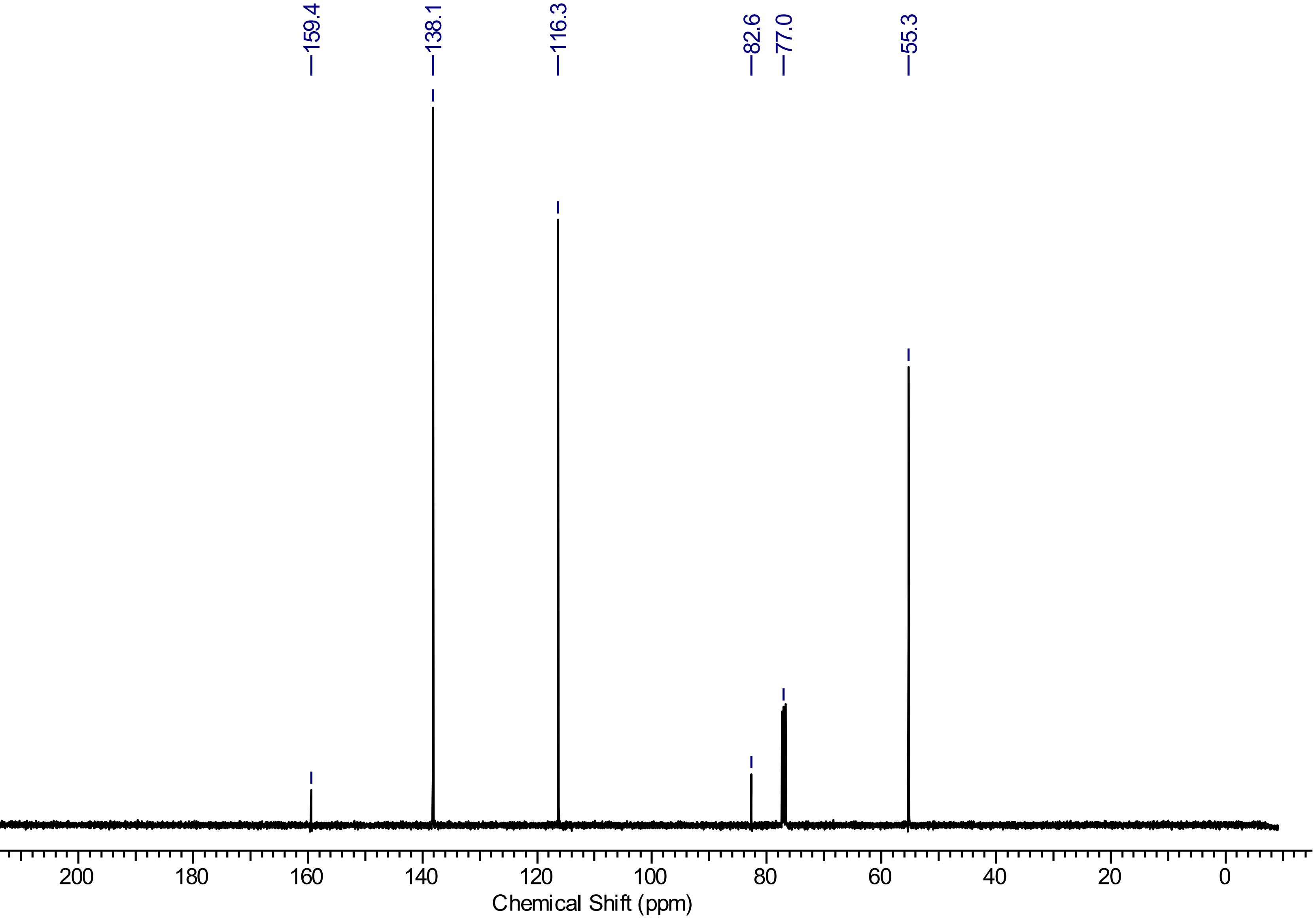

**10e**: $^1$H NMR (400 MHz, $CDCl_3$)

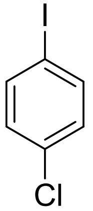


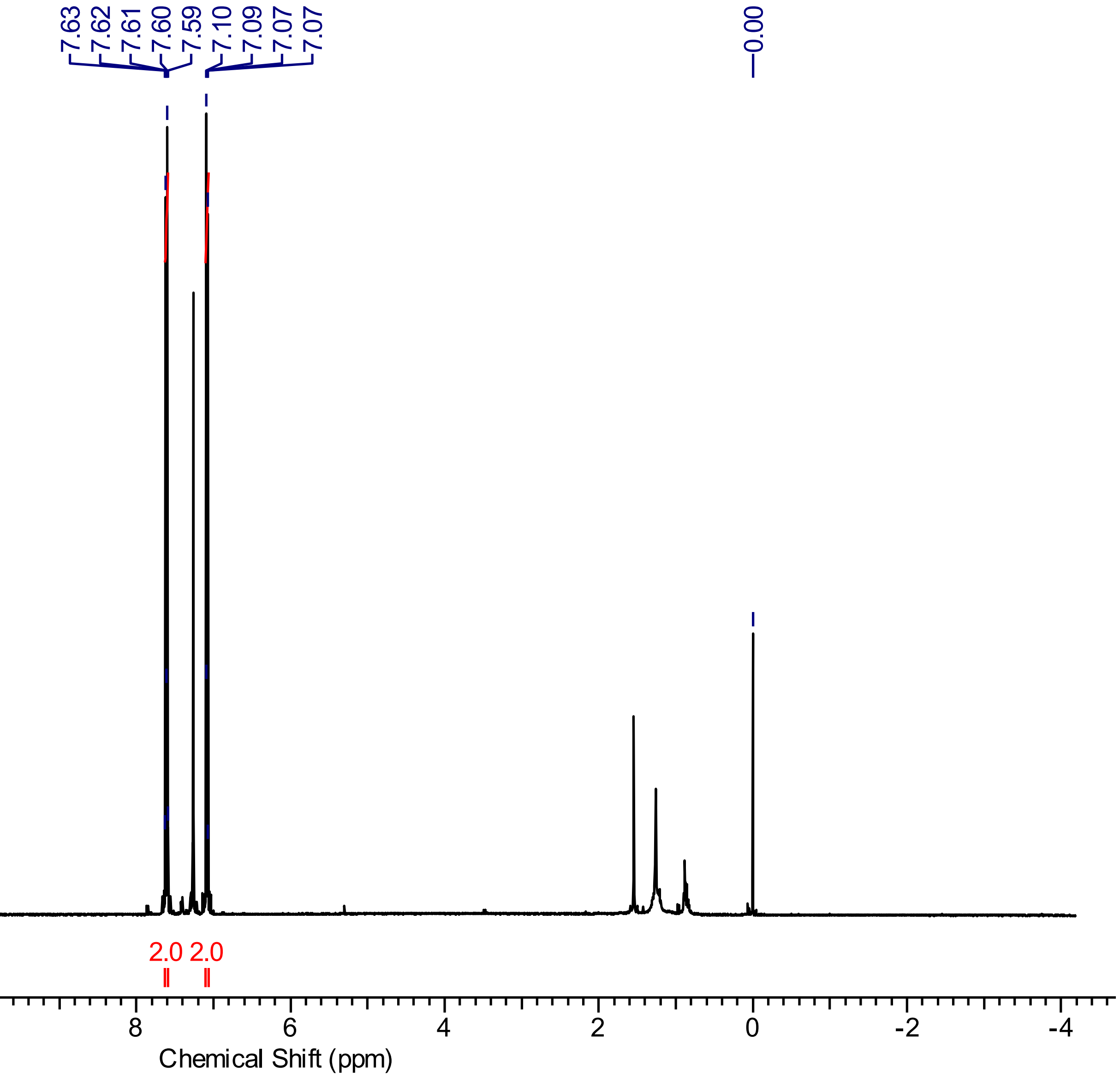

**10e**: $^{13}$C NMR (101 MHz, $CDCl_3$)

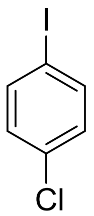


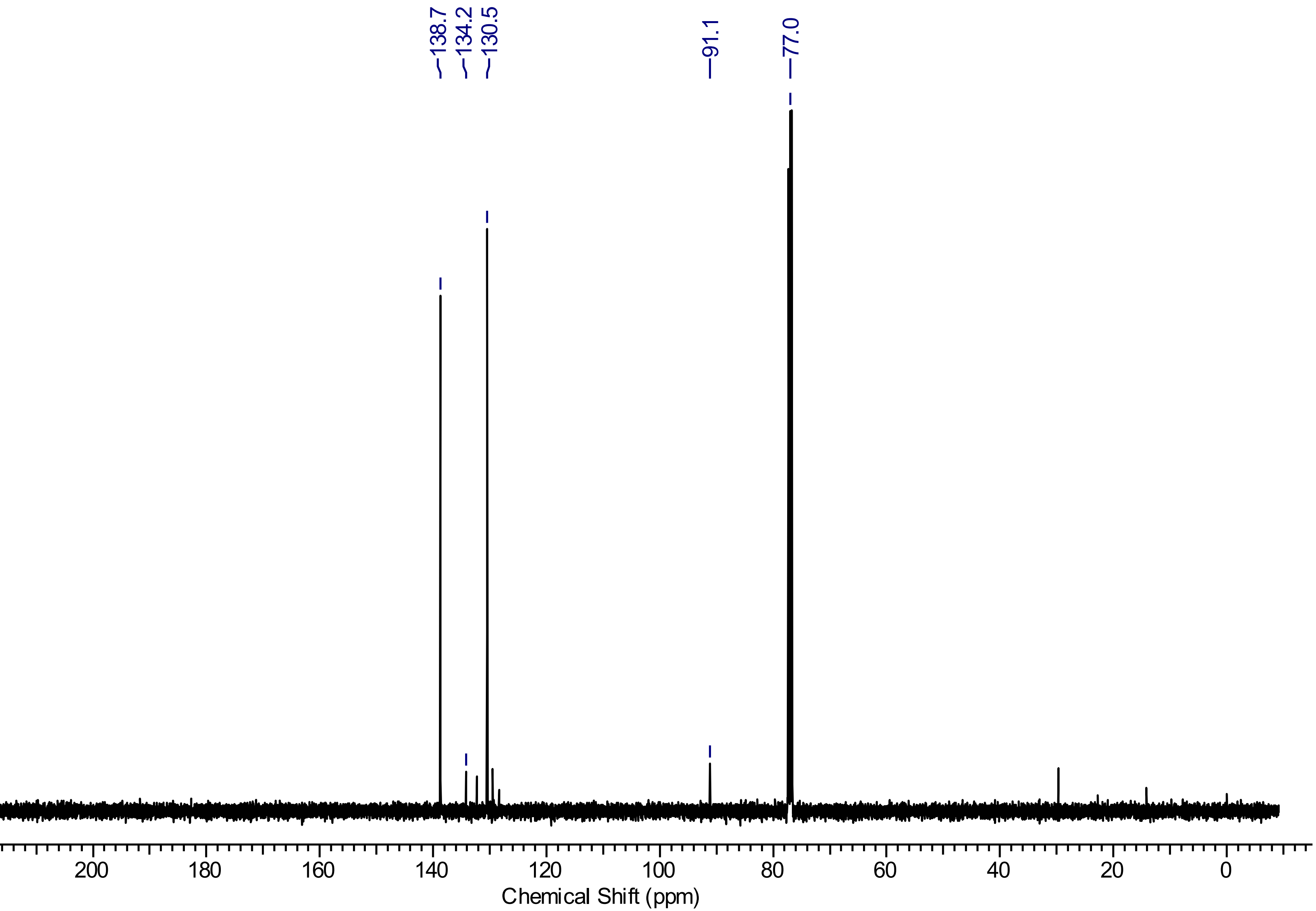

**10f**: $^{1}$H NMR (400 MHz, $CDCl_3$)

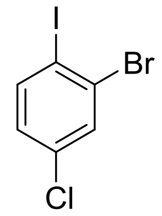


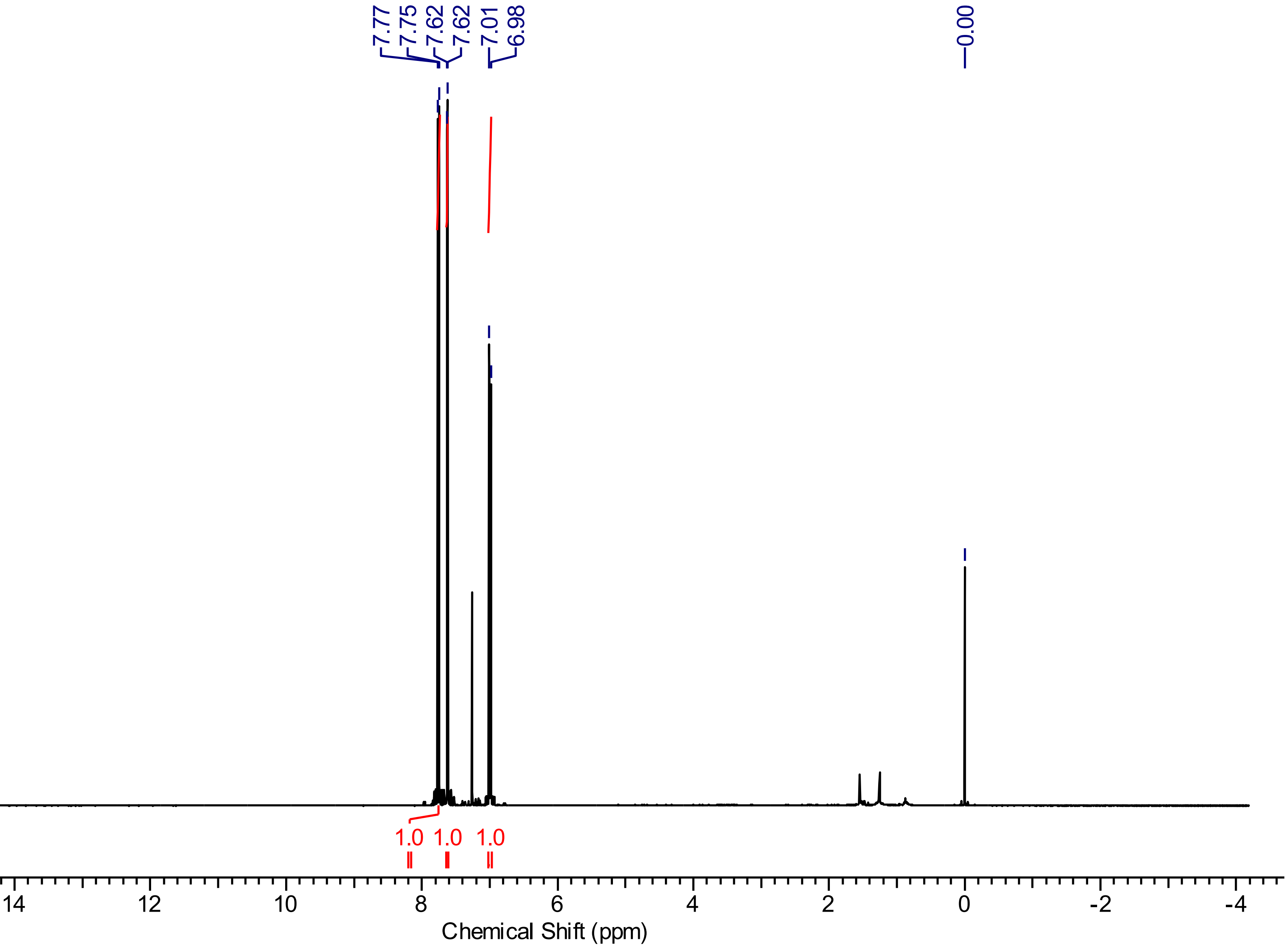

**10f**: $^{13}C$ NMR (101 MHz, $CDCl_3$)

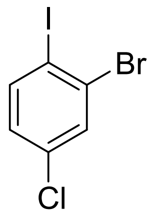


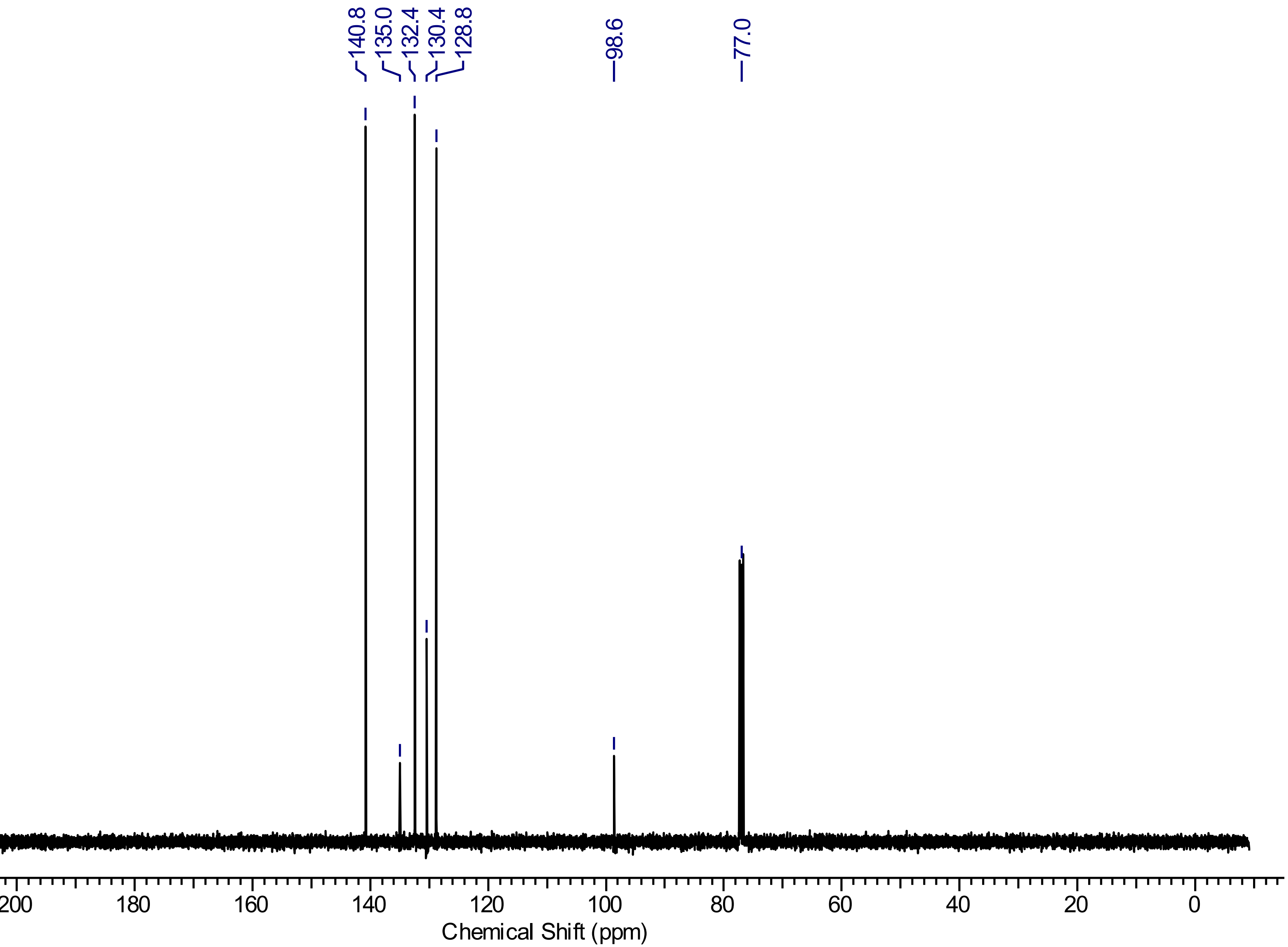

**10g**: $^{1}$H NMR (400 MHz, $CDCl_3$)

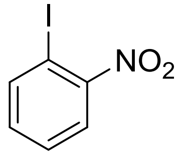

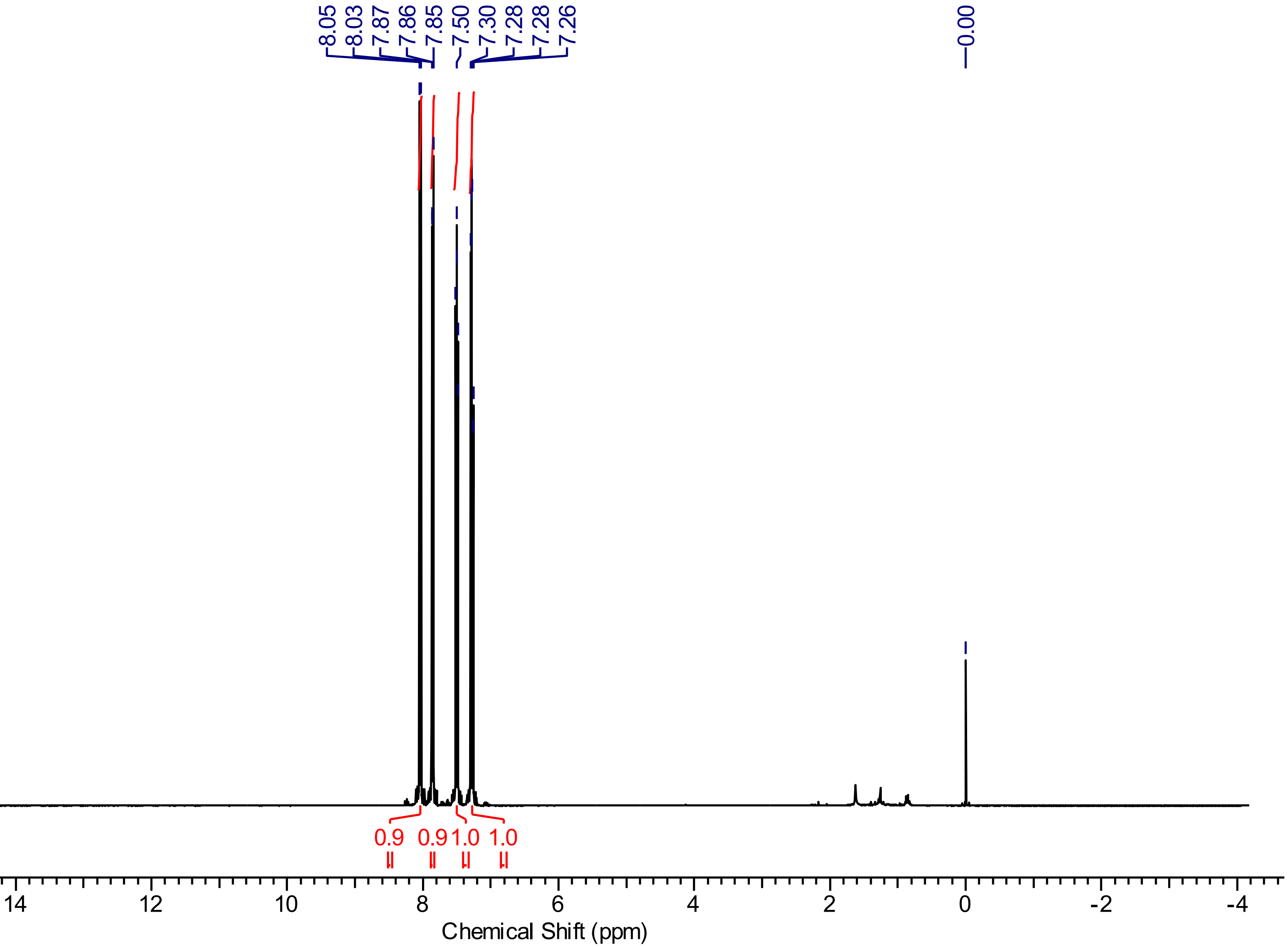

**10g**: $^{13}$C NMR (101 MHz, $CDCl_3$)

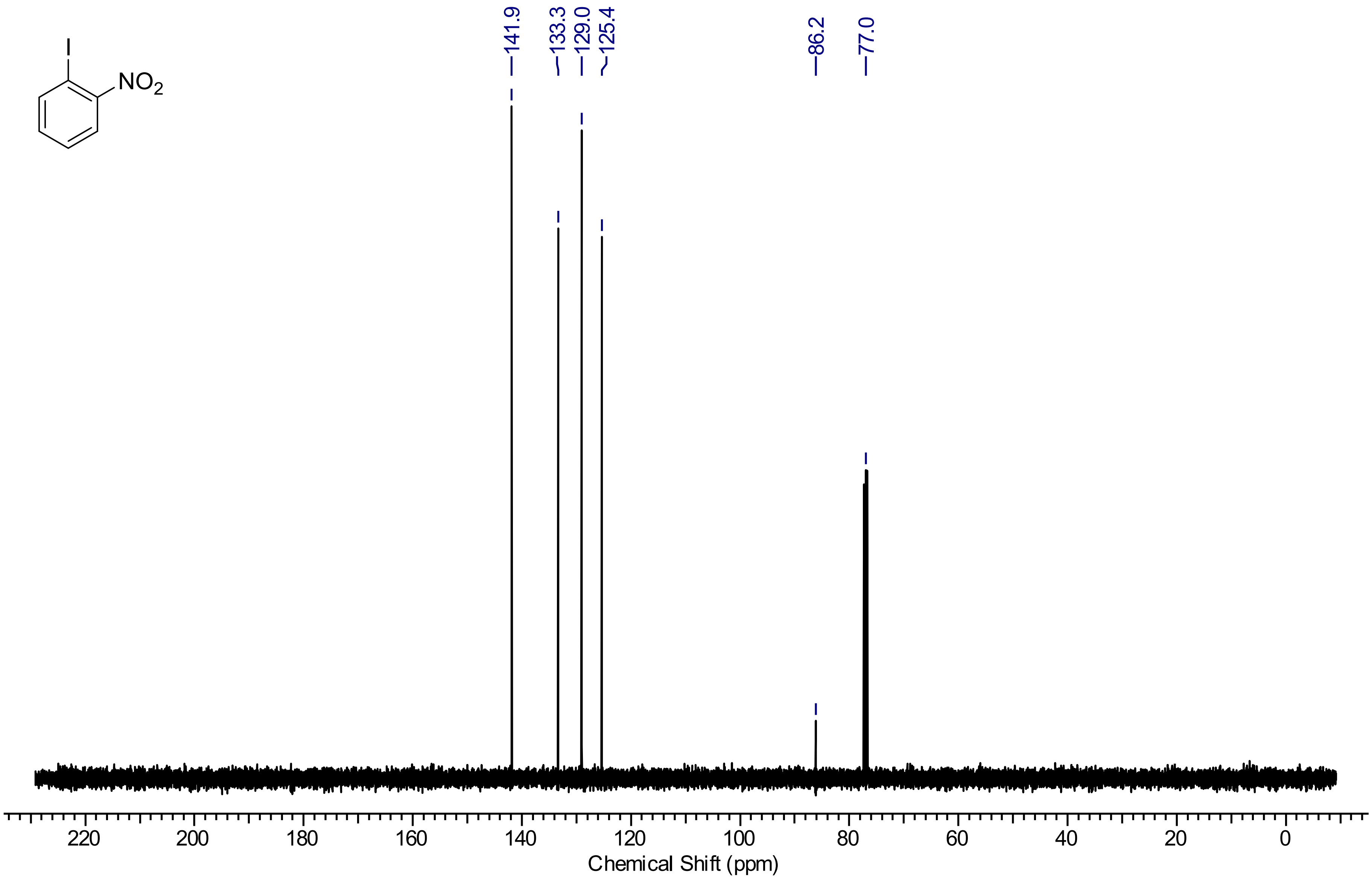

**10i**: $^{1}$H NMR (400 MHz, $CDCl_3$)

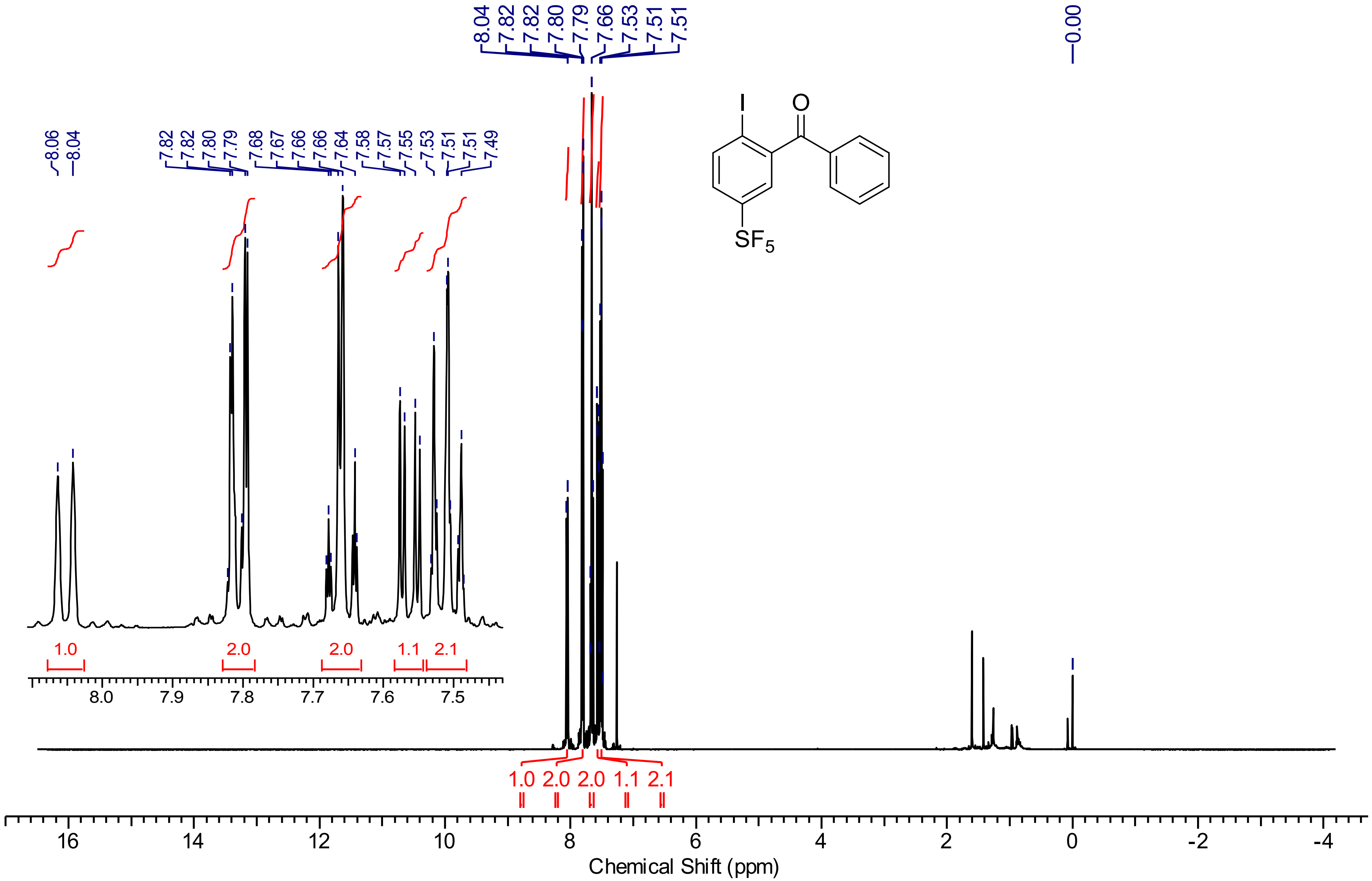

**10i**: $^{13}$C NMR (101 MHz, $CDCl_3$)

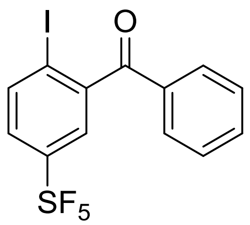

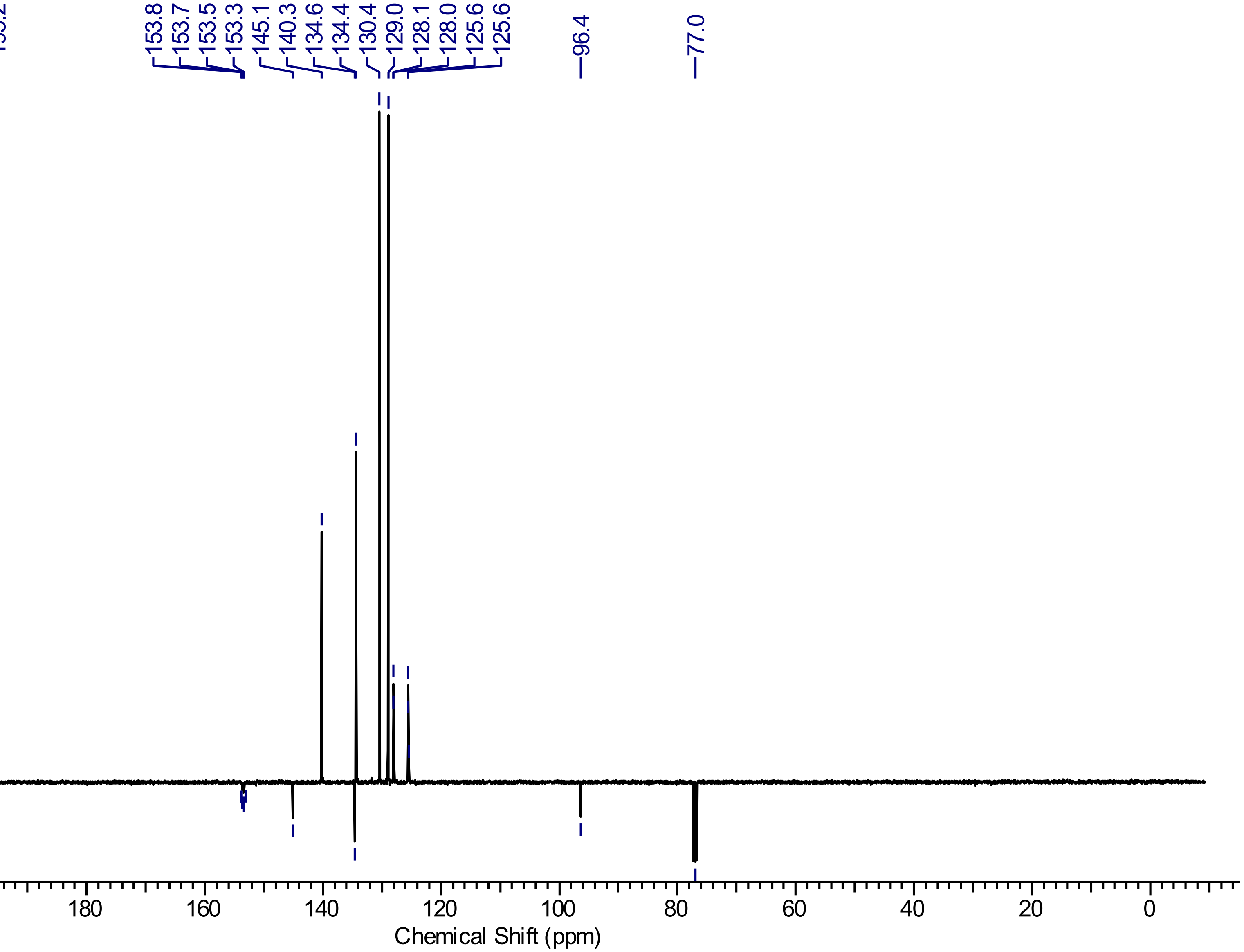

**10i**: $^{19}$F NMR (377 MHz, $CDCl_3$)

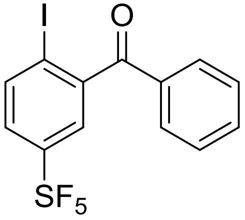


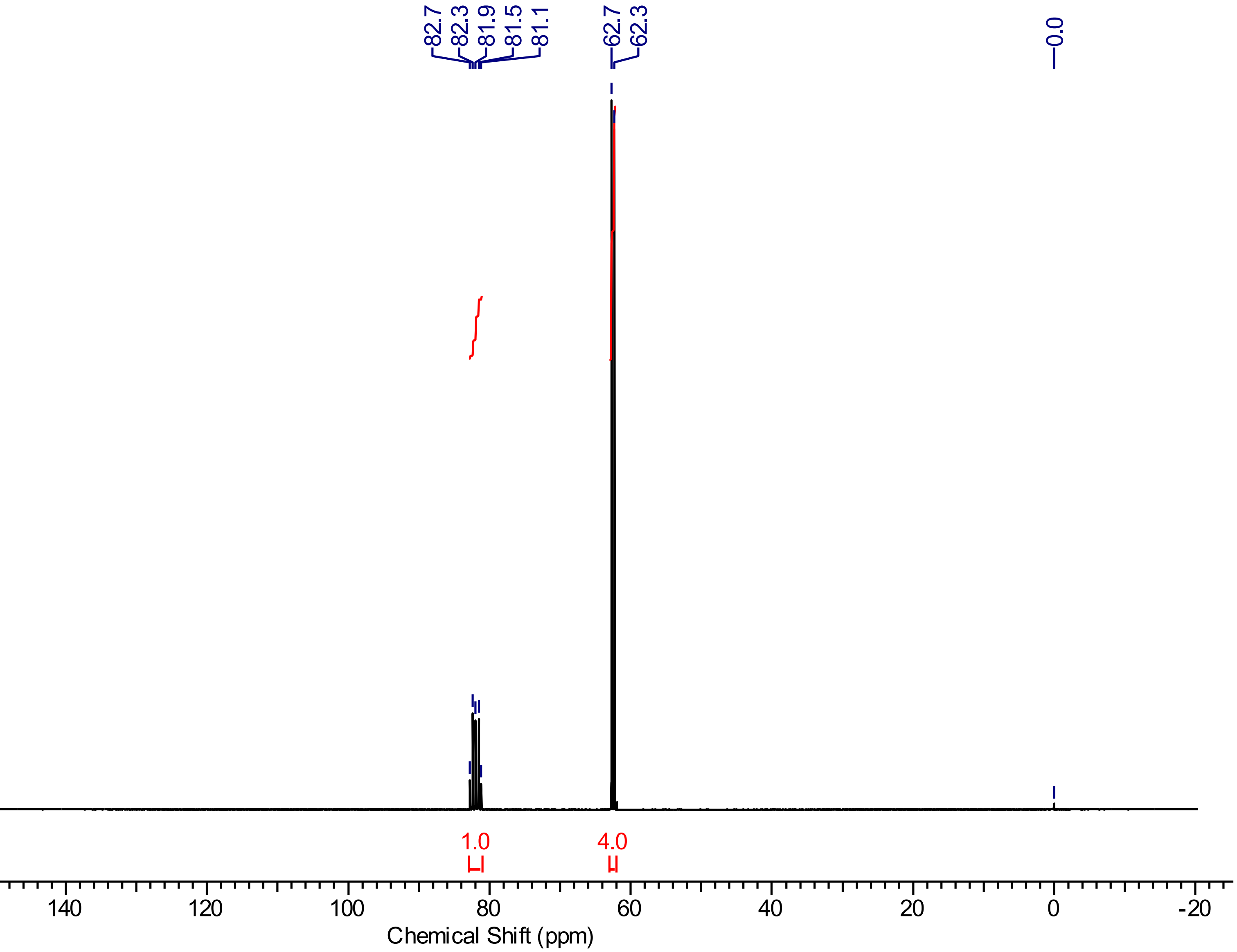

**10i**: HSQC ($CDCl_3$)

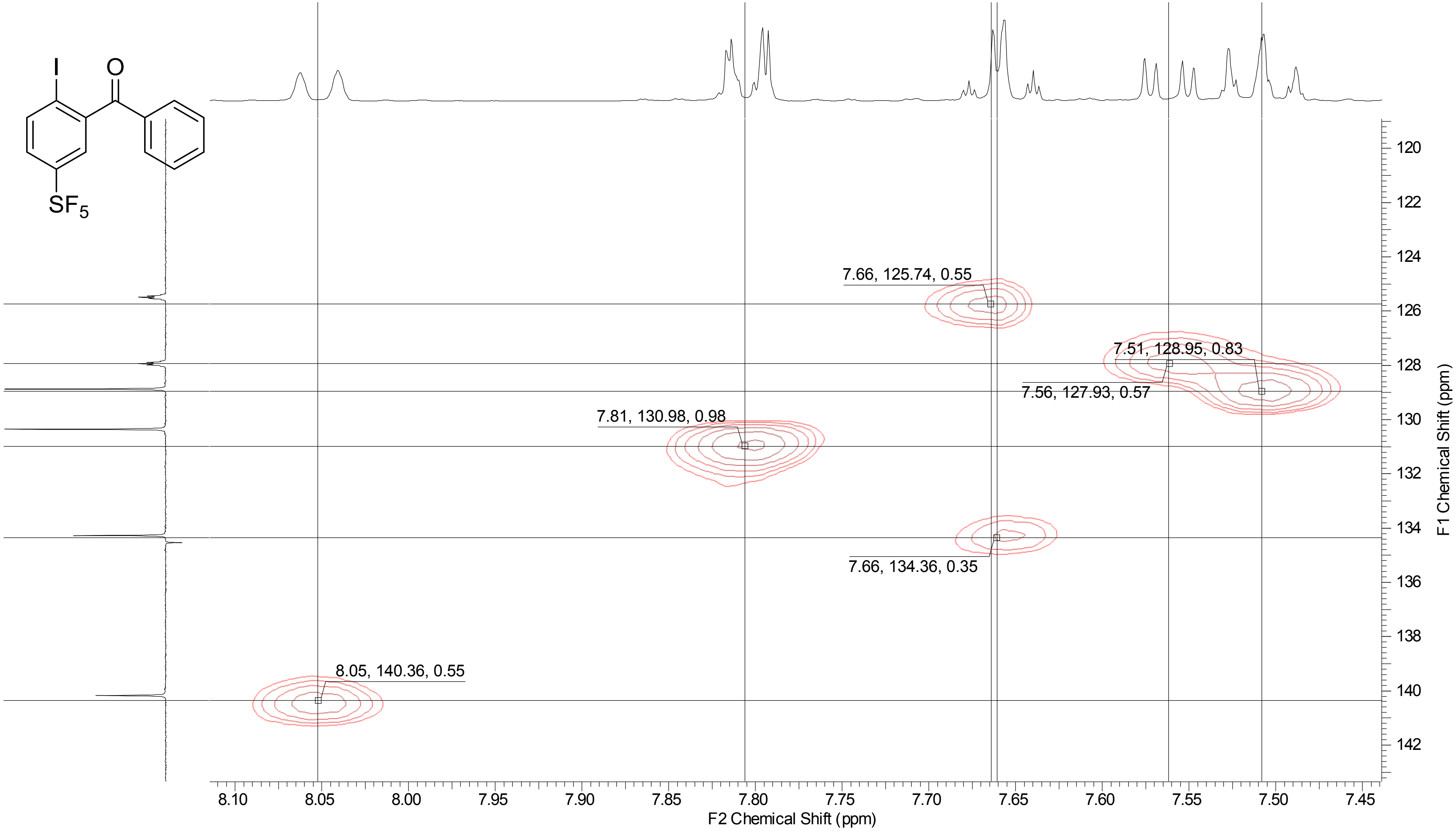

**Pyridinium tetrafluoroborate**: $^{11}B$ NMR (128 MHz, $CD_3CN$)

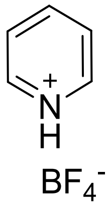


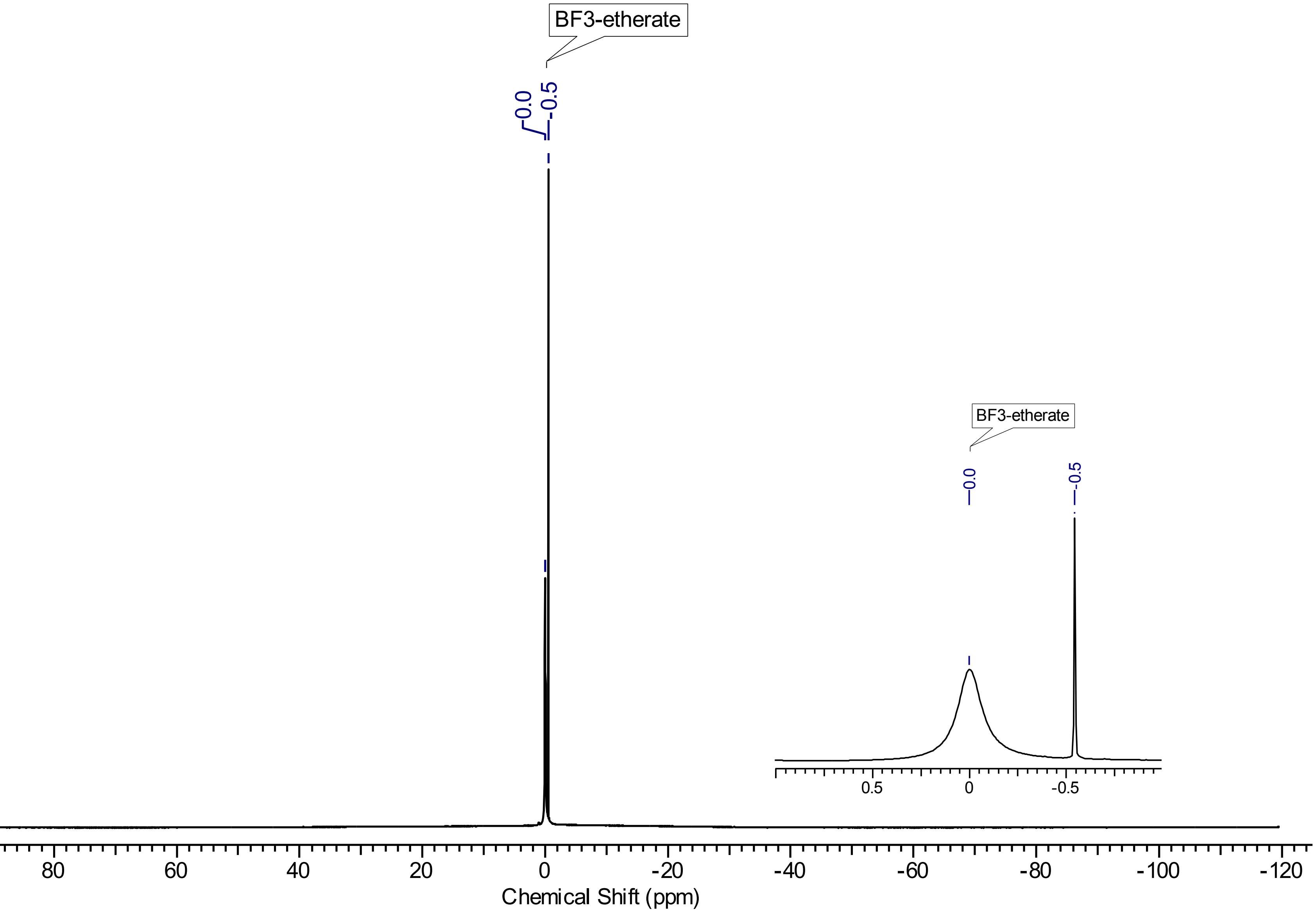

**Pyridinium tetrafluoroborate**: $^{19}$F NMR (376 MHz, $CD_3CN$)

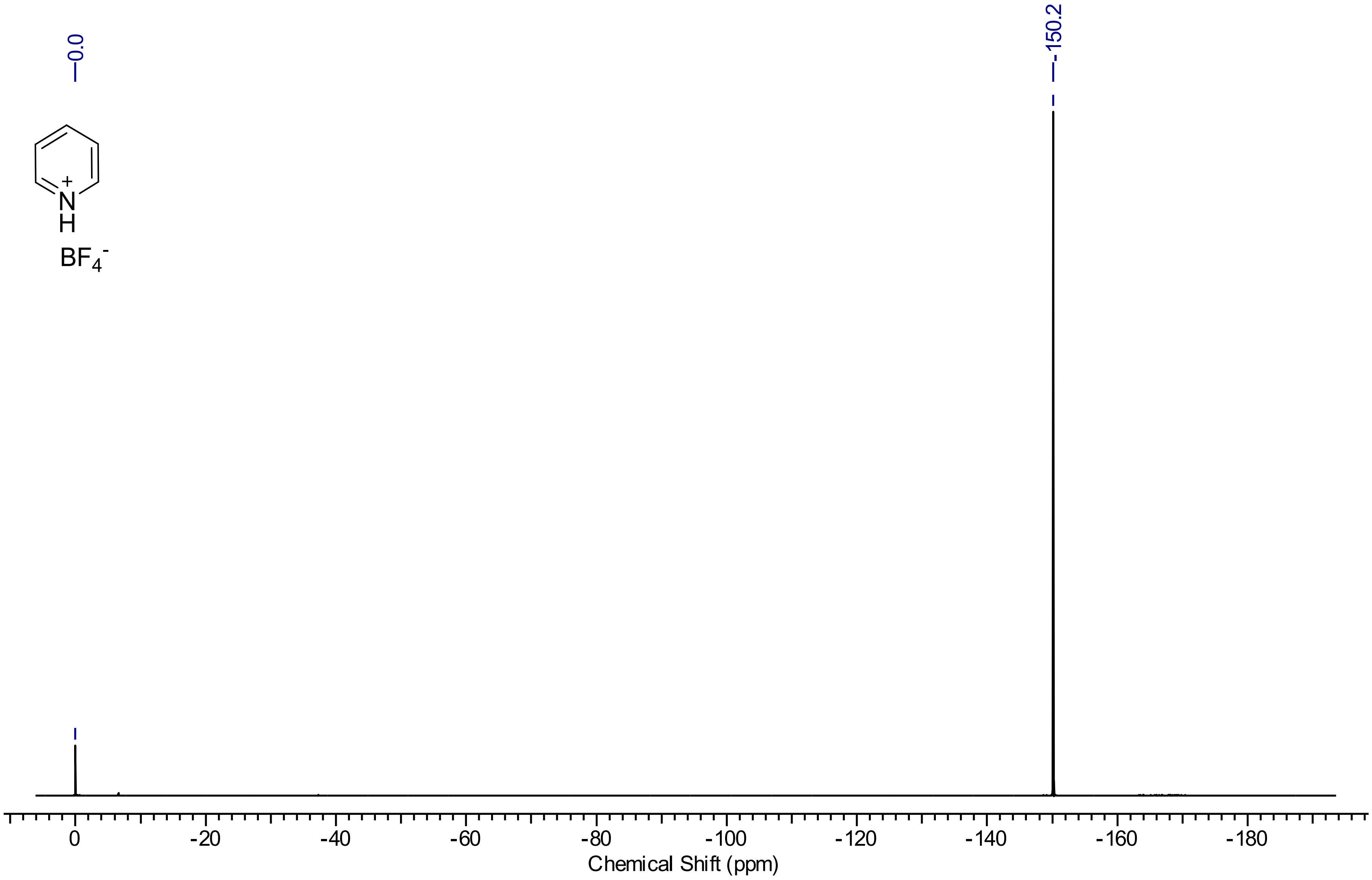

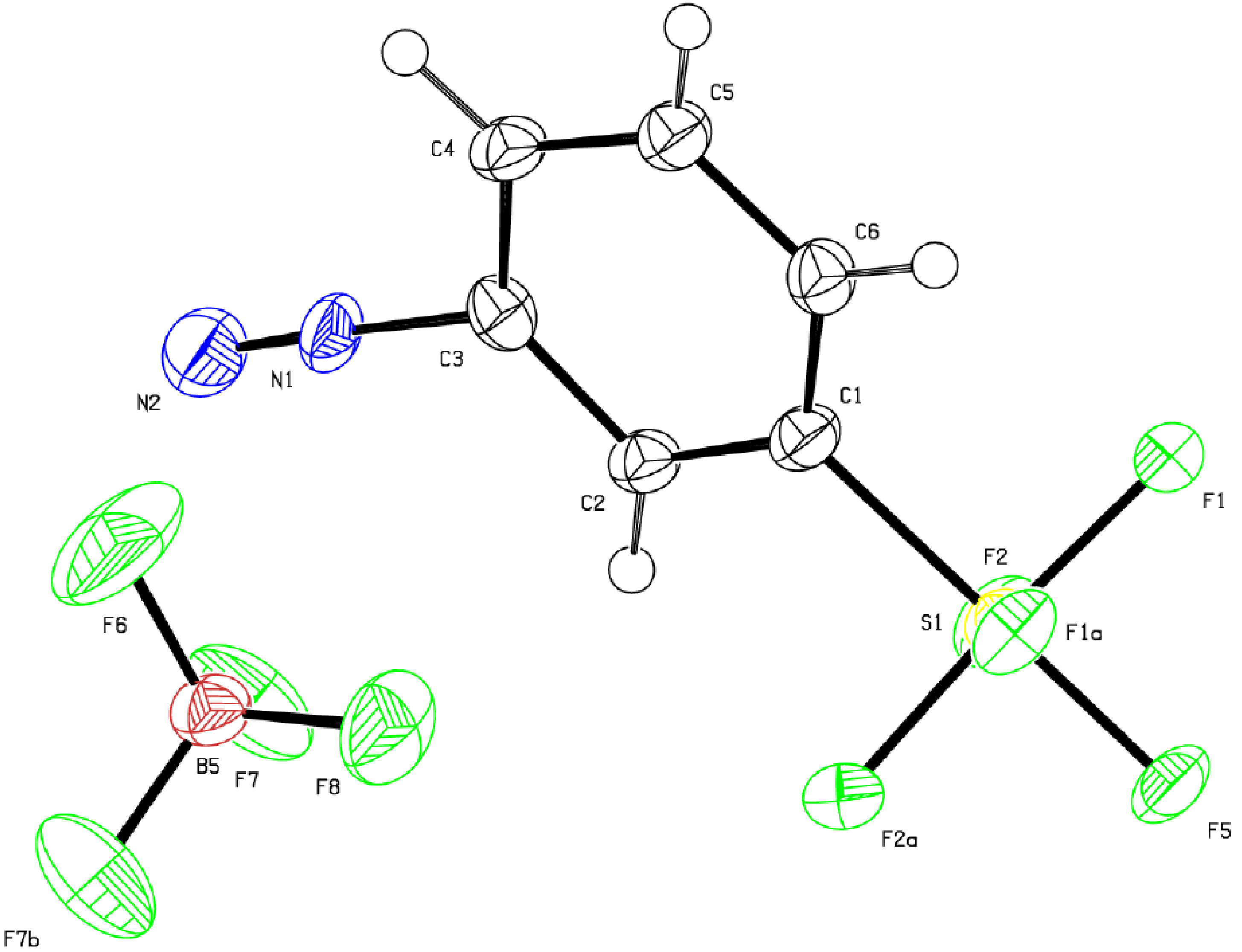


**Figure S12:** X-ray crystal structure of (3-pentafluorosulfanyl)benzenediazonium tetrafluoroborate (**3a**). Thermal ellipsoids are drawn at the 50% probability level.

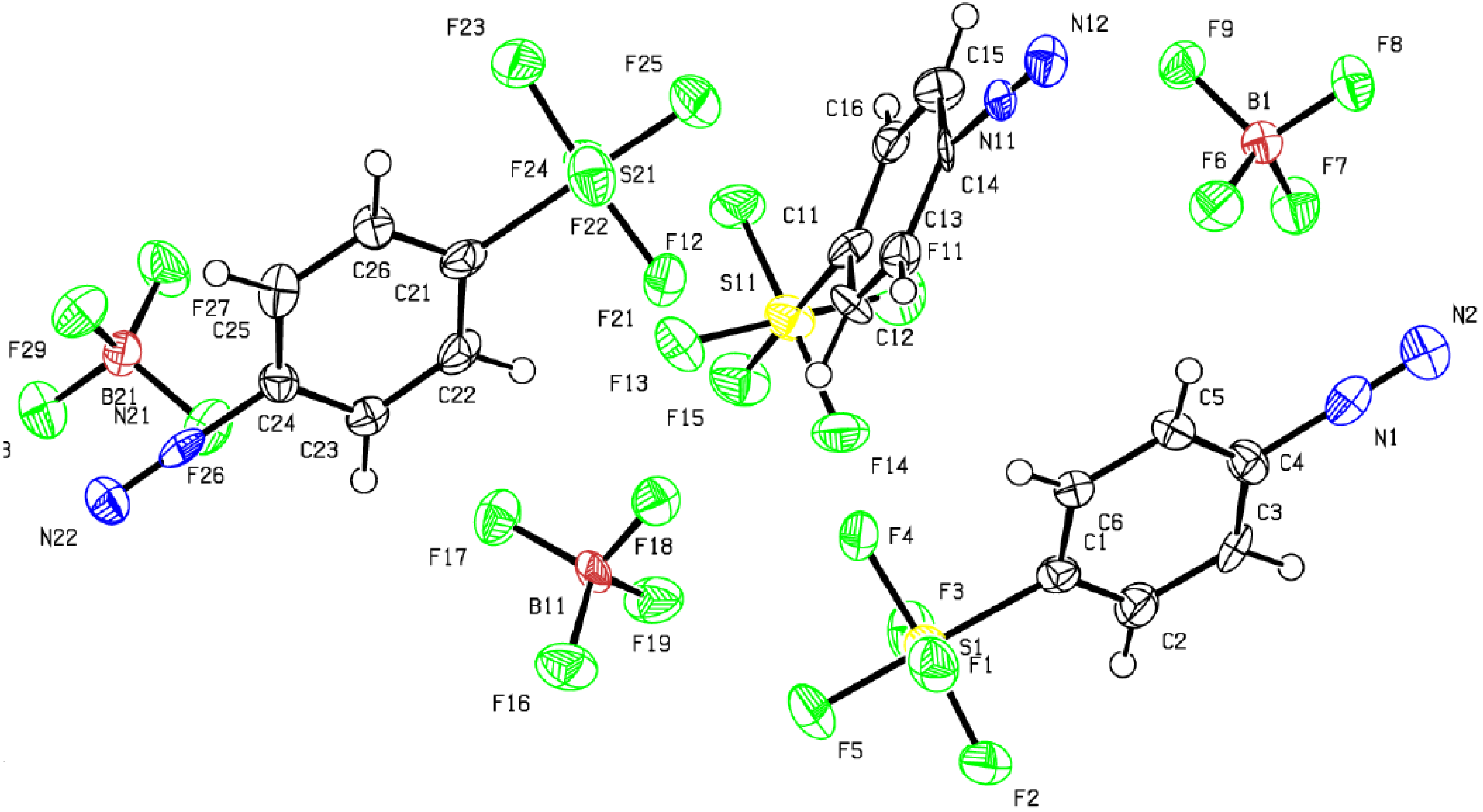


**Figure S13:** X-ray crystal structure of (4-pentafluorosulfanyl)benzenediazonium tetrafluoroborate (**3b**). Thermal ellipsoids are drawn at the 50% probability level.

## References


1. Okazaki, T.; Laali, K. K.; Bunge, S. D.; Adas, S. K. *Eur. J. Org. Chem.* **2014**, 1630-1644. doi:10.1002/ejoc.201301538
2. Heinrich, M. R.; Blank, O.; Ullrich, D.; Kirschstein, M. *J. Org. Chem.* **2007**, *72*, 9609-9616. doi:10.1021/jo701717k
3. Gruner, M.; Pfeifer, D.; Becker, H. G. O.; Radeglia, R.; Epperlein, J. *J. Prakt. Chem.* **1985**, *327*, 63-79. doi:10.1002/prac.19853270109
4. Prechter, A.; Heinrich, M. *Synthesis* **2011**, 1515-1525. doi:10.1055/s-0030-1260006
5. Mo, F.; Jiang, Y.; Qiu, D.; Zhang, Y.; Wang, J. *Angew. Chem., Int. Ed.* **2010**, *49*, 1846-1849. doi:10.1002/anie.200905824
6. Qiu, D.; Jin, L.; Zheng, Z.; Meng, H.; Mo, F.; Wang, X.; Zhang, Y.; Wang, J. *J. Org. Chem.* **2013**, *78*, 1923-1933. doi:10.1021/jo3018878
7. Steuber, E. V.; Elter, G.; Noltemeyer, M.; Schmidt, H.-G.; Meller, A. *Organometallics* **2000**, *19*, 5083-5091. doi:10.1021/om0005912
8. Wada, R.; Oisaki, K.; Kanai, M.; Shibasaki, M. *J. Am. Chem. Soc.* **2004**, *126*, 8910-8911. doi:10.1021/ja047200l
9. Fier, P. S.; Luo, J.; Hartwig, J. F. *J. Am. Chem. Soc.* **2013**, *135*, 2552-2559. doi:10.1021/ja310909q
10. Asachenko, A. F.; Sorochkina, K. R.; Dzhevakov, P. B.; Topchiy, M. A.; Nechaev, M. S. *Adv. Synth. Catal.* **2013**, *355*, 3553-3557. doi:10.1002/adsc.201300741
11. García-López, J.-A.; Greaney, M. F. *Org. Lett.* **2014**, *16*, 2338-2341. doi:10.1021/ol5006246
12. Ratniyom, J.; Dechnarong, N.; Yotphan, S.; Kiatisevi, S. *Eur. J. Org. Chem.* **2014**, 1381-1385. doi:10.1002/ejoc.201301634
13. Bowden, R. D.; Comina, P. J.; Greenhall, M. P.; Kariuki, B. M.; Loveday, A.; Philp, D. *Tetrahedron* **2000**, *56*, 3399-3408. doi:10.1016/s0040-4020(00)00184-8
14. Erb, W.; Hellal, A.; Albini, M.; Rouden, J.; Blanchet, J. *Chem. Eur. J.* **2014**, *20*, 6608-6612. doi:10.1002/chem.201402487
15. Barluenga, J.; González, J. M.; Campos, P. J.; Asensio, G. *Angew. Chem., Int. Ed.* **1985**, *24*, 319-320. doi:10.1002/anie.198503191
16. da Ribeiro, R.; Esteves, P.; de Mattos, M. *Synthesis* **2011**, 739-744. doi:10.1055/s-0030-1258429
17. Jakab, G.; Hosseini, A.; Hausmann, H.; Schreiner, P. *Synthesis* **2013**, 1635-1640. doi:10.1055/s-0033-1338468
18. Dudnik, A. S.; Chernyak, N.; Huang, C.; Gevorgyan, V. *Angew. Chem., Int. Ed.* **2010**, *49*, 8729-8732. doi:10.1002/anie.201004426
19. Smith, W. B.; Ho, O. C. *J. Org. Chem.* **1990**, *55*, 2543-2545. doi:10.1021/jo00295a056
20. Welch, J. T.; Lim, D. S. *Bioorg. Med. Chem.* **2007**, *15*, 6659-6666. doi:10.1016/j.bmc.2007.08.012
21. Umemoto, T.; Garrick, L. M.; Saito, N. *Beilstein J. Org. Chem.* **2012**, *8*, 461-471. doi:10.3762/bjoc.8.53
